# An efficient algorithm for the stochastic simulation of the hybridization of DNA to microarrays

**DOI:** 10.1186/1471-2105-10-411

**Published:** 2009-12-10

**Authors:** Erdem Arslan, Ian J Laurenzi

**Affiliations:** 1 Department of Chemical Engineering, Lehigh University, Bethlehem, PA, USA

## Abstract

**Background:**

Although oligonucleotide microarray technology is ubiquitous in genomic research, reproducibility and standardization of expression measurements still concern many researchers. Cross-hybridization between microarray probes and non-target ssDNA has been implicated as a primary factor in sensitivity and selectivity loss. Since hybridization is a chemical process, it may be modeled at a population-level using a combination of material balance equations and thermodynamics. However, the hybridization reaction network may be exceptionally large for commercial arrays, which often possess at least one reporter per transcript. Quantification of the kinetics and equilibrium of exceptionally large chemical systems of this type is numerically infeasible with customary approaches.

**Results:**

In this paper, we present a robust and computationally efficient algorithm for the simulation of hybridization processes underlying microarray assays. Our method may be utilized to identify the extent to which nucleic acid targets (e.g. cDNA) will cross-hybridize with probes, and by extension, characterize probe robustnessusing the information specified by MAGE-TAB. Using this algorithm, we characterize cross-hybridization in a modified commercial microarray assay.

**Conclusions:**

By integrating stochastic simulation with thermodynamic prediction tools for DNA hybridization, one may robustly and rapidly characterize of the selectivity of a proposed microarray design at the probe and "system" levels. Our code is available at http://www.laurenzi.net.

## Background

Presently, there are several high throughput methods of quantifying changes in gene expression including oligonucleotide microarrays, quantitative realtime PCR (qPCR) and "next generation sequencing" (e.g. [1]). Of these, high density oligonucleotide microarrays are arguably the most important tools for genomic investigation. Although next generation sequencing is a promising alternative to microarrays for genome-scale expression profiling, and exhibit more sensitivity in the low-expression limit [2,3], microarray technology is substantially less expensive and the resulting data sets require much less information processing. Moreover, microarrays have substantially higher throughput than qPCR.

Microarrays consist of DNA probes (reporters) which are orderly arranged on a glass slide. Probes may be attached to (or synthesized from) the slide surface via (1) mask-dependent [4-9] or maskless photolithographic DNA synthesis technology [10,11], or (2) robotic printing of PCR products or synthetic oligomers [12]. The first two of these methods yield oligonucleotide arrays (e.g. Affymetrix GeneChips), which are more reliable than PCR product-based arrays; they comprise the majority of commercial arrays by market share. Thus, we restrict our considerations to microarrays of these types.

Each probe is designed to hybridize with a specific cell-derived and fluorolabeled DNA species such as cDNA or cRNA [6,13]. If these targets originate from mRNA, then the fluorescence associated with each microarray feature is assumed to be proportional to the amount of each transcript [14-16]. However, in recent years, this assumption has been called into question. Several studies have shown that different microarray assays yield different results when used to quantify differences in expression (e.g. [17]). Moreover, significant differences have also been reported among the results of microarray and qPCR assays [18-21]. Although the MicroArray Quality Control consortium confirmed a "high level of interplatform concordance in terms of genes identified as differentially expressed" for several commercial microarrays targeting the human transcriptome in 2006 [22], the reliability of many other array designs and experimental protocols have not been characterized systematically.

Theoretical analyses of smaller systems have suggested that problems with probe reliability are thermodynamic in origin [23,24]. Sequence and GC content heuristics are commonly employed in the design of probes [25,26]. However, these heuristics do not preclude the possibility of finite lengths of complementary sequence between probe candidates and non-target ssDNA. Consequently, all probe-ssDNA interactions will exhibit favorable energetics of interaction to some extent, and there will always be a finite amount of *cross-hybridization *between probes and non-target cDNA. However, experimental identification and quantification of the relative amounts of correctly-hybridized and cross-hybridized probes is impractical, since the only measurement of an array reader is the fluorescence associated with each microarray feature.

### Chemical Dynamics of DNA Hybridization

However, the relative amounts of these species may be quantified using population-balances combined with an appropriate thermodynamic model of hybridization. Consider a microarray with *N*_*P *_oligonucleotide probes that target many if not all of the *N*_*T *_solution-phase ssDNAs (e.g. cDNA) The hybridization network may be written as(1)

where ℓ ∈ (0, *N*_*T*_] and *m *∈ (0, *NP*]. Note that *N*_*P *_and *N*_*T *_needn't be equal, since an array needn't measure the expression levels of all transcripts (*N*_*p *_*< N*_*T*_). Most arrays have multiple reporters per target [27]. The deterministic time evolution of the process is completely specified by the following chemical population balance equations(2)

In Eq. 2, *V *is the volume of cDNA solution added to the array,  is the population of unhybridized probes of type *m *(*m *= 1 ... *N*_*P*_),  is the population of unhybridized transcripts of type ℓ (*m *= 1 ... *N*_*P*_), and  is the population of hybrids composed of probe *m *and transcript ℓ. Before the addition of cDNA to the array, there are *N *unhybridized probes per feature and  molecules of each cDNA ℓ. Thus, the initial conditions of this system of *N*_*P *_+ *N*_*T *_+ *N*_*P *_*N*_*T *_ordinary differential equations are  (*t *= 0) = *N*, (*t *= 0) =  and  (*t *= 0) = 0. Numerical solution of Eq. 2 until *t *~∞ yields the equilibrium populations of desired hybrids (between probes and their targets) as well as cross-hybrids. Unfortunately, this deterministic approach exhibits certain practical pitfalls. Since the reaction rate constants and cDNA populations vary over many orders of magnitude, Eqs. 2 are "stiff". The size of the hybridization reaction network compounds the problem; typical genomic assays are designed to measure the expression levels of thousands of transcripts. For example, baker's yeast (*S. cerevisiae*) possesses approximately 6,700 genes (*N_T _*= 6700) [28] and humans possess approximately 25,000 [29]. Since most microarrays feature one or more distinct reporters (*N_P _*) for each target, the size of the hybridization network (2*N_P _N_T _*reactions) will be enormous. For these genomes, Eq. 2 represents millions to billions of stiff ordinary differential equations.

### Stochastic Simulation

Alternately, one may utilize the stochastic approach to chemical kinetics, which underlies the aforementioned rate equations [30,31]. To begin, let us consider the general case where a volume of solution containing *N*_*T *_cDNAs at populations  (ℓ = 1 ... *N*_*T*_) is added to an array with *N*_*P *_surface-bound probes at populations  = *N *(*m *= 1 ... *N*_*P*_), where again, the superscript "0" denotes the initial state. Upon addition of the solution to the slide, unhybridized probes and targets will randomly hybridize in accordance with Eq. 1. Assuming perfect mixing and isothermal hybridization - both ostensibly achieved with most assays - the state of this system may be defined in terms of the populations of unhybridized probes , unhybridized target DNA , hybrids , and the hybridization volume, *V*. The probability of a transition from one state to another is defined by the stoichiometry of Eq. 1. Since ssDNA molecules directly interact to form a dsDNA hybrid,(3)

is the probability that probe *m *will hybridize with cDNA ℓ within the imminent time interval *δt*, and(4)

is the probability that any of the  hybrids composed of probe *m *and cDNA ℓ will dehybridize within the imminent time interval *δt*. We recognize  and  as the rates of the forward and reverse reactions of Eq. 1, respectively. Eqs. 3 and 4 are microphysically valid [32-35] and are in fact the basis of the validity of the aforementioned rate equations (Eq. 2). We refer interested readers to Gillespie's paper on this subject [31].

Eqs. 3 and 4 are the bases of the stochastic simulation algorithms (SSAs) [34,36]. SSAs simulate the time evolution of a chemical process by repetitively (1) selecting a reaction *μ *among a set *M *of potential reactions, (2) selecting the time *τ *until that reaction occurs, (3) updating the state of the system to reflect the occurrence of the selected reaction, and (4) updating the time. Exact SSAs differ only in how the first two steps are implemented. Each methodology features its own memory and speed enhancements and restrictions.

#### Direct Method

The Direct Method samples two exact density functions to obtain the quiescence time and imminent reaction event. The selection rule for the quiescence time is(5)

where *a*_0 _is the sum of all reaction rates and *r*_1 _*~U *(0, 1) (i.e. *r*_1 _is a uniform random number). The selection rule for the imminent event *μ *is(6)

In a microarary hybridization network, these rates are defined by Eqs. 3 and 4, as discussed, such that *μ *∈ [1, 2*N*_*P *_*N*_*T*_].

Although the Direct Method is the faster and more memory efficient of the two SSAs first developed by Gillespie [34], it is numerically intensive when applied to large chemical networks. Since *O*(*M*) operations are required for Eq. 6, simulations with *M *reaction types require *O*(*M*) calculations per time step. Since each probe may potentially bind every target, *M *= 2*N*_*P *_*N*_*T *_for the reaction system described by Eq. 1. Thus, there are *O*(*N*_*P *_*N*_*T*_) operations per time step with the Direct Method. Considering typical values for *N*_*P *_and *N*_*T*_, there will be hundreds of millions of operations per time step in DM simulations of conventional microarrays.

#### Next Reaction Method

In 2000, Gibson & Bruck proposed a new exact SSA called Next Reaction Method. This approach purportedly reduces the number of required calculations per time step from the *O*(*M*) of the Direct Method to *O*(*k *log *M*), where *k *is tantamount to the number of chemical species with which the average chemical species will react [37] (e.g. the number of cross-hybrids per probe). The speed enhancement of this algorithm is most prominent when the reaction network is sparse (*k *≪ *M*), however, it should be much faster than the Direct Method for reaction networks as coupled as Eq. 1, where *k ~N*_*P *_and *M *= 2*N*_*p*_*N*_*T *_. The Next Reaction Method is significantly different than the Direct Method in both its data handling and MC selection rules. First, the absolute times at which all reactions *might *occur ((*τ *_*ν *_- *t*) *~ *Exponential(*α*_*ν*_), *ν *∈ [1, *M*]) are selected by MC, by contrast to the MC selection of just one quiescence time in the Direct Method. One may show that the smallest of these times (*τ*_*μ*_) is the time at which the next reaction (*μ*) occurs, and that this selection rule is an exact MC selection from the reaction probability density function, like Eqs. 5 and 6 [37].

Another noteworthy difference between the Direct Method and the Next Reaction Method is the employment of a "dependency graph" by the latter. This data structure reduces the number of calculations per reaction event, both for event selection and updating the reaction times. However, the dependency graph requires *O*(*kM*) objects to store the dependencies of reaction rates upon the populations of reactants and products shared with other reactions. For microarray hybridization, this translates to a storage requirement of *O*() reaction objects, which is prohibitively large for genome-sized microarray simulations; Even current supercomputers with terabytes of memory cannot meet these requirements.

## Results

### Algorithm

In simulations of microarray hybridization, the extreme computational burden of the Direct Method and the memory burden of the Next Reaction Method may be alleviated by judicious storage and summation of the terms in Eq. 6. Our algorithm employs a data structure called a *Hybridization Table *(HT) that stores partial sums of the terms in Eq. 6. For this reason, we call our approach the "method of partial sums" (Algorithm 1).

   Initialize ( = *N, j *= 1, 2 ... *N*_*P*_, , *i *= 1, 2 ... *N*_*T *_via the "gold standard", *t*)

   Calculate the auxiliary variables (*α*_*j *_, *j *= 1, 2 ... *NP *(Eq. 7), *ϕ*_*j*_, *j *= 1, 2 ... *N*_*P *_(Eq. 8), *α *and *ϕ *(Eqs. 9, 10)

   **repeat**

      Calculate the total reaction rate *α*_0 _(Eq. 11) and quiescence interval *τ *(Eq. 5)

      **if **the next reaction is a hybridization (Eq. 12) **then**

         Select the hybridizing probe *m *and cDNA ℓ (Eq. 14, 17)

      **else**

         Select the dehybridizing probe *m *and cDNA ℓ (Eq. 20, 21)

      **end if**

      Update populations 

      Update hybridization rates: *α*_*m *_and *α*_*j *≠ *m*_, *j *= 1, 2 ... *N*_*P *_(Eqs. 22, 23)

      Update Dehybridization Rates: *ϕ*_*m*_, *ϕ *(Eqs. 24, 25)

      *t *← *t *+ *τ*

   **until ***P >*0.05 for  = 

Algorithm 1: Method of partial sums for the simulation of the coupled reaction network composed of the hybridizations of *N*_*P *_oligonucleotide probes and *N*_*T *_cDNA species.

#### Hybridization Table

**Figure 1:**
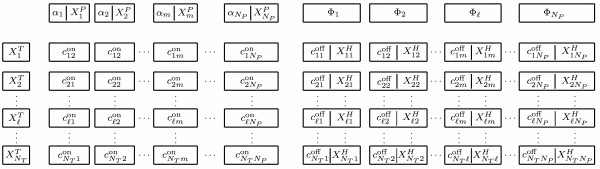
**Structure of the hybridization table (HT)**. Rate constants and populations are stored in such a way as to simplify the calculation of the quantities *α*_*j*_, *ϕ*_*j*_, *α *and Φ (See Eqs. 7, 8, 9, and 10) thereby reducing the number of operations per time step of the simulation from *O*(2*N*_*P *_*N*_*T*_) to *O*(*N*_*P *_+ *N*_*T*_).

The structure of the Hybridization Table is outlined in Fig. 1. In addition to storing the populations of targets {}, probes {}, hybrids {} and rate constants  and  (*m *∈ [1, *N*_*P*_], ℓ ∈ [1, *N*_*T*_]), the table also contains the rates at which probes of type *j *will hybridize with *any *target,(7)

and the rates at which *all hybrids *composed of probes *j *will dissociate,(8)

Two additional quantities are stored in the table: the total rate of hybridization(9)

and the total rate of dehybridization(10)

The total rate of reaction may then be calculated from(11)

Eqs. 7, 8, 9, and 10 effectively subdivide the running sum in Eq. 6 into independently manageable groups of information. This in turn reduces the number of operations required for event selection reaction rate updates.

### Reaction event selection

The selection of the imminent hybridization or dehybridization event is accomplished by summing the terms of Eq. 6 such that the total number of operations in Eq. 6 (including the calculation of *a*_0_) is minimized. We begin by ordering the reactions according to column and row in the HT (Fig. 1) such that . Eq. 6 is then implemented by summing the reaction rates corresponding to each entry in the HT, by column and then by row: first, by hybridization events, and then by dehybridization events.

One begins by identifying whether or not the imminent event will be a hybridization. Since the imminent event is defined by the reaction whose rate causes the sum of *α*_*ν *_to exceed the quantity *r*_2_*α*_0_, and all hybridization reactions precede the dehybridization reactions in the HT, it follows that(12)

If Eq. 12 results in the selection of a hybridization event, *α *>*r*_2_*a*_0_, and one needn't consider the dehybridization rates in the selection of the event to come.

The next step is the selection of the probe (*m*) that will hybridize in the imminent event. Using the notation of the Direct Method and the order of summation, the index of the event to come (*μ*) must be less than or equal to *N*_*P *_*N*_*T*_, where  is the last of the hybridization rates in the table (Fig. 1). Eq. 12 becomes(13)

In essence, the event is selected by summing the rates corresponding to each column of the "hybridization" section of the HT, column by column, followed by row, until the quantity *r*_2_*a*_0 _is exceeded. Noting that the quantity is equal to *α*_*j *_(Eq. 7), Eq. 13 may be simplified to(14)

which defines the probe that will hybridize in the imminent event.

The equation defining the selection of the target may be derived similarly First, we express the sums on the right and left sides of Eq. 13 as(15)

and(16)

Simplifying these expressions, we obtain(17)

Thus, the target (ℓ) in the imminent event may be obtained by summing the quantities  until the quantity  is exceeded.

If the event to come is a dehybridization, then *α *<*r*_2_*a*_0_. Hence, the sum of all hybridization rates may be subtracted from each term in Eq. 6, leaving(18)

where {*a*_*ν *_} are the rates of the dehybridization reactions and we have explicitly noted the fact that all *N*_*P *_*N*_*T *_hybridization rates are contained within *α*. Expressing this in terms of the hybrid indices, we obtain(19)

Again, the selection of the the event may be simplified using the auxiliary variables in the HT. If the probe *m *is released in the imminent event, the value of *m *may be defined by sequential addition of the values of {Φ_*j*_} until the quantity (*r*_2_*a*_0 _- *α*) is exceeded(20)

Note that this also defines the identity of the probe that will dissociate from the target. The cDNA to be released in the dehybridization event (ℓ) may be calculated by subtraction of Eq. 20 from Eq. 19:(21)

The selection rules for hybridization (Eqs. 14, 17) and dehybridization (Eqs. 20, 21) substantially reduce the number of operations required by Eq. 6. Whereas the Direct Method may require 2*N*_*P *_*N*_*T *_operations to select a reaction, our selection rules require at most (*N*_*P *_+ *N*_*T*_) operations. For a genome-sized microarray simulations, with *N*_*P *_*~N*_*T *_*~ *10^4^, our reaction selection approach will be several orders of magnitude faster than those of other algorithms.

#### System state accounting

Upon selection of the imminent event, the populations of the species involved (, , and  for the probe, target, and hybrid, respectively) must be updated in accordance with the stoichiometry of the reaction. The structure of the HT facilitates this procedure: the row and column of the selected event designate the identities of both the reactants and products as well as the stoichiometric changes in population.

However, other quantities must be updated, most notably the partial sums of reaction rates that facilitate event selection. The first quantity to be updated is the hybridization rate of the probe *m*(22)

where *I *= -1 for hybridization and +1 for dehybridization. Since each partial sum *α*_*j *_is a function of the population of the ℓth cDNA (Eq. 7), these quantities must also be updated:(23)

Subsequently, *a *must be recalculated using Eq. 9. Collectively, 2*N*_*P *_operations are required for the update of the hybridization section of the HT.

After a hybridization or dehybridization event, only one of the dehybridization rates must be updated. Thus, only one partial sum requires modification:(24)

Moreover, inasmuch as Φ_*m *_is the only affected partial sum, Φ may also be updated in one operation,(25)

Thus, only two operations are required to update the dehybridization section of the HT.

In summary, after selection of a reaction, the HT may be updated in *O*(*N*_*P*_) operations, a substantial improvement over the Direct Method. This is largely a result of the fact that reaction rates (*a*_*ν*_) are not stored in the HT, precluding the need to update *N*_*P *_rates of events featuring probe *m *and *N*_*T *_rates featuring target ℓ. The same argument applies to the dehybrization reaction rates {Φ_*j*_}. Ultimately, the method of partial sums is efficient so long as the partial sums can be updated easily.

#### Determination of equilibrium

Microarray analysis is predicated upon the assumption that the probes and solution-phase cDNA have equilibrated prior to scanning the slide and measuring the fluorescence associated with each feature. We follow this experimental convention *in silico*.

The hybridization process is at equilibrium when the rates of change of all chemical populations in Eq. 2 are zero, implying(26)

As straightforward as this criterion appears, it is difficult to employ in practice. Eq. 26 represents millions of comparisons for genome-scale microarrays, requiring *O*(*N*_*P *_*N*_*T*_) operations per time step. This many operations would also be required if one determined the steady states of all molecular species, which would additionally require storage of the current state as well as previous states. Clearly, this is memory-prohibitive. Furthermore, Eq. 26 is exact only on average [30,32]. Hence, it will never be exactly satisfied at any point in time within a single simulation.

To circumvent these issues, we propose an alternate approach that employs the average total rates of hybridization (*α*) and dehybridization (Φ). Considering the definitions of these quantities (Eqs. 7, 8, 9, and 10) summation of Eq. 26 over all ℓ and *m *yields the result that *α *= Φ at equilibrium. This criterion may be established using Student's *t *test for two populations with unknown means and standard deviations [38]. Strictly speaking, this is necessary but insufficient for the specification of thermodynamic equilibrium. However, it is remarkably effective as a heuristic. We implement it as follows:

   **repeat**

      Save *α *and Φ to disk every *O*(*N*_*P*_) reaction steps

      Maintain the last ten saved values of *α *and Φ in memory as vectors ***α ***and **Φ**

      Keep running averages of the numbers in these vectors,  and .

      **if ** <**then**

         **if ***P *< 0.05 for *H*_0_:  =  (*H*_1_:  ≠ ) **then**

            Reinitialize ***α ***and **Φ**

         **end if**

      **end if**

   **until ***P >*0.05 for *H*_0_:  = 

Testing

#### Heuristic for the determination of equilibrium

**Figure 2:**
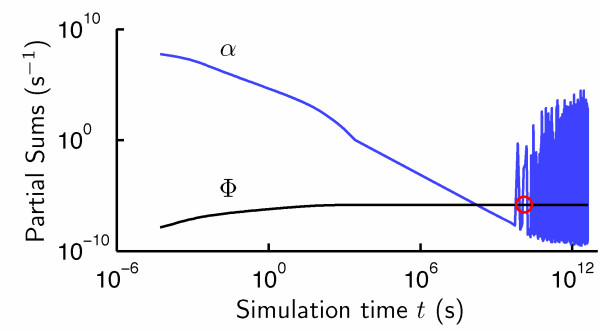
**Determination of equilibrium within simulations of hybridization**. Results are shown for the hybridization of all 6256 Agilent probes with 6718 full-length yeast cDNAs. The red circle indicates the time at which equilibrium is attained.

To evaluate the heuristic approach to the determination of the equilibrium state, we performed simulations of the hybridization of modified Agilent probes with yeast cDNA (Methods). A typical example of the time evolution of *a *and F is presented in Fig. 2, where the time at which equilibrium is attained (as defined by our heuristic) is marked by the red circle. At and after this point, the populations of all hybrid species were at steady state, fluctuating in accordance with the predictions of equilibrium statistical mechanics.

Subsequent comparison of hybrid populations with Eq. 26 confirmed the findings of our "equilibrium heuristic" for all simulations.

In standard hybridizations, imperfect mixing causes the transport of cDNA or cRNA to be diffusion limited [39]. This in turn presents an obstacle to hybridization, as the time required for a (large) target to diffuse to it's probe is large. This, in turn affects the kinetics. The most common solution to this problem is to increase the concentration of target cDNA or cRNA, which results in an abundance of these molecules at equilibrium. As we have discussed, our simulations feature perfect mixing. Thus, we may use substantially less solution-phase cDNA. As a result, free cDNA is sparse at equilibrium, which introduces fluctuation into *a*. By contrast, F exhibits little fluctuation because hybrid populations are fairly large compared to the change in population accompanying a (random) reaction.

#### Effect of reaction rate constants upon the steady state

As discussed, the values of the kinetic rate constants should not affect the equilibrium state of our simulations provided that their ratios are constant (Eq. 35). This assumption may be formulated as a testable hypothesis as follows: If the values of  and  affect the equilibrium state of simulation, then they will affect the fractional occupancy of each probe *m *∈ [1, *N*_*P*_] defined by(27)

In this expression(28)

is the total population of probes *m *hybridized with *any type *of cDNA at equilibrium.

We test these hypotheses at the system level by performing simulations using rate constants(29)

That is, we perturb the rate constants generated via Eqs. 37 and 35 by multiplying their results by a uniform random number *r*_ℓ*m*_. Since a unique random number is generated for each probe *m *and cDNA ℓ, one may quantify the effect of kinetic perturbations on the equilibrium state of a simulation via the hypothesis(30)

If kinetic rate constants significantly affect any of the fractional occupancies at the equilibrium state (*t *→ ∞), then this hypothesis will fail.

**Figure 3:**
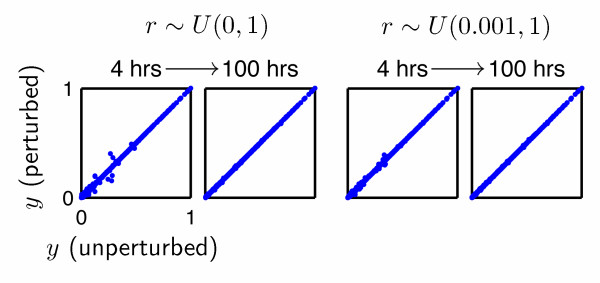
**Effect of kinetic rate constants upon the equilibrium state in microarray simulations**. The fractional occupancies of probes at equilibrium (Eq. 27) are unaffected by random perturbations of rate constants as *t *→ ∞. Each point represents a pair of results for each of the 6256 Agilent probes targeting yeast ORFs. The CPU time required for equilibration of simulated systems (4 h, 100 h, above), like the hybridization time (not shown) does, however, depend upon the rates. Our methodology for estimating rate constants yields rapidly converging simulations.

Simulations of the hybridization of all 6256 modified Agilent probes with 6718 full-length yeast cDNA molecules were conducted for two types of perturbations. In the first study, *r ~U *(0, 1), where *U *(*a, b*) is a uniform random number on the interval (*a, b*). This allowed us to evaluate the effect of the widest variation of the rate constants. In the second study, *r ~U *(0.001, 1). Rate constants for all 42,027,808 hybridization events were independently perturbed. The results of our hypothesis tests are illustrated in Fig. 3.

In both cases, our results clearly indicate that the equilibrium state is unaffected by the values of the rate constants, as expected. Interestingly, the time required to reach equilibrium correlates with the heterogeneity of the dehybridization rate constants: deviations of the results for the two cases at 100 hours of CPU time (not hybridization time) indicate that the hybridization of many probes with solution-phase cDNA was incomplete in cases where their rate constants were perturbed by factors less than 10^-3^.

Analyses of the timeseries of the overall rate of reaction (Fig. 2) revealed that the progression to equilibrium is considerably slower if *r ~U *(0, 1) than if *r ~U *(0.001, 1). This conforms with the experimental observations of Dai and coworkers [40], which demonstrated differences between the kinetics of specific and nonspecific hybridizations.

#### Comparison of stochastic simulation algorithms

Results of all SSAs applied to the process described by Eq. 1 should yield statistically indistinguishable results since they share a common stochastic process. In his seminal work [34], Gillespie showed that the "First Reaction Method" and "Direct Method" were equivalent. Subsequently, Gibson and Bruck demonstrated that their "Next Reaction Method" is equivalent to Gillespie's algorithms. Since the Method of Partial Sums shares the mathematical underpinnings of the Direct Method, its results should be indistinguishable from those generated by the Next Reaction Method or Gillespie's algorithms, as well as the law of mass action.

**Figure 4:**
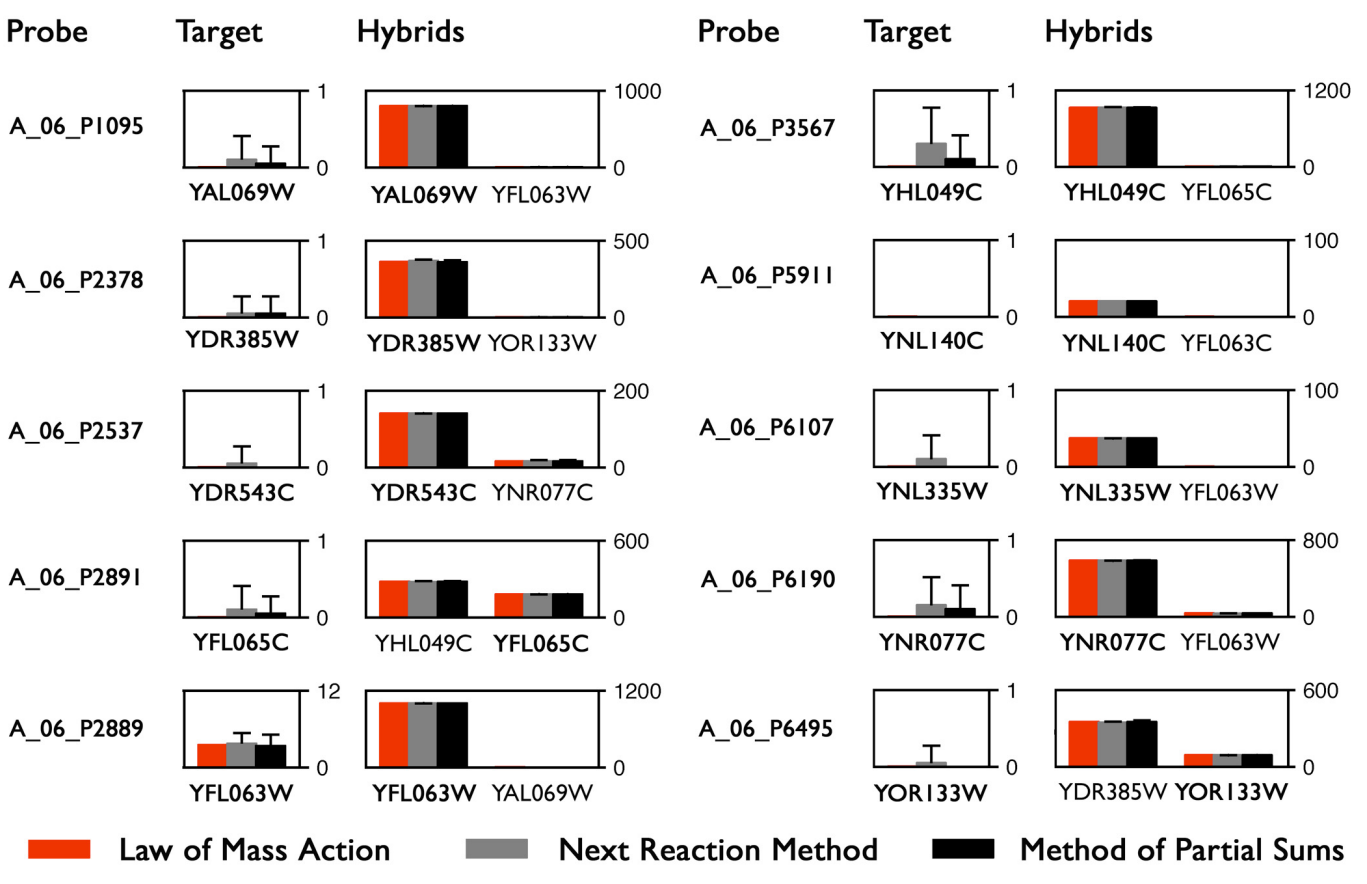
**Comparison of SSAs and the Law of Mass Action**. Results of simulations of the hybridization of ten Agilent probes with their targets (*N*_*P *_= *N*_*T *_= 10) under standard experimental conditions are illustrated (mean ± SD, *n *= 5). All three approaches yield statistically indistinguishable results. In these simulations there are 1000 probe molecules per feature (*N *= 1000), corresponding to a hybridization volume of 0.275 nL.

We initially tested this hypothesis by conducting simulations of the hybridization of ten full length cDNA species from yeast to ten probes for those species. Five simulations were conducted via both the Method of Partial Sums and the Next Reaction Method. Additionally, we solved the corresponding population balance equations (Eqs. 2) for this illustrative hybridization process. Our results (Fig. 4) clearly show that all methods yield equivalent results. Average populations for all 100 hybrid species could not be distinguished by simulation or calculation method via the T-test (*p *> 0.05).

Interestingly, several common differential equation solvers could not integrate Eqs. 2 for this ten by ten system from *t *= 0 until steady state, including the Matlab packages ode23 and ode45. The two hundred equations necessary to model this small array required the use of the ode15s, which is designed for stiff sets of ODEs. By contrast, the stochastic simulation algorithms were unimpeded in their numerical progress. Although the stochastic simulation algorithms give indistinguishable results for the population states both in time and at equilibrium, their computational performances are significantly different. The differences in the computational speeds of the SSAs were evaluated by conducting simulations of the hybridization of full length yeast cDNAs to microarrays featuring 32, 64, 128, 256, and 512 probes targeting those cDNAs (*N*_*P *_= *N*_*T *_for all cases). Additionally, a simulation with 6256 probes and 6718 targets was conducted with the Method of Partial Sums. The same initial populations of targets and the same set of probes (Methods) were used in both the Next Reaction and MPS simulations. Estimation of the time at which equilibrium is attained was determined as discussed, and then used in simulations conducted with the Next Reaction Method.

**Figure 5:**
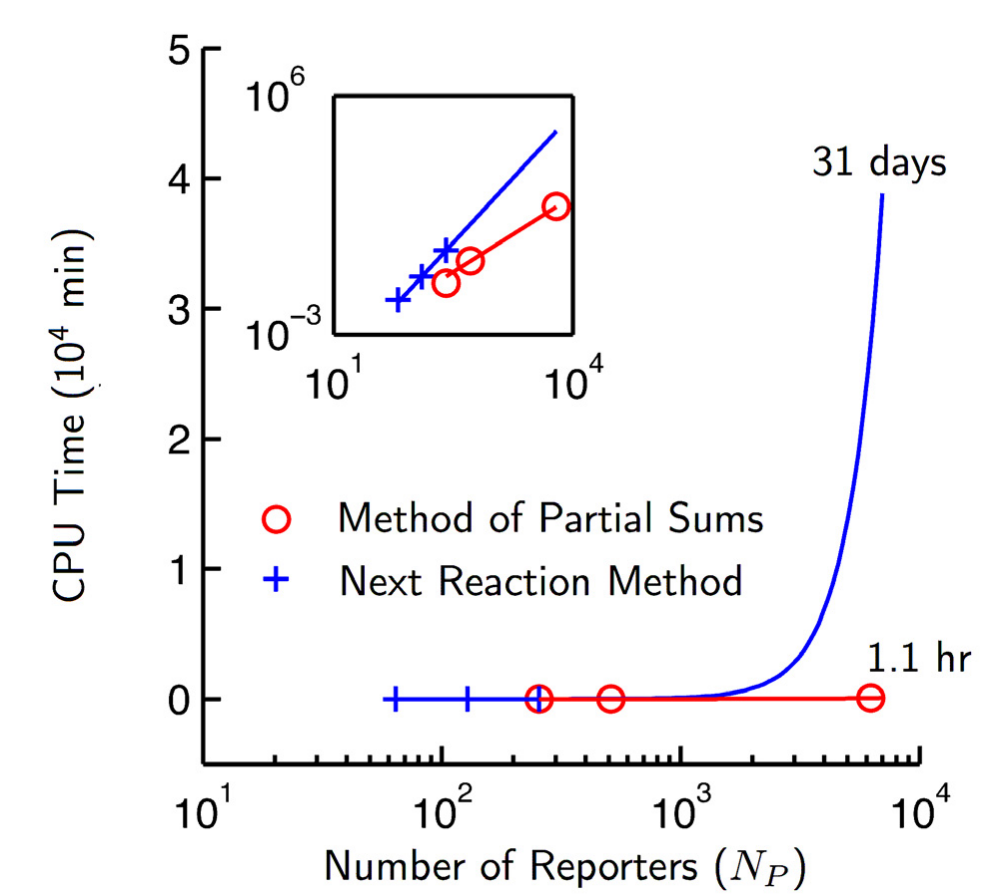
**Computational performance of the Method of Partial Sums and Next Reaction Method**. CPU times for of simulations of hybridization until equilibrium are illustrated. The *in silico *time *t *required to establish equilibrium is determined by our method and utilized as an end point in "Next Reaction" simulations. Our algorithm outperforms the Next Reaction Method in both absolute terms as well as on a per-probe basis (frame).

Our results are illustrated in Fig. 5, and the contrast is stark. The Method of Partial Sums outperforms the Next Reaction Method for simulations of microarrays of all sizes. It requires one hour to complete a simulation for an array large enough to be used for genomic characterization. By contrast, the Next Reaction Method is not capable of performing such simulations due to its memory requirements. If *N*_*P *_*~N*_*T *_*~ *6000, the pointers required by the Next Reaction Method will consume approximately three terabytes of 64 bit computer memory by themselves; the HT requires no such pointers. Next Reaction simulations with as few as 512 probes required 12 hrs of CPU time to reach equilibrium, and for a computer with extraordinary memory, we forecast that simulations using the Next Reaction Method would require a month to simulate the hybridization to the Agilent yeast array.

In addition, the scaling of the CPU time with respect to the number of probes differs among the two stochastic simulation algorithms. Both algorithms feature power-law scaling, however, the Method of Partial Sums scales as , whereas the Next Reaction Method scales as  (mean ± SE). These differences arise due to the differences in the data structures underlying the two methods, which in turn affect the number of calculations required per time step.

Our algorithm permits two concurrent simulations of an 84 million-reaction system (e.g. the Agilent yeast array with cDNA from the yeast transcriptome) to reach equilibrium within two hours using 2.1 GHz Dual G5 Apple servers with 2 GB memory. 4 GB of memory are sufficient for simulations featuring 12,000 probes, e.g. simulations of yeast arrays with one perfect match probe and one mismatch probe for each transcribed gene. Simulations of an array designed for the human genome with one probe per gene - at approximately 25,000 genes - possess hybridization reaction spaces approximately 16 times larger, and an estimated run time of 30 - 60 hours based on CPU time scaling of *t *~. Such simulations can be achieved on most University shared-resource machines (i.e. the SGI Altix at Lehigh University), which commonly feature hundreds of gigabytes of RAM.

#### Illustrative Example: Characterization of Cross Hybridization

The *in silico *characterization of cross hybridization is only as sensitive as the number of probe molecules per feature. Agilent arrays have 2.0 × 10^8 ^probe molecules per feature, facilitating the hybridization and measurement of as many target cDNAs and giving, in principle, as much resolution. However, the time required for stochastic simulations is proportional to the number of molecules therein [34,36,41]. Balancing these resolution and population considerations, we selected a hybridization volume of 0.275 nL corresponding to initial probe populations of 1000 molecules/feature and concomitant scaling of the feature diameter to maintain the surface concentration. We also employed a total concentration of 100 ng per 60 *μ*L hybridization volume. At this concentration, only a few if any probes become saturated. This concentration is a tenfold dilution of that recommended by Agilent's protocol, however, it is is within the range of commercial oligonucleotide microarray protocols [42]. Moreover, the Agilent array effectively has a probe population four times higher than the one we used, as it has four redundant features per probe (the 4 × 44 design, Methods).

**Figure 6:**
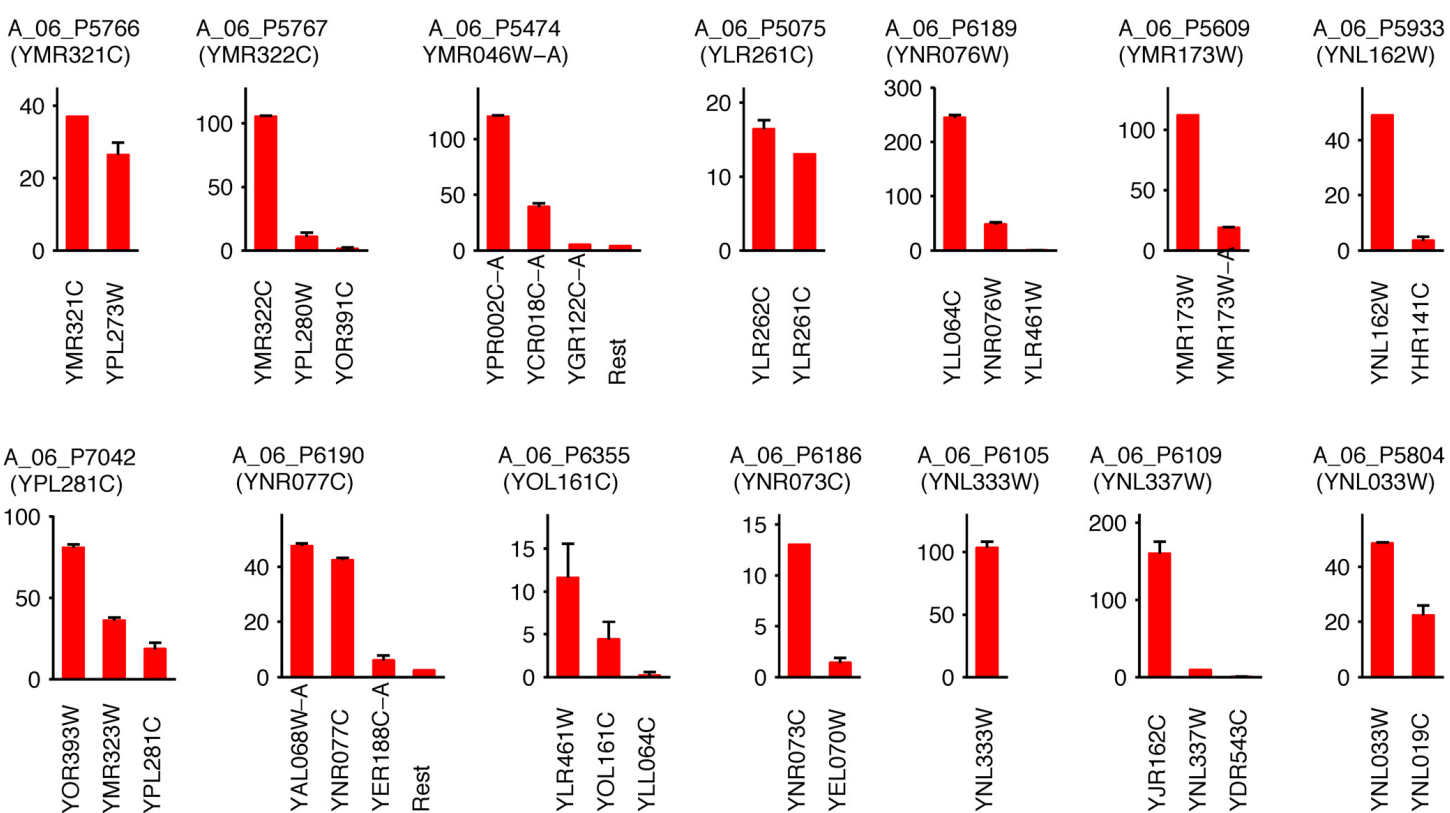
**Hybridization of cDNA with Agilent 4 × 44 array probes**. The populations of hybrids with these fourteen probes (mean ± SD) are a subset of the complete set of results, which are generated from five replicate simulations.

In Fig. 6 we illustrate a small subset of the hybrid populations predicted from our simulations. The average populations of hybrids were calculated from five replicate simulations, each of which required 1.1 hours of CPU time as discussed (Fig. 5). The complete sets of results are provided in Additional Files 1 and 2.

**Figure 7:**
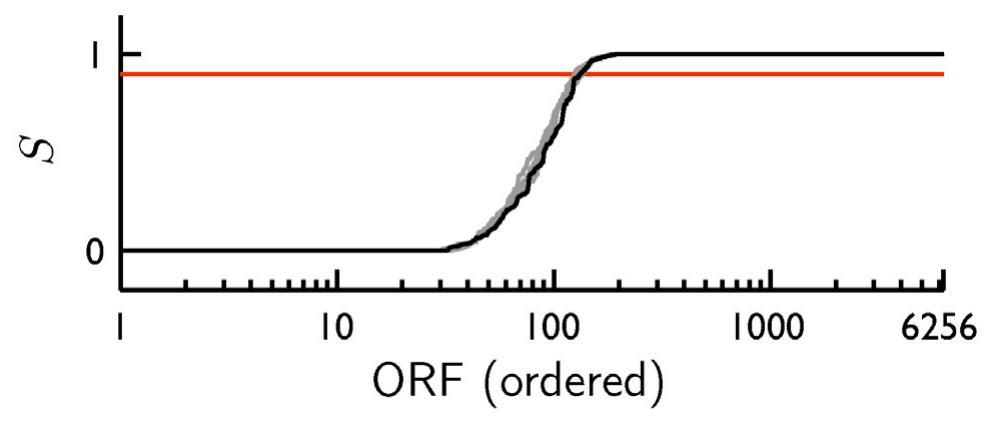
**Selectivity distribution for the Agilent probe set**. The black line illustrated the selectivities (Eq. eq:Selectivity) of all Agilent probes when the array is hybridized to yeast cDNAs at the initial concentrations described in Methods. The gray lines are results for four different initial initial conditions. The red line delimits 90% selectivity. The *distribution *is independent of the initial concentrations (*x*-axes are different for all five sets of results). About 100 probes will not be selective in any given microarray experiment.

Many of the cross-hybridizing cDNA species are exceptionally homologous. For instance, A_06_P7042 - the probe designed to target cDNA for YPL281C (*ERR2*) also hybridizes with cDNA for YOR393W (*ERR1*); in this case, the two ORFs are identical. The third hybrid, YMR323W (*ERR3*), shares all but twelve bases of the other two. Other cross hybridizing cDNA species have less overlap in sequence, but share the probe sequences completely or in part. All cross-hybrids listed in Fig. 6 share at least 90% of the target sequence. A probe's proclivity to cross hybridize in the presence of thousands of potentially competitive cDNAs may be expressed in terms of its selectivity, *S*, defined as the fraction of fluorescence intensity associated with a microarray feature that originates from the corresponding target. Mathematically,(31)

where  is the population of hybrids composed of probe *m *and its target ((*m*)), and  is calculated via Eq. 28. For example, the selectivity of the aforementioned Agilent probe A_06_P7042 is 13.8%. In Fig. 7, we present a summary of the selectivities for all probes in the Agilent set. As these results show, the vast majority of the probes for this commercial microarray are selective (e.g A_06_P6109 in Fig. 6), and do not exhibit cross hybridization when they bind yeast cDNA at the concentrations specified by the expression state. For other microarrays and probe sets designed by various publicly available software packages, the cross hybridization may be more extreme.

Since the populations of all hybrids depend upon the populations of potentially cross-hybridizing cDNA molecules, the value of *S *for each probe depends upon the expression state of a cell. Interestingly, the *distribution *of *S *among the probes is independent of the expression state. Simulations with different initial conditions corresponding to four additional MC-generated expression states yielded selectivity distributions that were indistinguishable from the aforementioned distribution (Fig. 7). This result suggests a method of characterizing the overall reliability of a proposed probe set. Since the distribution of selectivities is invariant with respect to the initial target populations (provided the total cDNA concentration does not force saturation of probes), a statistic that characterizes that distribution should be robust. We propose that the average selectivity can serve as such a metric. The use of such metrics should be used with care, however, inasmuch as they lend themselves to "ecological fallacy".

### Implementation

Our software is implemented in C++ and executed on Apple G5 processors running MacOS X (Tiger). UNAFOLD 3.5 is available for download at the DINAMelt server http://dinamelt.bioinfo.rpi.edu/ and compiles on a variety of operating systems.

Additionally, we have implemented the Next Reaction Method as described by Gibson and Bruck [37] in C++ for the purposes of comparing its performance with that of our algorithm.

## Discussion

In recent years, advances in microarray manufacturing have opened the doors to custom microarray design. Individual researchers can now upload their own microarray probe sequences to one of many sites (e.g. Agilent's e-array website) and have a custom array manufactured. In response, many probe design algorithms have been arisen to fill the needs of researchers intent on performing global investigations of gene expression [25,26,43-47]. However, none of the probe sets generated by these algorithms have been evaluated in terms of their selectivity or proclivity to give a linear relationship between feature fluorescence and target concentration. Studies of this type carried out by the MicroArray Quality Control Consortium [22] were costly, involving dozens of participants. Such laborious quality control is not feasible for each and every probe set designed by a novel probe tool. However, robust population-based simulations of the hybridization process may be employed to evaluate candidate probe sets, given robust estimates of the thermodynamic free energies of hybridization.

In the tests of our simulation algorithm, we have utilized equilibrium constants that were calculated via the NN model of Allawi and SantaLucia. This model predicts free-energies to within three significant digits of experimentally measured values for solution-phase hybridization [48] and has been validated for oligonucleotide arrays in which the oligonucleotides are connected to the surface by linkers, making them more "solution like" [49-52]. However, many studies have demonstrated that NN models do not accurately predict the thermodynamic properties of hybridization between solution-phase and surface-bound oligonucleotides (e.g. [53,54]).

Electrostatic interference in conventional microarrays - which do not feature 3D linkers of the type employed by Weckx and coworkers [52], is a major reason for this discrepancy. Solution-phase Na^+ ^may shield the phosphate groups of both hybridized and single-stranded probes and targets [55]. Cations will also shield the negatively-charged glass surface to which the probes are bound by organizing the formation of a layer of counter ions. The surface density the oligonucleotide probes also plays a role, partly via steric interference and partly via changes in the charge distribution at the glass-water interface [50,55-58]. Ultimately, surface electrostatic effects cause a location-dependent effect of mismatches [54,59].

Therefore, extra care must be taken when applying our algorithm to characterize cross hybridization in real microarray assays. If equilibrium constants are calculated using NN models (e.g. UNAFOLD [60,61], Pairfold [62,63] or BINDIGO [44]), corrections for the effect of the Debye layer should be introduced at a bare minimum. Theoretical predictions of the effect of the resulting "Debye layer" upon the melting temperature *T*_*m *_[64,65] have been confirmed via experimental measurements [66]. This correction may be applied to NN models for DNA-cRNA duplexes as well (e.g. the semi-empirical model of Wu and coworkers [67]), if simulations of cRNA-DNA assays are to be conducted.

We have not explicitly considered additional effects of single-strand secondary structure (SS) formation among full-length cDNA or probes(32)

Nor, for that matter, have we considered the possibility of hybridization between cDNA molecules in solution(34)

The hybridization between probes, or folding thereof, is customarily not a substantial problem in oligonucleotide microarrays. Probe design software packages such as OligoArray 2.0 routinely screen probes for secondary structure, and inter-probe hybrids are precluded due to spatial separation. The remaining interactions among cDNAs (folding and inter-hybridization) serve only to sequester single-stranded cDNA from the probes [68]. Inasmuch as this can only further degrade the sensitivity of probe-target interactions, we have restricted our algorithm to consider only probe-cDNA hybridization dynamics. Hence, the method we have presented for probe and array characterization (Figs. 6 and 7) gives the performance of a microarray under a "best case" scenario, even as it fully accounts for the interactions between oligonucleotide probes and full length cDNA targets at the system-scale.

For the end user we note that simulation-based characterization of putative microarray probe sets requires no more information than that contained in MAGE-TAB (Microarray Gene Expression Tabular) or MAGE-ML (Microarray Gene Expression Mark-up Language) formatted array information [69,70], which are required for publication of microarray data to ensure MIAME compliance. The Array Design Format (ADF) component of MAGE-TAB contains all of the probe sequence information and its target(s), whereas the Investigation Description Format (IDF) contains the experimental protocols, including the hybridization temperature and salt conditions. Additionally, the ADF contains information regarding the relationship between reporter sequences and features: there may be multiple features with the same reporter sequence, or alternately, signals from several reporters may be combined to produce a signal for a single gene (e.g. Affymetrix Gene Chips). As the total cDNA concentration is ostensibly included in the IDF, and the individual cDNA concentrations are randomly generated, the system-scale selectivity of any array can be computed via simulation from a proposed experimental protocol.

In this work, we have not explicitly considered the prediction of the time evolution of the hybridization process, focusing instead on the equilibrium state. Simulations employing our algorithm are most accurate when they utilize experimentally-determined rate constants [54,57,71]. However, care must be taken to ensure simulations are conducted for the same surface densities and ionic strengths employed in the experiments employed to estimate the rate constants, for the reasons previously discussed. Given accurate estimates of the rate constants  and , the time required for microarray hybridizations may be estimated via the method illustrated in Fig. 3, as the time variable would represent the actual hybridization time.

## Conclusions

In this work, we have developed an algorithm for the stochastic simulation of exceptionally large and complex probe-cDNA hybridization reaction networks that underlie microarray assays.

Using the method of partial sums in conjunction with the data structure we denote the "hybridization table", our algorithm requires *O*(*N*_*P*_) operations per reaction event. This is substantially fewer than Gillespie's Direct Method (*O*() operations per event) and the Next Reaction Method of Gibson and Bruck (*O*(*N*_*P *_log(*N*_*P*_)) operations per event). Moreover, our algorithm requires less the data storage than others, obviating the need for pointers that track of the dependencies of reactions. For instance, the Next Reaction Method requires (*N*_*P *_+ *N*_*T *_- 1) pointers for each of its 2*N*_*P *_*N*_*T *_reactions, which can consume a vast amount of memory for genome-scale simulations.

As a result of these innovations, our algorithm permits system-level simulation of the complete reaction network composed of all potential probe-target hybridizations (for any genome or array) without the need for high-performance computing. Furthermore, such simulations are now possible within a reasonable amount of time. Thus, given robust thermodynamic predictions of the free energies of DNA hybridization, one may obtain a conservative estimate of the reliability of a candidate probe set *in silico*.

## Methods

### Microarray specifications and simulation protocols

We investigated the hybridization of cDNA originating from *S. cerevisiae *to a reproduction of the Agilent Yeast Oligo Microarray Kit (V2, see Additional File 3). 6,256 of the probes on this array target yeast ORFs, each targeting one gene, and the remainder consist of randomly located oligonucleotide probes, control features, and empty spots. The Agilent array features multiple identical reporters, each of which is randomly distributed over the array surface to abrogate spatial artifacts. Insofar as our simulations treat the hybridization volume as homogeneous, we have not included redundant probes in our simulations. We have also removed control features that do not target yeast ORFs.

Agilent arrays of the type considered here possess of 65 *μ*m wide features with surface probe densities of is 6.0 × 10^12 ^probe molecules per cm^2 ^[72]. Therefore each (redundant) feature consists of 2.0 × 10^8 ^probe molecules. The hybridization protocol for Agilent microarrays suggests addition of 60 *μ*L of cDNA mixture to the microarray. Thus, a "total probe concentration" of 3.3 × 10^6 ^probe molecules/* μ*L was used for each feature in all simulations.

The Agilent protocol also suggests that the hybridization mixture contains 1.65 *μ*g of linearly amplified and labeled cRNA and possesses a NaCl concentration of 750 mM - specifications that are typical among experimental protocols [42,73,74]. Agilent also recommends hybridization at 65°C.

We have conformed to the Agilent protocol with three exceptions. First, full-length cDNA were used in lieu of cRNA in light of the observations of Eklund and coworkers, who demonstrated that replacement of cRNA targets with cDNA reduces microarray cross hybridization [13]. Second, although 1-100 *μ*g of non-amplified RNA is typically required in experimental protocols for microarrays [42,74], Nagino and coworkers have shown that it may be reduced to 10 - 100 ng by improving mixing [42]. This would also reduce steric effects and electrostatic effects induced by dense packing of charged oligonucleotides on the glass surface (cf [55,56]). Finally, we assumed that the probes are separated from the glass surface by linker molecules of lengths greater than the Debye length. In so doing, we may utilize the NN model of Allawi and Santalucia [75] to calculate the free energies of hybridization between probes and targets. We then investigated the effect of total DNA concentration upon cross hybridization and signal response by hybridizing 50, 100 and 200 ng of cDNA per 60 *μ*L aliquot (1.8, 3.6 and 7.2 nM).

### cDNA populations

Oligonucleotide probes were hybridized to cDNAs for each of the *N*_*T *_= 6718 protein-encoding ORFs in the November 10, 2006 version of the yeast genome. Differential expression studies akin to those employed by the MAQC [22] were performed using a "gold standard" expression state defined by the amounts of each cDNA, , ℓ = 1 ... 6718, which, by convention, we assume to be proportional to the amounts of the corresponding mRNA. The amounts of each transcript were selected via Monte Carlo from a log-normal distribution that was fit to the yeast expression dataset of Cho and coworkers (Additional File 4) [76]. The fits of all seventeen expression datasets (two cell cycles) revealed that the genomic expression levels of yeast are log-normally distributed with a coefficient of variation of 0.21, independent of the expression state; the mean of the distribution corresponds to the total solution-phase DNA concentration.

### Kinetics and equilibrium constants

The methodology thus presented for the simulation of the time evolution of the hybridization of cDNA to oligonucleotide microarrays is valid if and only if the rate constants employed *in silico *are valid. At equilibrium, which corresponds to the condition where microarray slides are scanned, the constraints on the population balance equations (Eq. 2) and stochastic simulation are less stringent. In this case, the populations of all ssDNA and dsDNA species predicted by stochastic simulation will be accurate if and only if the equilibrium constants *K*_ℓ*m *_are accurate, where(35)

This is a direct consequence of the thermodynamic principle of microscopic reversibility [77-79]. Therefore, our simulation procedure will accurately predict the extent of cross hybridization if and only if the equilibrium constants employed *in silico *are valid.

To this end, we have employed UNAFOLD [60,61] - a standard bioinformatics tool. Although UNAFOLD is largely used for the prediction of secondary structures of DNA and RNA (excluding pseudoknots), it is also capable of calculating free energies of hybridization (Δ*G*_ℓ*m*_) via the semi-empirical hybridization model of Allawi and Santalucia [75]. This "Nearest Neighbor" (NN) model is exceptionally accurate for two reasons. First, its mathematical form accounts for nearest neighbor interactions within hybrids (e.g. thermodynamic contributions due to basepair stacking). Second, its parameters are robustly calculated from an abundance of well-controlled experimental measurements on mismatched oligomers. Given the sequences of a pair of DNA molecules, the NN model will reproduce experimentally-measured Δ*G*_ℓ*m *_for (a) solution-phase hybrids and (b) oligomers that are separated from the slide surface by distances greater than the Debye length (cf. [52]).

Using the NN model via UNAFOLD, we calculated Δ*G*_ℓ*m *_between all probes and cDNAs between all probes and cDNAs under experimental temperatures and salt concentrations. Equilibrium constants were then evaluated using the standard formula(36)

where *K*_ℓ*m *_has units of M^-1^. Equilibrium constants are functions of temperature and salt concentrations alone, and are independent of cDNA concentration.

Although the equilibrium state of hybridization is fully determined by the equilibrium constants *K*_ℓ*m*_, SSAs require reaction rate constants. As we have discussed, microscopic reversibility precludes the possibility that the values of the rate constants will affect the populations of ssDNA and dsDNA species at equilibrium. Therefore, one of the rate constants may be estimated provided that the other is calculated using 35 and the NN-model prediction of the equilibrium constant.

In our simulations, we have chosen to estimate the "off rates", . We do so using Delisi's equation [80], where(37)

is the diffusion-limited off-rate. In this expression, *D*_ℓ _is the diffusion constant of cDNA ℓ, and *s*_ℓ*m *_is the sum of radii of gyration of the oligonucleotide probe *m *and cDNA ℓ, *K*_0 _accounts for the energetics associated with the transition state, and λ* and V* are associated with surface potentials. As the off-rate is an estimate, we treat the second term of Eq. 37 as a constant and assign(38)

where *N*_Av _is the Avogadro's number [81]. In so doing, we preclude all "on-rates" {} from exceeding the Smoluchowki rate of diffusion-limited association *k*_+ _= 2*πD*_ℓ_*s*_ℓ*m*_*N*_Av_

The on-rates are calculated from Eq. 35, with the estimates of the off-rate and the free-energies calculated with SantaLucia's NN model. As the kinetic rate constant estimation procedure preserves the equilibrium constants, the simulation predictions of the dsDNA and ssDNA populations will be accurate at equilibrium. The effect of kinetic parameters upon the time required to reach the equilibrium states of hybridization is discussed in the Results section.

## Authors' contributions

IL and EA developed the algorithm. EA implemented the algorithm and carried out the simulations and analyses. IL and EA wrote the manuscript.

## Supplementary Material

Additional file 1 **Arslan_Laurenzi_Supplemental**. This file contains a list of Agilent reporter names and the yeast ORFs targeted by each. The sequences of the Agilent probes may be obtained from https://earray.chem.agilent.com/earray/. Click here for file

Additional file 2 **Arslan_Laurenzi_Supplemental**. This file lists the populations of full length cDNA molecules used in all simulations conducted with *N *= 1000 probe molecules per feature. The concentrations of each cDNA species may be calculated (in number of molecules/nL) by dividing these populations by the hybridization volume corresponding to this probe population (0.275 nL). The sequences of these cDNA molecules may be obtained from the Saccharomyces Genome Database http://www.yeastgenome.org; we have utilized the November 10, 2006 version of the yeast genome. Click here for file

Additional file 3 **Arslan_Laurenzi_Supplemental**. This file contains the populations of all hybrids (*X*) at equilibrium for the experiments described in section "Characterization of Cross Hybridization". Simulations of the hybridization of full length yeast cDNA to the Agilent probe set were conducted at 65°C. Initial cDNA populations (prior to hybridization) are specified in the worksheet "Initial Target Populations". There are 1000 copies of each of the 6256 Agilent probes per feature. The simulation is conducted at 0.275 nL. The results of five replicate simulations are provided - differences between the results of each run are due to the probabilistic nature of chemical reaction. Data are organized by row and column: for instance, there are thirteen hybrids between A_06_P1002 and Q0010 at the end of the first replicate simulation (Run number 1) Click here for file

Additional file 4 **Arslan_Laurenzi_Supplemental**. The average (and standard deviation) of the populations of each hybrid are provided in this worksheet, as calculated from the results provided in Supplemental Table 3. Click here for file

## References

[B1] Margulies M Egholm M Altman WE Attiya S Bader JS Bemben LA Berka J Braverman MS Chen YJ Chen Z Dewell SB Du L Fierro JM Gomes XV Godwin BC He W Helgesen S Ho CH Irzyk GP Jando SC Alenquer MLI Jarvie TP Jirage KB Kim JB Knight JR Lanza JR Leamon JH Lefkowitz SM Lei M Li J Lohman KL Lu H Makhijani VB McDade KE McKenna MP Myers EW Nickerson E Nobile JR Plant R Puc BP Ronan MT Roth GT Sarkis GJ Simons JF Simpson JW Srinivasan M Tartaro KR Tomasz A Vogt KA Volkmer GA Wang SH Wang Y Weiner MP Yu P Begley RF Rothberg JM Genome sequencing in microfabricated high-density picolitre reactors Nature 2005 437 376 380 16056220 10.1038/nature03959 PMC1464427

[B2] Cloonan N Forrest ARR Kolle G Gardiner BBA Faulkner GJ Brown MK Taylor DF Steptoe AL Wani S Bethel G Robertson AJ Perkins AC Bruce SJ Lee CC Ranade SS Peckham HE Manning JM McKernan KJ Grimmond SM Stem cell transcriptome profiling via massive-scale mRNA sequencing Nature Methods 2008 5 613 619 10.1038/nmeth.1223 18516046

[B3] Chiang DY Getz G Jaffe DB O'Kelly MJT Zhao X Carter SL Russ C Nusbaum C Meyerson M Lander ES High-resolution mapping of copy-number alterations with massively parallel sequencing Nature Methods 2009 6 99 103 10.1038/nmeth.1276 19043412 PMC2630795

[B4] Fodor SP Rava RP Huang XC Pease AC Holmes CP Adams CL Multiplexed biochemical assays with biological chips Nature 1993 364 555 556 10.1038/364555a0 7687751

[B5] Pease AC Solas D Sullivan EJ Cronin MT Holmes CP Fodor SP Light-generated oligonucleotide arrays for rapid DNA sequence analysis Proc Natl Acad Sci USA 1994 91 5022 5026 10.1073/pnas.91.11.5022 8197176 PMC43922

[B6] Lockhart DJ Dong H Byrne MC Follettie MT Gallo MV Chee MS Mittmann M Wang C Kobayashi M Horton H Brown EL Expression monitoring by hybridization to high density oligonucleotide arrays Nature Biotechnol 1996 14 1675 1680 10.1038/nbt1296-1675 9634850

[B7] Fodor SP Read JL Pirrung MC Stryer L Lu AT Solas D Light-directed, spatially addressable parallel chemical synthesis Science 1991 251 767 773 10.1126/science.1990438 1990438

[B8] Lipshutz RJ Morris D Chee M Hubbell E Kozal MJ Shah N Shen N Yang R Fodor SP Using oligonucleotide probe arrays to access genetic diversity Biotechniques 1995 19 442 447 7495558

[B9] Chee M Yang R Hubbell E Berno A Huang XC Stern D Winkler J Lockhart DJ Morris MS Fodor SPA Accessing genetic information with high-density DNA arrays Science 1996 274 610 614 10.1126/science.274.5287.610 8849452

[B10] Nuwaysir EF Huang W Albert TJ Singh J Nuwaysir K Pitas A Richmond T Gorski T Berg JP Ballin J McCormick M Norton J Pollock T Sumwalt T Butcher L Porter D Molla M Hall C Blattner F Sussman MR Wallace RL Cerrina F Green RD Gene expression analysis using oligonucleotide arrays produced by maskless photolithography Genome Res 2002 12 1749 1755 10.1101/gr.362402 12421762 PMC187555

[B11] Albert TJ Norton J Ott M Richmond T Nuwaysir K Nuwaysir EF Stengele KP Green RD Light-directed 5'-3' synthesis of complex oligonucleotide microarrays Nucleic Acids Res 2003 31 7 e35 10.1093/nar/gng035 12655023 PMC152820

[B12] Schena M Shalon D Davis RW Brown PO Quantitative monitoring of gene-expression patterns with a complementary-DNA microarray Science 1995 270 467 470 10.1126/science.270.5235.467 7569999

[B13] Eklund AC Turner LR Chen P Jensen RV Defeo G Kopf-Sill AR Szallasi Z Replacing cRNA targets with cDNA reduces microarray cross-hybridization Nat Biotechnol 2006 24 1071 1073 10.1038/nbt0906-1071 16964210

[B14] Quackenbush J Microarray data normalization and transformation Nat Genet 2002 32 496 501 10.1038/ng1032 12454644

[B15] Cui X Churchill GA Statistical tests for differential expression in cDNA microarray experiments Genome Biol 2003 4 210 10.1186/gb-2003-4-4-210 12702200 PMC154570

[B16] Yang IV Chen E Hasseman JP Liang W Frank BC Wang S Sharov V Saeed AI White J Li J Within the fold: assessing differential expression measures and reproducibility in microarray assays Genome Biol 2002 3 research0062 12429061 10.1186/gb-2002-3-11-research0062 PMC133446

[B17] Tan PK Downey TJ Jr ELS Xu P Fu D Dimitrov DS Lempicki RA Raaka BM Cam MC Evaluation of gene expression measurements from commercial microarray platforms Nucleic Acids Res 2003 31 19 5676 5684 10.1093/nar/gkg763 14500831 PMC206463

[B18] Etienne W Meyer MH Peppers J Meyer RA Comparison of mRNA gene expression by RT-PCR and DNA microarray BioTechniques 2004 36 4 618 626 15088380 10.2144/04364ST02

[B19] Shippy R Sendera T Lockner R Palaniappan C Kaysser-Kranich T Watts G Alsobrook J Performance evaluation of commercial short-oligonucleotide microarrays and the impact of noise in making cross-platform correlations BMC Genomics 2004 5 61 http://www.biomedcentral.com/1471-2164/5/61 10.1186/1471-2164-5-61 15345031 PMC517929

[B20] Larkin JE Frank BC Gavras H Sultana R Quackenbush J Independence and reproducibility across microarray platforms Nature Methods 2006 2 337 344 10.1038/nmeth757 15846360

[B21] Canales RD Luo Y Willey JC Austermiller B Barbacioru CC Boysen C Hunkapiller K Jensen RV Knight CR Lee KY Ma Y Maqsodi B Papallo A Peters EH Poulter K Ruppel PL Samaha RR Shi L Yang W Zhang L Goodsaid FM Evaluation of DNA microarray results with quantitative gene expression platforms Nat Biotech 2006 24 1115 1122 10.1038/nbt1236 16964225

[B22] MAQC Consortium The MicroArray Quality Control (MAQC) project shows inter- and intraplarform reproducibility of gene expression measurements Nat Biotech 2006 24 9 1151 1160 10.1038/nbt1239 PMC3272078 16964229

[B23] Bhanot G Louzoun Y Zhu J DeLisi C The Importance of Thermodynamic Equilibrium for High Throughput Gene Expression Arrays Biophys J 2003 84 124 135 10.1016/S0006-3495(03)74837-1 12524270 PMC1302598

[B24] Zhang Y Hammer DA Graves DJ Competitive Hybridization Kinetics Reveals Unexpected Behavior Patterns Biophys J 2005 89 2950 2959 10.1529/biophysj.104.058552 16126833 PMC1366793

[B25] Roulliard JM Zuker M Gulari E OligoArray 2.0: design of oligonuleotide probes for DNA microarrays using a thermodynamic approach Nucleic Acids Res 2003 31 3057 3062 10.1093/nar/gkg426 12799432 PMC162330

[B26] Li F Stormo GD Selection of optimal DNA oligos for gene expresion arrays Bioinformatics 2001 17 11 1067 1076 10.1093/bioinformatics/17.11.1067 11724738

[B27] Chou CC Chen CH Lee TT Peck K Optimization of probe length and the number of probes per gene for optimal microarray analysis of gene expression Nuc Acids Res 2004 32 12 e99 10.1093/nar/gnh099 PMC484198 15243142

[B28] Cherry JM Ball C Weng S Juvik G Schmidt R Adler C Dunn B Dwight S Riles L Mortimer RK Botstein D Genetic and physical maps of Saccharomyces cerevisiae Nature 1997 387 67 73 10.1038/43025 9169866 PMC3057085

[B29] Versteeg R van Schaik BD van Batenburg MF Roos M Monajemi R Caron H Bussemaker HJ van Kampen AH The Human Transcriptome Map Reveals Extremes in Gene Density, Intron Length, GC Content, and Repeat Pattern for Domains of Highly and Weakly Expressed Genes Genome Res 2003 13 1998 2004 10.1101/gr.1649303 12915492 PMC403669

[B30] Renyi A A discussion of chemical reactions using the theory of stochastic processes MTA Alk Mat Int Kozl 1953 2 83 101

[B31] Gillespie DT A rigorous derivation of the stochastic master equation Physica A 1992 188 402 425 10.1016/0378-4371(92)90283-V

[B32] Darvey IG Ninham BW Staff PJ Stochastic models of second-order chemical reaction kinetics. The equilibrium state J Chem Phys 1966 45 6 2145 2155 10.1063/1.1727900

[B33] McQuarrie DA Stochastic approach to chemical kinetics J Appl Prob 1967 4 413 467 10.2307/3212214

[B34] Gillespie DT A general method for numerically simulating the stochastic time evolution of coupled chemical reactions J Comput Phys 1976 22 403 434 10.1016/0021-9991(76)90041-3

[B35] Laurenzi IJ An analytical solution of the stochastic master equation for reversible bimolecular reaction kinetics J Chem Phys 2000 113 8 3315 3322 10.1063/1.1287273

[B36] Gillespie DT Exact stochastic simulation of coupled chemical reactions J Phys Chem 1977 81 25 2340 2361 10.1021/j100540a008

[B37] Gibson MA Bruck J Efficient exact stochastic simulation of chemical systems with many species and many channels J Phys Chem A 2000 104 1876 1889 10.1021/jp993732q

[B38] Montgomery DC Runger GC Applied Statistics and Probability for Engineers 1999 2 John Wiley

[B39] Gadgil C Yeckel A Derby JJ Hu WS A diffusion-reaction model for DNA microarray assays J Biotechnol 2004 114 31 45 10.1016/j.jbiotec.2004.05.008 15464596

[B40] Dai H Meyer M Stepaniants S Ziman M Stoughton R Use of hybridization kinetics for differentiating specific form non-specific binding to oligonucleotide microarrays Nucleic Acids Res 2002 30 16 e86 10.1093/nar/gnf085 12177314 PMC134259

[B41] Laurenzi IJ Bartels JD Diamond SL A General algorithm for exact simulation of multicomponent aggregation processes J Comput Phys 2002 177 418 449 10.1006/jcph.2002.7017

[B42] Nagino K Nomura O Takii Y Myomoto A Ichikawa M Nakamura F Higasa M Akiyama H Nobumasa H Shiojima S Tsujimoto G Ultrasensitive DNA Chip: Gene Expression Profile Analysis without RNA amplification J Biochem 2006 139 4 697 703 10.1093/jb/mvj086 16672270

[B43] Gordon PMK Sensen CW Osprey: a comprehensive tool employing novel methods for the design of oligonucleotides for DNA sequencing and microarrays Nucleic Acids Res 2004 32 17 e133 10.1093/nar/gnh127 15456895 PMC521677

[B44] Hodas NO Aalberts DP Efficient computation of optimal oligo-RNA binding Nuclic Acids Res 2004 32 6636 6642 10.1093/nar/gkh1008 PMC545450 15608295

[B45] Roulliard JM Herbert CJ Zuker M OligoArray: Genome-scale oligonucleotide design for microarrays Bioinformatics 2002 18 486 487 10.1093/bioinformatics/18.3.486 11934750

[B46] Sharma A Srivastava GP Sharma VK Ramachandran S ArrayD: A general purpose software for Microarray design BMC Bioinformatics 2004 5 142 147 10.1186/1471-2105-5-142 15461789 PMC524372

[B47] Wernersson R Nielsen HB OligoWiz 2.0 - integrating sequence feature annotation into design of microarray probes Nucleic Acids Res 2005 33 W611 W615 10.1093/nar/gki399 15980547 PMC1160160

[B48] SantaLucia J Hicks D The Thermodynamics of DNA Structural Motifs Annual Rev Biophys Biomol Struct 2004 33 415 440 10.1146/annurev.biophys.32.110601.141800 15139820

[B49] Dorris DR Nguyen A Gieser L Lockner R Lublinksy A Patterson M Touma E Sendera TJ Elghanian R Mazumder A Oligodeoxyribonucleotide probe accessibility on a three-dimensional DNA microarray surface and the effect of hybridization time on the accuracy of expression ratios BMC Biotechnology 2003 3 6 16 10.1186/1472-6750-3-6 12801425 PMC165584

[B50] Hong BJ Sunkara V Park JW DNA microarrays on nanoscale-controlled surface Nucleic Acids Res 2005 33 e106 10.1093/nar/gni109 16002785 PMC1174902

[B51] Halperin A Buhot A Hybridization at a Surface: The Role of Spacers in DNA Microarrays Langmuir 2006 22 11290 11304 10.1021/la0616606 17154618

[B52] Weckx S Carlon E Vuyst LD Hummelen PV Thermodynamic Behavior of Short Oligonucleotides in Microarray Hybridizations Can Be Described Using Gibbs Free Energy in a Nearest-Neighbor Model J Phys Chem B 2007 111 13583 13590 10.1021/jp075197x 17994724

[B53] Pozhitkov A Noble PA Domazet-Losǒ T Nolte AW Sonnenberg R Staehler P Beier M Tautz D Tests of rRNA hybridization to microarrays suggest that hybridization characteristics of oligonucleotide probes for species discrimination cannot be predicted Nucleic Acids Res 2006 34 e66 10.1093/nar/gkl133 16707658 PMC1463897

[B54] Wick LM Rouillard JM Whittam TS Gulari E Tiedje JM Hashsham SA On-chip non-equilibrium dissociation curves and dissociation rate constants as methods to assess specificity of oligonucleotide probes Nucleic Acids Res 2006 34 e26 10.1093/nar/gnj024 16478712 PMC1369288

[B55] Yao D Kim J Yu F Nielsen PE Sinner EK Knoll W Surface Density Dependence of PCR Amplicon Hybridization on PNA/DNA Probe Layers Biophys J 2005 88 2745 2751 10.1529/biophysj.104.051656 15665129 PMC1305370

[B56] Shchepinov MS Case-Green SC Southern EM Steric factors influencing hybridisation of nucleic acids to oligonucleotide arrays Nucleic Acids Res 1997 25 1155 1161 10.1093/nar/25.6.1155 9092624 PMC146580

[B57] Peterson AW Wolf LK Georgiadis RM Hybridization of Mismatched or Partially Matched DNA at Surfaces J Am Chem Soc 2002 124 14601 14607 10.1021/ja0279996 12465970

[B58] Fixe F Dufva M Telleman P Christensen CBV Functionalization of poly(methyl methacrylate) (PMMA) as a substrate for DNA microarrays Nucleic Acids Res 2004 32 e9 10.1093/nar/gng157 14718554 PMC373302

[B59] Zhang L Wu C Carta R Zhao H Free energy of DNA duplex formation on short oligonucleotide microarrays Nucleic Acids Res 2007 35 e18 10.1093/nar/gkl1064 17169993 PMC1807971

[B60] Zuker M Mfold web server for nucleic acid folding and hybridization prediction Nucleic Acids Res 2003 31 13 3406 3415 10.1093/nar/gkg595 12824337 PMC169194

[B61] Dimitrov RA Zuker M Prediction of Hybridization and Melting for Double-Stranded Nucleic Acids Biophysical Journal 2004 87 215 226 10.1529/biophysj.103.020743 15240459 PMC1304344

[B62] Andronescu M Aguirre-Hern'eandez R Condon A Hoos HH RNAsoft: a suite of RNA secondary structure prediction and design software tools Nucleic Acids Res 2003 31 3416 3422 10.1093/nar/gkg612 12824338 PMC169018

[B63] Andronescu M Zhang ZC Condon A Secondary Structure Prediction of Interacting RNA Molecules J Mol Biol 2005 345 987 1001 10.1016/j.jmb.2004.10.082 15644199

[B64] Vainrub A Pettitt BM Coulomb blockage of hybridization in two-dimensional DNA arrays Phys Rev E 2002 66 041905 10.1103/PhysRevE.66.041905 12443233

[B65] Vainrub A Pettitt BM Surface Electrostatic Effects in Oligonucleotide Microarrays: Control and Optimization of Binding Thermodynamics Biopolymers 2003 68 265 270 10.1002/bip.10271 12548628

[B66] Poulsen L Soe MJ Snakenborg D Moller LB Dufva M Multi-stringency wash of partially hybridized 60-mer probes reveals that the stringency along the probe decreases with distance from the microarray surface Nucleic Acids Res 2008 36 e132 10.1093/nar/gkn600 18805905 PMC2582620

[B67] Wu P ichi Nakano S Sugimoto N Temperature dependence of thermodynamic properties for DNA/DNA and RNA/DNA duplex formation Eur J Biochem 2002 269 2821 2830 10.1046/j.1432-1033.2002.02970.x 12071944

[B68] Peplies J Glöckner FO Amann R Optimization Strategies for DNA Microarray-Based Detection of Bacteria with 16S rRNA-Targeting Oligonucleotide Probes Appl Environ Microbiol 2003 69 1397 1407 10.1128/AEM.69.3.1397-1407.2003 12620822 PMC150098

[B69] Spellman PT Miller M Stewart J Troup C Sarkans U Chervitz S Bernhart D Sherlock G Ball C Lepage M Swiatek M Marks W Goncalves J Markel S Iordan D Shojatalab M Pizarro A White J Hubley R Deutsch E Senger M Aronow BJ Robinson A Bassett D Christian J Stoeckert J Brazma A Design and implementation of microarray gene expression markup language (MAGE-ML) Genome Biol 2002 3 research0046.1 research0046.9 10.1186/gb-2002-3-9-research0046 PMC126871 12225585

[B70] Rayner T Rocca-Serra P Spellman P Causton H Farne A Holloway E Irizarry R Liu J Maier D Miller M Petersen K Quackenbush J Sherlock G Stoeckert C White J Whetzel P Wymore F Parkinson H Sarkans U Ball C Brazma A A simple spreadsheet-based, MIAME-supportive format for microarray data: MAGE-TAB BMC Bioinformatics 2006 7 489 10.1186/1471-2105-7-489 17087822 PMC1687205

[B71] Gao Y Wolf LK Georgiadis RM Secondary structure effects on DNA hybridization kinetics: a solution versus surface comparison Nucleic Acids Res 2006 34 3370 3377 10.1093/nar/gkl422 16822858 PMC1488884

[B72] Kong DS Carr PA Chen L Zhang S Jacobson JM Parallel gene synthesis in a microfluidic devixe Nuc Acids Res 2007 35 8 e61 10.1093/nar/gkm121 PMC1885655 17405768

[B73] Hedge P Qi R Abernathy K Gay C Dharap S Renee Gaspard JEH Snesrud E Lee N Quackenbush J A concise guide to cDNA microarray analysis BioTechniques 2000 29 3 548 562 10997270 10.2144/00293bi01

[B74] Smith L Underhill P Pritchard C Tymowska-Lalanne Z Abdul-Hussein S Hilton H Winchester L Williams D Freeman T Webb S Greenfield A Single primer amplification (SPA) of cDNA for microarray expression analysis Nucleic Acids Res 2003 31 9 e9 10.1093/nar/gng009 12560512 PMC149221

[B75] SantaLucia J A unified view of polymer, dumbbell, and oligonucleotide DNA nearest-neighbor thermodynamics Proc Natl Acad Sci USA 1998 95 1460 1465 10.1073/pnas.95.4.1460 9465037 PMC19045

[B76] Cho RJ Campbell MJ Winzeler EA Steinmetz L Conway A Wodicka L Wolfsburg TG Gabrielian AE Landsman D Lockhart DJ Davis RW A genome-wide transcriptional analysis of the mitotic cell cycle Molecular Cell 1998 2 65 73 10.1016/S1097-2765(00)80114-8 9702192

[B77] Onsager L Reciprocal relations in irreversible processes. II Phys Rev 1931 38 2265 2279 10.1103/PhysRev.38.2265

[B78] Tolman RC The Principles of Statistical Mechanics 1938 Oxford University Press, London, UK

[B79] Colquhoun D Dowsland KA Beato M Plested AJR How to Impose Microscopic Reversibility in Complex Reaction Mechanisms Biophys J 2004 86 3510 3518 10.1529/biophysj.103.038679 15189850 PMC1304255

[B80] DeLisi C Wiegel FW Effect of nonspecific forces and finite receptor number on rate constants of ligand-cell bound-receptor interactions Proc Natl Acad Sci USA 1981 78 5569 5572 10.1073/pnas.78.9.5569 6946494 PMC348789

[B81] Smoluchowski M Versuch einer mathematischen theorie der koagulationskinetic kolloider lösungen Z Phys Chem 1917 92 129 168

