# Supplementary material for: An efficient algorithm for the stochastic simulation of the hybridization of DNA to microarrays
Source: BMC Bioinformatics. 2009 Dec 10;10:411. doi: 10.1186/1471-2105-10-411 (PMC2805644; doi:10.1186/1471-2105-10-411)
Supplement: Additional file 2 — Arslan_Laurenzi_Supplemental. This file lists the populations of full length cDNA molecules used in all simulations conducted with N = 1000 probe molecules per feature. The concentrations of each cDNA species may be calculated (in number of molecules/nL) by dividing these populations by the hybridization volume corresponding to this probe population (0.275 nL). The sequences of these cDNA molecules may be obtained from the Saccharomyces Genome Database http://www.yeastgenome.org; we have utilized the November 10, 2006 version of the yeast genome. [file 1471-2105-10-411-S2.PDF]

The average (and standard deviation) of the populations of each hybrid are provided in this worksheet, as calculated from the results provided in Supplemental Table 3.

Probe Name	cDNA	Hybrid Population (Average)	Hybrid Population (Std. Dev.)
A_06_P1002	Q0010	13.000	0.000
A_06_P1002	Q0017	14.000	0.000
A_06_P1003	Q0045	100.000	0.000
A_06_P1004	Q0050	98.000	0.000
A_06_P1005	Q0055	8.000	0.000
A_06_P1006	Q0060	1.000	0.000
A_06_P1007	Q0065	137.400	0.894
A_06_P1008	Q0070	10.000	0.000
A_06_P1009	Q0075	56.000	0.000
A_06_P1010	Q0080	49.000	0.000
A_06_P1011	Q0085	201.000	0.000
A_06_P1012	Q0105	240.000	0.000
A_06_P1013	Q0110	16.000	0.000
A_06_P1014	Q0115	38.000	0.000
A_06_P1015	Q0120	99.400	0.894
A_06_P1016	Q0130	44.000	0.000
A_06_P1017	Q0140	120.000	0.000
A_06_P1018	Q0160	180.600	1.140
A_06_P1019	Q0182	56.000	0.000
A_06_P1020	Q0250	27.000	0.000
A_06_P1021	Q0255	73.000	0.000
A_06_P1022	Q0275	25.000	0.000
A_06_P1023	Q0297	123.000	0.000
A_06_P1024	YAL001C	258.000	0.000
A_06_P1025	YAL002W	53.000	0.000
A_06_P1026	YAL003W	43.000	0.000
A_06_P1027	YAL004W	13.000	0.000
A_06_P1028	YAL005C	152.000	0.000
A_06_P1029	YAL007C	28.000	0.000
A_06_P1030	YAL008W	79.000	0.000
A_06_P1031	YAL009W	97.000	0.000
A_06_P1032	YAL010C	26.000	0.000
A_06_P1033	YAL011W	26.000	0.000
A_06_P1034	YAL012W	6.000	0.000
A_06_P1035	YAL013W	97.000	0.000
A_06_P1036	YAL014C	22.000	0.000
A_06_P1037	YAL015C	121.000	0.000
A_06_P1038	YAL016W	58.000	0.000
A_06_P1039	YAL017W	22.000	0.000
A_06_P1040	YAL018C	100.000	0.000
A_06_P1041	YAL019W	2.000	0.000
A_06_P1042	YAL020C	16.000	0.000
A_06_P1043	YAL021C	54.000	0.000
A_06_P1044	YAL022C	220.000	0.000
A_06_P1045	YAL023C	78.000	0.000
A_06_P1046	YAL024C	178.000	0.000
A_06_P1047	YAL025C	99.000	0.000
A_06_P1048	YAL026C	23.000	0.000
A_06_P1048	YAL026C-A	96.000	0.000
A_06_P1049	YAL027W	6.000	0.000
A_06_P1050	YAL028W	236.000	0.000
A_06_P1051	YAL029C	102.000	0.000
A_06_P1052	YAL030W	44.000	0.000
A_06_P1053	YAL031C	13.000	0.000

A_06_P1054	YAL032C	31.000	0.000
A_06_P1055	YAL033W	12.000	0.000
A_06_P1056	YAL034C	22.000	0.000
A_06_P1057	YAL034W-A	41.000	0.000
A_06_P1058	YAL034C-B	36.000	0.000
A_06_P1059	YAL035W	76.000	0.000
A_06_P1059	YHR214C-B	0.200	0.447
A_06_P1060	YAL036C	40.000	0.000
A_06_P1061	YAL037W	77.000	0.000
A_06_P1062	YAL038W	26.000	0.000
A_06_P1063	YAL039C	53.000	0.000
A_06_P1064	YAL040C	12.000	0.000
A_06_P1065	YAL041W	40.000	0.000
A_06_P1066	YAL042W	117.000	0.000
A_06_P1067	YAL043C	232.000	0.000
A_06_P1068	YAL042C-A	742.000	0.000
A_06_P1069	YAL044C	27.000	0.000
A_06_P1070	YAL044W-A	7.000	0.000
A_06_P1071	YAL045C	418.000	0.000
A_06_P1072	YAL046C	65.000	0.000
A_06_P1073	YAL047C	13.000	0.000
A_06_P1074	YAL048C	22.000	0.000
A_06_P1075	YAL049C	8.000	0.000
A_06_P1076	YAL051W	11.000	0.000
A_06_P1077	YAL053W	400.000	0.000
A_06_P1078	YAL054C	2.000	0.000
A_06_P1079	YAL055W	25.000	0.000
A_06_P1080	YAL056W	116.000	0.000
A_06_P1081	YAL056C-A	41.000	0.000
A_06_P1082	YAL058W	155.000	0.000
A_06_P1083	YAL059W	56.000	0.000
A_06_P1084	YAL060W	29.000	0.000
A_06_P1085	YAL061W	13.000	0.000
A_06_P1086	YAL062W	37.000	0.000
A_06_P1087	YAL063C	10.800	0.447
A_06_P1088	YAL064C-A	3.000	0.000
A_06_P1089	YAL064W	24.000	0.000
A_06_P1090	YAL064W-B	37.000	0.000
A_06_P1091	YAL065C	70.800	0.447
A_06_P1092	YAL066W	106.000	0.000
A_06_P1093	YAL067C	32.000	0.000
A_06_P1094	YAL068C	1.000	1.000
A_06_P1094	YLL064C	0.600	0.548
A_06_P1094	YLR461W	2.000	1.000
A_06_P1094	YOL161C	3.000	2.000
A_06_P1095	YAL069W	52.000	0.000
A_06_P1095	YCR108C	15.800	2.864
A_06_P1095	YOR394C-A	104.600	3.435
A_06_P1096	YAR002C-A	80.000	0.000
A_06_P1097	YAR002W	124.000	0.000
A_06_P1098	YAR003W	43.000	0.000
A_06_P1099	YAR007C	187.000	0.000
A_06_P1100	YAR008W	103.000	0.000
A_06_P1101	YAR014C	228.000	0.000
A_06_P1102	YAR015W	226.000	0.000

A_06_P1103	YAR018C	36.000	0.000
A_06_P1104	YAR019C	58.000	0.000
A_06_P1105	YAR020C	138.000	0.000
A_06_P1106	YAR023C	8.000	0.000
A_06_P1107	YAR027W	28.000	0.000
A_06_P1108	YAR028W	9.000	0.000
A_06_P1109	YAR029W	112.000	0.000
A_06_P1110	YAR030C	41.000	0.000
A_06_P1111	YAR031W	46.000	0.000
A_06_P1112	YAR033W	8.000	0.000
A_06_P1113	YAR035W	13.000	0.000
A_06_P1114	YAR042W	29.000	0.000
A_06_P1116	YAR047C	15.000	0.000
A_06_P1117	YAR050W	34.400	1.517
A_06_P1118	YAR053W	21.000	0.000
A_06_P1119	YAR060C	16.800	3.347
A_06_P1119	YHR212C	14.600	1.140
A_06_P1120	YAR061W	60.000	0.000
A_06_P1120	YHR212W-A	21.000	0.000
A_06_P1121	YAR050W	0.600	0.548
A_06_P1121	YAR062W	54.800	4.087
A_06_P1121	YHR213W	22.600	3.362
A_06_P1122	YAR064W	10.000	0.000
A_06_P1122	YHR213W-B	18.000	0.000
A_06_P1123	YAR066W	64.400	0.548
A_06_P1123	YHR214W	0.800	0.447
A_06_P1124	YAR068W	16.200	0.837
A_06_P1124	YHR214W-A	3.200	1.304
A_06_P1125	YAR069C	50.000	0.000
A_06_P1125	YHR214C-D	10.000	0.000
A_06_P1126	YAR070C	266.000	0.000
A_06_P1126	YHR214C-E	12.000	0.000
A_06_P1127	YAR071W	37.000	0.707
A_06_P1128	YAR073W	89.000	0.000
A_06_P1129	YAR075W	180.000	0.000
A_06_P1130	YBL001C	276.000	0.000
A_06_P1131	YBL002W	10.000	0.000
A_06_P1132	YBL003C	7.000	0.000
A_06_P1133	YBL004W	8.000	0.000
A_06_P1134	YBL005W	23.000	0.000
A_06_P1135	YBL006C	9.000	0.000
A_06_P1136	YBL007C	117.000	0.000
A_06_P1137	YBL008W	38.000	0.000
A_06_P1138	YBL009W	159.000	0.000
A_06_P1139	YBL010C	345.000	0.000
A_06_P1140	YBL011W	19.000	0.000
A_06_P1141	YBL012C	117.000	0.000
A_06_P1142	YBL013W	11.000	0.000
A_06_P1143	YBL014C	35.000	0.000
A_06_P1144	YBL015W	4.000	0.000
A_06_P1145	YBL016W	28.000	0.000
A_06_P1146	YBL017C	13.000	0.000
A_06_P1147	YBL018C	19.000	0.000
A_06_P1148	YBL019W	30.000	0.000
A_06_P1149	YBL020W	54.000	0.000

A_06_P1150	YBL021C	31.000	0.000
A_06_P1151	YBL022C	10.000	0.000
A_06_P1152	YBL023C	3.000	0.000
A_06_P1153	YBL024W	66.000	0.000
A_06_P1154	YBL025W	39.000	0.000
A_06_P1155	YBL026W	6.000	0.000
A_06_P1156	YBL027W	26.000	0.000
A_06_P1157	YBL028C	129.000	0.000
A_06_P1158	YBL029C-A	265.000	0.000
A_06_P1159	YBL029W	9.000	0.000
A_06_P1160	YBL030C	101.000	0.000
A_06_P1161	YBL031W	47.000	0.000
A_06_P1162	YBL032W	66.000	0.000
A_06_P1163	YBL033C	35.000	0.000
A_06_P1164	YBL034C	31.000	0.000
A_06_P1165	YBL035C	49.000	0.000
A_06_P1166	YBL036C	25.000	0.000
A_06_P1167	YBL037W	409.000	0.000
A_06_P1168	YBL038W	27.000	0.000
A_06_P1169	YBL039C	110.000	0.000
A_06_P1170	YBL040C	4.000	0.000
A_06_P1171	YBL041W	20.000	0.000
A_06_P1172	YBL042C	27.000	0.000
A_06_P1173	YBL043W	62.000	0.000
A_06_P1174	YBL044W	72.000	0.000
A_06_P1175	YBL045C	562.000	0.000
A_06_P1176	YBL046W	33.000	0.000
A_06_P1177	YBL047C	61.000	0.000
A_06_P1178	YBL048W	27.000	0.000
A_06_P1179	YBL049W	15.000	0.000
A_06_P1180	YBL050W	7.000	0.000
A_06_P1181	YBL051C	70.000	0.000
A_06_P1182	YBL052C	33.000	0.000
A_06_P1183	YBL053W	23.000	0.000
A_06_P1184	YBL054W	21.000	0.000
A_06_P1185	YBL055C	180.000	0.000
A_06_P1186	YBL056W	52.000	0.000
A_06_P1187	YBL057C	18.000	0.000
A_06_P1188	YBL058W	56.000	0.000
A_06_P1189	YBL059C-A	5.000	0.000
A_06_P1190	YBL059W	166.000	0.000
A_06_P1191	YBL060W	21.000	0.000
A_06_P1192	YBL061C	7.000	0.000
A_06_P1193	YBL062W	35.000	0.000
A_06_P1194	YBL063W	552.000	0.000
A_06_P1195	YBL064C	870.000	0.000
A_06_P1196	YBL065W	112.000	0.000
A_06_P1197	YBL066C	101.000	0.000
A_06_P1198	YBL067C	27.000	0.000
A_06_P1199	YBL068W	162.000	0.000
A_06_P1200	YBL069W	39.000	0.000
A_06_P1201	YBL070C	94.000	0.000
A_06_P1202	YBL071C	9.000	0.000
A_06_P1203	YBL071W-A	68.000	0.000
A_06_P1204	YBL072C	11.000	0.000

A_06_P1205	YBL073W	12.000	0.000
A_06_P1206	YBL074C	147.000	0.000
A_06_P1207	YBL075C	35.000	0.000
A_06_P1208	YBL076C	98.000	0.000
A_06_P1209	YBL077W	188.000	0.000
A_06_P1210	YBL078C	22.000	0.000
A_06_P1211	YBL079W	128.000	0.000
A_06_P1212	YBL080C	70.000	0.000
A_06_P1213	YBL081W	494.000	0.000
A_06_P1214	YBL082C	0.200	0.447
A_06_P1215	YBL082C	1.800	0.447
A_06_P1215	YBL083C	36.000	0.000
A_06_P1216	YBL084C	53.000	0.000
A_06_P1217	YBL085W	218.000	0.000
A_06_P1218	YBL086C	154.000	0.000
A_06_P1219	YBL087C	4.000	0.000
A_06_P1220	YBL088C	52.000	0.000
A_06_P1221	YBL089W	3.000	0.000
A_06_P1222	YBL090W	42.000	0.000
A_06_P1223	YBL091C	125.000	0.000
A_06_P1224	YBL091C-A	216.000	0.000
A_06_P1225	YBL092W	72.000	0.000
A_06_P1226	YBL093C	13.000	0.000
A_06_P1227	YBL094C	69.000	0.000
A_06_P1228	YBL095W	385.000	0.000
A_06_P1229	YBL096C	46.000	0.000
A_06_P1230	YBL097W	10.000	0.000
A_06_P1231	YBL098W	4.000	0.000
A_06_P1232	YBL099W	196.000	0.000
A_06_P1233	YBL100C	41.000	0.000
A_06_P1234	YBL101C	39.000	0.000
A_06_P1235	YBL102W	291.000	0.000
A_06_P1236	YBL103C	94.000	0.000
A_06_P1237	YBL104C	10.000	0.000
A_06_P1238	YBL105C	286.000	0.000
A_06_P1239	YBL106C	135.000	0.000
A_06_P1240	YBL107C	121.000	0.000
A_06_P1241	YBL107W-A	5.000	0.000
A_06_P1241	YOR192C-C	2.000	1.000
A_06_P1242	YAL068C	10.800	3.114
A_06_P1242	YBL108C-A	13.800	2.490
A_06_P1242	YBR301W	3.000	2.000
A_06_P1242	YEL049W	5.400	2.191
A_06_P1242	YGL261C	4.400	1.140
A_06_P1242	YGR294W	12.600	3.362
A_06_P1242	YHL046C	1.000	1.225
A_06_P1242	YIL176C	259.400	15.043
A_06_P1242	YJL223C	10.000	1.581
A_06_P1243	YBL108W	54.000	0.000
A_06_P1244	YBL109W	100.000	0.000
A_06_P1244	YLL066W-B	8.800	0.447
A_06_P1244	YLR466C-B	85.000	0.000
A_06_P1244	YNL338W	26.000	0.000
A_06_P1245	YBL111C	36.400	4.827
A_06_P1245	YHR218W	37.800	3.834

A_06_P1245	YPR204W	1.000	0.707
A_06_P1246	YBL112C	44.000	0.000
A_06_P1246	YDR545W	1.400	0.548
A_06_P1246	YEL077C	40.400	3.715
A_06_P1246	YER190W	1.600	1.517
A_06_P1246	YGR296W	0.600	0.894
A_06_P1246	YHR218W-A	4.000	0.000
A_06_P1246	YJL225C	0.200	0.447
A_06_P1246	YLL067C	0.600	0.548
A_06_P1246	YLR467W	0.200	0.447
A_06_P1246	YML133C	0.200	0.447
A_06_P1246	YNL339C	0.600	0.894
A_06_P1246	YOR396W	11.400	1.517
A_06_P1246	YPR204W	23.800	4.970
A_06_P1247	YBL113C	1.800	1.789
A_06_P1247	YDR545W	0.400	0.894
A_06_P1247	YEL077C	0.200	0.447
A_06_P1247	YHL050C	0.200	0.447
A_06_P1247	YLR467W	0.200	0.447
A_06_P1247	YNL339C	0.200	0.447
A_06_P1247	YOR396W	0.200	0.447
A_06_P1247	YPR204W	2.600	0.548
A_06_P1248	YBR001C	40.000	0.000
A_06_P1249	YBR002C	74.000	0.000
A_06_P1250	YBR003W	43.000	0.000
A_06_P1251	YBR004C	50.000	0.000
A_06_P1252	YBR005W	21.000	0.000
A_06_P1253	YBR006W	53.000	0.000
A_06_P1254	YBR007C	53.000	0.000
A_06_P1255	YBR008C	67.000	0.000
A_06_P1256	YBR009C	40.000	0.000
A_06_P1256	YNL030W	2.200	1.483
A_06_P1257	YBR010W	9.000	0.000
A_06_P1258	YBR011C	188.000	0.000
A_06_P1259	YBR012C	6.000	0.000
A_06_P1260	YBR013C	39.000	0.000
A_06_P1261	YBR014C	27.000	0.000
A_06_P1262	YBR015C	264.000	0.000
A_06_P1263	YBR016W	19.000	0.000
A_06_P1264	YBR017C	104.000	0.000
A_06_P1265	YBR018C	54.000	0.000
A_06_P1266	YBR019C	218.000	0.000
A_06_P1267	YBR020W	70.000	0.000
A_06_P1268	YBR021W	292.000	0.000
A_06_P1269	YBR022W	5.000	0.000
A_06_P1270	YBR023C	53.000	0.000
A_06_P1271	YBR024W	4.000	0.000
A_06_P1272	YBR025C	88.000	0.000
A_06_P1273	YBR026C	18.000	0.000
A_06_P1274	YBR027C	11.000	0.000
A_06_P1275	YBR028C	23.000	0.000
A_06_P1276	YBR029C	8.000	0.000
A_06_P1277	YBR030W	101.000	0.000
A_06_P1278	YBR031W	12.000	0.000
A_06_P1279	YBR032W	251.000	0.000

A_06_P1280	YBR033W	194.000	0.000
A_06_P1281	YBR034C	10.000	0.000
A_06_P1282	YBR035C	19.000	0.000
A_06_P1283	YBR036C	36.000	0.000
A_06_P1284	YBR037C	76.000	0.000
A_06_P1285	YBR038W	142.000	0.000
A_06_P1286	YBR039W	393.000	0.000
A_06_P1287	YBR040W	49.000	0.000
A_06_P1288	YBR041W	274.000	0.000
A_06_P1289	YBR042C	20.000	0.000
A_06_P1290	YBR043C	82.000	0.000
A_06_P1291	YBR044C	251.000	0.000
A_06_P1292	YBR045C	171.000	0.000
A_06_P1293	YBR046C	7.000	0.000
A_06_P1294	YBR047W	63.000	0.000
A_06_P1295	YBR048W	15.000	0.000
A_06_P1296	YBR049C	25.000	0.000
A_06_P1297	YBR050C	11.000	0.000
A_06_P1298	YBR051W	82.000	0.000
A_06_P1299	YBR052C	18.000	0.000
A_06_P1300	YBR053C	21.000	0.000
A_06_P1301	YBR054W	81.000	0.000
A_06_P1302	YBR055C	447.000	0.000
A_06_P1303	YBR056W	5.000	0.000
A_06_P1304	YBR057C	4.000	0.000
A_06_P1305	YBR058C	30.000	0.000
A_06_P1306	YBR058C-A	68.000	0.000
A_06_P1307	YBR059C	3.000	0.000
A_06_P1308	YBR060C	23.000	0.000
A_06_P1309	YBR061C	16.000	0.000
A_06_P1310	YBR062C	21.000	0.000
A_06_P1311	YBR063C	55.000	0.000
A_06_P1312	YBR064W	20.000	0.000
A_06_P1313	YBR065C	197.000	0.000
A_06_P1314	YBR066C	20.000	0.000
A_06_P1315	YBR067C	84.000	0.000
A_06_P1316	YBR068C	146.000	0.000
A_06_P1317	YBR069C	19.000	0.000
A_06_P1318	YBR070C	5.000	0.000
A_06_P1319	YBR071W	94.000	0.000
A_06_P1320	YBR072W	98.000	0.000
A_06_P1321	YBR073W	8.000	0.000
A_06_P1322	YBR074W	0.400	0.548
A_06_P1323	YBR074W	87.600	0.548
A_06_P1324	YBR076W	81.000	0.000
A_06_P1325	YBR077C	15.000	0.000
A_06_P1326	YBR078W	106.000	0.000
A_06_P1327	YBR079C	320.000	0.000
A_06_P1328	YBR080C	4.000	0.000
A_06_P1329	YBR081C	174.000	0.000
A_06_P1330	YBR082C	25.000	0.000
A_06_P1331	YBR083W	10.000	0.000
A_06_P1332	YBR084C-A	4.000	0.000
A_06_P1333	YBR084W	5.000	0.000
A_06_P1334	YBR085C-A	37.000	0.000

A_06_P1335	YBR085W	17.000	0.000
A_06_P1336	YBR086C	667.000	0.000
A_06_P1337	YBR087W	11.000	0.000
A_06_P1338	YBR088C	147.000	0.000
A_06_P1339	YBR089C-A	151.000	0.000
A_06_P1340	YBR089W	31.000	0.000
A_06_P1341	YBR090C	173.000	0.000
A_06_P1342	YBR091C	41.000	0.000
A_06_P1343	YBR092C	73.000	0.000
A_06_P1344	YBR093C	6.000	0.000
A_06_P1345	YBR094W	131.000	0.000
A_06_P1346	YBR095C	40.000	0.000
A_06_P1347	YBR096W	29.000	0.000
A_06_P1348	YBR097W	73.000	0.000
A_06_P1350	YBR099C	30.000	0.000
A_06_P1351	YBR098W	36.000	0.000
A_06_P1352	YBR101C	66.000	0.000
A_06_P1353	YBR102C	13.000	0.000
A_06_P1354	YBR103C-A	23.000	0.000
A_06_P1355	YBR103W	28.000	0.000
A_06_P1356	YBR104W	41.000	0.000
A_06_P1357	YBR105C	193.000	0.000
A_06_P1358	YBR106W	39.000	0.000
A_06_P1359	YBR107C	41.000	0.000
A_06_P1360	YBR108W	85.000	0.000
A_06_P1361	YBR109C	62.000	0.000
A_06_P1362	YBR110W	55.000	0.000
A_06_P1363	YBR111C	39.000	0.000
A_06_P1364	YBR112C	67.000	0.000
A_06_P1365	YBR113W	835.000	0.000
A_06_P1366	YBR114W	56.000	0.000
A_06_P1367	YBR115C	22.000	0.000
A_06_P1368	YBR116C	77.000	0.000
A_06_P1369	YBR117C	31.000	0.000
A_06_P1370	YBR118W	15.000	0.000
A_06_P1370	YPR080W	11.800	2.775
A_06_P1371	YBR119W	5.000	0.000
A_06_P1372	YBR120C	20.000	0.000
A_06_P1373	YBR121C	142.000	0.000
A_06_P1374	YBR122C	29.000	0.000
A_06_P1375	YBR123C	10.000	0.000
A_06_P1376	YBR124W	21.000	0.000
A_06_P1377	YBR125C	51.000	0.000
A_06_P1378	YBR126C	238.000	0.000
A_06_P1379	YBR127C	636.000	0.000
A_06_P1380	YBR128C	110.000	0.000
A_06_P1381	YBR129C	17.000	0.000
A_06_P1382	YBR130C	20.000	0.000
A_06_P1383	YBR131W	39.000	0.000
A_06_P1384	YBR132C	148.000	0.000
A_06_P1385	YBR133C	147.000	0.000
A_06_P1386	YBR134W	47.000	0.000
A_06_P1387	YBR135W	589.000	0.000
A_06_P1388	YBR136W	56.000	0.000
A_06_P1389	YBR137W	110.000	0.000

A_06_P1390	YBR138C	41.000	0.000
A_06_P1391	YBR139W	26.000	0.000
A_06_P1392	YBR140C	23.000	0.000
A_06_P1393	YBR141C	12.000	0.000
A_06_P1394	YBR142W	119.000	0.000
A_06_P1395	YBR143C	51.000	0.000
A_06_P1396	YBR144C	51.000	0.000
A_06_P1397	YBR145W	14.000	0.000
A_06_P1398	YBR146W	27.000	0.000
A_06_P1399	YBR147W	17.000	0.000
A_06_P1400	YBR148W	13.000	0.000
A_06_P1401	YBR149W	43.000	0.000
A_06_P1402	YBR150C	78.000	0.000
A_06_P1403	YBR151W	412.000	0.000
A_06_P1404	YBR152W	26.000	0.000
A_06_P1405	YBR153W	402.000	0.000
A_06_P1406	YBR154C	61.000	0.000
A_06_P1407	YBR155W	33.000	0.000
A_06_P1408	YBR156C	14.000	0.000
A_06_P1409	YBR157C	1.000	0.000
A_06_P1410	YBR158W	47.000	0.000
A_06_P1411	YBR159W	62.000	0.000
A_06_P1412	YBR160W	180.000	0.000
A_06_P1413	YBR161W	39.000	0.000
A_06_P1414	YBR162C	13.000	0.000
A_06_P1415	YBR162W-A	41.000	0.000
A_06_P1416	YBR163W	19.000	0.000
A_06_P1417	YBR164C	11.000	0.000
A_06_P1418	YBR165W	35.000	0.000
A_06_P1419	YBR166C	70.000	0.000
A_06_P1420	YBR167C	20.000	0.000
A_06_P1421	YBR168W	47.000	0.000
A_06_P1422	YBR169C	52.000	0.000
A_06_P1423	YBR170C	55.000	0.000
A_06_P1424	YBR171W	14.000	0.000
A_06_P1425	YBR172C	14.000	0.000
A_06_P1426	YBR173C	195.000	0.000
A_06_P1427	YBR174C	407.000	0.000
A_06_P1428	YBR175W	29.000	0.000
A_06_P1429	YBR176W	174.000	0.000
A_06_P1430	YBR177C	39.000	0.000
A_06_P1431	YBR178W	44.000	0.000
A_06_P1432	YBR179C	353.000	0.000
A_06_P1433	YBR180W	27.000	0.000
A_06_P1434	YBR181C	22.000	0.000
A_06_P1434	YPL090C	0.600	0.894
A_06_P1435	YBR182C	57.000	0.000
A_06_P1436	YBR183W	8.000	0.000
A_06_P1437	YBR184W	5.000	0.000
A_06_P1438	YBR185C	264.000	0.000
A_06_P1439	YBR186W	21.000	0.000
A_06_P1440	YBR187W	164.000	0.000
A_06_P1441	YBR188C	53.000	0.000
A_06_P1442	YBR189W	138.000	0.000
A_06_P1443	YBR190W	63.000	0.000

A_06_P1444	YBR191W	18.000	0.000
A_06_P1445	YBR192W	143.000	0.000
A_06_P1446	YBR193C	29.000	0.000
A_06_P1447	YBR194W	394.000	0.000
A_06_P1448	YBR195C	29.000	0.000
A_06_P1449	YBR196C	30.000	0.000
A_06_P1450	YBR197C	75.000	0.000
A_06_P1451	YBR198C	36.000	0.000
A_06_P1452	YBR199W	67.000	0.000
A_06_P1453	YBR200W	64.000	0.000
A_06_P1454	YBR201W	110.000	0.000
A_06_P1455	YBR202W	13.000	0.000
A_06_P1456	YBR203W	13.000	0.000
A_06_P1457	YBR204C	19.000	0.000
A_06_P1458	YBR205W	6.000	0.000
A_06_P1459	YBR206W	41.000	0.000
A_06_P1460	YBR207W	15.000	0.000
A_06_P1461	YBR208C	200.000	0.000
A_06_P1462	YBR209W	217.000	0.000
A_06_P1463	YBR210W	13.000	0.000
A_06_P1464	YBR211C	65.000	0.000
A_06_P1465	YBR212W	59.000	0.000
A_06_P1466	YBR213W	170.000	0.000
A_06_P1467	YBR214W	15.000	0.000
A_06_P1468	YBR215W	100.000	0.000
A_06_P1469	YBR216C	87.000	0.000
A_06_P1470	YBR217W	5.000	0.000
A_06_P1471	YBR218C	576.000	0.000
A_06_P1472	YBR219C	13.000	0.000
A_06_P1473	YBR220C	17.000	0.000
A_06_P1474	YBR221C	164.000	0.000
A_06_P1475	YBR222C	10.000	0.000
A_06_P1476	YBR223C	34.000	0.000
A_06_P1477	YBR224W	6.000	0.000
A_06_P1478	YBR225W	92.000	0.000
A_06_P1479	YBR226C	14.000	0.000
A_06_P1480	YBR227C	34.000	0.000
A_06_P1481	YBR228W	14.000	0.000
A_06_P1482	YBR229C	11.000	0.000
A_06_P1483	YBR230C	16.000	0.000
A_06_P1484	YBR231C	80.000	0.000
A_06_P1485	YBR232C	8.000	0.000
A_06_P1486	YBR233W	30.000	0.000
A_06_P1487	YBR233W-A	46.000	0.000
A_06_P1488	YBR234C	3.000	0.000
A_06_P1489	YBR235W	38.000	0.000
A_06_P1490	YBR236C	5.000	0.000
A_06_P1491	YBR237W	408.000	0.000
A_06_P1492	YBR238C	9.000	0.000
A_06_P1493	YBR239C	8.000	0.000
A_06_P1494	YBR240C	22.000	0.000
A_06_P1495	YBR241C	64.000	0.000
A_06_P1496	YBR242W	151.000	0.000
A_06_P1497	YBR243C	68.000	0.000
A_06_P1498	YBR244W	46.000	0.000

A_06_P1499	YBR245C	24.000	0.000
A_06_P1500	YBR246W	430.000	0.000
A_06_P1501	YBR247C	176.000	0.000
A_06_P1502	YBR248C	111.000	0.000
A_06_P1503	YBR249C	43.000	0.000
A_06_P1504	YBR250W	22.000	0.000
A_06_P1505	YBR251W	26.000	0.000
A_06_P1506	YBR252W	25.000	0.000
A_06_P1507	YBR253W	5.000	0.000
A_06_P1508	YBR254C	17.000	0.000
A_06_P1509	YBR255W	39.000	0.000
A_06_P1510	YBR256C	85.000	0.000
A_06_P1511	YBR257W	40.000	0.000
A_06_P1512	YBR258C	164.000	0.000
A_06_P1513	YBR259W	29.000	0.000
A_06_P1514	YBR260C	200.000	0.000
A_06_P1515	YBR261C	12.000	0.000
A_06_P1516	YBR262C	159.000	0.000
A_06_P1517	YBR263W	159.000	0.000
A_06_P1518	YBR264C	81.000	0.000
A_06_P1519	YBR265W	26.000	0.000
A_06_P1520	YBR266C	64.000	0.000
A_06_P1521	YBR267W	157.000	0.000
A_06_P1522	YBR268W	27.000	0.000
A_06_P1523	YBR269C	14.000	0.000
A_06_P1524	YBR270C	12.000	0.000
A_06_P1525	YBR271W	22.000	0.000
A_06_P1526	YBR272C	29.000	0.000
A_06_P1527	YBR273C	125.000	0.000
A_06_P1528	YBR274W	41.000	0.000
A_06_P1529	YBR275C	57.000	0.000
A_06_P1530	YBR276C	170.000	0.000
A_06_P1531	YBR277C	102.000	0.000
A_06_P1532	YBR278W	231.000	0.000
A_06_P1533	YBR279W	720.000	0.000
A_06_P1534	YBR280C	25.000	0.000
A_06_P1535	YBR281C	39.000	0.000
A_06_P1536	YBR282W	142.000	0.000
A_06_P1537	YBR283C	7.000	0.000
A_06_P1538	YBR284W	33.000	0.000
A_06_P1539	YBR285W	106.000	0.000
A_06_P1540	YBR286W	66.000	0.000
A_06_P1541	YBR287W	24.000	0.000
A_06_P1542	YBR288C	105.000	0.000
A_06_P1543	YBR289W	4.000	0.000
A_06_P1544	YBR290W	29.000	0.000
A_06_P1545	YBR291C	107.000	0.000
A_06_P1546	YBR292C	20.000	0.000
A_06_P1547	YBR293W	36.000	0.000
A_06_P1548	YBR294W	4.000	0.000
A_06_P1549	YBR295W	101.000	0.000
A_06_P1550	YBR296C	746.000	0.000
A_06_P1551	YBR297W	276.000	0.000
A_06_P1552	YBR298C	58.000	0.000
A_06_P1553	YBR299W	38.800	0.447

A_06_P1554	YBR300C	52.000	0.000
A_06_P1555	YBL108C-A	0.200	0.447
A_06_P1555	YBR301W	10.400	2.510
A_06_P1555	YGL261C	0.200	0.447
A_06_P1555	YIL176C	1.600	1.140
A_06_P1555	YJL223C	0.200	0.447
A_06_P1556	YBR302C	5.000	1.581
A_06_P1556	YML132W	28.600	2.191
A_06_P1557	YCL001W	451.000	0.000
A_06_P1558	YCL001W-A	3.000	0.000
A_06_P1559	YCL001W-B	62.000	0.000
A_06_P1560	YCL002C	33.000	0.000
A_06_P1561	YCL004W	30.000	0.000
A_06_P1562	YCL005W	41.000	0.000
A_06_P1563	YCL007C	36.000	0.000
A_06_P1564	YCL008C	67.000	0.000
A_06_P1565	YCL009C	11.000	0.000
A_06_P1566	YCL010C	17.000	0.000
A_06_P1567	YCL011C	26.000	0.000
A_06_P1568	YCL014W	29.000	0.000
A_06_P1569	YCL016C	5.000	0.000
A_06_P1570	YCL017C	58.000	0.000
A_06_P1571	YCL018W	43.000	0.000
A_06_P1572	YCL021W-A	79.000	0.000
A_06_P1573	YCL022C	64.000	0.000
A_06_P1574	YCL023C	13.000	0.000
A_06_P1575	YCL024W	16.000	0.000
A_06_P1576	YCL025C	181.000	0.000
A_06_P1577	YCL026C-A	40.000	0.000
A_06_P1578	YCL026C-B	56.000	0.000
A_06_P1579	YCL027W	158.000	0.000
A_06_P1580	YCL028W	27.000	0.000
A_06_P1581	YCL029C	741.000	0.000
A_06_P1582	YCL030C	35.000	0.000
A_06_P1583	YCL031C	170.000	0.000
A_06_P1584	YCL032W	23.000	0.000
A_06_P1585	YCL033C	205.000	0.000
A_06_P1586	YCL034W	97.000	0.000
A_06_P1587	YCL035C	303.000	0.000
A_06_P1588	YCL036W	137.000	0.000
A_06_P1589	YCL037C	33.000	0.000
A_06_P1590	YCL038C	288.000	0.000
A_06_P1591	YCL039W	63.000	0.000
A_06_P1592	YCL040W	12.000	0.000
A_06_P1593	YCL041C	26.000	0.000
A_06_P1594	YCL042W	50.000	0.000
A_06_P1595	YCL043C	124.000	0.000
A_06_P1596	YCL044C	23.000	0.000
A_06_P1597	YCL045C	10.000	0.000
A_06_P1598	YCL046W	688.000	0.000
A_06_P1599	YCL047C	110.000	0.000
A_06_P1600	YCL048W	183.000	0.000
A_06_P1601	YCL049C	32.000	0.000
A_06_P1602	YCL050C	28.000	0.000
A_06_P1603	YCL051W	13.000	0.000

A_06_P1604	YCL052C	365.000	0.000
A_06_P1605	YCL054W	5.000	0.000
A_06_P1606	YCL055W	23.000	0.000
A_06_P1607	YCL056C	36.000	0.000
A_06_P1608	YCL057C-A	85.000	0.000
A_06_P1609	YCL057W	246.000	0.000
A_06_P1610	YCL058C	22.000	0.000
A_06_P1611	YCL059C	16.000	0.000
A_06_P1612	YCL061C	404.000	0.000
A_06_P1613	YCL063W	169.000	0.000
A_06_P1614	YCL064C	22.000	0.000
A_06_P1615	YCL065W	0.600	0.548
A_06_P1616	YCL065W	79.600	5.683
A_06_P1616	YCL066W	198.600	2.302
A_06_P1616	YCR040W	151.600	8.444
A_06_P1616	YCR041W	24.600	1.673
A_06_P1616	YCR097W-A	15.000	3.000
A_06_P1617	YCL067C	84.800	4.266
A_06_P1617	YCR039C	3.600	1.140
A_06_P1617	YCR096C	18.200	2.588
A_06_P1618	YCL068C	16.000	0.000
A_06_P1618	YCR038C	0.200	0.447
A_06_P1619	YCL069W	5.000	0.000
A_06_P1619	YKR105C	0.400	0.548
A_06_P1620	YCL073C	66.800	0.447
A_06_P1621	YCL076W	57.000	0.000
A_06_P1622	YCR001W	18.000	0.000
A_06_P1623	YCR002C	264.000	0.000
A_06_P1624	YCR003W	21.000	0.000
A_06_P1625	YCR004C	38.000	0.000
A_06_P1626	YCR005C	10.000	0.000
A_06_P1627	YCR006C	58.000	0.000
A_06_P1628	YCR007C	29.000	0.000
A_06_P1629	YCR008W	43.000	0.000
A_06_P1630	YCR009C	46.000	0.000
A_06_P1631	YCR010C	53.000	0.000
A_06_P1632	YCR011C	62.000	0.000
A_06_P1633	YCR012W	23.000	0.000
A_06_P1634	YCR013C	7.000	0.000
A_06_P1635	YCR014C	45.000	0.000
A_06_P1636	YCR015C	293.000	0.000
A_06_P1637	YCR016W	317.000	0.000
A_06_P1638	YCR017C	3.000	0.000
A_06_P1639	YCR018C	51.000	0.000
A_06_P1640	YCR018C-A	7.000	3.391
A_06_P1641	YCR019W	325.000	0.000
A_06_P1642	YCR020C	38.000	0.000
A_06_P1643	YCR020C-A	10.000	0.000
A_06_P1644	YCR020W-B	9.000	0.000
A_06_P1645	YCR021C	33.000	0.000
A_06_P1646	YCR022C	14.000	0.000
A_06_P1647	YCR023C	34.000	0.000
A_06_P1648	YCR024C	62.000	0.000
A_06_P1649	YCR024C-A	23.000	0.000
A_06_P1650	YCR025C	45.000	0.000

A_06_P1651	YCR026C	31.000	0.000
A_06_P1652	YCR027C	26.000	0.000
A_06_P1653	YCR028C	42.000	0.000
A_06_P1654	YCR028C-A	36.000	0.000
A_06_P1655	YCR030C	159.000	0.000
A_06_P1656	YCR031C	13.000	0.000
A_06_P1657	YCR032W	104.000	0.000
A_06_P1658	YCR033W	160.000	0.000
A_06_P1659	YCR034W	251.000	0.000
A_06_P1660	YCR035C	125.000	0.000
A_06_P1661	YCR036W	33.000	0.000
A_06_P1662	YCR037C	197.000	0.000
A_06_P1663	YCR038C	234.800	0.447
A_06_P1664	YCR038W-A	21.000	0.000
A_06_P1665	YCL067C	83.400	3.647
A_06_P1665	YCR039C	3.800	1.643
A_06_P1665	YCR096C	16.400	3.578
A_06_P1666	YCL065W	82.800	5.310
A_06_P1666	YCL066W	199.400	2.302
A_06_P1666	YCR040W	147.400	8.444
A_06_P1666	YCR041W	28.000	1.414
A_06_P1666	YCR097W-A	14.600	2.966
A_06_P1667	YCL065W	1.000	0.000
A_06_P1667	YCR041W	0.400	0.548
A_06_P1668	YCR042C	37.000	0.000
A_06_P1669	YCR043C	7.000	0.000
A_06_P1670	YCR044C	240.000	0.000
A_06_P1671	YCR045C	225.000	0.000
A_06_P1672	YCR046C	6.000	0.000
A_06_P1673	YCR047C	8.000	0.000
A_06_P1674	YCR048W	5.000	0.000
A_06_P1675	YCR049C	11.000	0.000
A_06_P1676	YCR050C	21.000	0.000
A_06_P1677	YCR051W	103.000	0.000
A_06_P1678	YCR052W	17.000	0.000
A_06_P1679	YCR053W	11.000	0.000
A_06_P1680	YCR054C	43.000	0.000
A_06_P1681	YCR057C	23.000	0.000
A_06_P1682	YCR059C	18.000	0.000
A_06_P1683	YCR060W	19.000	0.000
A_06_P1684	YCR061W	22.000	0.000
A_06_P1685	YCR063W	5.000	0.000
A_06_P1686	YCR064C	107.000	0.000
A_06_P1687	YCR065W	108.000	0.000
A_06_P1688	YCR066W	455.000	0.000
A_06_P1689	YCR067C	78.000	0.000
A_06_P1690	YCR068W	49.000	0.000
A_06_P1691	YCR069W	72.000	0.000
A_06_P1692	YCR071C	56.000	0.000
A_06_P1693	YCR072C	8.000	0.000
A_06_P1694	YCR073C	150.000	0.000
A_06_P1695	YCR073W-A	24.000	0.000
A_06_P1696	YCR075C	27.000	0.000
A_06_P1697	YCR076C	24.000	0.000
A_06_P1698	YCR077C	28.000	0.000

A_06_P1699	YCR079W	26.000	0.000
A_06_P1700	YCR081W	90.000	0.000
A_06_P1701	YCR082W	35.000	0.000
A_06_P1702	YCR083W	80.000	0.000
A_06_P1703	YCR084C	2.000	0.000
A_06_P1704	YCR085W	18.000	0.000
A_06_P1705	YCR086W	36.000	0.000
A_06_P1706	YCR087C-A	13.000	0.000
A_06_P1707	YCR087W	16.000	0.000
A_06_P1708	YCR088W	4.000	0.000
A_06_P1709	YCR089W	34.000	0.000
A_06_P1710	YCR090C	96.000	0.000
A_06_P1711	YCR091W	88.000	0.000
A_06_P1712	YCR092C	416.000	0.000
A_06_P1713	YCR093W	34.000	0.000
A_06_P1714	YCR094W	61.000	0.000
A_06_P1715	YCR095C	21.000	0.000
A_06_P1716	YCL067C	88.800	7.463
A_06_P1716	YCR039C	3.600	1.817
A_06_P1716	YCR096C	16.400	3.435
A_06_P1717	YCR097W	51.000	0.000
A_06_P1718	YCR097W-A	0.400	0.548
A_06_P1719	YCR098C	40.000	0.000
A_06_P1720	YCR099C	19.000	0.000
A_06_P1721	YCR100C	131.000	0.000
A_06_P1722	YCR101C	294.000	0.000
A_06_P1723	YCR102C	157.000	0.000
A_06_P1724	YCR102W-A	153.000	0.000
A_06_P1725	YCR104W	95.800	0.447
A_06_P1725	YIR041W	0.200	0.447
A_06_P1725	YMR325W	2.000	1.000
A_06_P1726	YCR105W	72.000	0.000
A_06_P1727	YCR106W	18.000	0.000
A_06_P1728	YCR107W	87.000	0.000
A_06_P1729	YDL001W	13.000	0.000
A_06_P1730	YDL002C	160.000	0.000
A_06_P1731	YDL003W	5.000	0.000
A_06_P1732	YDL004W	8.000	0.000
A_06_P1733	YDL005C	72.000	0.000
A_06_P1734	YDL006W	30.000	0.000
A_06_P1735	YDL007W	17.000	0.000
A_06_P1736	YDL008W	119.000	0.000
A_06_P1737	YDL009C	38.000	0.000
A_06_P1738	YDL010W	229.000	0.000
A_06_P1739	YDL011C	29.000	0.000
A_06_P1740	YDL012C	4.000	0.000
A_06_P1741	YDL013W	3.000	0.000
A_06_P1742	YDL014W	9.000	0.000
A_06_P1743	YDL015C	37.000	0.000
A_06_P1744	YDL016C	26.000	0.000
A_06_P1745	YDL017W	75.000	0.000
A_06_P1746	YDL018C	16.000	0.000
A_06_P1747	YDL019C	10.000	0.000
A_06_P1748	YDL020C	269.000	0.000
A_06_P1749	YDL021W	32.000	0.000

A_06_P1750	YDL022W	4.000	0.000
A_06_P1751	YDL023C	53.000	0.000
A_06_P1752	YDL024C	198.000	0.000
A_06_P1753	YDL025C	36.000	0.000
A_06_P1754	YDL026W	7.000	0.000
A_06_P1755	YDL027C	15.000	0.000
A_06_P1756	YDL028C	31.000	0.000
A_06_P1757	YDL029W	59.000	0.000
A_06_P1758	YDL030W	70.000	0.000
A_06_P1759	YDL031W	25.000	0.000
A_06_P1760	YDL032W	90.000	0.000
A_06_P1761	YDL033C	93.000	0.000
A_06_P1762	YDL034W	97.000	0.000
A_06_P1763	YDL035C	90.000	0.000
A_06_P1764	YDL036C	131.000	0.000
A_06_P1765	YDL037C	60.000	0.000
A_06_P1766	YDL038C	17.000	0.000
A_06_P1767	YDL039C	20.000	0.000
A_06_P1768	YDL040C	57.000	0.000
A_06_P1769	YDL041W	173.000	0.000
A_06_P1770	YDL042C	62.000	0.000
A_06_P1771	YDL043C	108.000	0.000
A_06_P1772	YDL044C	34.000	0.000
A_06_P1773	YDL045C	30.000	0.000
A_06_P1774	YDL045W-A	49.000	0.000
A_06_P1775	YDL046W	61.000	0.000
A_06_P1776	YDL047W	250.000	0.000
A_06_P1777	YDL048C	3.000	0.000
A_06_P1778	YDL049C	25.000	0.000
A_06_P1779	YDL050C	101.000	0.000
A_06_P1780	YDL051W	714.000	0.000
A_06_P1781	YDL052C	37.000	0.000
A_06_P1782	YDL053C	20.000	0.000
A_06_P1783	YDL054C	69.000	0.000
A_06_P1784	YDL055C	67.000	0.000
A_06_P1785	YDL056W	215.000	0.000
A_06_P1786	YDL057W	9.000	0.000
A_06_P1787	YDL058W	21.000	0.000
A_06_P1788	YDL059C	222.000	0.000
A_06_P1789	YDL060W	566.000	0.000
A_06_P1790	YDL061C	41.000	0.000
A_06_P1791	YDL062W	5.000	0.000
A_06_P1792	YDL063C	38.000	0.000
A_06_P1793	YDL064W	228.000	0.000
A_06_P1794	YDL065C	61.000	0.000
A_06_P1795	YDL066W	8.000	0.000
A_06_P1796	YDL067C	18.000	0.000
A_06_P1797	YDL068W	53.000	0.000
A_06_P1798	YDL069C	7.000	0.000
A_06_P1799	YDL070W	13.000	0.000
A_06_P1800	YDL071C	135.000	0.000
A_06_P1801	YDL072C	42.000	0.000
A_06_P1802	YDL073W	11.000	0.000
A_06_P1803	YDL074C	149.000	0.000
A_06_P1804	YDL075W	8.000	0.000

A_06_P1805	YDL076C	23.000	0.000
A_06_P1806	YDL077C	54.000	0.000
A_06_P1807	YDL078C	331.000	0.000
A_06_P1808	YDL079C	6.000	0.000
A_06_P1809	YDL080C	5.000	0.000
A_06_P1810	YDL081C	16.000	0.000
A_06_P1811	YDL082W	63.000	0.000
A_06_P1812	YDL083C	33.000	0.000
A_06_P1813	YDL084W	12.000	0.000
A_06_P1814	YDL085C-A	71.000	0.000
A_06_P1815	YDL085W	90.000	0.000
A_06_P1816	YDL086W	21.000	0.000
A_06_P1817	YDL087C	39.000	0.000
A_06_P1818	YDL088C	36.000	0.000
A_06_P1819	YDL089W	24.000	0.000
A_06_P1820	YDL090C	102.000	0.000
A_06_P1821	YDL091C	26.000	0.000
A_06_P1822	YDL092W	15.000	0.000
A_06_P1823	YDL093W	228.000	0.000
A_06_P1824	YDL094C	54.000	0.000
A_06_P1825	YDL095W	103.000	0.000
A_06_P1826	YDL096C	101.000	0.000
A_06_P1827	YDL097C	47.000	0.000
A_06_P1828	YDL098C	12.000	0.000
A_06_P1829	YDL099W	74.000	0.000
A_06_P1830	YDL100C	12.000	0.000
A_06_P1831	YDL101C	21.000	0.000
A_06_P1832	YDL102W	15.000	0.000
A_06_P1833	YDL103C	85.000	0.000
A_06_P1834	YDL104C	37.000	0.000
A_06_P1835	YDL105W	740.000	0.000
A_06_P1836	YDL106C	10.000	0.000
A_06_P1837	YDL107W	10.000	0.000
A_06_P1838	YDL108W	178.000	0.000
A_06_P1839	YDL109C	180.000	0.000
A_06_P1840	YDL110C	7.000	0.000
A_06_P1841	YDL111C	16.000	0.000
A_06_P1842	YDL112W	33.000	0.000
A_06_P1843	YDL113C	9.000	0.000
A_06_P1844	YDL114W	7.000	0.000
A_06_P1845	YDL114W-A	93.000	0.000
A_06_P1846	YDL115C	28.000	0.000
A_06_P1847	YDL116W	17.000	0.000
A_06_P1848	YDL117W	130.000	0.000
A_06_P1849	YDL118W	45.000	0.000
A_06_P1850	YDL119C	29.000	0.000
A_06_P1851	YDL120W	17.000	0.000
A_06_P1852	YDL121C	31.000	0.000
A_06_P1853	YDL122W	29.000	0.000
A_06_P1854	YDL123W	188.000	0.000
A_06_P1855	YDL124W	48.000	0.000
A_06_P1856	YDL125C	90.000	0.000
A_06_P1857	YDL126C	16.000	0.000
A_06_P1858	YDL127W	69.000	0.000
A_06_P1859	YDL128W	60.000	0.000

A_06_P1860	YDL129W	6.000	0.000
A_06_P1861	YDL130W	41.000	0.000
A_06_P1862	YDL130W-A	83.000	0.000
A_06_P1863	YDL131W	53.000	0.000
A_06_P1864	YDL132W	16.000	0.000
A_06_P1865	YDL133C-A	241.400	3.435
A_06_P1865	YDL184C	1.600	2.074
A_06_P1866	YDL133W	159.000	0.000
A_06_P1867	YDL134C	41.000	0.000
A_06_P1868	YDL135C	33.000	0.000
A_06_P1869	YDL136W	75.400	2.702
A_06_P1869	YDL191W	1.600	1.673
A_06_P1870	YDL137W	114.000	0.000
A_06_P1871	YDL138W	356.000	0.000
A_06_P1872	YDL139C	24.000	0.000
A_06_P1873	YDL140C	32.000	0.000
A_06_P1874	YDL141W	10.000	0.000
A_06_P1875	YDL142C	34.000	0.000
A_06_P1876	YDL143W	11.000	0.000
A_06_P1877	YDL144C	20.000	0.000
A_06_P1878	YDL145C	354.000	0.000
A_06_P1879	YDL146W	59.000	0.000
A_06_P1880	YDL147W	4.000	0.000
A_06_P1881	YDL148C	9.000	0.000
A_06_P1882	YDL149W	63.000	0.000
A_06_P1883	YDL150W	25.000	0.000
A_06_P1884	YDL151C	6.000	0.000
A_06_P1885	YDL152W	59.000	0.000
A_06_P1886	YDL153C	33.000	0.000
A_06_P1887	YDL154W	73.000	0.000
A_06_P1888	YDL155W	25.000	0.000
A_06_P1889	YDL156W	15.000	0.000
A_06_P1890	YDL157C	114.000	0.000
A_06_P1891	YDL158C	41.000	0.000
A_06_P1892	YDL159W	19.000	0.000
A_06_P1893	YDL159W-A	20.000	0.000
A_06_P1894	YDL160C	523.000	0.000
A_06_P1895	YDL161W	2.000	0.000
A_06_P1896	YDL162C	247.000	0.000
A_06_P1897	YDL163W	259.000	0.000
A_06_P1898	YDL164C	140.000	0.000
A_06_P1899	YDL165W	19.000	0.000
A_06_P1900	YDL166C	21.000	0.000
A_06_P1901	YDL167C	7.000	0.000
A_06_P1902	YDL168W	153.000	0.000
A_06_P1903	YDL169C	59.000	0.000
A_06_P1904	YDL170W	291.000	0.000
A_06_P1905	YDL171C	154.000	0.000
A_06_P1906	YDL172C	45.000	0.000
A_06_P1907	YDL173W	119.000	0.000
A_06_P1908	YDL174C	38.000	0.000
A_06_P1909	YDL175C	14.000	0.000
A_06_P1910	YDL176W	88.000	0.000
A_06_P1911	YDL177C	324.000	0.000
A_06_P1912	YDL178W	81.000	0.000

A_06_P1913	YDL179W	49.000	0.000
A_06_P1914	YDL180W	48.000	0.000
A_06_P1915	YDL181W	260.000	0.000
A_06_P1916	YDL182W	19.000	0.000
A_06_P1917	YDL183C	264.000	0.000
A_06_P1918	YDL133C-A	15.600	3.435
A_06_P1918	YDL184C	108.400	2.074
A_06_P1919	YDL185C-A	11.000	0.000
A_06_P1920	YDL185W	23.000	0.000
A_06_P1921	YDL186W	7.000	0.000
A_06_P1922	YDL187C	9.000	0.000
A_06_P1923	YDL188C	44.000	0.000
A_06_P1924	YDL189W	371.000	0.000
A_06_P1925	YDL190C	78.000	0.000
A_06_P1926	YDL136W	3.600	2.702
A_06_P1926	YDL191W	56.400	1.673
A_06_P1927	YDL192W	409.000	0.000
A_06_P1928	YDL193W	89.000	0.000
A_06_P1929	YDL194W	13.000	0.000
A_06_P1930	YDL195W	71.000	0.000
A_06_P1931	YDL196W	61.000	0.000
A_06_P1932	YDL197C	45.000	0.000
A_06_P1933	YDL198C	33.000	0.000
A_06_P1934	YDL199C	41.000	0.000
A_06_P1935	YDL200C	44.000	0.000
A_06_P1936	YDL201W	19.000	0.000
A_06_P1937	YDL202W	51.000	0.000
A_06_P1938	YDL203C	6.000	0.000
A_06_P1939	YDL204W	580.000	0.000
A_06_P1940	YDL205C	222.000	0.000
A_06_P1941	YDL206W	78.000	0.000
A_06_P1942	YDL207W	12.000	0.000
A_06_P1943	YDL208W	151.000	0.000
A_06_P1944	YDL209C	53.000	0.000
A_06_P1945	YDL210W	738.000	0.000
A_06_P1946	YDL211C	23.000	0.000
A_06_P1947	YDL212W	317.000	0.000
A_06_P1948	YDL213C	237.000	0.000
A_06_P1949	YDL214C	50.000	0.000
A_06_P1950	YDL215C	21.000	0.000
A_06_P1951	YDL216C	45.000	0.000
A_06_P1952	YDL217C	13.000	0.000
A_06_P1953	YDL218W	12.000	0.000
A_06_P1954	YDL219W	128.000	0.000
A_06_P1955	YDL220C	842.000	0.000
A_06_P1956	YDL221W	7.000	0.000
A_06_P1957	YDL222C	74.000	0.000
A_06_P1958	YDL223C	336.000	0.000
A_06_P1959	YDL224C	27.000	0.000
A_06_P1960	YDL225W	8.000	0.000
A_06_P1961	YDL226C	82.000	0.000
A_06_P1962	YDL227C	4.000	0.000
A_06_P1963	YDL228C	24.000	0.000
A_06_P1964	YDL229W	139.000	0.000
A_06_P1965	YDL230W	51.000	0.000

A_06_P1966	YDL231C	61.000	0.000
A_06_P1967	YDL232W	16.000	0.000
A_06_P1968	YDL233W	15.000	0.000
A_06_P1969	YDL234C	121.000	0.000
A_06_P1970	YDL235C	91.000	0.000
A_06_P1971	YDL236W	30.000	0.000
A_06_P1972	YDL237W	97.000	0.000
A_06_P1973	YDL238C	38.000	0.000
A_06_P1974	YDL239C	332.000	0.000
A_06_P1975	YDL240C-A	277.000	0.000
A_06_P1976	YDL240W	250.000	0.000
A_06_P1977	YDL241W	55.000	0.000
A_06_P1978	YDL242W	31.000	0.000
A_06_P1979	YDL243C	6.000	0.000
A_06_P1980	YDL244W	40.000	0.000
A_06_P1981	YDL245C	23.000	0.707
A_06_P1981	YJR158W	2.600	0.894
A_06_P1982	YDL246C	85.600	1.517
A_06_P1982	YJR159W	9.000	2.646
A_06_P1983	YDL247W	12.800	2.490
A_06_P1984	YDL247W-A	166.000	0.000
A_06_P1985	YDL248W	41.800	2.168
A_06_P1986	YDR001C	13.000	0.000
A_06_P1987	YDR002W	212.000	0.000
A_06_P1988	YDR003W	3.000	0.000
A_06_P1989	YDR004W	8.000	0.000
A_06_P1990	YDR005C	1000.000	0.000
A_06_P1991	YDR006C	121.000	0.000
A_06_P1992	YDR007W	105.000	0.000
A_06_P1993	YDR008C	57.000	0.000
A_06_P1994	YDR009W	53.000	0.000
A_06_P1995	YDR010C	29.000	0.000
A_06_P1996	YDR011W	27.000	0.000
A_06_P1997	YDR012W	10.000	0.000
A_06_P1998	YDR013W	797.000	0.000
A_06_P1999	YDR014W	16.000	0.000
A_06_P2000	YDR015C	24.000	0.000
A_06_P2001	YDR016C	28.000	0.000
A_06_P2002	YDR017C	63.000	0.000
A_06_P2003	YDR018C	68.000	0.000
A_06_P2004	YDR019C	31.000	0.000
A_06_P2005	YDR020C	102.000	0.000
A_06_P2006	YDR021W	3.000	0.000
A_06_P2007	YDR022C	23.000	0.000
A_06_P2008	YDR023W	64.000	0.000
A_06_P2009	YDR024W	26.000	0.000
A_06_P2010	YDR025W	45.000	0.000
A_06_P2011	YDR026C	6.000	0.000
A_06_P2012	YDR027C	18.000	0.000
A_06_P2013	YDR028C	29.000	0.000
A_06_P2014	YDR029W	17.000	0.000
A_06_P2015	YDR030C	26.000	0.000
A_06_P2016	YDR031W	69.000	0.000
A_06_P2017	YDR032C	50.000	0.000
A_06_P2018	YDR033W	18.000	0.000

A_06_P2019	YDR034C	11.000	0.000
A_06_P2020	YDR034C-A	35.000	0.000
A_06_P2021	YDR034W-B	97.000	0.000
A_06_P2022	YDR035W	147.000	0.000
A_06_P2023	YDR036C	52.000	0.000
A_06_P2024	YDR037W	12.000	0.000
A_06_P2025	YDR038C	30.000	1.000
A_06_P2026	YDR039C	0.800	1.095
A_06_P2027	YDR038C	1.000	1.000
A_06_P2027	YDR039C	19.200	1.095
A_06_P2027	YDR040C	111.000	0.000
A_06_P2028	YDR041W	261.000	0.000
A_06_P2029	YDR042C	62.000	0.000
A_06_P2030	YDR043C	19.000	0.000
A_06_P2031	YDR044W	53.000	0.000
A_06_P2032	YDR045C	56.000	0.000
A_06_P2033	YDR046C	39.000	0.000
A_06_P2034	YDR047W	22.000	0.000
A_06_P2035	YDR048C	24.000	0.000
A_06_P2036	YDR049W	26.000	0.000
A_06_P2037	YDR050C	12.000	0.000
A_06_P2038	YDR051C	21.000	0.000
A_06_P2039	YDR052C	171.000	0.000
A_06_P2040	YDR053W	35.000	0.000
A_06_P2041	YDR054C	90.000	0.000
A_06_P2042	YDR055W	8.000	0.000
A_06_P2043	YDR056C	58.000	0.000
A_06_P2044	YDR057W	15.000	0.000
A_06_P2045	YDR058C	15.000	0.000
A_06_P2046	YDR059C	99.000	0.000
A_06_P2047	YDR060W	165.000	0.000
A_06_P2048	YDR061W	20.000	0.000
A_06_P2049	YDR062W	15.000	0.000
A_06_P2050	YDR063W	37.000	0.000
A_06_P2051	YDR064W	123.000	0.000
A_06_P2052	YDR065W	261.000	0.000
A_06_P2053	YDR066C	137.000	0.000
A_06_P2054	YDR067C	95.000	0.000
A_06_P2055	YDR068W	29.000	0.000
A_06_P2056	YDR069C	13.000	0.000
A_06_P2057	YDR070C	56.000	0.000
A_06_P2058	YDR071C	33.000	0.000
A_06_P2059	YDR072C	26.000	0.000
A_06_P2060	YDR073W	3.000	0.000
A_06_P2061	YDR074W	203.000	0.000
A_06_P2062	YDR075W	55.000	0.000
A_06_P2063	YDR076W	92.000	0.000
A_06_P2064	YDR077W	31.000	0.000
A_06_P2065	YDR078C	7.000	0.000
A_06_P2066	YDR079C-A	87.000	0.000
A_06_P2067	YDR079W	109.000	0.000
A_06_P2068	YDR080W	607.000	0.000
A_06_P2069	YDR081C	7.000	0.000
A_06_P2070	YDR082W	378.000	0.000
A_06_P2071	YDR083W	57.000	0.000

A_06_P2072	YDR084C	39.000	0.000
A_06_P2073	YDR085C	342.000	0.000
A_06_P2074	YDR086C	177.000	0.000
A_06_P2075	YDR087C	38.000	0.000
A_06_P2076	YDR088C	226.000	0.000
A_06_P2077	YDR089W	61.000	0.000
A_06_P2078	YDR090C	36.000	0.000
A_06_P2079	YDR091C	24.000	0.000
A_06_P2080	YDR092W	402.000	0.000
A_06_P2082	YDR093W	34.000	0.000
A_06_P2082	YDR094W	16.000	0.000
A_06_P2082	YMR062C	0.200	0.447
A_06_P2083	YDR095C	5.000	0.000
A_06_P2084	YDR096W	36.000	0.000
A_06_P2085	YDR097C	60.000	0.000
A_06_P2086	YDR098C	144.000	0.000
A_06_P2087	YDR099W	24.000	0.000
A_06_P2088	YDR100W	85.000	0.000
A_06_P2089	YDR101C	121.000	0.000
A_06_P2090	YDR102C	218.000	0.000
A_06_P2091	YDR103W	11.000	0.000
A_06_P2092	YDR104C	1.000	0.000
A_06_P2093	YDR105C	39.000	0.000
A_06_P2094	YDR106W	287.000	0.000
A_06_P2095	YDR107C	41.000	0.000
A_06_P2096	YDR108W	19.000	0.000
A_06_P2097	YDR109C	85.000	0.000
A_06_P2098	YDR110W	192.000	0.000
A_06_P2099	YDR111C	66.000	0.000
A_06_P2100	YDR112W	203.000	0.000
A_06_P2101	YDR113C	14.000	0.000
A_06_P2102	YDR114C	51.000	0.000
A_06_P2103	YDR115W	11.000	0.000
A_06_P2104	YDR116C	47.000	0.000
A_06_P2105	YDR117C	23.000	0.000
A_06_P2106	YDR118W	20.000	0.000
A_06_P2107	YDR119W	17.000	0.000
A_06_P2108	YDR120C	50.000	0.000
A_06_P2109	YDR121W	21.000	0.000
A_06_P2110	YDR122W	23.000	0.000
A_06_P2111	YDR123C	3.000	0.000
A_06_P2112	YDR124W	72.000	0.000
A_06_P2113	YDR125C	39.000	0.000
A_06_P2114	YDR126W	5.000	0.000
A_06_P2115	YDR127W	7.000	0.000
A_06_P2116	YDR128W	179.000	0.000
A_06_P2117	YDR129C	140.000	0.000
A_06_P2118	YDR130C	28.000	0.000
A_06_P2119	YDR131C	34.000	0.000
A_06_P2120	YDR132C	13.000	0.000
A_06_P2121	YDR133C	17.000	0.000
A_06_P2122	YDR134C	17.000	0.000
A_06_P2123	YDR135C	6.000	0.000
A_06_P2124	YDR136C	37.000	0.000
A_06_P2125	YDR137W	11.000	0.000

A_06_P2126	YDR138W	34.000	0.000
A_06_P2127	YDR139C	331.000	0.000
A_06_P2128	YDR140W	37.000	0.000
A_06_P2129	YDR141C	6.000	0.000
A_06_P2130	YDR142C	35.000	0.000
A_06_P2131	YDR143C	20.000	0.000
A_06_P2132	YDR144C	87.000	0.000
A_06_P2133	YDR145W	58.000	0.000
A_06_P2134	YDR146C	27.000	0.000
A_06_P2135	YDR147W	96.000	0.000
A_06_P2136	YDR148C	45.000	0.000
A_06_P2137	YDR149C	138.000	0.000
A_06_P2138	YDR150W	56.000	0.000
A_06_P2139	YDR151C	135.000	0.000
A_06_P2140	YDR152W	198.000	0.000
A_06_P2141	YDR153C	52.000	0.000
A_06_P2142	YDR154C	45.000	0.000
A_06_P2143	YDR155C	15.000	0.000
A_06_P2144	YDR156W	32.000	0.000
A_06_P2145	YDR157W	3.000	0.000
A_06_P2146	YDR158W	27.000	0.000
A_06_P2147	YDR159W	43.000	0.000
A_06_P2148	YDR160W	47.000	0.000
A_06_P2149	YDR161W	51.000	0.000
A_06_P2150	YDR162C	41.000	0.000
A_06_P2151	YDR163W	10.000	0.000
A_06_P2152	YDR164C	45.000	0.000
A_06_P2153	YDR165W	27.000	0.000
A_06_P2154	YDR166C	74.000	0.000
A_06_P2155	YDR167W	51.000	0.000
A_06_P2156	YDR168W	50.000	0.000
A_06_P2157	YDR169C	42.000	0.000
A_06_P2158	YDR170C	61.000	0.000
A_06_P2159	YDR171W	364.000	0.000
A_06_P2160	YDR172W	25.000	0.000
A_06_P2161	YDR173C	137.000	0.000
A_06_P2162	YDR174W	72.000	0.000
A_06_P2163	YDR175C	192.000	0.000
A_06_P2164	YDR176W	51.000	0.000
A_06_P2165	YDR177W	51.000	0.000
A_06_P2166	YDR178W	23.000	0.000
A_06_P2167	YDR179C	97.000	0.000
A_06_P2168	YDR179W-A	81.000	0.000
A_06_P2169	YDR180W	2.000	0.000
A_06_P2170	YDR181C	9.000	0.000
A_06_P2171	YDR182W	35.000	0.000
A_06_P2171	YJL181W	0.200	0.447
A_06_P2172	YDR183W	247.000	0.000
A_06_P2173	YDR184C	20.000	0.000
A_06_P2174	YDR185C	25.000	0.000
A_06_P2175	YDR186C	121.000	0.000
A_06_P2176	YDR187C	76.000	0.000
A_06_P2177	YDR188W	493.000	0.000
A_06_P2178	YDR189W	76.000	0.000
A_06_P2179	YDR190C	11.000	0.000

A_06_P2180	YDR191W	223.000	0.000
A_06_P2181	YDR192C	79.000	0.000
A_06_P2182	YDR193W	1.000	0.000
A_06_P2183	YDR194C	123.000	0.000
A_06_P2184	YDR195W	95.000	0.000
A_06_P2185	YDR196C	56.000	0.000
A_06_P2186	YDR197W	18.000	0.000
A_06_P2187	YDR198C	120.000	0.000
A_06_P2188	YDR199W	14.000	0.000
A_06_P2189	YDR200C	36.000	0.000
A_06_P2190	YDR201W	227.000	0.000
A_06_P2191	YDR202C	214.000	0.000
A_06_P2192	YDR203W	3.000	0.000
A_06_P2193	YDR204W	259.000	0.000
A_06_P2194	YDR205W	23.000	0.000
A_06_P2195	YDR206W	277.000	0.000
A_06_P2196	YDR207C	9.000	0.000
A_06_P2197	YDR208W	11.000	0.000
A_06_P2198	YDR209C	38.000	0.000
A_06_P2199	YDR210W	20.000	0.000
A_06_P2200	YDR211W	139.000	0.000
A_06_P2201	YDR212W	5.000	0.000
A_06_P2202	YDR213W	17.000	0.000
A_06_P2203	YDR214W	94.000	0.000
A_06_P2204	YDR215C	14.000	0.000
A_06_P2205	YDR216W	79.000	0.000
A_06_P2206	YDR217C	184.000	0.000
A_06_P2207	YDR218C	41.000	0.000
A_06_P2208	YDR219C	16.000	0.000
A_06_P2209	YDR220C	147.000	0.000
A_06_P2210	YDR221W	10.000	0.000
A_06_P2211	YDR222W	110.000	0.000
A_06_P2212	YDR223W	335.000	0.000
A_06_P2213	YDR224C	80.000	0.000
A_06_P2214	YDR225W	23.000	0.000
A_06_P2215	YDR226W	651.000	0.000
A_06_P2216	YDR227W	11.000	0.000
A_06_P2217	YDR228C	61.000	0.000
A_06_P2218	YDR229W	17.000	0.000
A_06_P2219	YDR230W	44.000	0.000
A_06_P2220	YDR231C	69.000	0.000
A_06_P2221	YDR232W	138.000	0.000
A_06_P2222	YDR233C	70.000	0.000
A_06_P2223	YDR234W	8.000	0.000
A_06_P2224	YDR235W	30.000	0.000
A_06_P2225	YDR236C	8.000	0.000
A_06_P2226	YDR237W	20.000	0.000
A_06_P2227	YDR238C	54.000	0.000
A_06_P2228	YDR239C	27.000	0.000
A_06_P2229	YDR240C	28.000	0.000
A_06_P2230	YDR241W	91.000	0.000
A_06_P2231	YDR242W	143.000	0.000
A_06_P2232	YDR243C	63.000	0.000
A_06_P2233	YDR244W	10.000	0.000
A_06_P2234	YDR245W	14.000	0.000

A_06_P2235	YDR246W	98.000	0.000
A_06_P2236	YDR247W	94.000	0.000
A_06_P2237	YDR248C	21.000	0.000
A_06_P2238	YDR249C	18.000	0.000
A_06_P2239	YDR250C	91.000	0.000
A_06_P2240	YDR251W	44.000	0.000
A_06_P2241	YDR252W	295.000	0.000
A_06_P2242	YDR253C	30.000	0.000
A_06_P2243	YDR254W	35.000	0.000
A_06_P2244	YDR255C	319.000	0.000
A_06_P2245	YDR256C	105.000	0.000
A_06_P2246	YDR257C	2.000	0.000
A_06_P2247	YDR258C	103.000	0.000
A_06_P2248	YDR259C	31.000	0.000
A_06_P2249	YDR260C	8.000	0.000
A_06_P2250	YDR261C	107.000	0.000
A_06_P2251	YDR262W	22.000	0.000
A_06_P2252	YDR263C	5.000	0.000
A_06_P2253	YDR264C	21.000	0.000
A_06_P2254	YDR265W	26.000	0.000
A_06_P2255	YDR266C	63.000	0.000
A_06_P2256	YDR267C	19.000	0.000
A_06_P2257	YDR268W	16.000	0.000
A_06_P2258	YDR269C	51.000	0.000
A_06_P2259	YDR270W	237.000	0.000
A_06_P2260	YDR271C	175.000	0.000
A_06_P2261	YDR272W	39.000	0.000
A_06_P2262	YDR273W	66.000	0.000
A_06_P2263	YDR274C	135.000	0.000
A_06_P2264	YDR275W	20.000	0.000
A_06_P2265	YDR276C	35.000	0.000
A_06_P2266	YDR277C	221.000	0.000
A_06_P2267	YDR278C	83.000	0.000
A_06_P2268	YDR279W	24.000	0.000
A_06_P2269	YDR280W	28.000	0.000
A_06_P2270	YDR281C	164.000	0.000
A_06_P2271	YDR282C	427.000	0.000
A_06_P2272	YDR283C	60.000	0.000
A_06_P2273	YDR284C	85.000	0.000
A_06_P2274	YDR285W	80.000	0.000
A_06_P2275	YDR286C	11.000	0.000
A_06_P2276	YDR287W	78.000	0.000
A_06_P2277	YDR288W	20.000	0.000
A_06_P2278	YDR289C	4.000	0.000
A_06_P2279	YDR290W	173.000	0.000
A_06_P2280	YDR291W	24.000	0.000
A_06_P2281	YDR292C	30.000	0.000
A_06_P2282	YDR293C	237.000	0.000
A_06_P2283	YDR294C	49.000	0.000
A_06_P2284	YDR295C	32.000	0.000
A_06_P2285	YDR296W	26.000	0.000
A_06_P2286	YDR297W	55.000	0.000
A_06_P2287	YDR298C	44.000	0.000
A_06_P2288	YDR299W	39.000	0.000
A_06_P2289	YDR300C	186.000	0.000

A_06_P2290	YDR301W	15.000	0.000
A_06_P2291	YDR302W	5.000	0.000
A_06_P2292	YDR303C	254.000	0.000
A_06_P2293	YDR304C	104.000	0.000
A_06_P2294	YDR305C	5.000	0.000
A_06_P2295	YDR306C	104.000	0.000
A_06_P2296	YDR307W	17.000	0.000
A_06_P2297	YDR308C	63.000	0.000
A_06_P2298	YDR309C	76.000	0.000
A_06_P2299	YDR310C	13.000	0.000
A_06_P2300	YDR311W	24.000	0.000
A_06_P2301	YDR312W	10.000	0.000
A_06_P2302	YDR313C	18.000	0.000
A_06_P2303	YDR314C	28.000	0.000
A_06_P2304	YDR315C	10.000	0.000
A_06_P2305	YDR316W	28.000	0.000
A_06_P2306	YDR317W	67.000	0.000
A_06_P2307	YDR318W	110.000	0.000
A_06_P2308	YDR319C	59.000	0.000
A_06_P2309	YDR320C	6.000	0.000
A_06_P2310	YDR320C-A	20.000	0.000
A_06_P2311	YDR321W	1.000	0.000
A_06_P2312	YDR322C-A	6.000	0.000
A_06_P2313	YDR322W	87.000	0.000
A_06_P2314	YDR323C	22.000	0.000
A_06_P2315	YDR324C	51.000	0.000
A_06_P2316	YDR325W	473.000	0.000
A_06_P2317	YDR326C	2.000	0.000
A_06_P2318	YDR327W	418.000	0.000
A_06_P2319	YDR328C	20.000	0.000
A_06_P2320	YDR329C	15.000	0.000
A_06_P2321	YDR330W	638.000	0.000
A_06_P2322	YDR331W	190.000	0.000
A_06_P2323	YDR332W	567.000	0.000
A_06_P2324	YDR333C	37.000	0.000
A_06_P2325	YDR334W	67.000	0.000
A_06_P2326	YDR335W	124.000	0.000
A_06_P2327	YDR336W	113.000	0.000
A_06_P2328	YDR337W	19.000	0.000
A_06_P2329	YDR338C	40.000	0.000
A_06_P2330	YDR339C	51.000	0.000
A_06_P2331	YDR340W	3.000	0.000
A_06_P2332	YDR341C	14.000	0.000
A_06_P2333	YDR342C	27.800	0.447
A_06_P2333	YDR343C	0.800	0.447
A_06_P2334	YDR342C	0.200	0.447
A_06_P2334	YDR343C	149.200	0.447
A_06_P2335	YDR344C	93.000	0.000
A_06_P2336	YDR345C	10.000	0.000
A_06_P2337	YDR346C	13.000	0.000
A_06_P2338	YDR347W	23.000	0.000
A_06_P2339	YDR348C	21.000	0.000
A_06_P2340	YDR349C	46.000	0.000
A_06_P2341	YDR350C	58.000	0.000
A_06_P2342	YDR351W	243.000	0.000

A_06_P2343	YDR352W	212.000	0.000
A_06_P2344	YDR353W	169.000	0.000
A_06_P2345	YDR354W	102.000	0.000
A_06_P2346	YDR355C	9.000	0.000
A_06_P2347	YDR356W	47.000	0.000
A_06_P2348	YDR357C	95.000	0.000
A_06_P2349	YDR358W	146.000	0.000
A_06_P2350	YDR359C	163.000	0.000
A_06_P2351	YDR360W	74.000	0.000
A_06_P2352	YDR361C	21.000	0.000
A_06_P2353	YDR362C	26.000	0.000
A_06_P2354	YDR363W	299.000	0.000
A_06_P2355	YDR363W-A	45.000	0.000
A_06_P2356	YDR364C	90.000	0.000
A_06_P2357	YDR365C	31.000	0.000
A_06_P2358	YDR366C	24.000	0.000
A_06_P2359	YDR367W	111.000	0.000
A_06_P2360	YDR368W	116.000	0.000
A_06_P2361	YDR369C	70.000	0.000
A_06_P2362	YDR370C	53.000	0.000
A_06_P2363	YDR371W	83.000	0.000
A_06_P2364	YDR372C	3.000	0.000
A_06_P2365	YDR373W	30.000	0.000
A_06_P2366	YDR374C	69.000	0.000
A_06_P2367	YDR375C	11.000	0.000
A_06_P2368	YDR376W	99.000	0.000
A_06_P2369	YDR377W	111.000	0.000
A_06_P2370	YDR378C	6.000	0.000
A_06_P2371	YDR379C-A	5.000	0.000
A_06_P2372	YDR379W	31.000	0.000
A_06_P2373	YDR380W	24.000	0.000
A_06_P2374	YDR381W	235.000	0.000
A_06_P2375	YDR382W	125.000	0.000
A_06_P2376	YDR383C	112.000	0.000
A_06_P2377	YDR384C	106.000	0.000
A_06_P2378	YDR385W	20.800	3.033
A_06_P2379	YDR386W	141.000	0.000
A_06_P2380	YDR387C	63.000	0.000
A_06_P2381	YDR388W	75.000	0.000
A_06_P2382	YDR389W	21.000	0.000
A_06_P2383	YDR390C	793.000	0.000
A_06_P2384	YDR391C	90.000	0.000
A_06_P2385	YDR392W	23.000	0.000
A_06_P2386	YDR393W	41.000	0.000
A_06_P2387	YDR394W	62.000	0.000
A_06_P2388	YDR395W	113.000	0.000
A_06_P2389	YDR396W	131.000	0.000
A_06_P2390	YDR397C	31.000	0.000
A_06_P2391	YDR398W	90.000	0.000
A_06_P2392	YDR399W	121.000	0.000
A_06_P2393	YDR400W	288.000	0.000
A_06_P2394	YDR401W	24.000	0.000
A_06_P2395	YDR402C	50.000	0.000
A_06_P2396	YDR403W	206.000	0.000
A_06_P2397	YDR404C	82.000	0.000

A_06_P2398	YDR405W	40.000	0.000
A_06_P2399	YDR406W	8.000	0.000
A_06_P2400	YDR407C	138.000	0.000
A_06_P2401	YDR408C	23.000	0.000
A_06_P2402	YDR409W	86.000	0.000
A_06_P2403	YDR410C	28.000	0.000
A_06_P2404	YDR411C	357.000	0.000
A_06_P2405	YDR412W	70.000	0.000
A_06_P2406	YDR413C	56.000	0.000
A_06_P2407	YDR414C	35.000	0.000
A_06_P2408	YDR415C	68.000	0.000
A_06_P2409	YDR416W	102.000	0.000
A_06_P2410	YDR417C	60.000	0.000
A_06_P2411	YDR418W	76.000	0.000
A_06_P2412	YDR419W	109.000	0.000
A_06_P2413	YDR420W	27.000	0.000
A_06_P2414	YDR421W	24.000	0.000
A_06_P2415	YDR422C	59.000	0.000
A_06_P2416	YDR423C	5.000	0.000
A_06_P2417	YDR424C	88.000	0.000
A_06_P2418	YDR425W	131.000	0.000
A_06_P2419	YDR426C	27.000	0.000
A_06_P2420	YDR427W	38.000	0.000
A_06_P2421	YDR428C	10.000	0.000
A_06_P2422	YDR429C	167.000	0.000
A_06_P2423	YDR430C	48.000	0.000
A_06_P2424	YDR431W	29.000	0.000
A_06_P2425	YDR432W	49.400	2.608
A_06_P2426	YDR432W	2.600	2.608
A_06_P2426	YDR433W	44.000	0.000
A_06_P2427	YDR434W	96.000	0.000
A_06_P2428	YDR435C	32.000	0.000
A_06_P2429	YDR436W	19.000	0.000
A_06_P2430	YDR437W	23.000	0.000
A_06_P2431	YDR438W	103.000	0.000
A_06_P2432	YDR439W	10.000	0.000
A_06_P2433	YDR440W	91.000	0.000
A_06_P2434	YDR441C	61.000	0.000
A_06_P2435	YDR442W	8.000	0.000
A_06_P2436	YDR443C	17.000	0.000
A_06_P2437	YDR444W	35.000	0.000
A_06_P2438	YDR445C	25.000	0.000
A_06_P2439	YDR446W	25.000	0.000
A_06_P2440	YDR447C	114.000	0.000
A_06_P2441	YDR448W	45.000	0.000
A_06_P2442	YDR449C	64.000	0.000
A_06_P2443	YDR450W	10.000	0.000
A_06_P2444	YDR451C	121.000	0.000
A_06_P2445	YDR452W	12.000	0.000
A_06_P2446	YDR453C	38.000	0.000
A_06_P2447	YDR454C	13.000	0.000
A_06_P2448	YDR455C	9.000	0.000
A_06_P2449	YDR456W	9.000	0.000
A_06_P2450	YDR457W	79.000	0.000
A_06_P2451	YDR458C	23.000	0.000

A_06_P2452	YDR459C	9.000	0.000
A_06_P2453	YDR460W	28.000	0.000
A_06_P2454	YDR461W	288.000	0.000
A_06_P2455	YDR462W	488.000	0.000
A_06_P2456	YDR463W	181.000	0.000
A_06_P2457	YDR464W	118.000	0.000
A_06_P2458	YDR465C	85.000	0.000
A_06_P2459	YDR466W	11.000	0.000
A_06_P2460	YDR467C	88.000	0.000
A_06_P2461	YDR468C	22.000	0.000
A_06_P2462	YDR469W	28.000	0.000
A_06_P2463	YDR470C	49.000	0.000
A_06_P2464	YDR471W	97.000	0.000
A_06_P2465	YDR472W	196.000	0.000
A_06_P2466	YDR473C	36.000	0.000
A_06_P2467	YDR475C	19.600	1.140
A_06_P2468	YDR475C	85.400	1.140
A_06_P2469	YDR476C	63.000	0.000
A_06_P2470	YDR477W	21.000	0.000
A_06_P2471	YDR478W	192.000	0.000
A_06_P2472	YDR479C	17.000	0.000
A_06_P2473	YDR480W	20.000	0.000
A_06_P2474	YDR481C	137.000	0.000
A_06_P2475	YDR482C	47.000	0.000
A_06_P2476	YDR483W	152.000	0.000
A_06_P2477	YDR484W	20.000	0.000
A_06_P2478	YDR485C	9.000	0.000
A_06_P2479	YDR486C	14.000	0.000
A_06_P2480	YDR487C	27.000	0.000
A_06_P2481	YDR488C	26.000	0.000
A_06_P2482	YDR489W	182.000	0.000
A_06_P2483	YDR490C	63.000	0.000
A_06_P2484	YDR491C	500.000	0.000
A_06_P2485	YDR492W	22.000	0.000
A_06_P2486	YDR493W	375.000	0.000
A_06_P2487	YDR494W	62.000	0.000
A_06_P2488	YDR495C	21.000	0.000
A_06_P2489	YDR496C	14.000	0.000
A_06_P2490	YDR497C	342.000	0.000
A_06_P2491	YDR498C	31.000	0.000
A_06_P2492	YDR499W	33.000	0.000
A_06_P2493	YDR500C	27.000	0.000
A_06_P2494	YDR501W	35.000	0.000
A_06_P2495	YDR502C	18.000	0.000
A_06_P2496	YDR503C	122.000	0.000
A_06_P2497	YDR504C	32.000	0.000
A_06_P2498	YDR505C	52.000	0.000
A_06_P2499	YDR506C	36.000	0.000
A_06_P2500	YDR507C	29.000	0.000
A_06_P2501	YDR508C	469.000	0.000
A_06_P2502	YDR509W	108.000	0.000
A_06_P2503	YDR510W	50.000	0.000
A_06_P2504	YDR511W	76.000	0.000
A_06_P2505	YDR512C	5.000	0.000
A_06_P2506	YDR513W	101.000	0.000

A_06_P2507	YDR514C	68.000	0.000
A_06_P2508	YDR515W	13.000	0.000
A_06_P2509	YDR516C	183.000	0.000
A_06_P2510	YDR517W	24.000	0.000
A_06_P2511	YDR518W	54.000	0.000
A_06_P2512	YDR519W	21.000	0.000
A_06_P2513	YDR520C	36.000	0.000
A_06_P2514	YDR521W	164.000	0.000
A_06_P2514	YPR158W-B	0.200	0.447
A_06_P2515	YDR522C	122.000	0.000
A_06_P2516	YDR523C	58.000	0.000
A_06_P2517	YDR524C	49.000	0.000
A_06_P2518	YDR525W	4.000	0.000
A_06_P2519	YDR525W-A	512.000	0.000
A_06_P2520	YDR526C	51.000	0.000
A_06_P2521	YDR527W	84.000	0.000
A_06_P2522	YDR528W	551.000	0.000
A_06_P2523	YDR529C	80.000	0.000
A_06_P2524	YDR530C	63.000	0.000
A_06_P2525	YDR531W	24.000	0.000
A_06_P2526	YDR532C	49.000	0.000
A_06_P2527	YDR533C	22.000	0.000
A_06_P2528	YDR534C	70.000	0.000
A_06_P2529	YDR535C	240.000	0.000
A_06_P2530	YDR536W	5.000	0.000
A_06_P2531	YDR537C	29.000	0.000
A_06_P2532	YDR538W	5.000	0.000
A_06_P2533	YDR539W	15.000	0.000
A_06_P2534	YDR540C	44.000	0.000
A_06_P2535	YDR541C	70.000	0.000
A_06_P2536	YBL108C-A	1.200	0.837
A_06_P2536	YDR542W	20.400	0.548
A_06_P2536	YEL049W	0.400	0.548
A_06_P2536	YGL261C	0.600	0.548
A_06_P2536	YGR294W	0.400	0.548
A_06_P2536	YIL176C	10.000	2.739
A_06_P2536	YJL223C	0.200	0.447
A_06_P2537	YAL068W-A	1.400	0.894
A_06_P2537	YDR543C	13.800	0.447
A_06_P2537	YER188C-A	466.400	2.966
A_06_P2537	YJR162C	27.200	6.221
A_06_P2537	YNR077C	0.600	0.894
A_06_P2537	YOL166W-A	15.800	0.447
A_06_P2538	YDR544C	433.000	0.000
A_06_P2540	YEL001C	237.000	0.000
A_06_P2541	YEL002C	328.000	0.000
A_06_P2542	YEL003W	8.000	0.000
A_06_P2543	YEL004W	84.000	0.000
A_06_P2544	YEL005C	10.000	0.000
A_06_P2545	YEL006W	18.000	0.000
A_06_P2546	YEL007W	23.000	0.000
A_06_P2547	YEL008W	56.000	0.000
A_06_P2548	YEL009C	25.000	0.000
A_06_P2549	YEL010W	72.000	0.000
A_06_P2550	YEL011W	10.000	0.000

A_06_P2551	YEL012W	33.000	0.000
A_06_P2552	YEL013W	36.000	0.000
A_06_P2553	YEL014C	10.000	0.000
A_06_P2554	YEL015W	15.000	0.000
A_06_P2554	YMR062C	0.200	0.447
A_06_P2555	YEL016C	268.000	0.000
A_06_P2556	YEL017C-A	46.000	0.000
A_06_P2557	YEL017W	26.000	0.000
A_06_P2558	YEL018W	84.000	0.000
A_06_P2559	YEL019C	17.000	0.000
A_06_P2560	YEL020C	68.000	0.000
A_06_P2561	YEL020W-A	127.000	0.000
A_06_P2562	YEL021W	5.000	0.000
A_06_P2563	YEL022W	350.000	0.000
A_06_P2564	YEL023C	16.000	0.000
A_06_P2565	YEL024W	4.000	0.000
A_06_P2566	YEL025C	105.000	0.000
A_06_P2567	YEL026W	34.800	0.447
A_06_P2568	YEL027W	10.000	0.000
A_06_P2569	YEL028W	112.000	0.000
A_06_P2570	YEL029C	220.000	0.000
A_06_P2571	YEL030W	8.000	0.000
A_06_P2572	YEL031W	728.000	0.000
A_06_P2573	YEL032W	75.000	0.000
A_06_P2574	YEL033W	37.000	0.000
A_06_P2575	YEL034W	29.000	0.000
A_06_P2576	YEL035C	44.000	0.000
A_06_P2577	YEL036C	36.000	0.000
A_06_P2578	YEL037C	77.000	0.000
A_06_P2579	YEL038W	124.000	0.000
A_06_P2580	YEL039C	10.000	0.000
A_06_P2581	YEL040W	93.000	0.000
A_06_P2582	YEL041W	250.000	0.000
A_06_P2583	YEL042W	58.000	0.000
A_06_P2584	YEL043W	45.000	0.000
A_06_P2585	YEL044W	35.000	0.000
A_06_P2586	YEL045C	28.000	0.000
A_06_P2587	YEL046C	89.000	0.000
A_06_P2588	YEL047C	289.000	0.000
A_06_P2589	YEL048C	82.000	0.000
A_06_P2590	YEL049W	233.200	6.221
A_06_P2591	YEL050C	492.000	0.000
A_06_P2592	YEL051W	11.000	0.000
A_06_P2593	YEL052W	26.000	0.000
A_06_P2594	YEL053C	53.000	0.000
A_06_P2595	YEL054C	25.000	0.000
A_06_P2596	YEL055C	120.000	0.000
A_06_P2597	YEL056W	17.000	0.000
A_06_P2598	YEL057C	150.000	0.000
A_06_P2599	YEL058W	36.000	0.000
A_06_P2600	YEL059C-A	24.000	0.000
A_06_P2601	YEL059W	17.000	0.000
A_06_P2602	YEL060C	16.000	0.000
A_06_P2603	YEL061C	32.000	0.000
A_06_P2604	YEL062W	77.000	0.000

A_06_P2605	YEL063C	82.000	0.000
A_06_P2606	YEL064C	155.000	0.000
A_06_P2607	YEL065W	112.000	0.000
A_06_P2608	YEL066W	19.000	0.000
A_06_P2609	YEL067C	16.000	0.000
A_06_P2610	YEL068C	17.000	0.000
A_06_P2611	YEL069C	99.800	0.447
A_06_P2612	YEL070W	168.600	0.548
A_06_P2613	YEL071W	14.000	0.000
A_06_P2614	YEL072W	45.000	0.000
A_06_P2615	YEL073C	57.000	0.000
A_06_P2616	YEL074W	131.000	0.000
A_06_P2617	YEL075C	265.200	9.783
A_06_P2617	YER189W	9.000	3.162
A_06_P2618	YEL076C	32.000	0.000
A_06_P2619	YDR545W	58.000	9.695
A_06_P2619	YEL076C-A	11.800	3.114
A_06_P2619	YER190W	73.600	5.683
A_06_P2619	YGR296W	21.000	4.183
A_06_P2619	YIL177C	5.200	2.168
A_06_P2619	YJL225C	36.600	4.980
A_06_P2619	YLR464W	17.200	2.280
A_06_P2619	YLR466W	2.000	1.000
A_06_P2619	YLR467W	31.400	5.857
A_06_P2619	YNL339C	71.200	6.535
A_06_P2619	YPL283C	4.800	0.837
A_06_P2620	YEL075W-A	65.000	0.000
A_06_P2621	YBL113C	0.800	0.447
A_06_P2621	YEL077C	287.800	8.497
A_06_P2621	YER190W	0.800	0.447
A_06_P2621	YHL050C	0.800	0.447
A_06_P2621	YLR467W	0.200	0.447
A_06_P2621	YNL339C	0.800	1.304
A_06_P2621	YOR396W	2.200	0.447
A_06_P2621	YPR204W	3.400	1.517
A_06_P2622	YER001W	18.000	0.000
A_06_P2623	YER002W	32.000	0.000
A_06_P2624	YER003C	76.000	0.000
A_06_P2625	YER004W	19.000	0.000
A_06_P2626	YER005W	165.000	0.000
A_06_P2627	YER006W	65.000	0.000
A_06_P2628	YER007C-A	84.000	0.000
A_06_P2629	YER007W	6.000	0.000
A_06_P2630	YER008C	39.000	0.000
A_06_P2631	YER009W	38.000	0.000
A_06_P2632	YER010C	21.000	0.000
A_06_P2633	YER011W	92.000	0.000
A_06_P2634	YER012W	12.000	0.000
A_06_P2635	YER013W	37.000	0.000
A_06_P2636	YER014C-A	151.000	0.000
A_06_P2637	YER014W	288.000	0.000
A_06_P2638	YER015W	23.000	0.000
A_06_P2639	YER016W	91.000	0.000
A_06_P2640	YER017C	37.000	0.000
A_06_P2641	YER018C	149.000	0.000

A_06_P2642	YER019C-A	56.000	0.000
A_06_P2643	YER019W	11.000	0.000
A_06_P2644	YER020W	21.000	0.000
A_06_P2645	YER021W	159.000	0.000
A_06_P2646	YER022W	17.000	0.000
A_06_P2647	YER023W	3.000	0.000
A_06_P2648	YER024W	5.000	0.000
A_06_P2649	YER025W	72.000	0.000
A_06_P2650	YER026C	318.000	0.000
A_06_P2651	YER027C	3.000	0.000
A_06_P2652	YER028C	96.000	0.000
A_06_P2653	YER029C	52.000	0.000
A_06_P2654	YER030W	61.000	0.000
A_06_P2655	YER031C	73.000	0.000
A_06_P2656	YER032W	107.000	0.000
A_06_P2657	YER033C	29.000	0.000
A_06_P2658	YER034W	17.000	0.000
A_06_P2659	YER035W	4.000	0.000
A_06_P2660	YER036C	46.000	0.000
A_06_P2661	YER037W	23.000	0.000
A_06_P2662	YER038C	121.000	0.000
A_06_P2663	YER039C	11.000	0.000
A_06_P2664	YER039C-A	313.000	0.000
A_06_P2665	YER040W	16.000	0.000
A_06_P2666	YER041W	60.000	0.000
A_06_P2667	YER042W	6.000	0.000
A_06_P2668	YER043C	42.000	0.000
A_06_P2669	YER044C	6.000	0.000
A_06_P2670	YER044C-A	41.000	0.000
A_06_P2671	YER045C	74.000	0.000
A_06_P2672	YER046W	74.000	0.000
A_06_P2673	YER047C	57.000	0.000
A_06_P2674	YER048C	33.000	0.000
A_06_P2675	YER048W-A	123.000	0.000
A_06_P2676	YER049W	26.000	0.000
A_06_P2677	YER050C	26.000	0.000
A_06_P2678	YER051W	87.000	0.000
A_06_P2679	YER052C	74.000	0.000
A_06_P2680	YER053C	77.000	0.000
A_06_P2681	YER053C-A	10.000	0.000
A_06_P2682	YER054C	20.000	0.000
A_06_P2683	YER055C	18.000	0.000
A_06_P2684	YER056C	15.000	0.000
A_06_P2685	YER056C-A	5.000	0.000
A_06_P2686	YER057C	36.000	0.000
A_06_P2687	YER058W	57.000	0.000
A_06_P2688	YER059W	22.000	0.000
A_06_P2689	YER060W	242.000	0.000
A_06_P2690	YER060W-A	28.000	0.000
A_06_P2691	YER061C	32.000	0.000
A_06_P2692	YER062C	118.000	0.000
A_06_P2693	YER063W	12.000	0.000
A_06_P2694	YER064C	14.000	0.000
A_06_P2695	YER065C	28.000	0.000
A_06_P2696	YER066C-A	206.000	0.000

A_06_P2697	YER066W	83.000	0.000
A_06_P2698	YER067W	41.000	0.000
A_06_P2699	YER068W	28.000	0.000
A_06_P2700	YER069W	2.000	0.000
A_06_P2701	YER070W	33.000	0.000
A_06_P2702	YER071C	183.000	0.000
A_06_P2703	YER072W	238.000	0.000
A_06_P2704	YER073W	55.000	0.000
A_06_P2705	YER074W	297.000	0.000
A_06_P2706	YER075C	13.000	0.000
A_06_P2707	YER076C	34.000	0.000
A_06_P2708	YER077C	75.000	0.000
A_06_P2709	YER078C	64.000	0.000
A_06_P2710	YER079W	13.000	0.000
A_06_P2711	YER080W	123.000	0.000
A_06_P2712	YER081W	222.000	0.000
A_06_P2713	YER082C	194.000	0.000
A_06_P2714	YER083C	13.000	0.000
A_06_P2715	YER084W	34.000	0.000
A_06_P2716	YER085C	229.000	0.000
A_06_P2717	YER086W	77.000	0.000
A_06_P2718	YER087W	12.000	0.000
A_06_P2719	YER088C	14.000	0.000
A_06_P2720	YER089C	15.000	0.000
A_06_P2721	YER090W	222.000	0.000
A_06_P2722	YER091C	39.000	0.000
A_06_P2723	YER091C-A	50.000	0.000
A_06_P2724	YER092W	38.000	0.000
A_06_P2725	YER093C	266.000	0.000
A_06_P2726	YER093C-A	27.000	0.000
A_06_P2727	YER094C	131.000	0.000
A_06_P2728	YER095W	31.000	0.000
A_06_P2729	YER096W	539.000	0.000
A_06_P2730	YER097W	84.000	0.000
A_06_P2731	YER098W	3.000	0.000
A_06_P2732	YER099C	120.000	0.000
A_06_P2733	YER100W	15.000	0.000
A_06_P2734	YER101C	139.000	0.000
A_06_P2735	YER102W	14.000	0.000
A_06_P2736	YER103W	100.000	0.000
A_06_P2737	YER104W	235.000	0.000
A_06_P2738	YER105C	75.000	0.000
A_06_P2739	YER106W	22.000	0.000
A_06_P2740	YER107C	56.000	0.000
A_06_P2741	YER109C	57.000	0.000
A_06_P2742	YER110C	25.000	0.000
A_06_P2743	YER111C	16.000	0.000
A_06_P2744	YER112W	47.000	0.000
A_06_P2745	YER113C	290.000	0.000
A_06_P2746	YER114C	975.000	0.000
A_06_P2747	YER115C	59.000	0.000
A_06_P2748	YER116C	91.000	0.000
A_06_P2749	YER117W	149.000	0.000
A_06_P2750	YER118C	96.000	0.000
A_06_P2751	YER119C	204.000	0.000

A_06_P2752	YER119C-A	37.000	0.000
A_06_P2753	YER120W	10.000	0.000
A_06_P2754	YER121W	62.000	0.000
A_06_P2755	YER122C	6.000	0.000
A_06_P2756	YER123W	116.000	0.000
A_06_P2757	YER124C	65.000	0.000
A_06_P2758	YER125W	232.000	0.000
A_06_P2759	YER126C	5.000	0.000
A_06_P2760	YER127W	117.000	0.000
A_06_P2761	YER128W	26.000	0.000
A_06_P2762	YER129W	4.000	0.000
A_06_P2763	YER130C	5.000	0.000
A_06_P2764	YER131W	909.000	0.000
A_06_P2765	YER132C	70.000	0.000
A_06_P2766	YER133W	179.000	0.000
A_06_P2767	YER134C	204.000	0.000
A_06_P2768	YER135C	125.000	0.000
A_06_P2769	YER136W	7.000	0.000
A_06_P2770	YER137C	17.000	0.000
A_06_P2771	YER138W-A	12.000	0.000
A_06_P2771	YOR192C-C	9.800	1.304
A_06_P2772	YER139C	122.000	0.000
A_06_P2773	YER140W	20.000	0.000
A_06_P2774	YER141W	39.000	0.000
A_06_P2775	YER142C	10.000	0.000
A_06_P2776	YER143W	25.000	0.000
A_06_P2777	YER144C	59.000	0.000
A_06_P2778	YER145C	99.000	0.000
A_06_P2779	YER146W	34.000	0.000
A_06_P2780	YER147C	6.000	0.000
A_06_P2781	YER148W	9.000	0.000
A_06_P2782	YER149C	23.000	0.000
A_06_P2783	YER150W	41.000	0.000
A_06_P2784	YER151C	45.000	0.000
A_06_P2785	YER152C	52.000	0.000
A_06_P2786	YER153C	155.000	0.000
A_06_P2787	YER154W	48.000	0.000
A_06_P2788	YER155C	850.000	0.000
A_06_P2789	YER156C	32.000	0.000
A_06_P2790	YER157W	66.000	0.000
A_06_P2791	YER158C	11.000	0.000
A_06_P2792	YER159C	87.000	0.000
A_06_P2793	YER161C	49.000	0.000
A_06_P2794	YER162C	72.000	0.000
A_06_P2795	YER163C	336.000	0.000
A_06_P2796	YER164W	101.000	0.000
A_06_P2797	YER165W	83.000	0.000
A_06_P2798	YER166W	52.000	0.000
A_06_P2799	YER167W	172.000	0.000
A_06_P2800	YER168C	15.000	0.000
A_06_P2801	YER169W	18.000	0.000
A_06_P2802	YER170W	239.000	0.000
A_06_P2803	YER171W	23.000	0.000
A_06_P2804	YER172C	107.000	0.000
A_06_P2805	YER173W	29.000	0.000

A_06_P2806	YER174C	25.000	0.000
A_06_P2807	YER175C	42.000	0.000
A_06_P2808	YER176W	30.000	0.000
A_06_P2809	YER177W	4.000	0.000
A_06_P2810	YER178W	59.000	0.000
A_06_P2811	YER179W	34.000	0.000
A_06_P2812	YER180C	16.000	0.000
A_06_P2813	YER181C	24.000	0.000
A_06_P2814	YER182W	74.000	0.000
A_06_P2815	YER183C	34.000	0.000
A_06_P2816	YER184C	11.000	0.000
A_06_P2817	YER185W	39.000	0.000
A_06_P2818	YER186C	14.000	0.000
A_06_P2819	YER187W	42.000	0.000
A_06_P2821	YER188W	37.000	0.000
A_06_P2822	YEL075C	265.000	9.247
A_06_P2822	YER189W	9.600	3.050
A_06_P2824	YFL001W	143.000	0.000
A_06_P2825	YFL002C	53.000	0.000
A_06_P2826	YFL003C	203.000	0.000
A_06_P2827	YFL004W	47.000	0.000
A_06_P2828	YFL005W	6.000	0.000
A_06_P2829	YFL007W	1.600	1.517
A_06_P2830	YFL007W	143.400	1.517
A_06_P2831	YFL008W	8.000	0.000
A_06_P2832	YFL009W	26.000	0.000
A_06_P2833	YFL010C	99.000	0.000
A_06_P2834	YFL010W-A	115.000	0.000
A_06_P2835	YFL011W	27.000	0.000
A_06_P2836	YFL012W	157.000	0.000
A_06_P2837	YFL013C	31.000	0.000
A_06_P2838	YFL012W-A	43.000	0.000
A_06_P2839	YFL014W	15.000	0.000
A_06_P2840	YFL015C	12.000	0.000
A_06_P2841	YFL016C	62.000	0.000
A_06_P2842	YFL017C	104.000	0.000
A_06_P2843	YFL017W-A	2.000	0.000
A_06_P2844	YFL018C	16.000	0.000
A_06_P2845	YFL019C	534.000	0.000
A_06_P2846	YFL020C	42.000	0.000
A_06_P2847	YFL021W	97.000	0.000
A_06_P2848	YFL022C	67.000	0.000
A_06_P2849	YFL023W	25.000	0.000
A_06_P2850	YFL024C	33.000	0.000
A_06_P2851	YFL025C	22.000	0.000
A_06_P2852	YFL026W	12.000	0.000
A_06_P2853	YFL027C	165.000	0.000
A_06_P2854	YFL028C	11.000	0.000
A_06_P2855	YFL029C	14.000	0.000
A_06_P2856	YFL030W	367.000	0.000
A_06_P2857	YFL031W	21.000	0.000
A_06_P2858	YFL032W	46.000	0.000
A_06_P2859	YFL033C	67.000	0.000
A_06_P2860	YFL034C-A	28.000	0.000
A_06_P2861	YFL034C-B	23.000	0.000

A_06_P2862	YFL034W	40.000	0.000
A_06_P2863	YFL036W	134.000	0.000
A_06_P2864	YFL037W	42.000	0.000
A_06_P2865	YFL038C	581.000	0.000
A_06_P2866	YFL039C	71.000	0.000
A_06_P2867	YFL040W	181.000	0.000
A_06_P2868	YFL041W	229.000	0.000
A_06_P2869	YFL042C	21.000	0.000
A_06_P2870	YFL044C	93.000	0.000
A_06_P2871	YFL045C	19.000	0.000
A_06_P2872	YFL046W	21.000	0.000
A_06_P2873	YFL047W	12.000	0.000
A_06_P2874	YFL048C	3.000	0.000
A_06_P2875	YFL049W	40.000	0.000
A_06_P2876	YFL050C	27.000	0.000
A_06_P2877	YFL051C	9.000	0.000
A_06_P2878	YFL052W	150.000	0.000
A_06_P2879	YFL053W	9.000	0.000
A_06_P2880	YFL054C	21.000	0.000
A_06_P2881	YFL055W	10.000	0.000
A_06_P2882	YFL056C	16.000	0.000
A_06_P2883	YFL057C	50.000	0.000
A_06_P2884	YFL058W	414.000	3.317
A_06_P2884	YNL332W	6.800	2.387
A_06_P2885	YFL059W	16.000	0.000
A_06_P2885	YNL333W	8.800	5.404
A_06_P2886	YFL060C	125.000	0.000
A_06_P2886	YNL334C	1.000	1.732
A_06_P2887	YFL061W	49.800	2.588
A_06_P2887	YNL335W	1.000	1.000
A_06_P2888	YFL062W	114.800	0.447
A_06_P2889	YCR108C	8.200	2.864
A_06_P2889	YFL063W	30.000	0.000
A_06_P2889	YOR394C-A	10.400	3.362
A_06_P2890	YEL075C	7.800	2.683
A_06_P2890	YER189W	0.400	0.548
A_06_P2890	YFL064C	156.000	0.000
A_06_P2890	YPR202W	87.400	2.074
A_06_P2891	YFL065C	17.000	0.707
A_06_P2891	YHL049C	4.200	2.168
A_06_P2891	YPR203W	1.800	1.095
A_06_P2892	YDR545W	0.200	0.447
A_06_P2892	YEL077C	12.200	4.817
A_06_P2892	YER190W	0.200	0.447
A_06_P2892	YFL066C	5.000	0.000
A_06_P2892	YGR296W	0.400	0.548
A_06_P2892	YLR467W	0.200	0.447
A_06_P2892	YNL339C	0.200	0.447
A_06_P2892	YOR396W	1.200	0.837
A_06_P2892	YPL283C	0.200	0.447
A_06_P2892	YPR204W	5.000	4.416
A_06_P2893	YER190C-A	52.000	0.000
A_06_P2893	YFL067W	160.000	0.000
A_06_P2893	YGR296C-A	59.000	0.000
A_06_P2893	YML133W-A	160.000	0.000

A_06_P2893	YNL339W-A	171.000	0.000
A_06_P2893	YPL283W-A	28.000	0.000
A_06_P2894	YBL113W-A	1.800	1.483
A_06_P2894	YDR545C-A	28.200	2.168
A_06_P2894	YEL077W-A	0.600	0.894
A_06_P2894	YER190C-B	48.800	1.924
A_06_P2894	YFL068W	32.000	0.000
A_06_P2894	YGR296C-B	1.600	1.140
A_06_P2894	YHL050W-A	127.200	2.168
A_06_P2894	YHR219C-A	265.600	0.894
A_06_P2894	YIL177W-A	0.400	0.894
A_06_P2894	YJL225W-A	0.600	0.894
A_06_P2894	YLL066W-A	24.800	0.447
A_06_P2894	YLL067W-A	99.800	0.447
A_06_P2894	YLR466C-A	45.200	4.764
A_06_P2894	YLR467C-A	5.400	1.517
A_06_P2894	YML133W-B	88.800	0.447
A_06_P2894	YNL339W-B	180.800	0.447
A_06_P2894	YOR396C-A	6.800	0.837
A_06_P2894	YPL283W-B	35.600	2.408
A_06_P2894	YPR204C-A	6.000	0.707
A_06_P2895	YFR001W	63.000	0.000
A_06_P2896	YFR002W	67.000	0.000
A_06_P2897	YFR003C	60.000	0.000
A_06_P2898	YFR004W	208.000	0.000
A_06_P2899	YFR005C	38.000	0.000
A_06_P2900	YFR006W	53.000	0.000
A_06_P2901	YFR007W	126.000	0.000
A_06_P2902	YFR008W	5.000	0.000
A_06_P2903	YFR009W	34.000	0.000
A_06_P2904	YFR010W	129.000	0.000
A_06_P2905	YFR011C	99.000	0.000
A_06_P2906	YFR012W	9.000	0.000
A_06_P2907	YFR012W-A	32.000	0.000
A_06_P2908	YFR013W	30.000	0.000
A_06_P2909	YFR014C	7.000	0.000
A_06_P2910	YFR015C	61.000	0.000
A_06_P2911	YFR016C	12.000	0.000
A_06_P2912	YFR017C	42.000	0.000
A_06_P2913	YFR018C	88.000	0.000
A_06_P2914	YFR019W	15.000	0.000
A_06_P2915	YFR020W	36.000	0.000
A_06_P2916	YFR021W	111.000	0.000
A_06_P2917	YFR022W	26.000	0.000
A_06_P2918	YFR023W	27.000	0.000
A_06_P2919	YFR024C-A	11.400	2.510
A_06_P2920	YFR024C-A	12.600	2.510
A_06_P2921	YFR025C	10.000	0.000
A_06_P2922	YFR026C	80.000	0.000
A_06_P2923	YFR027W	9.000	0.000
A_06_P2924	YFR028C	5.000	0.000
A_06_P2925	YFR029W	34.000	0.000
A_06_P2926	YFR030W	207.000	0.000
A_06_P2927	YFR031C	74.000	0.000
A_06_P2928	YFR031C-A	24.000	0.000

A_06_P2929	YFR032C	14.000	0.000
A_06_P2930	YFR032C-A	68.000	0.000
A_06_P2931	YFR033C	85.000	0.000
A_06_P2932	YFR034C	118.000	0.000
A_06_P2933	YFR035C	11.000	0.000
A_06_P2934	YFR036W	69.000	0.000
A_06_P2935	YFR037C	115.000	0.000
A_06_P2936	YFR038W	206.000	0.000
A_06_P2937	YFR039C	90.000	0.000
A_06_P2938	YFR040W	101.000	0.000
A_06_P2939	YFR041C	483.000	0.000
A_06_P2940	YFR042W	98.000	0.000
A_06_P2941	YFR043C	112.000	0.000
A_06_P2942	YFR044C	12.000	0.000
A_06_P2943	YFR045W	9.000	0.000
A_06_P2944	YFR046C	62.000	0.000
A_06_P2945	YFR047C	96.000	0.000
A_06_P2946	YFR048W	50.000	0.000
A_06_P2947	YFR049W	48.000	0.000
A_06_P2948	YFR050C	113.000	0.000
A_06_P2949	YFR051C	38.000	0.000
A_06_P2950	YFR052W	12.000	0.000
A_06_P2951	YFR053C	35.000	0.000
A_06_P2952	YFR054C	425.000	0.000
A_06_P2953	YFR055W	10.000	0.000
A_06_P2954	YFR056C	61.000	0.000
A_06_P2955	YFR057W	334.000	0.000
A_06_P2956	YGL001C	30.000	0.000
A_06_P2957	YGL002W	1.000	0.000
A_06_P2958	YGL003C	37.000	0.000
A_06_P2959	YGL004C	10.000	0.000
A_06_P2960	YGL005C	10.000	0.000
A_06_P2961	YGL006W	10.000	0.000
A_06_P2962	YGL007W	9.000	0.000
A_06_P2963	YGL008C	4.000	0.000
A_06_P2964	YGL009C	46.000	0.000
A_06_P2965	YGL010W	307.000	0.000
A_06_P2966	YGL011C	169.000	0.000
A_06_P2967	YGL012W	19.000	0.000
A_06_P2968	YGL013C	14.000	0.000
A_06_P2969	YGL014W	14.000	0.000
A_06_P2970	YGL015C	648.000	0.000
A_06_P2971	YGL016W	131.000	0.000
A_06_P2972	YGL017W	72.000	0.000
A_06_P2973	YGL018C	15.000	0.000
A_06_P2974	YGL019W	147.000	0.000
A_06_P2975	YGL020C	21.000	0.000
A_06_P2976	YGL021W	368.000	0.000
A_06_P2977	YGL022W	74.000	0.000
A_06_P2978	YGL023C	25.000	0.000
A_06_P2979	YGL024W	63.000	0.000
A_06_P2980	YGL025C	15.000	0.000
A_06_P2981	YGL026C	188.000	0.000
A_06_P2982	YGL027C	44.000	0.000
A_06_P2983	YGL028C	48.000	0.000

A_06_P2984	YGL029W	26.000	0.000
A_06_P2985	YGL030W	32.000	0.000
A_06_P2986	YGL031C	11.000	0.000
A_06_P2987	YGL032C	3.000	0.000
A_06_P2988	YGL033W	140.000	0.000
A_06_P2989	YGL034C	152.000	0.000
A_06_P2990	YGL035C	42.000	0.000
A_06_P2991	YGL036W	14.000	0.000
A_06_P2992	YGL037C	28.000	0.000
A_06_P2993	YGL038C	167.000	0.000
A_06_P2994	YGL039W	25.000	0.000
A_06_P2995	YGL040C	67.000	0.000
A_06_P2996	YGL041C	121.000	0.000
A_06_P2997	YGL042C	14.000	0.000
A_06_P2998	YGL043W	48.000	0.000
A_06_P2999	YGL044C	11.000	0.000
A_06_P3000	YGL045W	38.000	0.000
A_06_P3002	YGL047W	64.000	0.000
A_06_P3003	YGL048C	60.000	0.000
A_06_P3004	YGL049C	81.000	0.000
A_06_P3005	YGL050W	129.000	0.000
A_06_P3006	YGL051W	144.000	0.000
A_06_P3007	YGL052W	115.000	0.000
A_06_P3008	YGL053W	480.000	0.000
A_06_P3009	YGL054C	24.000	0.000
A_06_P3010	YGL055W	4.000	0.000
A_06_P3011	YGL056C	44.000	0.000
A_06_P3012	YGL057C	45.000	0.000
A_06_P3013	YGL058W	24.000	0.000
A_06_P3014	YGL059W	260.000	0.000
A_06_P3015	YGL060W	29.000	0.000
A_06_P3016	YGL061C	25.000	0.000
A_06_P3017	YGL062W	5.000	0.000
A_06_P3018	YGL063W	107.000	0.000
A_06_P3019	YGL064C	75.000	0.000
A_06_P3020	YGL065C	34.000	0.000
A_06_P3021	YGL066W	29.000	0.000
A_06_P3022	YGL067W	28.000	0.000
A_06_P3023	YGL068W	27.000	0.000
A_06_P3024	YGL069C	67.000	0.000
A_06_P3025	YGL070C	5.000	0.000
A_06_P3026	YGL071W	17.000	0.000
A_06_P3027	YGL072C	8.000	0.000
A_06_P3028	YGL073W	31.000	0.000
A_06_P3029	YGL074C	84.000	0.000
A_06_P3030	YGL075C	44.000	0.000
A_06_P3031	YGL076C	46.000	0.000
A_06_P3032	YGL077C	21.000	0.000
A_06_P3033	YGL078C	106.000	0.000
A_06_P3034	YGL079W	136.000	0.000
A_06_P3035	YGL080W	22.000	0.000
A_06_P3036	YGL081W	22.000	0.000
A_06_P3037	YGL082W	11.000	0.000
A_06_P3038	YGL083W	71.000	0.000
A_06_P3039	YGL084C	19.000	0.000

A_06_P3040	YGL085W	55.000	0.000
A_06_P3041	YGL086W	270.000	0.000
A_06_P3042	YGL087C	4.000	0.000
A_06_P3043	YGL088W	21.000	0.000
A_06_P3044	YGL089C	30.000	0.000
A_06_P3045	YGL090W	24.000	0.000
A_06_P3046	YGL091C	430.000	0.000
A_06_P3047	YGL092W	186.000	0.000
A_06_P3048	YGL093W	118.000	0.000
A_06_P3049	YGL094C	33.000	0.000
A_06_P3050	YGL095C	86.000	0.000
A_06_P3051	YGL096W	22.000	0.000
A_06_P3052	YGL097W	71.000	0.000
A_06_P3053	YGL098W	10.000	0.000
A_06_P3054	YGL099W	201.000	0.000
A_06_P3055	YGL100W	31.000	0.000
A_06_P3056	YGL101W	29.000	0.000
A_06_P3057	YGL102C	4.000	0.000
A_06_P3058	YGL103W	17.000	0.000
A_06_P3059	YGL104C	6.000	0.000
A_06_P3060	YGL105W	88.000	0.000
A_06_P3061	YGL106W	48.000	0.000
A_06_P3062	YGL107C	3.000	0.000
A_06_P3063	YGL108C	9.000	0.000
A_06_P3064	YGL109W	24.000	0.000
A_06_P3065	YGL110C	22.000	0.000
A_06_P3066	YGL111W	43.000	0.000
A_06_P3067	YGL112C	42.000	0.000
A_06_P3068	YGL113W	485.000	0.000
A_06_P3069	YGL114W	3.000	0.000
A_06_P3070	YGL115W	7.000	0.000
A_06_P3071	YGL116W	24.000	0.000
A_06_P3072	YGL117W	57.000	0.000
A_06_P3073	YGL118C	165.000	0.000
A_06_P3074	YGL119W	164.000	0.000
A_06_P3075	YGL120C	15.000	0.000
A_06_P3076	YGL121C	15.000	0.000
A_06_P3077	YGL122C	36.000	0.000
A_06_P3078	YGL123W	92.000	0.000
A_06_P3079	YGL124C	39.000	0.000
A_06_P3080	YGL125W	36.000	0.000
A_06_P3081	YGL126W	8.000	0.000
A_06_P3082	YGL127C	29.000	0.000
A_06_P3083	YGL128C	1000.000	0.000
A_06_P3084	YGL129C	349.000	0.000
A_06_P3085	YGL130W	28.000	0.000
A_06_P3086	YGL131C	82.000	0.000
A_06_P3087	YGL132W	626.000	0.000
A_06_P3088	YGL133W	169.000	0.000
A_06_P3089	YGL134W	12.000	0.000
A_06_P3090	YGL135W	83.000	0.000
A_06_P3090	YPL220W	15.200	3.421
A_06_P3091	YGL136C	92.000	0.000
A_06_P3092	YGL137W	10.000	0.000
A_06_P3093	YGL138C	10.000	0.000

A_06_P3094	YGL139W	6.000	0.000
A_06_P3095	YGL140C	20.000	0.000
A_06_P3096	YGL141W	3.000	0.000
A_06_P3097	YGL142C	29.000	0.000
A_06_P3098	YGL143C	81.000	0.000
A_06_P3099	YGL144C	85.000	0.000
A_06_P3100	YGL145W	44.000	0.000
A_06_P3101	YGL146C	12.000	0.000
A_06_P3102	YGL147C	193.000	0.000
A_06_P3103	YGL148W	345.000	0.000
A_06_P3104	YGL149W	473.000	0.000
A_06_P3105	YGL150C	15.000	0.000
A_06_P3106	YGL151W	38.000	0.000
A_06_P3107	YGL152C	20.000	0.000
A_06_P3108	YGL153W	47.000	0.000
A_06_P3109	YGL154C	3.000	0.000
A_06_P3110	YGL155W	100.000	0.000
A_06_P3111	YGL156W	56.000	0.000
A_06_P3112	YGL157W	97.000	0.000
A_06_P3113	YGL158W	342.000	0.000
A_06_P3114	YGL159W	7.000	0.000
A_06_P3115	YGL160W	36.000	0.000
A_06_P3116	YGL161C	67.000	0.000
A_06_P3117	YGL162W	15.000	0.000
A_06_P3118	YGL163C	108.000	0.000
A_06_P3119	YGL164C	34.000	0.000
A_06_P3120	YGL165C	4.000	0.000
A_06_P3121	YGL166W	37.000	0.000
A_06_P3122	YGL167C	51.000	0.000
A_06_P3123	YGL168W	27.000	0.000
A_06_P3124	YGL169W	128.000	0.000
A_06_P3125	YGL170C	23.000	0.000
A_06_P3126	YGL171W	8.000	0.000
A_06_P3127	YGL172W	38.000	0.000
A_06_P3128	YGL173C	27.000	0.000
A_06_P3129	YGL174W	89.000	0.000
A_06_P3130	YGL175C	41.000	0.000
A_06_P3131	YGL176C	12.000	0.000
A_06_P3132	YGL177W	117.000	0.000
A_06_P3133	YGL178W	43.000	0.000
A_06_P3134	YGL179C	43.000	0.000
A_06_P3135	YGL180W	27.000	0.000
A_06_P3136	YGL181W	75.000	0.000
A_06_P3137	YGL182C	48.000	0.000
A_06_P3138	YGL183C	74.000	0.000
A_06_P3139	YGL184C	11.000	0.000
A_06_P3140	YGL185C	7.000	0.000
A_06_P3141	YGL186C	148.000	0.000
A_06_P3142	YGL187C	19.000	0.000
A_06_P3143	YGL188C	4.000	0.000
A_06_P3144	YGL189C	357.000	0.000
A_06_P3145	YGL190C	204.000	0.000
A_06_P3146	YGL191W	202.000	0.000
A_06_P3147	YGL192W	60.000	0.000
A_06_P3148	YGL193C	33.000	0.000

A_06_P3149	YGL194C	56.000	0.000
A_06_P3150	YGL195W	9.000	0.000
A_06_P3151	YGL196W	57.000	0.000
A_06_P3152	YGL197W	195.000	0.000
A_06_P3154	YGL199C	115.000	0.000
A_06_P3155	YGL200C	12.000	0.000
A_06_P3156	YGL201C	59.000	0.000
A_06_P3157	YGL202W	60.000	0.000
A_06_P3158	YGL203C	168.000	0.000
A_06_P3159	YGL204C	46.000	0.000
A_06_P3160	YGL205W	85.000	0.000
A_06_P3161	YGL206C	73.000	0.000
A_06_P3162	YGL207W	12.000	0.000
A_06_P3163	YGL208W	21.000	0.000
A_06_P3164	YGL209W	32.000	0.000
A_06_P3165	YGL210W	70.000	0.000
A_06_P3166	YGL211W	47.000	0.000
A_06_P3167	YGL212W	200.000	0.000
A_06_P3168	YGL213C	8.000	0.000
A_06_P3169	YGL214W	4.000	0.000
A_06_P3170	YGL215W	3.000	0.000
A_06_P3171	YGL216W	333.000	0.000
A_06_P3172	YGL217C	89.000	0.000
A_06_P3173	YGL218W	5.000	0.000
A_06_P3174	YGL219C	41.000	0.000
A_06_P3175	YGL220W	17.000	0.000
A_06_P3176	YGL221C	154.000	0.000
A_06_P3177	YGL222C	61.000	0.000
A_06_P3178	YGL223C	38.000	0.000
A_06_P3179	YGL224C	184.000	0.000
A_06_P3180	YGL225W	29.000	0.000
A_06_P3181	YGL226C-A	22.000	0.000
A_06_P3182	YGL226W	56.000	0.000
A_06_P3183	YGL227W	966.000	0.000
A_06_P3184	YGL228W	1.000	0.000
A_06_P3185	YGL229C	89.000	0.000
A_06_P3186	YGL230C	30.000	0.000
A_06_P3187	YGL231C	66.000	0.000
A_06_P3188	YGL232W	25.000	0.000
A_06_P3189	YGL233W	502.000	0.000
A_06_P3190	YGL234W	194.000	0.000
A_06_P3191	YGL235W	318.000	0.000
A_06_P3192	YGL236C	19.000	0.000
A_06_P3193	YGL237C	19.000	0.000
A_06_P3194	YGL238W	108.000	0.000
A_06_P3195	YGL239C	79.000	0.000
A_06_P3196	YGL240W	81.000	0.000
A_06_P3197	YGL241W	106.000	0.000
A_06_P3198	YGL242C	41.000	0.000
A_06_P3199	YGL243W	65.000	0.000
A_06_P3200	YGL244W	21.000	0.000
A_06_P3201	YGL245W	96.000	0.000
A_06_P3202	YGL246C	91.000	0.000
A_06_P3203	YGL247W	95.000	0.000
A_06_P3204	YGL248W	28.000	0.000

A_06_P3205	YGL249W	276.000	0.000
A_06_P3206	YGL250W	8.000	0.000
A_06_P3207	YGL251C	242.000	0.000
A_06_P3208	YGL252C	36.000	0.000
A_06_P3209	YGL253W	19.000	0.000
A_06_P3210	YGL254W	120.000	0.000
A_06_P3211	YGL255W	296.000	0.000
A_06_P3212	YGL256W	5.000	0.000
A_06_P3213	YGL257C	277.000	0.000
A_06_P3214	YGL258W	28.000	0.000
A_06_P3215	YGL258W-A	63.000	0.000
A_06_P3216	YGL259W	240.000	0.000
A_06_P3217	YGL260W	33.000	3.391
A_06_P3217	YIR040C	0.200	0.447
A_06_P3217	YJL222W-B	21.000	0.000
A_06_P3218	YLR461W	0.200	0.447
A_06_P3219	YGL262W	344.000	0.000
A_06_P3220	YGL263W	106.000	0.000
A_06_P3221	YGR001C	52.000	0.000
A_06_P3222	YGR002C	53.000	0.000
A_06_P3223	YGR003W	38.000	0.000
A_06_P3224	YGR004W	23.000	0.000
A_06_P3225	YGR005C	164.000	0.000
A_06_P3226	YGR006W	23.000	0.000
A_06_P3227	YGR007W	64.000	0.000
A_06_P3228	YGR008C	66.000	0.000
A_06_P3229	YGR009C	111.000	0.000
A_06_P3230	YGR010W	640.000	0.000
A_06_P3231	YGR011W	6.000	0.000
A_06_P3232	YGR012W	120.000	0.000
A_06_P3233	YGR013W	45.000	0.000
A_06_P3234	YGR014W	42.000	0.000
A_06_P3235	YGR015C	29.000	0.000
A_06_P3236	YGR016W	1000.000	0.000
A_06_P3237	YGR017W	171.000	0.000
A_06_P3238	YGR018C	14.000	0.000
A_06_P3239	YGR019W	57.000	0.000
A_06_P3240	YGR020C	217.000	0.000
A_06_P3241	YGR021W	44.000	0.000
A_06_P3242	YGR022C	224.000	0.000
A_06_P3243	YGR023W	19.000	0.000
A_06_P3244	YGR024C	125.000	0.000
A_06_P3245	YGR025W	16.000	0.000
A_06_P3246	YGR026W	85.000	0.000
A_06_P3247	YGR027C	29.000	0.000
A_06_P3248	YGR028W	90.000	0.000
A_06_P3249	YGR029W	39.000	0.000
A_06_P3250	YGR030C	32.000	0.000
A_06_P3251	YGR031W	37.000	0.000
A_06_P3252	YGR032W	20.000	0.000
A_06_P3253	YGR033C	28.000	0.000
A_06_P3254	YGR034W	12.000	0.000
A_06_P3255	YGR035C	25.000	0.000
A_06_P3256	YGR036C	20.000	0.000
A_06_P3257	YGR037C	89.000	0.000

A_06_P3258	YGR038W	52.000	0.000
A_06_P3259	YGR039W	106.000	0.000
A_06_P3260	YGR040W	11.000	0.000
A_06_P3261	YGR041W	16.000	0.000
A_06_P3262	YGR042W	51.000	0.000
A_06_P3263	YGR043C	123.000	0.000
A_06_P3264	YGR044C	44.000	0.000
A_06_P3265	YGR045C	15.000	0.000
A_06_P3266	YGR046W	46.000	0.000
A_06_P3267	YGR047C	69.000	0.000
A_06_P3268	YGR048W	1000.000	0.000
A_06_P3269	YGR049W	19.000	0.000
A_06_P3270	YGR050C	85.000	0.000
A_06_P3271	YGR051C	154.000	0.000
A_06_P3272	YGR052W	16.000	0.000
A_06_P3273	YGR053C	238.000	0.000
A_06_P3274	YGR054W	69.000	0.000
A_06_P3275	YGR055W	191.000	0.000
A_06_P3276	YGR056W	32.000	0.000
A_06_P3277	YGR057C	263.000	0.000
A_06_P3278	YGR058W	32.000	0.000
A_06_P3279	YGR059W	72.000	0.000
A_06_P3280	YGR060W	145.000	0.000
A_06_P3281	YGR061C	57.000	0.000
A_06_P3282	YGR062C	315.000	0.000
A_06_P3283	YGR063C	62.000	0.000
A_06_P3284	YGR064W	28.000	0.000
A_06_P3285	YGR065C	11.000	0.000
A_06_P3286	YGR066C	85.000	0.000
A_06_P3287	YGR067C	11.000	0.000
A_06_P3288	YGR068C	230.000	0.000
A_06_P3289	YGR069W	31.000	0.000
A_06_P3290	YGR070W	19.000	0.000
A_06_P3291	YGR071C	61.000	0.000
A_06_P3292	YGR072W	33.000	0.000
A_06_P3293	YGR073C	47.000	0.000
A_06_P3294	YGR074W	3.000	0.000
A_06_P3295	YGR075C	30.000	0.000
A_06_P3296	YGR076C	440.000	0.000
A_06_P3297	YGR077C	11.000	0.000
A_06_P3298	YGR078C	17.000	0.000
A_06_P3299	YGR079W	305.000	0.000
A_06_P3300	YGR080W	39.000	0.000
A_06_P3301	YGR081C	625.000	0.000
A_06_P3302	YGR082W	116.000	0.000
A_06_P3303	YGR083C	66.000	0.000
A_06_P3304	YGR084C	30.000	0.000
A_06_P3305	YGR085C	25.000	0.000
A_06_P3306	YGR086C	65.000	0.000
A_06_P3307	YGR087C	7.000	0.000
A_06_P3308	YGR088W	151.000	0.000
A_06_P3309	YGR089W	319.000	0.000
A_06_P3310	YGR090W	14.000	0.000
A_06_P3311	YGR091W	48.000	0.000
A_06_P3312	YGR092W	105.000	0.000

A_06_P3313	YGR093W	31.000	0.000
A_06_P3314	YGR094W	24.000	0.000
A_06_P3315	YGR095C	37.000	0.000
A_06_P3316	YGR096W	25.000	0.000
A_06_P3317	YGR097W	157.000	0.000
A_06_P3318	YGR098C	35.000	0.000
A_06_P3319	YGR099W	83.000	0.000
A_06_P3320	YGR100W	18.000	0.000
A_06_P3321	YGR101W	72.000	0.000
A_06_P3322	YGR102C	108.000	0.000
A_06_P3323	YGR103W	41.000	0.000
A_06_P3324	YGR104C	111.000	0.000
A_06_P3325	YGR105W	46.000	0.000
A_06_P3326	YGR106C	341.000	0.000
A_06_P3327	YGR107W	12.000	0.000
A_06_P3328	YGR108W	22.000	0.000
A_06_P3329	YGR109C	50.000	0.000
A_06_P3330	YGR110W	124.000	0.000
A_06_P3331	YGR111W	621.000	0.000
A_06_P3332	YGR112W	36.000	0.000
A_06_P3333	YGR113W	59.000	0.000
A_06_P3334	YGR114C	22.000	0.000
A_06_P3335	YGR115C	18.000	0.000
A_06_P3336	YGR116W	261.000	0.000
A_06_P3337	YGR117C	14.000	0.000
A_06_P3338	YGR118W	18.000	0.000
A_06_P3339	YGR119C	12.000	0.000
A_06_P3340	YGR120C	86.000	0.000
A_06_P3341	YGR121C	205.000	0.000
A_06_P3343	YGR122W	8.000	0.000
A_06_P3344	YGR123C	27.000	0.000
A_06_P3345	YGR124W	8.000	0.000
A_06_P3346	YGR125W	35.000	0.000
A_06_P3347	YGR126W	256.000	0.000
A_06_P3348	YGR127W	58.000	0.000
A_06_P3349	YGR128C	31.000	0.000
A_06_P3350	YGR129W	70.000	0.000
A_06_P3351	YGR130C	22.000	0.000
A_06_P3352	YGR131W	10.000	0.000
A_06_P3353	YGR132C	52.000	0.000
A_06_P3354	YGR133W	170.000	0.000
A_06_P3355	YGR134W	44.000	0.000
A_06_P3356	YGR135W	147.000	0.000
A_06_P3357	YGR136W	19.000	0.000
A_06_P3358	YGR137W	21.000	0.000
A_06_P3359	YGR138C	18.000	0.000
A_06_P3360	YGR139W	18.000	0.000
A_06_P3361	YGR140W	69.000	0.000
A_06_P3362	YGR141W	94.000	0.000
A_06_P3363	YGR142W	17.000	0.000
A_06_P3364	YGR143W	68.000	0.000
A_06_P3365	YGR144W	59.000	0.000
A_06_P3366	YGR145W	249.000	0.000
A_06_P3367	YGR146C	109.000	0.000
A_06_P3368	YGR147C	58.000	0.000

A_06_P3369	YGR148C	8.000	0.000
A_06_P3370	YGR149W	23.000	0.000
A_06_P3371	YGR150C	1000.000	0.000
A_06_P3372	YGR151C	32.000	0.000
A_06_P3373	YGR152C	12.000	0.000
A_06_P3374	YGR153W	60.000	0.000
A_06_P3375	YGR154C	22.000	0.000
A_06_P3376	YGR155W	149.000	0.000
A_06_P3377	YGR156W	81.000	0.000
A_06_P3378	YGR157W	107.000	0.000
A_06_P3379	YGR158C	87.000	0.000
A_06_P3380	YGR159C	30.000	0.000
A_06_P3381	YGR160W	53.000	0.000
A_06_P3382	YGR161C	262.000	0.000
A_06_P3383	YGR162W	10.000	0.000
A_06_P3384	YGR163W	7.000	0.000
A_06_P3385	YGR164W	101.000	0.000
A_06_P3386	YGR165W	18.000	0.000
A_06_P3387	YGR166W	243.000	0.000
A_06_P3388	YGR167W	75.000	0.000
A_06_P3389	YGR168C	63.000	0.000
A_06_P3390	YGR169C	28.000	0.000
A_06_P3391	YGR170W	65.000	0.000
A_06_P3392	YGR171C	41.000	0.000
A_06_P3393	YGR172C	18.000	0.000
A_06_P3394	YGR173W	18.000	0.000
A_06_P3395	YGR174C	155.000	0.000
A_06_P3396	YGR175C	21.000	0.000
A_06_P3397	YGR176W	41.000	0.000
A_06_P3398	YGR177C	36.000	0.000
A_06_P3399	YGR178C	198.000	0.000
A_06_P3400	YGR179C	52.000	0.000
A_06_P3401	YGR180C	120.000	0.000
A_06_P3402	YGR181W	18.000	0.000
A_06_P3403	YGR182C	81.000	0.000
A_06_P3404	YGR183C	48.000	0.000
A_06_P3405	YGR184C	303.000	0.000
A_06_P3406	YGR185C	66.000	0.000
A_06_P3407	YGR186W	3.000	0.000
A_06_P3408	YGR187C	69.000	0.000
A_06_P3409	YGR188C	14.000	0.000
A_06_P3410	YGR189C	27.000	0.000
A_06_P3411	YGR190C	440.000	0.000
A_06_P3412	YGR191W	81.000	0.000
A_06_P3413	YGR192C	125.000	0.000
A_06_P3414	YGR193C	16.000	0.000
A_06_P3415	YGR194C	4.000	0.000
A_06_P3416	YGR195W	43.000	0.000
A_06_P3417	YGR196C	20.000	0.000
A_06_P3418	YGR197C	144.000	0.000
A_06_P3419	YGR198W	214.000	0.000
A_06_P3420	YGR199W	9.000	0.000
A_06_P3421	YGR200C	95.000	0.000
A_06_P3422	YGR201C	46.000	0.000
A_06_P3423	YGR202C	144.000	0.000

A_06_P3424	YGR203W	19.000	0.000
A_06_P3425	YGR204W	81.000	0.000
A_06_P3426	YGR205W	57.000	0.000
A_06_P3427	YGR206W	50.000	0.000
A_06_P3428	YGR207C	57.000	0.000
A_06_P3429	YGR208W	22.000	0.000
A_06_P3430	YGR209C	16.000	0.000
A_06_P3431	YGR210C	20.000	0.000
A_06_P3432	YGR211W	96.000	0.000
A_06_P3433	YGR212W	39.000	0.000
A_06_P3434	YGR213C	104.000	0.000
A_06_P3435	YGR214W	330.000	0.000
A_06_P3436	YGR215W	31.000	0.000
A_06_P3437	YGR216C	56.000	0.000
A_06_P3438	YGR217W	15.000	0.000
A_06_P3439	YGR218W	7.000	0.000
A_06_P3440	YGR219W	137.000	0.000
A_06_P3441	YGR220C	2.000	0.000
A_06_P3442	YGR221C	7.000	0.000
A_06_P3443	YGR222W	107.000	0.000
A_06_P3444	YGR223C	40.000	0.000
A_06_P3445	YGR224W	32.000	0.000
A_06_P3446	YGR225W	76.000	0.000
A_06_P3448	YGR227W	35.000	0.000
A_06_P3449	YGR228W	17.000	0.000
A_06_P3450	YGR229C	19.000	0.000
A_06_P3451	YGR230W	99.000	0.000
A_06_P3452	YGR231C	41.000	0.000
A_06_P3453	YGR232W	41.000	0.000
A_06_P3454	YGR233C	38.000	0.000
A_06_P3455	YGR234W	142.000	0.000
A_06_P3456	YGR235C	20.000	0.000
A_06_P3457	YGR236C	11.400	1.817
A_06_P3458	YGR237C	67.000	0.000
A_06_P3459	YGR238C	77.000	0.000
A_06_P3460	YGR239C	73.000	0.000
A_06_P3461	YGR240C	60.000	0.000
A_06_P3462	YGR241C	593.000	0.000
A_06_P3463	YGR242W	46.000	0.000
A_06_P3464	YGR243W	9.000	0.000
A_06_P3465	YGR244C	50.000	0.000
A_06_P3466	YGR245C	7.000	0.000
A_06_P3467	YGR246C	9.000	0.000
A_06_P3468	YGR247W	20.000	0.000
A_06_P3469	YGR248W	122.000	0.000
A_06_P3470	YGR249W	62.000	0.000
A_06_P3471	YGR250C	59.000	0.000
A_06_P3472	YGR251W	13.000	0.000
A_06_P3473	YGR252W	7.000	0.000
A_06_P3474	YGR253C	143.000	0.000
A_06_P3475	YGR254W	61.000	0.000
A_06_P3476	YGR255C	73.000	0.000
A_06_P3477	YGR256W	71.000	0.000
A_06_P3478	YGR257C	21.000	0.000
A_06_P3479	YGR258C	21.000	0.000

A_06_P3480	YGR259C	261.000	0.000
A_06_P3481	YGR260W	487.000	0.000
A_06_P3482	YGR261C	23.000	0.000
A_06_P3483	YGR262C	131.000	0.000
A_06_P3484	YGR263C	93.000	0.000
A_06_P3485	YGR264C	31.000	0.000
A_06_P3486	YGR265W	278.000	0.000
A_06_P3487	YGR266W	68.000	0.000
A_06_P3488	YGR267C	1.000	0.000
A_06_P3489	YGR268C	17.000	0.000
A_06_P3490	YGR269W	78.000	0.000
A_06_P3491	YGR270W	902.000	0.000
A_06_P3492	YGR271C-A	55.000	0.000
A_06_P3493	YGR271W	38.000	0.000
A_06_P3494	YGR272C	232.000	0.000
A_06_P3495	YGR273C	127.000	0.000
A_06_P3496	YGR274C	311.000	0.000
A_06_P3497	YGR275W	76.000	0.000
A_06_P3498	YGR276C	24.000	0.000
A_06_P3499	YGR277C	65.000	0.000
A_06_P3500	YGR278W	269.000	0.000
A_06_P3501	YGR279C	3.000	0.000
A_06_P3502	YGR280C	36.000	0.000
A_06_P3503	YGR281W	147.000	0.000
A_06_P3504	YGR282C	26.000	0.000
A_06_P3505	YGR283C	43.000	0.000
A_06_P3506	YGR284C	15.000	0.000
A_06_P3507	YGR285C	64.000	0.000
A_06_P3508	YGR286C	125.000	0.000
A_06_P3509	YGR287C	30.000	0.000
A_06_P3510	YGR288W	188.000	0.000
A_06_P3511	YGR289C	73.000	0.000
A_06_P3512	YGR290W	46.000	0.000
A_06_P3513	YBR298C-A	12.000	0.000
A_06_P3513	YGR291C	7.000	0.000
A_06_P3514	YBR299W	0.200	0.447
A_06_P3514	YGR292W	114.000	0.000
A_06_P3515	YGR293C	124.000	0.000
A_06_P3516	YBR301W	4.000	1.581
A_06_P3516	YDR542W	0.200	0.447
A_06_P3516	YEL049W	0.200	0.447
A_06_P3516	YGL261C	0.200	0.447
A_06_P3516	YGR294W	30.200	3.564
A_06_P3516	YHL046C	1.200	1.643
A_06_P3516	YIL176C	1.400	1.517
A_06_P3516	YIR041W	1.000	0.707
A_06_P3516	YJR150C	15.000	3.536
A_06_P3516	YKL224C	0.200	0.447
A_06_P3516	YLL064C	13.200	2.490
A_06_P3516	YLR461W	67.000	6.519
A_06_P3516	YMR325W	1.000	0.707
A_06_P3516	YNR076W	3.600	1.949
A_06_P3516	YOL161C	1.600	1.517
A_06_P3517	YGR295C	439.000	0.000
A_06_P3518	YPR204W	0.200	0.447

A_06_P3519	YHL001W	38.000	0.000
A_06_P3520	YHL002W	120.000	0.000
A_06_P3521	YHL003C	40.000	0.000
A_06_P3522	YHL004W	163.000	0.000
A_06_P3523	YHL005C	54.000	0.000
A_06_P3524	YHL006C	72.200	10.035
A_06_P3525	YHL007C	72.000	0.000
A_06_P3526	YHL008C	115.000	0.000
A_06_P3527	YHL009C	147.000	0.000
A_06_P3528	YHL010C	120.000	0.000
A_06_P3529	YHL011C	47.000	0.000
A_06_P3530	YHL012W	23.000	0.000
A_06_P3531	YHL013C	12.000	0.000
A_06_P3532	YHL014C	176.000	0.000
A_06_P3533	YHL015W	30.000	0.000
A_06_P3534	YHL016C	53.000	0.000
A_06_P3535	YHL017W	226.000	0.000
A_06_P3536	YHL018W	70.000	0.000
A_06_P3537	YHL019C	22.000	0.000
A_06_P3538	YHL020C	18.000	0.000
A_06_P3539	YHL021C	9.000	0.000
A_06_P3540	YHL022C	43.000	0.000
A_06_P3541	YHL023C	10.000	0.000
A_06_P3542	YHL024W	20.000	0.000
A_06_P3543	YHL025W	74.000	0.000
A_06_P3544	YHL026C	87.000	0.000
A_06_P3545	YHL027W	4.000	0.000
A_06_P3546	YHL028W	3.000	0.000
A_06_P3547	YHL029C	20.000	0.000
A_06_P3548	YHL030W	31.000	0.000
A_06_P3549	YHL031C	131.000	0.000
A_06_P3550	YHL032C	89.000	0.000
A_06_P3551	YHL033C	44.000	0.000
A_06_P3552	YHL034C	49.000	0.000
A_06_P3553	YHL035C	116.000	0.000
A_06_P3554	YHL036W	92.000	0.000
A_06_P3555	YHL037C	33.000	0.000
A_06_P3556	YHL038C	27.000	0.000
A_06_P3557	YHL039W	80.000	0.000
A_06_P3558	YHL040C	4.000	0.000
A_06_P3559	YHL041W	70.000	0.000
A_06_P3560	YHL042W	41.000	0.000
A_06_P3561	YHL043W	45.000	0.000
A_06_P3562	YHL044W	23.000	0.000
A_06_P3563	YHL045W	9.000	0.000
A_06_P3564	YBL108C-A	0.200	0.447
A_06_P3564	YGR294W	0.200	0.447
A_06_P3564	YHL046C	25.000	1.414
A_06_P3564	YIL176C	3.200	1.095
A_06_P3565	YHL047C	217.000	0.000
A_06_P3566	YHL048W	92.000	0.000
A_06_P3567	YFL065C	0.400	0.548
A_06_P3567	YHL049C	96.200	6.099
A_06_P3567	YPR203W	29.000	3.536
A_06_P3569	YHR001W	65.000	0.000

A_06_P3570	YHR001W-A	900.000	0.000
A_06_P3571	YHR002W	64.000	0.000
A_06_P3572	YHR003C	22.000	0.000
A_06_P3573	YHR004C	20.000	0.000
A_06_P3574	YHR005C	228.000	0.000
A_06_P3575	YHR005C-A	28.000	0.000
A_06_P3576	YHR006W	100.000	0.000
A_06_P3577	YHR007C	90.000	0.000
A_06_P3578	YHR008C	17.000	0.000
A_06_P3579	YHR009C	105.000	0.000
A_06_P3580	YHR010W	13.000	0.000
A_06_P3581	YHR011W	65.000	0.000
A_06_P3582	YHR012W	35.000	0.000
A_06_P3583	YHR013C	5.000	0.000
A_06_P3584	YHR014W	13.000	0.000
A_06_P3585	YHR015W	57.000	0.000
A_06_P3586	YHR016C	45.000	0.000
A_06_P3587	YHR017W	13.000	0.000
A_06_P3588	YHR018C	25.000	0.000
A_06_P3589	YHR019C	187.000	0.000
A_06_P3590	YHR020W	166.000	0.000
A_06_P3591	YHR021C	35.000	0.000
A_06_P3592	YHR021W-A	32.000	0.000
A_06_P3593	YHR022C	49.000	0.000
A_06_P3594	YHR023W	27.000	0.000
A_06_P3595	YHR024C	161.000	0.000
A_06_P3596	YHR025W	70.000	0.000
A_06_P3597	YHR026W	59.000	0.000
A_06_P3598	YHR027C	12.000	0.000
A_06_P3599	YHR028C	47.000	0.000
A_06_P3600	YHR029C	54.000	0.000
A_06_P3601	YHR030C	325.000	0.000
A_06_P3602	YHR031C	28.000	0.000
A_06_P3603	YHR032W	53.000	0.000
A_06_P3604	YHR033W	13.000	0.000
A_06_P3605	YHR034C	7.000	0.000
A_06_P3606	YHR035W	106.000	0.000
A_06_P3607	YHR036W	29.000	0.000
A_06_P3608	YHR037W	51.000	0.000
A_06_P3609	YHR038W	21.000	0.000
A_06_P3610	YHR039C	26.000	0.000
A_06_P3611	YHR039C-A	29.000	0.000
A_06_P3612	YHR040W	136.000	0.000
A_06_P3613	YHR041C	38.000	0.000
A_06_P3614	YHR042W	25.000	0.000
A_06_P3615	YHR043C	54.000	0.000
A_06_P3616	YHR044C	51.000	0.000
A_06_P3617	YHR045W	33.000	0.000
A_06_P3618	YHR046C	19.000	0.000
A_06_P3619	YHR047C	70.000	0.000
A_06_P3620	YHR048W	172.000	0.000
A_06_P3621	YHR049C-A	34.000	0.000
A_06_P3622	YHR049W	14.000	0.000
A_06_P3623	YHR050W	198.000	0.000
A_06_P3624	YHR051W	70.000	0.000

A_06_P3625	YHR052W	18.000	0.000
A_06_P3626	YHR053C	37.000	4.743
A_06_P3626	YHR055C	23.800	3.114
A_06_P3627	YHR054C	18.200	3.114
A_06_P3627	YHR056C	6.800	1.643
A_06_P3628	YHR053C	34.000	4.743
A_06_P3628	YHR055C	25.200	3.114
A_06_P3629	YHR054C	22.800	3.114
A_06_P3629	YHR056C	8.200	1.643
A_06_P3630	YHR057C	58.000	0.000
A_06_P3631	YHR058C	9.000	0.000
A_06_P3632	YHR059W	201.000	0.000
A_06_P3633	YHR060W	14.000	0.000
A_06_P3634	YHR061C	44.000	0.000
A_06_P3635	YHR062C	33.000	0.000
A_06_P3636	YHR063C	183.000	0.000
A_06_P3637	YHR064C	129.000	0.000
A_06_P3638	YHR065C	20.000	0.000
A_06_P3639	YHR066W	39.000	0.000
A_06_P3640	YHR067W	11.000	0.000
A_06_P3641	YHR068W	42.000	0.000
A_06_P3642	YHR069C	35.000	0.000
A_06_P3643	YHR070W	71.000	0.000
A_06_P3644	YHR071W	49.000	0.000
A_06_P3645	YHR072W	5.000	0.000
A_06_P3646	YHR072W-A	18.000	0.000
A_06_P3647	YHR073W	35.000	0.000
A_06_P3648	YHR074W	285.000	0.000
A_06_P3649	YHR075C	41.000	0.000
A_06_P3650	YHR076W	284.000	0.000
A_06_P3651	YHR077C	203.000	0.000
A_06_P3652	YHR078W	54.000	0.000
A_06_P3653	YHR079C	121.000	0.000
A_06_P3654	YHR079C-A	0.200	0.447
A_06_P3655	YHR080C	13.000	0.000
A_06_P3656	YHR081W	38.000	0.000
A_06_P3657	YHR082C	88.000	0.000
A_06_P3658	YHR083W	64.000	0.000
A_06_P3659	YHR084W	17.000	0.000
A_06_P3660	YHR085W	43.000	0.000
A_06_P3661	YHR086W	48.000	0.000
A_06_P3662	YHR087W	23.000	0.000
A_06_P3663	YHR088W	6.000	0.000
A_06_P3664	YHR089C	85.000	0.000
A_06_P3665	YHR090C	4.000	0.000
A_06_P3666	YHR091C	78.000	0.000
A_06_P3667	YHR092C	16.000	0.000
A_06_P3668	YHR093W	29.000	0.000
A_06_P3669	YHR094C	96.000	0.000
A_06_P3670	YHR095W	9.000	0.000
A_06_P3671	YHR096C	96.000	0.000
A_06_P3672	YHR097C	23.000	0.000
A_06_P3673	YHR098C	10.000	0.000
A_06_P3674	YHR099W	18.000	0.000
A_06_P3675	YHR100C	31.000	0.000

A_06_P3676	YHR101C	159.000	0.000
A_06_P3677	YHR102W	34.000	0.000
A_06_P3678	YHR103W	152.000	0.000
A_06_P3679	YHR104W	4.000	0.000
A_06_P3680	YHR105W	239.000	0.000
A_06_P3681	YHR106W	5.000	0.000
A_06_P3682	YHR107C	62.000	0.000
A_06_P3683	YHR108W	172.000	0.000
A_06_P3684	YHR109W	13.000	0.000
A_06_P3685	YHR110W	18.000	0.000
A_06_P3686	YHR111W	1000.000	0.000
A_06_P3687	YHR112C	14.000	0.000
A_06_P3688	YHR113W	68.000	0.000
A_06_P3689	YHR114W	94.000	0.000
A_06_P3690	YHR115C	39.000	0.000
A_06_P3691	YHR116W	14.000	0.000
A_06_P3692	YHR117W	18.000	0.000
A_06_P3693	YHR118C	52.000	0.000
A_06_P3694	YHR119W	26.000	0.000
A_06_P3695	YHR120W	25.000	0.000
A_06_P3696	YHR121W	169.000	0.000
A_06_P3697	YHR122W	46.000	0.000
A_06_P3698	YHR123W	66.000	0.000
A_06_P3699	YHR124W	76.000	0.000
A_06_P3700	YHR125W	9.000	0.000
A_06_P3701	YHR126C	150.000	0.000
A_06_P3702	YHR127W	12.000	0.000
A_06_P3703	YHR128W	19.000	0.000
A_06_P3704	YHR129C	555.000	0.000
A_06_P3705	YHR130C	125.000	0.000
A_06_P3706	YHR131C	16.000	0.000
A_06_P3707	YHR132C	53.000	0.000
A_06_P3708	YHR132W-A	201.000	0.000
A_06_P3709	YHR133C	192.000	0.000
A_06_P3710	YHR134W	25.000	0.000
A_06_P3711	YHR135C	159.000	0.000
A_06_P3712	YHR136C	65.000	0.000
A_06_P3713	YHR137W	31.000	0.000
A_06_P3714	YHR138C	12.000	0.000
A_06_P3715	YHR139C	18.000	0.000
A_06_P3716	YHR139C-A	13.000	0.000
A_06_P3717	YHR140W	234.000	0.000
A_06_P3718	YHR141C	92.400	1.517
A_06_P3719	YHR142W	332.000	0.000
A_06_P3720	YHR143W	126.000	0.000
A_06_P3721	YHR143W-A	48.000	0.000
A_06_P3722	YHR144C	18.000	0.000
A_06_P3723	YHR145C	24.000	0.000
A_06_P3724	YHR146W	38.000	0.000
A_06_P3725	YHR147C	65.000	0.000
A_06_P3726	YHR148W	26.000	0.000
A_06_P3727	YHR149C	182.000	0.000
A_06_P3728	YHR150W	412.000	0.000
A_06_P3729	YHR151C	124.000	0.000
A_06_P3730	YHR152W	5.000	0.000

A_06_P3731	YHR153C	19.000	0.000
A_06_P3732	YHR154W	25.000	0.000
A_06_P3733	YHR155W	107.000	0.000
A_06_P3734	YHR156C	71.000	0.000
A_06_P3735	YHR157W	23.000	0.000
A_06_P3736	YHR158C	91.000	0.000
A_06_P3737	YHR159W	405.000	0.000
A_06_P3738	YHR160C	192.000	0.000
A_06_P3739	YHR161C	5.000	0.000
A_06_P3740	YHR162W	18.000	0.000
A_06_P3741	YHR163W	88.000	0.000
A_06_P3742	YHR164C	294.000	0.000
A_06_P3743	YHR165C	134.000	0.000
A_06_P3744	YHR166C	98.000	0.000
A_06_P3745	YHR167W	35.000	0.000
A_06_P3746	YHR168W	6.000	0.000
A_06_P3747	YHR169W	23.000	0.000
A_06_P3748	YHR170W	14.000	0.000
A_06_P3749	YHR171W	23.000	0.000
A_06_P3750	YHR172W	297.000	0.000
A_06_P3751	YHR173C	103.000	0.000
A_06_P3752	YHR174W	103.000	0.000
A_06_P3753	YHR175W	95.000	0.000
A_06_P3754	YHR176W	31.000	0.000
A_06_P3755	YHR177W	25.000	0.000
A_06_P3756	YHR178W	17.000	0.000
A_06_P3757	YHR179W	407.000	0.000
A_06_P3758	YHR180W	12.000	0.000
A_06_P3759	YHR181W	78.000	0.000
A_06_P3760	YHR182W	25.000	0.000
A_06_P3761	YHR183W	46.000	0.000
A_06_P3762	YHR184W	942.000	0.000
A_06_P3763	YHR185C	66.000	0.000
A_06_P3764	YHR186C	24.000	0.000
A_06_P3765	YHR187W	35.000	0.000
A_06_P3766	YHR188C	458.000	0.000
A_06_P3767	YHR189W	41.000	0.000
A_06_P3768	YHR190W	37.000	0.000
A_06_P3769	YHR191C	121.000	0.000
A_06_P3770	YHR192W	27.000	0.000
A_06_P3771	YHR193C	42.000	0.000
A_06_P3772	YHR194W	15.000	0.000
A_06_P3773	YHR195W	80.000	0.000
A_06_P3774	YHR196W	9.000	0.000
A_06_P3775	YHR197W	78.000	0.000
A_06_P3776	YHR198C	286.000	0.000
A_06_P3777	YHR199C	18.000	0.000
A_06_P3778	YHR200W	52.000	0.000
A_06_P3779	YHR201C	52.000	0.000
A_06_P3780	YHR202W	83.000	0.000
A_06_P3781	YHR203C	137.000	0.000
A_06_P3782	YHR204W	97.000	0.000
A_06_P3783	YHR205W	255.000	0.000
A_06_P3784	YHR206W	467.000	0.000
A_06_P3785	YHR207C	15.000	0.000

A_06_P3786	YHR208W	94.000	0.000
A_06_P3787	YHR209W	47.000	0.000
A_06_P3788	YHR210C	47.000	0.000
A_06_P3789	YAL065C	0.200	0.447
A_06_P3789	YHR211W	13.000	0.000
A_06_P3790	YAR060C	18.200	3.347
A_06_P3790	YHR212C	14.400	1.140
A_06_P3791	YAL063C	0.200	0.447
A_06_P3791	YAR050W	1.000	1.414
A_06_P3791	YAR062W	59.200	4.087
A_06_P3791	YHR213W	22.400	3.362
A_06_P3792	YAR066W	1.600	0.548
A_06_P3792	YHR214W	65.200	0.447
A_06_P3793	YAR068W	0.800	0.837
A_06_P3793	YHR214W-A	48.800	1.304
A_06_P3794	YAR071W	1.000	0.707
A_06_P3794	YHR215W	14.000	0.000
A_06_P3795	YHR216W	58.000	0.000
A_06_P3796	YHR217C	14.000	0.000
A_06_P3797	YBL111C	33.600	4.827
A_06_P3797	YHR218W	37.200	3.834
A_06_P3797	YNL339C	0.200	0.447
A_06_P3797	YOR396W	1.000	1.225
A_06_P3797	YPR204W	1.600	1.140
A_06_P3799	YIL001W	11.000	0.000
A_06_P3800	YIL002C	57.000	0.000
A_06_P3801	YIL003W	92.000	0.000
A_06_P3802	YIL004C	44.000	0.000
A_06_P3803	YIL005W	373.000	0.000
A_06_P3804	YIL006W	12.000	0.000
A_06_P3805	YIL007C	26.000	0.000
A_06_P3806	YIL008W	21.000	0.000
A_06_P3807	YIL009C-A	49.000	0.000
A_06_P3808	YIL009W	74.000	0.000
A_06_P3809	YIL010W	265.000	0.000
A_06_P3810	YIL011W	51.000	0.000
A_06_P3811	YIL012W	23.000	0.000
A_06_P3812	YIL013C	9.000	0.000
A_06_P3813	YIL014W	62.000	0.000
A_06_P3814	YIL014C-A	690.000	0.000
A_06_P3815	YIL015W	63.000	0.000
A_06_P3816	YIL016W	102.000	0.000
A_06_P3817	YIL017C	125.000	0.000
A_06_P3818	YIL018W	61.000	0.000
A_06_P3819	YIL019W	31.000	0.000
A_06_P3820	YIL020C	41.000	0.000
A_06_P3821	YIL021W	93.000	0.000
A_06_P3822	YIL022W	60.000	0.000
A_06_P3823	YIL023C	195.000	0.000
A_06_P3824	YIL024C	12.000	0.000
A_06_P3825	YIL025C	78.000	0.000
A_06_P3826	YIL026C	71.000	0.000
A_06_P3827	YIL027C	40.000	0.000
A_06_P3828	YIL028W	68.000	0.000
A_06_P3829	YIL029C	37.000	0.000

A_06_P3830	YIL030C	8.000	0.000
A_06_P3831	YIL030W-A	63.000	0.000
A_06_P3831	YIL031W	8.000	0.000
A_06_P3832	YIL032C	73.000	0.000
A_06_P3833	YIL033C	61.000	0.000
A_06_P3834	YIL034C	473.000	0.000
A_06_P3835	YIL035C	99.000	0.000
A_06_P3836	YIL036W	60.000	0.000
A_06_P3837	YIL037C	39.000	0.000
A_06_P3838	YIL038C	22.000	0.000
A_06_P3839	YIL039W	57.000	0.000
A_06_P3840	YIL040W	63.000	0.000
A_06_P3841	YIL041W	28.000	0.000
A_06_P3842	YIL042C	11.000	0.000
A_06_P3843	YIL043C	381.000	0.000
A_06_P3844	YIL044C	19.000	0.000
A_06_P3845	YIL045W	26.000	0.000
A_06_P3846	YIL046W	7.000	0.000
A_06_P3847	YIL047C	39.000	0.000
A_06_P3847	YIL047C-A	17.000	0.000
A_06_P3848	YIL048W	42.000	0.000
A_06_P3849	YIL049W	177.000	0.000
A_06_P3850	YIL050W	8.000	0.000
A_06_P3851	YIL051C	117.000	0.000
A_06_P3852	YIL052C	23.000	0.000
A_06_P3853	YIL053W	167.000	0.000
A_06_P3854	YIL054W	51.000	0.000
A_06_P3855	YIL055C	63.000	0.000
A_06_P3856	YIL056W	25.000	0.000
A_06_P3857	YIL057C	26.000	0.000
A_06_P3858	YIL058W	85.000	0.000
A_06_P3859	YIL059C	93.000	0.000
A_06_P3860	YIL060W	112.000	0.000
A_06_P3861	YIL061C	30.000	0.000
A_06_P3862	YIL062C	22.000	0.000
A_06_P3863	YIL063C	141.000	0.000
A_06_P3864	YIL064W	141.000	0.000
A_06_P3865	YIL065C	53.000	0.000
A_06_P3866	YIL066C	130.000	0.000
A_06_P3867	YIL067C	113.000	0.000
A_06_P3868	YIL068C	109.000	0.000
A_06_P3869	YIL069C	11.000	0.000
A_06_P3870	YIL070C	248.000	0.000
A_06_P3871	YIL071C	14.000	0.000
A_06_P3872	YIL072W	19.000	0.000
A_06_P3873	YIL073C	23.000	0.000
A_06_P3874	YIL074C	65.000	0.000
A_06_P3875	YIL075C	298.000	0.000
A_06_P3876	YIL076W	418.000	0.000
A_06_P3877	YIL077C	353.000	0.000
A_06_P3878	YIL078W	94.000	0.000
A_06_P3879	YIL079C	57.000	0.000
A_06_P3880	YGR109W-A	9.000	0.000
A_06_P3880	YGR109W-B	30.000	4.950
A_06_P3880	YIL082W	12.000	0.000

A_06_P3880	YIL082W-A	0.200	0.447
A_06_P3881	YGR109W-B	16.000	4.950
A_06_P3881	YIL080W	1.000	0.000
A_06_P3881	YIL082W-A	25.800	0.447
A_06_P3882	YIL083C	51.000	0.000
A_06_P3883	YIL084C	19.000	0.000
A_06_P3884	YIL085C	121.000	0.000
A_06_P3885	YIL086C	129.000	0.000
A_06_P3886	YIL087C	49.000	0.000
A_06_P3887	YIL088C	127.000	0.000
A_06_P3888	YIL089W	12.000	0.000
A_06_P3889	YIL090W	122.000	0.000
A_06_P3890	YIL091C	31.000	0.000
A_06_P3891	YIL092W	24.000	0.000
A_06_P3892	YIL093C	355.000	0.000
A_06_P3893	YIL094C	29.000	0.000
A_06_P3894	YIL095W	9.000	0.000
A_06_P3895	YIL096C	11.000	0.000
A_06_P3896	YIL097W	12.000	0.000
A_06_P3897	YIL098C	298.000	0.000
A_06_P3898	YIL099W	66.000	0.000
A_06_P3899	YIL100W	49.000	0.000
A_06_P3900	YIL101C	5.000	0.000
A_06_P3901	YIL102C	114.000	0.000
A_06_P3902	YIL103W	48.000	0.000
A_06_P3903	YIL104C	278.000	0.000
A_06_P3904	YIL105C	26.000	0.000
A_06_P3905	YIL106W	172.000	0.000
A_06_P3906	YIL107C	75.000	0.000
A_06_P3907	YIL108W	81.000	0.000
A_06_P3908	YIL109C	6.000	0.000
A_06_P3909	YIL110W	157.000	0.000
A_06_P3910	YIL111W	66.000	0.000
A_06_P3911	YIL112W	71.000	0.000
A_06_P3912	YIL113W	38.000	0.000
A_06_P3913	YIL114C	103.000	0.000
A_06_P3914	YIL115C	29.000	0.000
A_06_P3915	YIL116W	105.000	0.000
A_06_P3916	YIL117C	14.000	0.000
A_06_P3917	YIL118W	72.000	0.000
A_06_P3918	YIL119C	162.000	0.000
A_06_P3919	YIL120W	20.000	0.000
A_06_P3920	YIL121W	41.000	0.000
A_06_P3921	YIL122W	12.000	0.000
A_06_P3922	YIL123W	217.000	0.000
A_06_P3923	YIL124W	92.000	0.000
A_06_P3924	YIL125W	39.000	0.000
A_06_P3925	YIL126W	82.000	0.000
A_06_P3926	YIL127C	5.000	0.000
A_06_P3927	YIL128W	215.000	0.000
A_06_P3928	YIL129C	189.000	0.000
A_06_P3929	YIL130W	30.000	0.000
A_06_P3930	YIL131C	18.000	0.000
A_06_P3931	YIL132C	29.000	0.000
A_06_P3932	YIL133C	32.000	0.000

A_06_P3933	YIL134W	30.000	0.000
A_06_P3934	YIL135C	239.000	0.000
A_06_P3935	YIL136W	100.000	0.000
A_06_P3936	YIL137C	694.000	0.000
A_06_P3937	YIL138C	19.000	0.000
A_06_P3938	YIL139C	87.000	0.000
A_06_P3939	YIL140W	221.000	0.000
A_06_P3940	YIL141W	7.000	0.000
A_06_P3941	YIL142W	108.000	0.000
A_06_P3942	YIL143C	546.000	0.000
A_06_P3943	YIL144W	183.000	0.000
A_06_P3944	YIL145C	143.000	0.000
A_06_P3945	YIL146C	62.000	0.000
A_06_P3946	YIL147C	38.000	0.000
A_06_P3947	YIL148W	8.000	0.000
A_06_P3948	YIL149C	52.000	0.000
A_06_P3949	YIL150C	76.000	0.000
A_06_P3950	YIL151C	2.000	0.000
A_06_P3951	YIL152W	154.000	0.000
A_06_P3952	YIL153W	5.000	0.000
A_06_P3953	YIL154C	50.000	0.000
A_06_P3954	YIL155C	112.000	0.000
A_06_P3955	YIL156W	55.000	0.000
A_06_P3956	YIL157C	51.000	0.000
A_06_P3957	YIL158W	94.000	0.000
A_06_P3958	YIL159W	27.000	0.000
A_06_P3959	YIL160C	136.000	0.000
A_06_P3960	YIL161W	15.000	0.000
A_06_P3961	YIL162W	174.000	0.000
A_06_P3962	YIL163C	10.000	0.000
A_06_P3963	YIL164C	28.000	0.000
A_06_P3964	YIL165C	40.000	0.000
A_06_P3965	YIL166C	23.000	0.000
A_06_P3966	YIL167W	27.000	0.000
A_06_P3967	YIL168W	51.000	0.000
A_06_P3968	YIL169C	75.000	0.000
A_06_P3968	YOL155C	2.000	0.707
A_06_P3969	YIL170W	26.000	0.000
A_06_P3970	YIL171W	362.000	0.000
A_06_P3971	YIL172C	43.600	2.608
A_06_P3971	YJL221C	21.400	3.912
A_06_P3971	YOL157C	10.800	2.775
A_06_P3972	YIL173W	17.200	2.387
A_06_P3972	YJL222W	1.400	1.140
A_06_P3973	YAL067W-A	44.000	0.000
A_06_P3973	YIL174W	60.000	0.000
A_06_P3973	YJL222W-A	12.000	0.000
A_06_P3973	YNR075C-A	8.000	0.000
A_06_P3974	YIL175W	16.000	0.000
A_06_P3975	YAL068C	10.400	1.342
A_06_P3975	YBL108C-A	19.400	1.949
A_06_P3975	YBR301W	3.200	1.304
A_06_P3975	YDR542W	0.200	0.447
A_06_P3975	YEL049W	6.000	3.536
A_06_P3975	YGL261C	4.400	0.548

A_06_P3975	YGR294W	12.600	3.050
A_06_P3975	YHL046C	0.600	0.548
A_06_P3975	YIL176C	266.400	16.041
A_06_P3975	YJL223C	8.800	2.588
A_06_P3975	YLL064C	0.400	0.548
A_06_P3977	YIR001C	32.000	0.000
A_06_P3978	YIR002C	174.000	0.000
A_06_P3979	YIR003W	55.000	0.000
A_06_P3980	YIR004W	35.000	0.000
A_06_P3981	YIR005W	15.000	0.000
A_06_P3982	YIR006C	188.000	0.000
A_06_P3983	YIR007W	23.000	0.000
A_06_P3984	YIR008C	104.000	0.000
A_06_P3985	YIR009W	49.000	0.000
A_06_P3986	YIR010W	36.000	0.000
A_06_P3987	YIR011C	86.000	0.000
A_06_P3988	YIR012W	59.000	0.000
A_06_P3989	YIR013C	13.000	0.000
A_06_P3990	YIR014W	27.000	0.000
A_06_P3991	YIR015W	173.000	0.000
A_06_P3992	YIR016W	80.000	0.000
A_06_P3993	YIR017C	98.000	0.000
A_06_P3994	YIR018W	33.000	0.000
A_06_P3995	YIR019C	5.000	0.000
A_06_P3996	YIR020C	7.000	0.000
A_06_P3997	YIR020W-A	30.000	0.000
A_06_P3998	YIR021W	322.000	0.000
A_06_P3999	YIR022W	119.000	0.000
A_06_P4000	YIR023W	27.000	0.000
A_06_P4001	YIR024C	206.000	0.000
A_06_P4002	YIR025W	70.000	0.000
A_06_P4003	YIR026C	53.000	0.000
A_06_P4004	YIR027C	33.000	0.000
A_06_P4005	YIR028W	26.000	0.000
A_06_P4006	YIR029W	41.000	0.000
A_06_P4007	YIR030C	112.000	0.000
A_06_P4008	YIR031C	99.000	0.000
A_06_P4009	YIR032C	6.000	0.000
A_06_P4010	YIR033W	7.000	0.000
A_06_P4011	YIR034C	34.000	0.000
A_06_P4012	YIR035C	341.000	0.000
A_06_P4013	YIR036C	19.000	0.000
A_06_P4014	YIR037W	45.000	0.000
A_06_P4015	YIR038C	5.000	0.000
A_06_P4016	YIR039C	381.000	0.000
A_06_P4017	YGL260W	0.400	0.894
A_06_P4017	YIR040C	12.800	0.447
A_06_P4018	YIR041W	37.600	0.548
A_06_P4019	YIR042C	56.000	0.000
A_06_P4020	YIR043C	27.000	0.000
A_06_P4021	YIR044C	37.000	0.000
A_06_P4022	YJL001W	36.000	0.000
A_06_P4023	YJL002C	38.000	0.000
A_06_P4024	YJL003W	692.000	0.000
A_06_P4025	YJL004C	48.000	0.000

A_06_P4026	YJL005W	4.000	0.000
A_06_P4027	YJL006C	26.000	0.000
A_06_P4028	YJL007C	217.000	0.000
A_06_P4029	YJL008C	152.000	0.000
A_06_P4030	YJL009W	66.000	0.000
A_06_P4030	YJL181W	0.200	0.447
A_06_P4031	YJL010C	13.000	0.000
A_06_P4032	YJL011C	4.000	0.000
A_06_P4034	YJL012C	466.000	0.000
A_06_P4035	YJL013C	22.000	0.000
A_06_P4036	YJL014W	35.000	0.000
A_06_P4037	YJL015C	91.000	0.000
A_06_P4038	YJL016W	47.000	1.225
A_06_P4039	YJL016W	7.000	1.225
A_06_P4040	YJL019W	23.600	0.894
A_06_P4041	YJL019W	2.400	0.894
A_06_P4042	YJL020C	32.000	0.000
A_06_P4043	YJL022W	122.000	0.000
A_06_P4044	YJL023C	89.000	0.000
A_06_P4045	YJL024C	7.000	0.000
A_06_P4046	YJL025W	28.000	0.000
A_06_P4047	YJL026W	397.000	0.000
A_06_P4048	YJL027C	201.000	0.000
A_06_P4049	YJL028W	19.000	0.000
A_06_P4050	YJL029C	149.000	0.000
A_06_P4051	YJL030W	95.000	0.000
A_06_P4052	YJL031C	63.000	0.000
A_06_P4053	YJL032W	13.000	0.000
A_06_P4054	YJL033W	14.000	0.000
A_06_P4055	YJL034W	17.000	0.000
A_06_P4056	YJL035C	81.000	0.000
A_06_P4057	YJL036W	84.000	0.000
A_06_P4058	YJL037W	17.000	0.000
A_06_P4059	YJL038C	1000.000	0.000
A_06_P4060	YJL039C	45.000	0.000
A_06_P4061	YJL041W	29.000	0.000
A_06_P4062	YJL042W	97.000	0.000
A_06_P4063	YJL043W	28.000	0.000
A_06_P4064	YJL044C	80.000	0.000
A_06_P4065	YJL045W	172.000	0.000
A_06_P4066	YJL046W	132.000	0.000
A_06_P4067	YJL047C	71.000	0.000
A_06_P4068	YJL048C	7.000	0.000
A_06_P4069	YJL049W	118.000	0.000
A_06_P4070	YJL050W	407.000	0.000
A_06_P4071	YJL051W	28.000	0.000
A_06_P4072	YJL052C-A	294.000	0.000
A_06_P4073	YJL052W	46.000	0.000
A_06_P4074	YJL053W	19.000	0.000
A_06_P4075	YJL054W	126.000	0.000
A_06_P4076	YJL055W	11.000	0.000
A_06_P4077	YJL056C	18.000	0.000
A_06_P4078	YJL057C	48.000	0.000
A_06_P4079	YJL058C	199.000	0.000
A_06_P4080	YJL059W	58.000	0.000

A_06_P4081	YJL060W	22.000	0.000
A_06_P4082	YJL061W	17.000	0.000
A_06_P4083	YJL062W	188.000	0.000
A_06_P4084	YJL062W-A	32.000	0.000
A_06_P4085	YJL063C	12.000	0.000
A_06_P4086	YJL064W	54.000	0.000
A_06_P4087	YJL065C	46.000	0.000
A_06_P4088	YJL066C	12.000	0.000
A_06_P4089	YJL067W	20.000	0.000
A_06_P4090	YJL068C	48.000	0.000
A_06_P4091	YJL069C	80.000	0.000
A_06_P4092	YJL070C	82.000	0.000
A_06_P4093	YJL071W	68.000	0.000
A_06_P4094	YJL072C	82.000	0.000
A_06_P4095	YJL073W	15.000	0.000
A_06_P4096	YJL074C	7.000	0.000
A_06_P4097	YJL075C	74.000	0.000
A_06_P4098	YJL076W	2.000	0.000
A_06_P4099	YJL077C	10.000	0.000
A_06_P4100	YJL078C	90.000	0.000
A_06_P4101	YJL079C	18.000	0.000
A_06_P4102	YJL080C	67.000	0.000
A_06_P4103	YJL081C	32.000	0.000
A_06_P4104	YJL082W	32.000	0.000
A_06_P4105	YJL083W	7.000	0.000
A_06_P4106	YJL084C	5.000	0.000
A_06_P4107	YJL085W	14.000	0.000
A_06_P4108	YJL086C	9.000	0.000
A_06_P4109	YJL087C	4.000	0.000
A_06_P4110	YJL088W	76.000	0.000
A_06_P4111	YJL089W	29.000	0.000
A_06_P4112	YJL090C	29.000	0.000
A_06_P4113	YJL091C	73.000	0.000
A_06_P4114	YJL092W	67.000	0.000
A_06_P4115	YJL093C	59.000	0.000
A_06_P4116	YJL094C	126.000	0.000
A_06_P4117	YJL095W	20.000	0.000
A_06_P4118	YJL096W	48.000	0.000
A_06_P4119	YJL097W	79.000	0.000
A_06_P4120	YJL098W	122.000	0.000
A_06_P4121	YJL099W	11.000	0.000
A_06_P4122	YJL100W	139.000	0.000
A_06_P4123	YJL101C	145.000	0.000
A_06_P4124	YJL102W	15.000	0.000
A_06_P4125	YJL103C	192.000	0.000
A_06_P4126	YJL104W	68.000	0.000
A_06_P4127	YJL105W	112.000	0.000
A_06_P4128	YJL106W	5.000	0.000
A_06_P4129	YJL107C	191.000	0.000
A_06_P4130	YJL108C	90.000	0.000
A_06_P4131	YJL109C	81.000	0.000
A_06_P4132	YJL110C	205.000	0.000
A_06_P4133	YJL111W	68.000	0.000
A_06_P4134	YJL112W	29.000	0.000
A_06_P4135	YJL115W	32.000	0.000

A_06_P4136	YJL116C	109.000	0.000
A_06_P4137	YJL117W	127.000	0.000
A_06_P4138	YJL118W	82.000	0.000
A_06_P4139	YJL119C	218.000	0.000
A_06_P4140	YJL120W	60.000	0.000
A_06_P4141	YJL121C	12.000	0.000
A_06_P4142	YJL122W	95.000	0.000
A_06_P4143	YJL123C	104.000	0.000
A_06_P4144	YJL124C	152.000	0.000
A_06_P4145	YJL125C	135.000	0.000
A_06_P4146	YJL126W	94.000	0.000
A_06_P4147	YJL127C	42.000	0.000
A_06_P4148	YJL127W-A	159.000	0.000
A_06_P4149	YJL128C	179.000	0.000
A_06_P4150	YJL129C	48.000	0.000
A_06_P4151	YJL130C	117.000	0.000
A_06_P4152	YJL131C	27.000	0.000
A_06_P4153	YJL132W	172.000	0.000
A_06_P4154	YJL133W	13.000	0.000
A_06_P4155	YJL134W	32.000	0.000
A_06_P4156	YJL135W	65.000	0.000
A_06_P4157	YJL136C	13.000	0.000
A_06_P4158	YJL137C	166.000	0.000
A_06_P4159	YJL138C	19.000	0.000
A_06_P4160	YJL139C	11.000	0.000
A_06_P4161	YJL140W	59.000	0.000
A_06_P4162	YJL141C	7.000	0.000
A_06_P4163	YJL142C	126.000	0.000
A_06_P4164	YJL143W	43.000	0.000
A_06_P4165	YJL144W	38.000	0.000
A_06_P4166	YJL145W	332.000	0.000
A_06_P4167	YJL146W	266.000	0.000
A_06_P4168	YJL147C	29.000	0.000
A_06_P4169	YJL148W	32.000	0.000
A_06_P4170	YJL149W	12.000	0.000
A_06_P4171	YJL150W	68.000	0.000
A_06_P4172	YJL151C	8.000	0.000
A_06_P4173	YJL152W	9.000	0.000
A_06_P4174	YJL153C	41.000	0.000
A_06_P4175	YJL154C	7.000	0.000
A_06_P4176	YJL155C	67.000	0.000
A_06_P4177	YJL156C	12.000	0.000
A_06_P4178	YJL156W-A	35.000	0.000
A_06_P4179	YJL157C	141.000	0.000
A_06_P4180	YJL158C	21.000	0.000
A_06_P4181	YJL159W	173.000	0.000
A_06_P4182	YJL160C	62.000	0.000
A_06_P4183	YJL161W	578.000	0.000
A_06_P4184	YJL162C	130.000	0.000
A_06_P4185	YJL163C	6.000	0.000
A_06_P4186	YJL164C	163.000	0.000
A_06_P4187	YJL165C	79.000	0.000
A_06_P4188	YJL166W	116.000	0.000
A_06_P4189	YJL167W	10.000	0.000
A_06_P4190	YJL168C	17.000	0.000

A_06_P4191	YJL169W	91.000	0.000
A_06_P4192	YJL170C	3.000	0.000
A_06_P4193	YJL171C	153.000	0.000
A_06_P4194	YJL172W	80.000	0.000
A_06_P4195	YJL173C	40.000	0.000
A_06_P4196	YJL174W	12.000	0.000
A_06_P4197	YJL175W	48.000	0.000
A_06_P4198	YJL176C	42.000	0.000
A_06_P4199	YJL177W	107.000	0.000
A_06_P4200	YJL178C	1.000	0.000
A_06_P4201	YJL179W	40.000	0.000
A_06_P4202	YJL180C	36.000	0.000
A_06_P4203	YJL181W	1000.000	0.000
A_06_P4204	YJL182C	271.000	0.000
A_06_P4205	YJL183W	764.000	0.000
A_06_P4206	YJL184W	17.000	0.000
A_06_P4207	YJL185C	9.000	0.000
A_06_P4208	YJL186W	24.000	0.000
A_06_P4209	YJL187C	26.000	0.000
A_06_P4210	YJL188C	17.000	0.000
A_06_P4211	YJL189W	306.000	0.000
A_06_P4212	YJL190C	52.000	0.000
A_06_P4213	YJL191W	55.000	0.000
A_06_P4214	YJL192C	42.000	0.000
A_06_P4215	YJL193W	39.000	0.000
A_06_P4216	YJL194W	42.000	0.000
A_06_P4217	YJL195C	18.000	0.000
A_06_P4218	YJL196C	62.000	0.000
A_06_P4219	YJL197W	830.000	0.000
A_06_P4220	YJL198W	24.000	0.000
A_06_P4221	YJL199C	45.000	0.000
A_06_P4222	YJL200C	103.000	0.000
A_06_P4223	YJL201W	157.000	0.000
A_06_P4224	YJL202C	323.000	0.000
A_06_P4225	YJL203W	36.000	0.000
A_06_P4226	YJL204C	9.000	0.000
A_06_P4227	YJL205C	516.000	0.000
A_06_P4228	YJL206C	32.000	0.000
A_06_P4229	YJL207C	12.000	0.000
A_06_P4230	YJL208C	7.000	0.000
A_06_P4231	YJL209W	46.000	0.000
A_06_P4232	YJL210W	19.000	0.000
A_06_P4233	YJL211C	171.000	0.000
A_06_P4234	YJL212C	273.000	0.000
A_06_P4235	YJL213W	101.000	0.000
A_06_P4236	YJL214W	13.000	0.000
A_06_P4237	YJL215C	35.000	0.000
A_06_P4238	YJL216C	29.000	0.000
A_06_P4239	YJL217W	35.000	0.000
A_06_P4240	YJL218W	56.000	0.000
A_06_P4241	YJL219W	66.000	0.000
A_06_P4242	YIL171W-A	275.000	0.000
A_06_P4242	YJL220W	307.000	0.000
A_06_P4243	YIL172C	39.400	2.608
A_06_P4243	YJL221C	19.600	3.912

A_06_P4243	YOL157C	8.200	2.775
A_06_P4244	YIL173W	2.800	2.387
A_06_P4244	YJL222W	242.600	1.140
A_06_P4245	YAL068C	9.800	3.194
A_06_P4245	YBL108C-A	21.200	3.033
A_06_P4245	YBR301W	5.400	1.949
A_06_P4245	YDR542W	0.200	0.447
A_06_P4245	YEL049W	6.800	2.683
A_06_P4245	YGL261C	4.200	1.304
A_06_P4245	YGR294W	16.000	2.236
A_06_P4245	YHL046C	0.200	0.447
A_06_P4245	YIL176C	258.000	12.470
A_06_P4245	YJL223C	6.800	3.033
A_06_P4245	YLL064C	0.200	0.447
A_06_P4247	YJR001W	126.000	0.000
A_06_P4248	YJR002W	227.000	0.000
A_06_P4249	YJR003C	37.000	0.000
A_06_P4250	YJR004C	53.000	0.000
A_06_P4251	YJR005W	8.000	0.000
A_06_P4252	YJR006W	117.000	0.000
A_06_P4253	YJR007W	70.000	0.000
A_06_P4254	YJR008W	130.000	0.000
A_06_P4255	YJR009C	29.000	0.000
A_06_P4256	YJR010C-A	165.000	0.000
A_06_P4257	YJR010W	13.000	0.000
A_06_P4258	YJR011C	145.000	0.000
A_06_P4259	YJR012C	251.000	0.000
A_06_P4260	YJR013W	13.000	0.000
A_06_P4261	YJR014W	24.000	0.000
A_06_P4262	YJR015W	45.000	0.000
A_06_P4263	YJR016C	92.000	0.000
A_06_P4264	YJR017C	65.000	0.000
A_06_P4265	YJR018W	139.000	0.000
A_06_P4266	YJR019C	64.000	0.000
A_06_P4267	YJR020W	54.000	0.000
A_06_P4268	YJR021C	93.000	0.000
A_06_P4269	YJR022W	355.000	0.000
A_06_P4270	YJR023C	70.000	0.000
A_06_P4271	YJR024C	38.000	0.000
A_06_P4272	YJR025C	39.000	0.000
A_06_P4273	YJR030C	233.000	0.000
A_06_P4274	YJR031C	51.000	0.000
A_06_P4275	YJR032W	45.000	0.000
A_06_P4276	YJR033C	78.000	0.000
A_06_P4277	YJR034W	211.000	0.000
A_06_P4278	YJR035W	45.000	0.000
A_06_P4279	YJR036C	151.000	0.000
A_06_P4280	YJR037W	37.000	0.000
A_06_P4281	YJR038C	128.000	0.000
A_06_P4282	YJR039W	9.000	0.000
A_06_P4283	YJR040W	159.000	0.000
A_06_P4284	YJR041C	130.000	0.000
A_06_P4285	YJR042W	59.000	0.000
A_06_P4286	YJR043C	114.000	0.000
A_06_P4287	YJR044C	103.000	0.000

A_06_P4288	YJR045C	39.000	0.000
A_06_P4289	YJR046W	39.000	0.000
A_06_P4290	YJR047C	75.000	0.000
A_06_P4291	YJR048W	249.000	0.000
A_06_P4292	YJR049C	255.000	0.000
A_06_P4293	YJR050W	53.000	0.000
A_06_P4294	YJR051W	17.000	0.000
A_06_P4295	YJR052W	44.000	0.000
A_06_P4296	YJR053W	61.000	0.000
A_06_P4297	YJR054W	105.000	0.000
A_06_P4298	YJR055W	293.000	0.000
A_06_P4299	YJR056C	45.000	0.000
A_06_P4300	YJR057W	117.000	0.000
A_06_P4301	YJR058C	18.000	0.000
A_06_P4302	YJR059W	52.000	0.000
A_06_P4303	YJR060W	84.000	0.000
A_06_P4304	YJR061W	53.000	0.000
A_06_P4305	YJR062C	32.000	0.000
A_06_P4306	YJR063W	112.000	0.000
A_06_P4307	YJR064W	95.000	0.000
A_06_P4308	YJR065C	10.000	0.000
A_06_P4309	YJR066W	117.000	0.000
A_06_P4310	YJR067C	17.000	0.000
A_06_P4311	YJR068W	27.000	0.000
A_06_P4312	YJR069C	24.000	0.000
A_06_P4313	YJR070C	70.000	0.000
A_06_P4314	YJR071W	499.000	0.000
A_06_P4315	YJR072C	32.000	0.000
A_06_P4316	YJR073C	26.000	0.000
A_06_P4317	YJR074W	109.000	0.000
A_06_P4318	YJR075W	45.000	0.000
A_06_P4319	YJR076C	47.000	0.000
A_06_P4320	YJR077C	51.000	0.000
A_06_P4321	YJR078W	64.000	0.000
A_06_P4322	YJR079W	34.000	0.000
A_06_P4323	YJR080C	45.000	0.000
A_06_P4324	YJR082C	30.000	0.000
A_06_P4325	YJR083C	35.000	0.000
A_06_P4326	YJR084W	224.000	0.000
A_06_P4327	YJR085C	50.000	0.000
A_06_P4328	YJR086W	16.000	0.000
A_06_P4329	YJR087W	105.000	0.000
A_06_P4330	YJR088C	199.000	0.000
A_06_P4331	YJR089W	190.000	0.000
A_06_P4332	YJR090C	11.000	0.000
A_06_P4333	YJR091C	20.000	0.000
A_06_P4334	YJR092W	140.000	0.000
A_06_P4335	YJR093C	116.000	0.000
A_06_P4336	YJR094C	21.000	0.000
A_06_P4337	YJR094W-A	9.000	0.000
A_06_P4338	YJR095W	15.000	0.000
A_06_P4339	YJR096W	67.000	0.000
A_06_P4340	YJR097W	54.000	0.000
A_06_P4341	YJR098C	55.000	0.000
A_06_P4342	YJR099W	75.000	0.000

A_06_P4343	YJR100C	38.000	0.000
A_06_P4344	YJR101W	14.000	0.000
A_06_P4345	YJR102C	27.000	0.000
A_06_P4346	YJR103W	11.000	0.000
A_06_P4347	YJR104C	2.000	0.000
A_06_P4348	YJR105W	137.000	0.000
A_06_P4349	YJR106W	8.000	0.000
A_06_P4350	YJR107W	30.000	0.000
A_06_P4351	YJR108W	56.000	0.000
A_06_P4352	YJR109C	47.000	0.000
A_06_P4353	YJR110W	198.000	0.000
A_06_P4354	YJR111C	3.000	0.000
A_06_P4355	YJR112W	126.000	0.000
A_06_P4356	YJR113C	7.000	0.000
A_06_P4357	YJR114W	8.000	0.000
A_06_P4358	YJR115W	19.000	0.000
A_06_P4359	YJR116W	124.000	0.000
A_06_P4360	YJR117W	65.000	0.000
A_06_P4361	YJR118C	229.000	0.000
A_06_P4362	YJR119C	236.000	0.000
A_06_P4363	YJR120W	8.000	0.000
A_06_P4364	YJR121W	79.000	0.000
A_06_P4365	YJR122W	17.000	0.000
A_06_P4366	YJR123W	82.000	0.000
A_06_P4367	YJR124C	28.000	0.000
A_06_P4368	YJR125C	42.000	0.000
A_06_P4369	YJR126C	81.000	0.000
A_06_P4370	YJR127C	61.000	0.000
A_06_P4371	YJR128W	119.000	0.000
A_06_P4372	YJR129C	122.000	0.000
A_06_P4373	YJR130C	33.000	0.000
A_06_P4374	YJR131W	109.000	0.000
A_06_P4375	YJR132W	65.000	0.000
A_06_P4376	YJR133W	43.000	0.000
A_06_P4377	YJR134C	34.000	0.000
A_06_P4378	YJR135C	75.000	0.000
A_06_P4379	YJR135W-A	12.000	0.000
A_06_P4380	YJR136C	40.000	0.000
A_06_P4381	YJR137C	88.000	0.000
A_06_P4382	YJR138W	17.000	0.000
A_06_P4383	YJR139C	61.000	0.000
A_06_P4384	YJR140C	22.000	0.000
A_06_P4385	YJR141W	60.000	0.000
A_06_P4386	YJR142W	101.000	0.000
A_06_P4387	YJR143C	13.000	0.000
A_06_P4388	YJR144W	63.000	0.000
A_06_P4389	YJR145C	47.000	0.000
A_06_P4390	YJR146W	64.000	0.000
A_06_P4391	YJR147W	19.000	0.000
A_06_P4392	YJR148W	17.000	0.000
A_06_P4393	YJR149W	270.000	0.000
A_06_P4394	YJR150C	1000.000	0.000
A_06_P4395	YJR151C	168.000	0.000
A_06_P4396	YJR152W	391.000	0.000
A_06_P4397	YJR153W	8.000	0.000

A_06_P4398	YJR154W	106.000	0.000
A_06_P4399	YJR155W	12.000	0.000
A_06_P4400	YFL058W	12.400	3.362
A_06_P4400	YJR156C	7.000	0.000
A_06_P4400	YNL332W	9.000	3.000
A_06_P4401	YJR157W	25.000	0.000
A_06_P4402	YDL245C	2.600	0.894
A_06_P4402	YJR158W	88.600	1.140
A_06_P4403	YDL246C	2.400	1.517
A_06_P4403	YJR159W	118.000	2.646
A_06_P4404	YDL247W	67.200	2.490
A_06_P4404	YJR160C	2.000	0.000
A_06_P4405	YDL248W	3.200	2.168
A_06_P4405	YFL062W	0.200	0.447
A_06_P4405	YJR161C	14.000	0.000
A_06_P4405	YML132W	0.200	0.447
A_06_P4405	YNL336W	0.400	0.548
A_06_P4406	YER188C-A	0.200	0.447
A_06_P4406	YJR162C	681.400	11.845
A_06_P4407	YKL001C	43.000	0.000
A_06_P4408	YKL002W	98.000	0.000
A_06_P4409	YKL003C	12.000	0.000
A_06_P4410	YKL004W	170.000	0.000
A_06_P4411	YKL005C	88.000	0.000
A_06_P4412	YKL006C-A	79.000	0.000
A_06_P4413	YKL006W	17.000	0.000
A_06_P4414	YKL007W	292.000	0.000
A_06_P4415	YKL008C	2.000	0.000
A_06_P4416	YKL009W	37.000	0.000
A_06_P4417	YKL010C	175.000	0.000
A_06_P4418	YKL011C	16.000	0.000
A_06_P4419	YKL012W	17.000	0.000
A_06_P4420	YKL013C	31.000	0.000
A_06_P4421	YKL014C	3.000	0.000
A_06_P4422	YKL015W	97.000	0.000
A_06_P4423	YKL016C	71.000	0.000
A_06_P4424	YKL017C	23.000	0.000
A_06_P4425	YKL018C-A	21.000	0.000
A_06_P4426	YKL018W	61.000	0.000
A_06_P4427	YKL019W	48.000	0.000
A_06_P4428	YKL020C	101.000	0.000
A_06_P4429	YKL021C	35.000	0.000
A_06_P4430	YKL022C	4.000	0.000
A_06_P4431	YKL023W	8.000	0.000
A_06_P4432	YKL024C	20.000	0.000
A_06_P4433	YKL025C	32.000	0.000
A_06_P4434	YKL026C	4.000	0.000
A_06_P4435	YKL027W	19.000	0.000
A_06_P4436	YKL028W	3.000	0.000
A_06_P4437	YKL029C	194.000	0.000
A_06_P4438	YKL030W	7.000	0.000
A_06_P4439	YKL031W	85.000	0.000
A_06_P4440	YKL032C	25.000	0.000
A_06_P4441	YKL033W	57.000	0.000
A_06_P4442	YKL033W-A	14.000	0.000

A_06_P4443	YKL034W	23.000	0.000
A_06_P4444	YKL035W	41.000	0.000
A_06_P4445	YKL036C	149.000	0.000
A_06_P4446	YKL037W	72.000	0.000
A_06_P4447	YKL038W	290.000	0.000
A_06_P4448	YKL039W	81.000	0.000
A_06_P4449	YKL040C	8.000	0.000
A_06_P4450	YKL041W	52.000	0.000
A_06_P4451	YKL042W	12.000	0.000
A_06_P4452	YKL043W	100.000	0.000
A_06_P4453	YKL044W	108.000	0.000
A_06_P4454	YKL045W	218.000	0.000
A_06_P4455	YKL046C	230.000	0.000
A_06_P4456	YKL047W	28.000	0.000
A_06_P4457	YKL048C	20.000	0.000
A_06_P4458	YKL049C	30.000	0.000
A_06_P4459	YKL050C	21.000	0.000
A_06_P4460	YKL051W	13.000	0.000
A_06_P4461	YKL052C	12.000	0.000
A_06_P4462	YKL053C-A	72.000	0.000
A_06_P4463	YKL053W	288.000	0.000
A_06_P4464	YKL054C	215.000	0.000
A_06_P4465	YKL055C	24.000	0.000
A_06_P4466	YKL056C	407.000	0.000
A_06_P4467	YKL057C	329.000	0.000
A_06_P4468	YKL058W	20.000	0.000
A_06_P4469	YKL059C	34.000	0.000
A_06_P4470	YKL060C	271.000	0.000
A_06_P4471	YKL061W	40.000	0.000
A_06_P4472	YKL062W	130.000	0.000
A_06_P4473	YKL063C	21.000	0.000
A_06_P4474	YKL064W	13.000	0.000
A_06_P4475	YKL065C	2.000	0.000
A_06_P4476	YKL066W	112.000	0.000
A_06_P4477	YKL067W	8.000	0.000
A_06_P4478	YKL068W	122.000	0.000
A_06_P4479	YKL069W	360.000	0.000
A_06_P4480	YKL070W	97.000	0.000
A_06_P4481	YKL071W	9.000	0.000
A_06_P4482	YKL072W	31.000	0.000
A_06_P4483	YKL073W	193.000	0.000
A_06_P4484	YKL074C	8.000	0.000
A_06_P4485	YKL075C	44.000	0.000
A_06_P4486	YKL076C	19.000	0.000
A_06_P4487	YKL077W	8.000	0.000
A_06_P4488	YKL078W	51.000	0.000
A_06_P4489	YKL079W	163.000	0.000
A_06_P4490	YKL080W	4.000	0.000
A_06_P4491	YKL081W	15.000	0.000
A_06_P4492	YKL082C	16.000	0.000
A_06_P4493	YKL083W	45.000	0.000
A_06_P4494	YKL084W	71.000	0.000
A_06_P4495	YKL085W	279.000	0.000
A_06_P4496	YKL086W	27.000	0.000
A_06_P4497	YKL087C	202.000	0.000

A_06_P4498	YKL088W	5.000	0.000
A_06_P4499	YKL089W	567.000	0.000
A_06_P4500	YKL090W	15.000	0.000
A_06_P4501	YKL091C	289.000	0.000
A_06_P4502	YKL092C	32.000	0.000
A_06_P4503	YKL093W	43.000	0.000
A_06_P4504	YKL094W	65.000	0.000
A_06_P4505	YKL095W	189.000	0.000
A_06_P4506	YKL096W	82.000	0.000
A_06_P4507	YKL096W-A	29.000	0.000
A_06_P4508	YKL097C	41.000	0.000
A_06_P4509	YKL098W	25.000	0.000
A_06_P4510	YKL099C	80.000	0.000
A_06_P4511	YKL100C	61.000	0.000
A_06_P4512	YKL101W	8.000	0.000
A_06_P4513	YKL102C	241.000	0.000
A_06_P4514	YKL103C	20.000	0.000
A_06_P4515	YKL104C	16.000	0.000
A_06_P4516	YKL105C	25.000	0.000
A_06_P4517	YKL106C-A	24.000	0.000
A_06_P4518	YKL106W	21.000	0.000
A_06_P4519	YKL107W	32.000	0.000
A_06_P4520	YKL108W	4.000	0.000
A_06_P4521	YKL109W	68.000	0.000
A_06_P4522	YKL110C	11.000	0.000
A_06_P4523	YKL111C	11.000	0.000
A_06_P4524	YKL112W	226.000	0.000
A_06_P4525	YKL113C	23.000	0.000
A_06_P4526	YKL114C	78.000	0.000
A_06_P4527	YKL115C	289.000	0.000
A_06_P4528	YKL116C	15.000	0.000
A_06_P4529	YKL117W	86.000	0.000
A_06_P4530	YKL118W	36.000	0.000
A_06_P4531	YKL119C	10.000	0.000
A_06_P4532	YKL120W	26.000	0.000
A_06_P4533	YKL121W	172.000	0.000
A_06_P4534	YKL122C	175.000	0.000
A_06_P4535	YKL123W	254.000	0.000
A_06_P4535	YKL124W	5.400	2.881
A_06_P4536	YKL124W	70.600	2.881
A_06_P4537	YKL125W	20.000	0.000
A_06_P4538	YKL126W	21.000	0.000
A_06_P4539	YKL127W	254.000	0.000
A_06_P4540	YKL128C	66.000	0.000
A_06_P4541	YKL129C	8.000	0.000
A_06_P4542	YKL130C	230.000	0.000
A_06_P4543	YKL131W	6.000	0.000
A_06_P4544	YKL132C	60.000	0.000
A_06_P4545	YKL133C	25.000	0.000
A_06_P4546	YKL134C	122.000	0.000
A_06_P4547	YKL135C	87.000	0.000
A_06_P4548	YKL136W	69.000	0.000
A_06_P4549	YKL137W	75.000	0.000
A_06_P4550	YKL138C	25.000	0.000
A_06_P4551	YKL139W	16.000	0.000

A_06_P4552	YKL140W	49.000	0.000
A_06_P4553	YKL141W	114.000	0.000
A_06_P4554	YKL142W	62.000	0.000
A_06_P4555	YKL143W	10.000	0.000
A_06_P4556	YKL144C	300.000	0.000
A_06_P4557	YKL145W	123.000	0.000
A_06_P4558	YKL146W	14.000	0.000
A_06_P4559	YKL147C	54.000	0.000
A_06_P4560	YKL148C	24.000	0.000
A_06_P4561	YKL149C	401.000	0.000
A_06_P4562	YKL150W	16.000	0.000
A_06_P4563	YKL151C	59.000	0.000
A_06_P4564	YKL152C	24.000	0.000
A_06_P4565	YKL153W	142.000	0.000
A_06_P4566	YKL154W	93.000	0.000
A_06_P4567	YKL155C	23.000	0.000
A_06_P4568	YKL156W	147.000	0.000
A_06_P4569	YKL157W	1.000	0.000
A_06_P4570	YKL159C	20.000	0.000
A_06_P4571	YKL160W	39.000	0.000
A_06_P4572	YKL161C	112.000	0.000
A_06_P4573	YKL162C	43.000	0.000
A_06_P4574	YKL162C-A	308.000	0.000
A_06_P4575	YKL163W	16.000	0.000
A_06_P4576	YKL164C	21.000	0.000
A_06_P4577	YKL165C	55.000	0.000
A_06_P4578	YKL165C-A	216.000	0.000
A_06_P4579	YKL166C	194.000	0.000
A_06_P4580	YKL167C	82.000	0.000
A_06_P4581	YKL168C	20.000	0.000
A_06_P4582	YKL169C	88.000	0.000
A_06_P4583	YKL170W	31.000	0.000
A_06_P4584	YKL171W	301.000	0.000
A_06_P4585	YKL172W	23.000	0.000
A_06_P4586	YKL173W	82.000	0.000
A_06_P4587	YKL174C	178.000	0.000
A_06_P4588	YKL175W	20.000	0.000
A_06_P4589	YKL176C	159.000	0.000
A_06_P4590	YKL177W	119.000	0.000
A_06_P4591	YKL178C	43.000	0.000
A_06_P4592	YKL179C	72.000	0.000
A_06_P4593	YKL180W	305.000	0.000
A_06_P4594	YKL181W	452.000	0.000
A_06_P4595	YKL182W	294.000	0.000
A_06_P4596	YKL183W	239.000	0.000
A_06_P4597	YKL184W	110.000	0.000
A_06_P4598	YKL185W	54.000	0.000
A_06_P4599	YKL186C	20.000	0.000
A_06_P4600	YKL187C	87.000	0.000
A_06_P4601	YKL188C	2.000	0.000
A_06_P4602	YKL189W	3.000	0.000
A_06_P4603	YKL190W	149.000	0.000
A_06_P4604	YKL191W	25.000	0.000
A_06_P4605	YKL192C	7.000	0.000
A_06_P4606	YKL193C	123.000	0.000

A_06_P4607	YKL194C	14.000	0.000
A_06_P4608	YKL195W	42.000	0.000
A_06_P4609	YKL196C	15.000	0.000
A_06_P4610	YKL197C	17.000	0.000
A_06_P4611	YKL198C	23.800	0.447
A_06_P4612	YKL198C	0.200	0.447
A_06_P4613	YKL201C	20.000	0.000
A_06_P4614	YKL202W	112.000	0.000
A_06_P4615	YKL203C	295.000	0.000
A_06_P4616	YKL204W	8.000	0.000
A_06_P4617	YKL205W	72.000	0.000
A_06_P4618	YKL206C	131.000	0.000
A_06_P4619	YKL207W	430.000	0.000
A_06_P4620	YKL208W	9.000	0.000
A_06_P4621	YKL209C	73.000	0.000
A_06_P4622	YKL210W	10.000	0.000
A_06_P4623	YKL211C	35.000	0.000
A_06_P4624	YKL212W	106.000	0.000
A_06_P4625	YKL213C	39.000	0.000
A_06_P4626	YKL214C	88.000	0.000
A_06_P4627	YKL215C	32.000	0.000
A_06_P4628	YKL216W	23.000	0.000
A_06_P4629	YKL217W	151.000	0.000
A_06_P4630	YKL218C	582.000	0.000
A_06_P4631	YKL219W	27.000	0.000
A_06_P4632	YKL220C	48.000	0.000
A_06_P4633	YKL221W	9.000	0.000
A_06_P4634	YKL222C	92.000	0.000
A_06_P4635	YGL260W	8.600	3.782
A_06_P4635	YKL223W	30.000	0.000
A_06_P4636	YKL224C	66.800	0.447
A_06_P4637	YKL225W	36.000	0.000
A_06_P4638	YKR001C	462.000	0.000
A_06_P4639	YKR002W	219.000	0.000
A_06_P4640	YKR003W	64.000	0.000
A_06_P4641	YKR004C	61.000	0.000
A_06_P4642	YKR005C	109.000	0.000
A_06_P4643	YKR006C	11.000	0.000
A_06_P4644	YKR007W	65.000	0.000
A_06_P4645	YKR008W	16.000	0.000
A_06_P4646	YKR009C	157.000	0.000
A_06_P4647	YKR010C	297.000	0.000
A_06_P4648	YKR011C	29.000	0.000
A_06_P4649	YKR012C	238.000	0.000
A_06_P4650	YKR013W	15.000	0.000
A_06_P4651	YKR014C	29.000	0.000
A_06_P4652	YKR015C	21.000	0.000
A_06_P4653	YKR016W	14.000	0.000
A_06_P4654	YKR017C	45.000	0.000
A_06_P4655	YKR018C	117.000	0.000
A_06_P4656	YKR019C	96.000	0.000
A_06_P4657	YKR020W	68.000	0.000
A_06_P4658	YKR021W	56.000	0.000
A_06_P4659	YKR022C	15.000	0.000
A_06_P4660	YKR023W	20.000	0.000

A_06_P4661	YKR024C	76.000	0.000
A_06_P4662	YKR025W	5.000	0.000
A_06_P4663	YKR026C	47.000	0.000
A_06_P4664	YKR027W	157.000	0.000
A_06_P4665	YKR028W	38.000	0.000
A_06_P4666	YKR029C	11.000	0.000
A_06_P4667	YKR030W	23.000	0.000
A_06_P4668	YKR031C	119.000	0.000
A_06_P4669	YKR032W	33.000	0.000
A_06_P4670	YKR033C	17.000	0.000
A_06_P4671	YKR034W	71.000	0.000
A_06_P4672	YKR035C	26.000	0.000
A_06_P4673	YKR035W-A	111.000	0.000
A_06_P4674	YKR036C	10.000	0.000
A_06_P4675	YKR037C	16.000	0.000
A_06_P4676	YKR038C	27.000	0.000
A_06_P4677	YKR039W	29.000	0.000
A_06_P4678	YKR040C	10.000	0.000
A_06_P4679	YKR041W	21.000	0.000
A_06_P4680	YKR042W	4.000	0.000
A_06_P4681	YKR043C	112.000	0.000
A_06_P4682	YKR044W	53.000	0.000
A_06_P4683	YKR045C	16.000	0.000
A_06_P4684	YKR046C	23.000	0.000
A_06_P4685	YKR047W	10.000	0.000
A_06_P4686	YKR048C	25.000	0.000
A_06_P4687	YKR049C	150.000	0.000
A_06_P4688	YKR050W	15.000	0.000
A_06_P4689	YKR051W	40.000	0.000
A_06_P4690	YKR052C	118.000	0.000
A_06_P4691	YKR053C	150.000	0.000
A_06_P4692	YKR054C	33.000	0.000
A_06_P4693	YKR055W	49.000	0.000
A_06_P4694	YKR056W	93.000	0.000
A_06_P4695	YKR057W	32.000	0.000
A_06_P4696	YKR058W	7.000	0.000
A_06_P4697	YKR059W	50.000	0.000
A_06_P4698	YKR060W	73.000	0.000
A_06_P4699	YKR061W	74.000	0.000
A_06_P4700	YKR062W	61.000	0.000
A_06_P4701	YKR063C	142.000	0.000
A_06_P4702	YKR064W	55.000	0.000
A_06_P4703	YKR065C	35.000	0.000
A_06_P4704	YKR066C	89.000	0.000
A_06_P4705	YKR067W	128.000	0.000
A_06_P4706	YKR068C	42.000	0.000
A_06_P4707	YKR069W	66.000	0.000
A_06_P4708	YKR070W	443.000	0.000
A_06_P4709	YKR071C	25.000	0.000
A_06_P4710	YKR072C	74.000	0.000
A_06_P4711	YKR073C	24.000	0.000
A_06_P4712	YKR074W	47.000	0.000
A_06_P4713	YKR075C	124.000	0.000
A_06_P4714	YKR076W	164.000	0.000
A_06_P4715	YKR077W	42.000	0.000

A_06_P4716	YKR078W	305.000	0.000
A_06_P4717	YKR079C	12.000	0.000
A_06_P4718	YKR080W	72.000	0.000
A_06_P4719	YKR081C	262.000	0.000
A_06_P4720	YKR082W	30.000	0.000
A_06_P4721	YKR083C	33.000	0.000
A_06_P4722	YKR084C	14.000	0.000
A_06_P4723	YKR085C	3.000	0.000
A_06_P4724	YKR086W	38.000	0.000
A_06_P4725	YKR087C	335.000	0.000
A_06_P4726	YKR088C	99.000	0.000
A_06_P4727	YKR089C	12.000	0.000
A_06_P4728	YKR090W	78.000	0.000
A_06_P4729	YKR091W	60.000	0.000
A_06_P4730	YKR092C	35.000	0.000
A_06_P4731	YKR093W	42.000	0.000
A_06_P4732	YKR094C	58.000	0.000
A_06_P4733	YKR095W	78.000	0.000
A_06_P4734	YKR096W	62.000	0.000
A_06_P4735	YKR097W	46.000	0.000
A_06_P4736	YKR098C	14.000	0.000
A_06_P4737	YKR099W	71.000	0.000
A_06_P4738	YKR100C	22.000	0.000
A_06_P4739	YKR101W	76.000	0.000
A_06_P4740	YKR102W	129.000	0.000
A_06_P4741	YKR103W	19.000	0.000
A_06_P4742	YKR104W	6.000	0.000
A_06_P4743	YKR105C	30.600	0.548
A_06_P4744	YCL073C	0.200	0.447
A_06_P4744	YKR106W	34.000	0.000
A_06_P4745	YLL001W	227.000	0.000
A_06_P4746	YLL002W	21.000	0.000
A_06_P4747	YLL003W	55.000	0.000
A_06_P4748	YLL004W	69.000	0.000
A_06_P4749	YLL005C	92.000	0.000
A_06_P4750	YLL006W	481.000	0.000
A_06_P4751	YLL007C	28.000	0.000
A_06_P4752	YLL008W	6.000	0.000
A_06_P4753	YLL009C	5.000	0.000
A_06_P4754	YLL010C	39.000	0.000
A_06_P4755	YLL011W	32.000	0.000
A_06_P4756	YLL012W	11.000	0.000
A_06_P4757	YLL013C	29.000	0.000
A_06_P4758	YLL014W	282.000	0.000
A_06_P4759	YLL015W	15.000	0.000
A_06_P4760	YLL016W	92.000	0.000
A_06_P4761	YLL017W	27.000	0.000
A_06_P4762	YLL018C	229.000	0.000
A_06_P4763	YLL018C-A	64.000	0.000
A_06_P4764	YLL019C	17.000	0.000
A_06_P4765	YLL020C	58.000	0.000
A_06_P4766	YLL021W	63.000	0.000
A_06_P4767	YLL022C	13.000	0.000
A_06_P4768	YLL023C	10.000	0.000
A_06_P4769	YLL024C	29.000	0.000

A_06_P4770	YLL025W	44.000	0.000
A_06_P4771	YLL026W	53.000	0.000
A_06_P4772	YLL027W	14.000	0.000
A_06_P4773	YLL028W	7.000	0.000
A_06_P4774	YLL029W	13.000	0.000
A_06_P4775	YLL030C	5.000	0.000
A_06_P4776	YLL031C	23.000	0.000
A_06_P4777	YLL032C	73.000	0.000
A_06_P4778	YLL033W	72.000	0.000
A_06_P4779	YLL034C	21.000	0.000
A_06_P4780	YLL035W	7.000	0.000
A_06_P4781	YLL036C	17.000	0.000
A_06_P4782	YLL037W	52.000	0.000
A_06_P4783	YLL038C	10.000	0.000
A_06_P4784	YLL039C	21.000	0.000
A_06_P4785	YLL040C	19.000	0.000
A_06_P4786	YLL041C	193.000	0.000
A_06_P4787	YLL042C	42.000	0.000
A_06_P4788	YLL043W	2.000	0.000
A_06_P4789	YLL044W	3.000	0.000
A_06_P4790	YLL045C	10.000	0.000
A_06_P4791	YLL046C	23.000	0.000
A_06_P4792	YLL047W	9.000	0.000
A_06_P4793	YLL048C	46.000	0.000
A_06_P4794	YLL049W	21.000	0.000
A_06_P4795	YLL050C	151.000	0.000
A_06_P4796	YLL051C	19.000	0.000
A_06_P4797	YLL052C	106.000	0.000
A_06_P4798	YLL053C	29.000	0.000
A_06_P4799	YLL054C	145.000	0.000
A_06_P4800	YLL055W	16.000	0.000
A_06_P4801	YLL056C	33.000	0.000
A_06_P4802	YLL057C	12.000	0.000
A_06_P4803	YLL058W	568.000	0.000
A_06_P4804	YLL059C	16.000	0.000
A_06_P4805	YLL060C	61.000	0.000
A_06_P4806	YLL061W	29.000	0.000
A_06_P4807	YLL062C	32.000	0.000
A_06_P4808	YLL063C	474.000	0.000
A_06_P4809	YLL064C	233.000	8.216
A_06_P4809	YNR076W	46.200	2.490
A_06_P4810	YAL068W-A	0.200	0.447
A_06_P4810	YER188C-A	1.400	1.140
A_06_P4810	YLL065W	59.000	0.000
A_06_P4810	YOL166W-A	0.200	0.447
A_06_P4812	YBL113C	5.400	1.817
A_06_P4812	YDR545W	1.200	0.447
A_06_P4812	YER190W	1.600	1.517
A_06_P4812	YGR296W	0.600	0.894
A_06_P4812	YHL050C	4.000	0.707
A_06_P4812	YHR219W	1.000	0.000
A_06_P4812	YLL066C	40.000	0.000
A_06_P4812	YLL067C	88.400	0.548
A_06_P4812	YLR467W	0.600	0.548
A_06_P4812	YML133C	17.800	0.447

A_06_P4812	YNL339C	1.400	0.548
A_06_P4812	YOR396W	12.000	2.236
A_06_P4812	YPL283C	0.200	0.447
A_06_P4812	YPR204W	23.000	3.742
A_06_P4813	YLR001C	52.000	0.000
A_06_P4814	YLR002C	266.000	0.000
A_06_P4815	YLR003C	50.000	0.000
A_06_P4816	YLR004C	36.000	0.000
A_06_P4817	YLR005W	69.000	0.000
A_06_P4818	YLR006C	90.000	0.000
A_06_P4819	YLR007W	22.000	0.000
A_06_P4820	YLR008C	131.000	0.000
A_06_P4821	YLR009W	62.000	0.000
A_06_P4822	YLR010C	49.000	0.000
A_06_P4823	YLR011W	54.000	0.000
A_06_P4824	YLR012C	26.000	0.000
A_06_P4825	YLR013W	85.000	0.000
A_06_P4826	YLR014C	15.000	0.000
A_06_P4827	YLR015W	59.000	0.000
A_06_P4828	YLR016C	93.000	0.000
A_06_P4829	YLR017W	124.000	0.000
A_06_P4830	YLR018C	63.000	0.000
A_06_P4831	YLR019W	76.000	0.000
A_06_P4832	YLR020C	71.000	0.000
A_06_P4833	YLR021W	47.000	0.000
A_06_P4834	YLR022C	15.000	0.000
A_06_P4835	YLR023C	121.000	0.000
A_06_P4836	YLR024C	6.000	0.000
A_06_P4837	YLR025W	46.000	0.000
A_06_P4838	YLR026C	104.000	0.000
A_06_P4839	YLR027C	244.000	0.000
A_06_P4840	YLR028C	55.000	0.000
A_06_P4841	YLR029C	54.000	0.000
A_06_P4842	YLR030W	153.000	0.000
A_06_P4843	YLR031W	36.000	0.000
A_06_P4844	YLR032W	18.000	0.000
A_06_P4845	YLR033W	64.000	0.000
A_06_P4846	YLR034C	66.000	0.000
A_06_P4847	YLR035C	11.000	0.000
A_06_P4848	YLR036C	4.000	0.000
A_06_P4849	YLR037C	60.000	0.000
A_06_P4850	YLR038C	6.000	0.000
A_06_P4851	YLR039C	12.000	0.000
A_06_P4852	YLR040C	77.000	0.000
A_06_P4853	YLR041W	73.000	0.000
A_06_P4854	YLR042C	51.000	0.000
A_06_P4855	YLR043C	543.000	0.000
A_06_P4856	YLR044C	152.000	0.000
A_06_P4857	YLR045C	106.000	0.000
A_06_P4858	YLR046C	48.000	0.000
A_06_P4859	YLR047C	95.000	0.000
A_06_P4860	YLR048W	43.000	0.000
A_06_P4861	YLR049C	25.000	0.000
A_06_P4862	YLR050C	112.000	0.000
A_06_P4863	YLR051C	16.000	0.000

A_06_P4864	YLR052W	48.000	0.000
A_06_P4865	YLR053C	35.000	0.000
A_06_P4866	YLR054C	17.000	0.000
A_06_P4867	YLR055C	21.000	0.000
A_06_P4868	YLR056W	76.000	0.000
A_06_P4869	YLR057W	73.000	0.000
A_06_P4870	YLR058C	42.000	0.000
A_06_P4871	YLR059C	5.000	0.000
A_06_P4872	YLR060W	105.000	0.000
A_06_P4873	YLR061W	66.000	0.000
A_06_P4873	YLR362W	0.200	0.447
A_06_P4874	YLR062C	45.000	0.000
A_06_P4875	YLR063W	20.000	0.000
A_06_P4876	YLR064W	35.000	0.000
A_06_P4877	YLR065C	134.000	0.000
A_06_P4878	YLR066W	400.000	0.000
A_06_P4879	YLR067C	82.000	0.000
A_06_P4880	YLR068W	360.000	0.000
A_06_P4881	YLR069C	41.000	0.000
A_06_P4882	YLR070C	16.000	0.000
A_06_P4883	YLR071C	113.000	0.000
A_06_P4884	YLR072W	71.000	0.000
A_06_P4885	YLR073C	97.000	0.000
A_06_P4886	YLR074C	103.000	0.000
A_06_P4887	YLR075W	65.000	0.000
A_06_P4888	YLR076C	28.000	0.000
A_06_P4889	YLR077W	42.000	0.000
A_06_P4890	YLR078C	59.000	0.000
A_06_P4891	YLR079W	115.000	0.000
A_06_P4892	YLR080W	146.000	0.000
A_06_P4893	YLR081W	13.000	0.000
A_06_P4894	YLR082C	173.000	0.000
A_06_P4895	YLR083C	46.000	0.000
A_06_P4896	YLR084C	14.000	0.000
A_06_P4897	YLR085C	93.000	0.000
A_06_P4898	YLR086W	4.000	0.000
A_06_P4899	YLR087C	449.000	0.000
A_06_P4900	YLR088W	52.000	0.000
A_06_P4901	YLR089C	8.000	0.000
A_06_P4902	YLR090W	3.000	0.000
A_06_P4903	YLR091W	19.000	0.000
A_06_P4904	YLR092W	56.000	0.000
A_06_P4905	YLR093C	1000.000	0.000
A_06_P4906	YLR094C	55.000	0.000
A_06_P4907	YLR095C	13.000	0.000
A_06_P4908	YLR096W	27.000	0.000
A_06_P4909	YLR097C	309.000	0.000
A_06_P4910	YLR098C	11.000	0.000
A_06_P4911	YLR099C	4.000	0.000
A_06_P4912	YLR099W-A	31.000	0.000
A_06_P4913	YLR100W	52.000	0.000
A_06_P4914	YLR101C	238.000	0.000
A_06_P4915	YLR102C	23.000	0.000
A_06_P4916	YLR103C	78.000	0.000
A_06_P4917	YLR104W	149.000	0.000

A_06_P4918	YLR105C	183.000	0.000
A_06_P4919	YLR106C	163.000	0.000
A_06_P4920	YLR107W	59.000	0.000
A_06_P4921	YLR108C	79.000	0.000
A_06_P4922	YLR109W	11.000	0.000
A_06_P4923	YLR110C	2.000	0.000
A_06_P4924	YLR111W	90.000	0.000
A_06_P4925	YLR112W	198.000	0.000
A_06_P4926	YLR113W	24.000	0.000
A_06_P4927	YLR114C	62.000	0.000
A_06_P4928	YLR115W	62.000	0.000
A_06_P4929	YLR116W	88.000	0.000
A_06_P4930	YLR117C	10.000	0.000
A_06_P4931	YLR118C	146.000	0.000
A_06_P4932	YLR119W	15.000	0.000
A_06_P4933	YLR120C	19.000	0.000
A_06_P4934	YLR121C	152.000	0.000
A_06_P4935	YLR122C	277.000	0.000
A_06_P4936	YLR123C	348.000	0.000
A_06_P4937	YLR124W	6.000	0.000
A_06_P4938	YLR125W	131.000	0.000
A_06_P4939	YLR126C	68.000	0.000
A_06_P4940	YLR127C	72.000	0.000
A_06_P4941	YLR128W	59.000	0.000
A_06_P4942	YLR129W	206.000	0.000
A_06_P4943	YLR130C	209.000	0.000
A_06_P4944	YLR131C	34.000	0.000
A_06_P4945	YLR132C	58.000	0.000
A_06_P4946	YLR133W	20.000	0.000
A_06_P4947	YLR134W	87.000	0.000
A_06_P4948	YLR135W	80.000	0.000
A_06_P4949	YLR136C	52.000	0.000
A_06_P4950	YLR137W	56.000	0.000
A_06_P4951	YLR138W	19.000	0.000
A_06_P4952	YLR139C	437.000	0.000
A_06_P4953	YLR140W	21.000	0.000
A_06_P4953	YLR141W	53.600	9.529
A_06_P4954	YLR141W	70.400	9.529
A_06_P4955	YLR142W	19.000	0.000
A_06_P4956	YLR143W	28.000	0.000
A_06_P4957	YLR144C	12.000	0.000
A_06_P4958	YLR145W	112.000	0.000
A_06_P4959	YLR146C	153.000	0.000
A_06_P4960	YLR147C	17.000	0.000
A_06_P4961	YLR148W	101.000	0.000
A_06_P4962	YLR149C	30.000	0.000
A_06_P4963	YLR149C-A	21.000	0.000
A_06_P4964	YLR150W	465.000	0.000
A_06_P4965	YLR151C	107.000	0.000
A_06_P4966	YLR152C	28.000	0.000
A_06_P4967	YLR153C	6.000	0.000
A_06_P4968	YLR154C	33.000	0.000
A_06_P4969	YLR155C	16.000	4.743
A_06_P4969	YLR157C	11.200	1.924
A_06_P4969	YLR158C	1.800	0.837

A_06_P4969	YLR160C	1.200	0.447
A_06_P4970	YLR156W	92.600	2.408
A_06_P4970	YLR157W-E	1.200	0.447
A_06_P4970	YLR159W	4.400	2.702
A_06_P4970	YLR161W	10.000	1.581
A_06_P4971	YLR155C	16.000	0.707
A_06_P4971	YLR157C	12.600	3.507
A_06_P4971	YLR158C	2.400	0.548
A_06_P4971	YLR160C	0.800	0.837
A_06_P4972	YLR155C	16.400	5.177
A_06_P4972	YLR157C	14.800	1.483
A_06_P4972	YLR158C	2.200	1.304
A_06_P4972	YLR160C	0.600	0.894
A_06_P4973	YLR156W	89.200	6.099
A_06_P4973	YLR157W-E	1.200	0.447
A_06_P4973	YLR159W	6.600	2.074
A_06_P4973	YLR161W	10.600	1.517
A_06_P4974	YLR155C	16.600	2.702
A_06_P4974	YLR157C	10.400	2.702
A_06_P4974	YLR158C	2.600	1.517
A_06_P4974	YLR160C	1.400	0.894
A_06_P4975	YLR156W	98.200	5.404
A_06_P4975	YLR157W-E	0.600	0.548
A_06_P4975	YLR159W	4.000	1.414
A_06_P4975	YLR161W	8.400	1.517
A_06_P4976	YLR162W	66.000	0.000
A_06_P4977	YLR163C	26.000	0.000
A_06_P4978	YLR164W	39.000	0.000
A_06_P4979	YLR165C	46.000	0.000
A_06_P4980	YLR166C	38.000	0.000
A_06_P4981	YLR167W	29.000	0.000
A_06_P4982	YLR168C	185.000	0.000
A_06_P4983	YLR169W	137.000	0.000
A_06_P4984	YLR170C	93.000	0.000
A_06_P4985	YLR171W	155.000	0.000
A_06_P4986	YLR172C	163.000	0.000
A_06_P4987	YLR173W	7.000	0.000
A_06_P4988	YLR174W	9.000	0.000
A_06_P4989	YLR175W	834.000	0.000
A_06_P4990	YLR176C	388.000	0.000
A_06_P4991	YLR177W	98.000	0.000
A_06_P4992	YLR178C	167.000	0.000
A_06_P4993	YLR179C	55.000	0.000
A_06_P4994	YLR180W	32.000	0.000
A_06_P4995	YLR181C	25.000	0.000
A_06_P4996	YLR182W	8.000	0.000
A_06_P4997	YLR183C	29.000	0.000
A_06_P4998	YLR184W	65.000	0.000
A_06_P4999	YLR185W	264.000	0.000
A_06_P5000	YLR186W	6.000	0.000
A_06_P5001	YLR187W	241.000	0.000
A_06_P5002	YLR188W	9.000	0.000
A_06_P5003	YLR189C	162.000	0.000
A_06_P5004	YLR190W	22.000	0.000
A_06_P5005	YLR191W	13.000	0.000

A_06_P5006	YLR192C	717.000	0.000
A_06_P5007	YLR193C	161.000	0.000
A_06_P5008	YLR194C	34.000	0.000
A_06_P5009	YLR195C	193.000	0.000
A_06_P5010	YLR196W	36.000	0.000
A_06_P5011	YLR197W	106.000	0.000
A_06_P5012	YLR198C	43.000	0.000
A_06_P5013	YLR199C	152.000	0.000
A_06_P5014	YLR200W	96.000	0.000
A_06_P5015	YLR201C	146.000	0.000
A_06_P5016	YLR202C	40.000	0.000
A_06_P5017	YLR203C	750.000	0.000
A_06_P5018	YLR204W	38.000	0.000
A_06_P5019	YLR205C	46.000	0.000
A_06_P5020	YLR206W	71.000	0.000
A_06_P5021	YLR207W	17.000	0.000
A_06_P5022	YLR208W	33.000	0.000
A_06_P5023	YLR209C	48.000	0.000
A_06_P5024	YLR210W	77.000	0.000
A_06_P5025	YLR211C	111.000	0.000
A_06_P5026	YLR212C	19.000	0.000
A_06_P5027	YLR213C	59.000	0.000
A_06_P5028	YLR214W	18.000	0.000
A_06_P5029	YLR215C	14.000	0.000
A_06_P5030	YLR216C	158.000	0.000
A_06_P5031	YLR217W	81.000	0.000
A_06_P5032	YLR218C	133.000	0.000
A_06_P5033	YLR219W	4.000	0.000
A_06_P5034	YLR220W	5.000	0.000
A_06_P5035	YLR221C	31.000	0.000
A_06_P5036	YLR222C	41.000	0.000
A_06_P5037	YLR223C	11.000	0.000
A_06_P5038	YLR224W	244.000	0.000
A_06_P5039	YLR225C	37.000	0.000
A_06_P5040	YLR226W	44.000	0.000
A_06_P5041	YLR227C	4.000	0.000
A_06_P5042	YLR228C	63.000	0.000
A_06_P5043	YLR229C	37.000	0.000
A_06_P5044	YLR230W	78.000	0.000
A_06_P5045	YLR231C	70.000	0.000
A_06_P5046	YLR232W	104.000	0.000
A_06_P5047	YLR233C	89.000	0.000
A_06_P5048	YLR234W	39.000	0.000
A_06_P5049	YLR235C	36.000	0.000
A_06_P5050	YLR236C	16.000	0.000
A_06_P5051	YLR237W	83.000	0.000
A_06_P5052	YLR238W	50.000	0.000
A_06_P5053	YLR239C	111.000	0.000
A_06_P5054	YLR240W	55.000	0.000
A_06_P5055	YLR241W	30.000	0.000
A_06_P5056	YLR242C	203.000	0.000
A_06_P5057	YLR243W	79.000	0.000
A_06_P5058	YLR244C	52.000	0.000
A_06_P5059	YLR245C	213.000	0.000
A_06_P5060	YLR246W	140.000	0.000

A_06_P5061	YLR247C	126.000	0.000
A_06_P5062	YLR248W	165.000	0.000
A_06_P5063	YLR249W	42.000	0.000
A_06_P5064	YLR250W	8.000	0.000
A_06_P5065	YLR251W	12.000	0.000
A_06_P5066	YLR252W	30.000	0.000
A_06_P5067	YLR253W	1.000	0.000
A_06_P5068	YLR254C	12.000	0.000
A_06_P5069	YLR255C	64.000	0.000
A_06_P5070	YLR256W	156.000	0.000
A_06_P5071	YLR257W	19.000	0.000
A_06_P5072	YLR258W	33.000	0.000
A_06_P5073	YLR259C	50.000	0.000
A_06_P5074	YLR260W	113.000	0.000
A_06_P5075	YLR261C	13.000	0.000
A_06_P5075	YLR262C	16.400	1.342
A_06_P5076	YLR262C	3.600	1.342
A_06_P5077	YLR262C-A	180.000	0.000
A_06_P5078	YLR263W	41.000	0.000
A_06_P5079	YLR264W	211.000	0.000
A_06_P5080	YLR265C	125.000	0.000
A_06_P5081	YLR266C	113.000	0.000
A_06_P5082	YLR267W	10.000	0.000
A_06_P5083	YLR268W	58.000	0.000
A_06_P5084	YLR269C	17.000	0.000
A_06_P5085	YLR270W	143.000	0.000
A_06_P5086	YLR271W	26.000	0.000
A_06_P5087	YLR272C	447.000	0.000
A_06_P5088	YLR273C	38.000	0.000
A_06_P5089	YLR274W	16.000	0.000
A_06_P5090	YLR275W	22.000	0.000
A_06_P5091	YLR276C	18.000	0.000
A_06_P5092	YLR277C	10.000	0.000
A_06_P5093	YLR278C	39.000	0.000
A_06_P5094	YLR279W	101.000	0.000
A_06_P5095	YLR280C	161.000	0.000
A_06_P5096	YLR281C	4.000	0.000
A_06_P5097	YLR282C	26.000	0.000
A_06_P5098	YLR283W	55.000	0.000
A_06_P5099	YLR284C	94.000	0.000
A_06_P5100	YLR285W	7.000	0.000
A_06_P5101	YLR286C	27.000	0.000
A_06_P5102	YLR287C	27.000	0.000
A_06_P5103	YLR287C-A	65.000	0.000
A_06_P5104	YLR288C	51.000	0.000
A_06_P5105	YLR289W	47.000	0.000
A_06_P5106	YLR290C	43.000	0.000
A_06_P5107	YLR291C	22.000	0.000
A_06_P5108	YLR292C	53.000	0.000
A_06_P5109	YLR293C	80.000	0.000
A_06_P5110	YLR294C	21.000	0.000
A_06_P5111	YLR295C	37.000	0.000
A_06_P5112	YLR296W	21.000	0.000
A_06_P5113	YLR297W	298.000	0.000
A_06_P5114	YLR298C	20.000	0.000

A_06_P5115	YLR299W	57.000	0.000
A_06_P5116	YLR300W	79.000	0.000
A_06_P5117	YLR301W	132.000	0.000
A_06_P5118	YLR302C	35.000	0.000
A_06_P5119	YLR303W	18.000	0.000
A_06_P5120	YLR304C	6.000	0.000
A_06_P5121	YLR305C	66.000	0.000
A_06_P5122	YLR306W	43.000	0.000
A_06_P5123	YLR307W	8.000	0.000
A_06_P5124	YLR308W	37.000	0.000
A_06_P5125	YLR309C	191.000	0.000
A_06_P5126	YLR310C	19.000	0.000
A_06_P5127	YLR311C	82.000	0.000
A_06_P5128	YLR312C	40.000	0.000
A_06_P5129	YLR312W-A	13.000	0.000
A_06_P5130	YLR313C	1.000	0.000
A_06_P5131	YLR314C	9.000	0.000
A_06_P5132	YLR315W	22.000	0.000
A_06_P5133	YLR316C	16.000	0.000
A_06_P5134	YLR317W	20.000	0.000
A_06_P5135	YLR318W	52.000	0.000
A_06_P5136	YLR319C	77.000	0.000
A_06_P5137	YLR320W	17.000	0.000
A_06_P5138	YLR321C	54.000	0.000
A_06_P5139	YLR322W	157.000	0.000
A_06_P5140	YLR323C	65.000	0.000
A_06_P5141	YLR324W	44.000	0.000
A_06_P5142	YLR325C	101.000	0.000
A_06_P5143	YLR326W	23.000	0.000
A_06_P5144	YLR327C	20.000	0.000
A_06_P5145	YLR328W	34.000	0.000
A_06_P5146	YLR329W	89.000	0.000
A_06_P5147	YLR330W	51.000	0.000
A_06_P5148	YLR331C	62.000	0.000
A_06_P5149	YLR332W	81.000	0.000
A_06_P5150	YLR333C	112.000	0.000
A_06_P5151	YLR334C	134.000	0.000
A_06_P5152	YLR335W	5.000	0.000
A_06_P5153	YLR336C	47.000	0.000
A_06_P5154	YLR337C	28.000	0.000
A_06_P5155	YLR338W	16.000	0.000
A_06_P5156	YLR339C	256.000	0.000
A_06_P5157	YLR340W	200.000	0.000
A_06_P5158	YLR341W	158.000	0.000
A_06_P5159	YLR342W	216.000	0.000
A_06_P5160	YLR343W	34.000	0.000
A_06_P5161	YLR344W	116.000	0.000
A_06_P5162	YLR345W	13.000	0.000
A_06_P5163	YLR346C	15.000	0.000
A_06_P5164	YLR347C	102.000	0.000
A_06_P5165	YLR348C	59.000	0.000
A_06_P5166	YLR349W	3.000	0.000
A_06_P5167	YLR350W	43.000	0.000
A_06_P5168	YLR351C	39.000	0.000
A_06_P5169	YLR352W	21.000	0.000

A_06_P5170	YLR353W	30.000	0.000
A_06_P5171	YLR354C	15.000	0.000
A_06_P5172	YLR355C	25.000	0.000
A_06_P5173	YLR356W	42.000	0.000
A_06_P5174	YLR357W	16.000	0.000
A_06_P5175	YLR358C	3.000	0.000
A_06_P5176	YLR359W	120.000	0.000
A_06_P5177	YLR360W	57.000	0.000
A_06_P5178	YLR361C	25.000	0.000
A_06_P5179	YLR362W	1000.000	0.000
A_06_P5180	YLR363C	31.000	0.000
A_06_P5181	YLR363W-A	14.000	0.000
A_06_P5182	YLR364W	9.000	0.000
A_06_P5183	YLR365W	15.000	0.000
A_06_P5184	YLR366W	68.000	0.000
A_06_P5185	YLR367W	11.000	0.000
A_06_P5186	YLR368W	203.000	0.000
A_06_P5187	YLR369W	415.000	0.000
A_06_P5188	YLR370C	103.000	0.000
A_06_P5189	YLR371W	70.000	0.000
A_06_P5190	YLR372W	29.000	0.000
A_06_P5191	YLR373C	104.000	0.000
A_06_P5192	YLR374C	110.000	0.000
A_06_P5193	YLR375W	6.000	0.000
A_06_P5194	YLR376C	12.000	0.000
A_06_P5195	YLR377C	102.000	0.000
A_06_P5196	YLR378C	6.000	0.000
A_06_P5197	YLR379W	43.000	0.000
A_06_P5198	YLR380W	28.000	0.000
A_06_P5199	YLR381W	167.000	0.000
A_06_P5200	YLR382C	57.000	0.000
A_06_P5201	YLR383W	62.000	0.000
A_06_P5202	YLR384C	22.000	0.000
A_06_P5203	YLR385C	106.000	0.000
A_06_P5204	YLR386W	98.000	0.000
A_06_P5205	YLR387C	149.000	0.000
A_06_P5206	YLR388W	185.000	0.000
A_06_P5207	YLR389C	77.000	0.000
A_06_P5208	YLR390W	93.000	0.000
A_06_P5209	YLR390W-A	19.000	0.000
A_06_P5210	YLR392C	29.000	0.000
A_06_P5211	YLR393W	120.000	0.000
A_06_P5212	YLR394W	230.000	0.000
A_06_P5213	YLR395C	18.000	0.000
A_06_P5214	YLR396C	43.000	0.000
A_06_P5215	YLR397C	17.000	0.000
A_06_P5216	YLR398C	17.000	0.000
A_06_P5217	YLR399C	195.000	0.000
A_06_P5218	YLR400W	56.000	0.000
A_06_P5219	YLR401C	67.000	0.000
A_06_P5220	YLR402W	21.000	0.000
A_06_P5221	YLR403W	254.000	0.000
A_06_P5222	YLR404W	3.000	0.000
A_06_P5223	YLR405W	9.000	0.000
A_06_P5224	YLR406C	177.000	0.000

A_06_P5225	YLR407W	35.000	0.000
A_06_P5226	YLR408C	102.000	0.000
A_06_P5227	YLR409C	115.000	0.000
A_06_P5228	YLR410W	403.000	0.000
A_06_P5229	YLR411W	45.000	0.000
A_06_P5230	YLR412W	117.000	0.000
A_06_P5231	YLR413W	7.000	0.000
A_06_P5232	YLR414C	341.000	0.000
A_06_P5233	YLR415C	12.000	0.000
A_06_P5234	YLR416C	20.000	0.000
A_06_P5235	YLR417W	173.000	0.000
A_06_P5236	YLR418C	75.000	0.000
A_06_P5237	YLR419W	28.000	0.000
A_06_P5238	YLR420W	23.000	0.000
A_06_P5239	YLR421C	28.000	0.000
A_06_P5240	YLR422W	31.000	0.000
A_06_P5241	YLR423C	45.000	0.000
A_06_P5242	YLR424W	50.000	0.000
A_06_P5243	YLR425W	25.000	0.000
A_06_P5244	YLR426W	199.000	0.000
A_06_P5245	YLR427W	95.000	0.000
A_06_P5246	YLR428C	13.000	0.000
A_06_P5247	YLR429W	7.000	0.000
A_06_P5248	YLR430W	11.000	0.000
A_06_P5249	YLR431C	16.000	0.000
A_06_P5250	YLR432W	615.000	0.000
A_06_P5251	YLR433C	40.000	0.000
A_06_P5252	YLR434C	8.000	0.000
A_06_P5253	YLR435W	27.000	0.000
A_06_P5254	YLR436C	50.000	0.000
A_06_P5255	YLR437C	80.000	0.000
A_06_P5256	YLR438C-A	9.000	0.000
A_06_P5257	YLR438W	88.000	0.000
A_06_P5258	YLR439W	53.000	0.000
A_06_P5259	YLR440C	27.000	0.000
A_06_P5260	YLR441C	122.000	0.000
A_06_P5261	YLR442C	22.000	0.000
A_06_P5262	YLR443W	25.000	0.000
A_06_P5263	YLR444C	119.000	0.000
A_06_P5264	YLR445W	6.000	0.000
A_06_P5265	YLR446W	63.000	0.000
A_06_P5266	YLR447C	214.000	0.000
A_06_P5267	YLR448W	210.000	0.000
A_06_P5268	YLR449W	33.000	0.000
A_06_P5269	YLR450W	51.000	0.000
A_06_P5270	YLR451W	41.000	0.000
A_06_P5271	YLR452C	84.000	0.000
A_06_P5272	YLR453C	97.000	0.000
A_06_P5273	YLR454W	8.000	0.000
A_06_P5274	YLR455W	53.000	0.000
A_06_P5275	YLR456W	66.000	0.000
A_06_P5276	YLR457C	227.000	0.000
A_06_P5277	YLR458W	29.000	0.000
A_06_P5278	YLR459W	20.000	0.000
A_06_P5279	YLR460C	138.000	0.000

A_06_P5280	YLR461W	372.000	5.701
A_06_P5281	YIL177C	0.200	0.447
A_06_P5281	YLR462W	58.000	0.000
A_06_P5282	YLR463C	104.000	0.000
A_06_P5283	YDR545W	63.800	8.526
A_06_P5283	YEL076C-A	11.200	3.114
A_06_P5283	YER190W	76.200	3.564
A_06_P5283	YGR296W	18.400	3.050
A_06_P5283	YIL177C	2.600	1.817
A_06_P5283	YJL225C	36.200	5.020
A_06_P5283	YLR464W	16.800	2.280
A_06_P5283	YLR466W	2.000	1.000
A_06_P5283	YLR467W	32.200	5.933
A_06_P5283	YNL339C	64.400	7.537
A_06_P5283	YPL283C	4.800	1.095
A_06_P5284	YLR465C	28.000	0.000
A_06_P5287	YML001W	163.000	0.000
A_06_P5288	YML002W	447.000	0.000
A_06_P5289	YML003W	15.000	0.000
A_06_P5290	YML004C	14.000	0.000
A_06_P5291	YML005W	24.000	0.000
A_06_P5292	YML006C	37.000	0.000
A_06_P5293	YML007C-A	15.000	0.000
A_06_P5294	YML007W	57.000	0.000
A_06_P5295	YML008C	18.000	0.000
A_06_P5296	YML009C	53.000	0.000
A_06_P5297	YML009C-A	57.000	0.000
A_06_P5298	YML010W	135.000	0.000
A_06_P5299	YML009W-B	12.000	0.000
A_06_P5300	YML011C	15.000	0.000
A_06_P5301	YML012W	14.000	0.000
A_06_P5302	YML012C-A	5.000	0.000
A_06_P5303	YML013W	172.000	0.000
A_06_P5304	YML014W	20.000	0.000
A_06_P5305	YML015C	294.000	0.000
A_06_P5306	YML016C	19.000	0.000
A_06_P5307	YML017W	54.000	0.000
A_06_P5308	YML018C	72.000	0.000
A_06_P5309	YML019W	70.000	0.000
A_06_P5310	YML020W	16.000	0.000
A_06_P5311	YML021C	443.000	0.000
A_06_P5312	YML022W	44.000	0.000
A_06_P5313	YML023C	23.000	0.000
A_06_P5314	YML024W	49.000	0.000
A_06_P5315	YML025C	52.000	0.000
A_06_P5316	YML026C	46.000	0.000
A_06_P5317	YML027W	72.000	0.000
A_06_P5318	YML028W	19.000	0.000
A_06_P5319	YML029W	59.000	0.000
A_06_P5320	YML030W	55.000	0.000
A_06_P5321	YML031W	86.000	0.000
A_06_P5322	YML032C	64.000	0.000
A_06_P5323	YML034W	600.000	0.000
A_06_P5324	YML035C	99.000	0.000
A_06_P5325	YML034C-A	28.000	0.000

A_06_P5326	YML036W	43.000	0.000
A_06_P5327	YML037C	46.000	0.000
A_06_P5328	YML038C	139.000	0.000
A_06_P5329	YML041C	70.000	0.000
A_06_P5330	YML042W	93.000	0.000
A_06_P5331	YML043C	270.000	0.000
A_06_P5332	YML046W	17.000	0.000
A_06_P5333	YML047C	19.000	0.000
A_06_P5334	YML048W	8.000	0.000
A_06_P5335	YML047W-A	16.000	0.000
A_06_P5336	YML049C	71.000	0.000
A_06_P5337	YML050W	136.000	0.000
A_06_P5338	YML051W	413.000	0.000
A_06_P5339	YML052W	17.000	0.000
A_06_P5340	YML053C	54.000	0.000
A_06_P5341	YML054C	29.000	0.000
A_06_P5342	YML055W	9.000	0.000
A_06_P5343	YML056C	32.000	0.000
A_06_P5344	YML057W	57.000	0.000
A_06_P5345	YML057C-A	11.000	0.000
A_06_P5346	YML058W	91.000	0.000
A_06_P5347	YML058W-A	514.000	0.000
A_06_P5348	YML059C	57.000	0.000
A_06_P5349	YML060W	39.000	0.000
A_06_P5350	YML061C	442.000	0.000
A_06_P5351	YML062C	252.000	0.000
A_06_P5352	YML063W	47.000	0.000
A_06_P5353	YML064C	28.000	0.000
A_06_P5354	YML065W	21.000	0.000
A_06_P5355	YML066C	16.000	0.000
A_06_P5356	YML067C	21.000	0.000
A_06_P5357	YML068W	14.000	0.000
A_06_P5358	YML069W	75.000	0.000
A_06_P5359	YML070W	21.000	0.000
A_06_P5360	YML071C	74.000	0.000
A_06_P5361	YML072C	73.000	0.000
A_06_P5362	YML073C	26.000	0.000
A_06_P5363	YML074C	12.000	0.000
A_06_P5364	YML075C	64.000	0.000
A_06_P5365	YML076C	8.000	0.000
A_06_P5366	YML077W	120.000	0.000
A_06_P5367	YML078W	112.000	0.000
A_06_P5368	YML079W	31.000	0.000
A_06_P5369	YML080W	16.000	0.000
A_06_P5370	YML081C-A	71.000	0.000
A_06_P5371	YML081W	74.000	0.000
A_06_P5372	YML082W	28.000	0.000
A_06_P5373	YML083C	141.000	0.000
A_06_P5374	YML084W	102.000	0.000
A_06_P5375	YML085C	22.000	0.000
A_06_P5376	YML086C	1.000	0.000
A_06_P5377	YML087C	28.000	0.000
A_06_P5378	YML088W	8.000	0.000
A_06_P5379	YML089C	5.000	0.000
A_06_P5380	YML090W	123.000	0.000

A_06_P5381	YML091C	23.000	0.000
A_06_P5382	YML092C	221.000	0.000
A_06_P5383	YML093W	41.000	0.000
A_06_P5384	YML094W	154.000	0.000
A_06_P5385	YML095C	493.000	0.000
A_06_P5386	YML094C-A	6.000	0.000
A_06_P5387	YML096W	59.000	0.000
A_06_P5388	YML097C	10.000	0.000
A_06_P5389	YML098W	38.000	0.000
A_06_P5390	YML099C	143.000	0.000
A_06_P5391	YML100W	361.000	0.000
A_06_P5392	YML099W-A	37.000	0.000
A_06_P5393	YML101C	36.000	0.000
A_06_P5394	YML101C-A	1000.000	0.000
A_06_P5395	YML102W	52.000	0.000
A_06_P5396	YML103C	104.000	0.000
A_06_P5397	YML104C	26.000	0.000
A_06_P5398	YML105C	179.000	0.000
A_06_P5399	YML106W	89.000	0.000
A_06_P5400	YML107C	286.000	0.000
A_06_P5401	YML108W	27.000	0.000
A_06_P5402	YML109W	10.000	0.000
A_06_P5403	YML110C	6.000	0.000
A_06_P5404	YML111W	59.000	0.000
A_06_P5405	YML112W	1.000	0.000
A_06_P5406	YML113W	10.000	0.000
A_06_P5407	YML114C	53.000	0.000
A_06_P5408	YML115C	6.000	0.000
A_06_P5409	YML116W	259.000	0.000
A_06_P5410	YML117W	13.000	0.000
A_06_P5411	YML116W-A	32.000	0.000
A_06_P5412	YML118W	54.000	0.000
A_06_P5413	YML119W	18.000	0.000
A_06_P5414	YML120C	19.000	0.000
A_06_P5415	YML121W	143.000	0.000
A_06_P5416	YML122C	9.000	0.000
A_06_P5417	YML123C	30.000	0.000
A_06_P5418	YML124C	95.000	0.000
A_06_P5419	YML125C	7.000	0.000
A_06_P5420	YML126C	295.000	0.000
A_06_P5421	YML127W	225.000	0.000
A_06_P5422	YML128C	127.000	0.000
A_06_P5423	YML129C	38.000	0.000
A_06_P5424	YML130C	83.000	0.000
A_06_P5425	YML131W	18.000	0.000
A_06_P5426	YBR302C	7.000	1.581
A_06_P5426	YML132W	29.200	2.049
A_06_P5427	YEL077C	0.200	0.447
A_06_P5428	YMR001C	49.000	0.000
A_06_P5429	YMR002W	398.000	0.000
A_06_P5430	YMR003W	61.000	0.000
A_06_P5431	YMR004W	33.000	0.000
A_06_P5432	YMR005W	88.000	0.000
A_06_P5433	YMR006C	476.000	0.000
A_06_P5434	YMR007W	130.000	0.000

A_06_P5435	YMR008C	323.000	0.000
A_06_P5436	YMR009W	32.000	0.000
A_06_P5437	YMR010W	184.000	0.000
A_06_P5438	YMR011W	19.000	0.000
A_06_P5439	YMR012W	92.000	0.000
A_06_P5440	YMR013C	32.000	0.000
A_06_P5441	YMR013W-A	54.000	0.000
A_06_P5442	YMR014W	50.000	0.000
A_06_P5443	YMR015C	160.000	0.000
A_06_P5444	YMR016C	7.000	0.000
A_06_P5445	YMR017W	44.000	0.000
A_06_P5446	YMR018W	3.000	0.000
A_06_P5447	YMR019W	63.000	0.000
A_06_P5448	YMR020W	4.000	0.000
A_06_P5449	YMR021C	10.000	0.000
A_06_P5450	YMR022W	34.000	0.000
A_06_P5451	YMR023C	55.000	0.000
A_06_P5452	YMR024W	6.000	0.000
A_06_P5453	YMR025W	108.000	0.000
A_06_P5454	YMR026C	8.000	0.000
A_06_P5455	YMR027W	22.000	0.000
A_06_P5456	YMR028W	795.000	0.000
A_06_P5457	YMR029C	22.000	0.000
A_06_P5458	YMR030W	30.000	0.000
A_06_P5459	YMR031C	8.000	0.000
A_06_P5460	YMR031W-A	9.000	0.000
A_06_P5461	YMR032W	13.000	0.000
A_06_P5462	YMR033W	58.000	0.000
A_06_P5463	YMR034C	626.000	0.000
A_06_P5464	YMR035W	36.000	0.000
A_06_P5465	YMR036C	42.000	0.000
A_06_P5466	YMR037C	46.000	0.000
A_06_P5467	YMR038C	45.000	0.000
A_06_P5468	YMR039C	40.000	0.000
A_06_P5469	YMR040W	13.000	0.000
A_06_P5470	YMR041C	74.000	0.000
A_06_P5471	YMR042W	204.000	0.000
A_06_P5472	YMR043W	180.000	0.000
A_06_P5473	YMR044W	113.000	0.000
A_06_P5474	YCR018C-A	39.000	3.391
A_06_P5474	YGR122C-A	5.000	0.000
A_06_P5474	YMR046W-A	4.000	0.000
A_06_P5474	YPR002C-A	119.800	1.643
A_06_P5475	YMR047C	70.000	0.000
A_06_P5476	YMR048W	19.000	0.000
A_06_P5477	YMR049C	41.000	0.000
A_06_P5478	YMR052C-A	38.000	0.000
A_06_P5479	YMR052W	131.000	0.000
A_06_P5480	YMR053C	9.000	0.000
A_06_P5481	YMR054W	16.000	0.000
A_06_P5482	YMR055C	108.000	0.000
A_06_P5483	YMR056C	58.000	0.000
A_06_P5484	YMR057C	186.000	0.000
A_06_P5485	YMR058W	43.000	0.000
A_06_P5486	YMR059W	21.000	0.000

A_06_P5487	YMR060C	9.000	0.000
A_06_P5488	YMR061W	80.000	0.000
A_06_P5489	YMR062C	1000.000	0.000
A_06_P5490	YMR063W	10.000	0.000
A_06_P5491	YMR064W	32.000	0.000
A_06_P5492	YMR065W	544.000	0.000
A_06_P5493	YMR066W	120.000	0.000
A_06_P5494	YMR067C	15.000	0.000
A_06_P5495	YMR068W	93.000	0.000
A_06_P5496	YMR069W	41.000	0.000
A_06_P5497	YMR070W	48.000	0.000
A_06_P5498	YMR071C	238.000	0.000
A_06_P5499	YMR072W	388.000	0.000
A_06_P5500	YMR073C	30.000	0.000
A_06_P5501	YMR074C	43.000	0.000
A_06_P5502	YMR075C-A	299.000	0.000
A_06_P5503	YMR075W	20.000	0.000
A_06_P5504	YMR076C	502.000	0.000
A_06_P5505	YMR077C	142.000	0.000
A_06_P5506	YMR078C	113.000	0.000
A_06_P5507	YMR079W	7.000	0.000
A_06_P5508	YMR080C	44.000	0.000
A_06_P5509	YMR081C	59.000	0.000
A_06_P5510	YMR082C	46.000	0.000
A_06_P5511	YMR083W	63.000	0.000
A_06_P5512	YMR084W	44.000	0.000
A_06_P5513	YMR085W	18.000	0.000
A_06_P5514	YMR086C-A	39.000	0.000
A_06_P5515	YMR086W	12.000	0.000
A_06_P5516	YMR087W	6.000	0.000
A_06_P5517	YMR088C	14.000	0.000
A_06_P5518	YMR089C	10.000	0.000
A_06_P5519	YMR090W	127.000	0.000
A_06_P5520	YMR091C	39.000	0.000
A_06_P5521	YMR092C	86.000	0.000
A_06_P5522	YMR093W	99.000	0.000
A_06_P5523	YMR094W	23.000	0.000
A_06_P5524	YMR095C	44.000	0.000
A_06_P5525	YMR096W	177.000	0.000
A_06_P5526	YMR097C	10.000	0.000
A_06_P5527	YMR098C	344.000	0.000
A_06_P5528	YMR099C	16.000	0.000
A_06_P5529	YMR100W	36.000	0.000
A_06_P5530	YMR101C	40.000	0.000
A_06_P5531	YMR102C	46.000	0.000
A_06_P5532	YMR103C	24.000	0.000
A_06_P5533	YMR104C	24.000	0.000
A_06_P5534	YMR105C	25.000	0.000
A_06_P5535	YMR106C	28.000	0.000
A_06_P5536	YMR107W	9.000	0.000
A_06_P5537	YMR108W	47.000	0.000
A_06_P5538	YMR109W	115.000	0.000
A_06_P5539	YMR110C	38.000	0.000
A_06_P5540	YMR111C	15.000	0.000
A_06_P5541	YMR112C	92.000	0.000

A_06_P5542	YMR113W	7.000	0.000
A_06_P5543	YMR114C	31.000	0.000
A_06_P5544	YMR115W	3.000	0.000
A_06_P5545	YMR116C	10.000	0.000
A_06_P5546	YMR117C	1000.000	0.000
A_06_P5547	YMR118C	161.000	0.000
A_06_P5548	YMR119W	49.000	0.000
A_06_P5549	YMR119W-A	75.000	0.000
A_06_P5550	YMR120C	18.000	0.000
A_06_P5551	YMR121C	5.000	0.000
A_06_P5552	YMR122C	218.000	0.000
A_06_P5553	YMR122W-A	36.000	0.000
A_06_P5554	YMR123W	15.000	0.000
A_06_P5555	YMR124W	226.000	0.000
A_06_P5556	YMR125W	203.000	0.000
A_06_P5557	YMR126C	13.000	0.000
A_06_P5558	YMR127C	174.000	0.000
A_06_P5559	YMR128W	521.000	0.000
A_06_P5560	YMR129W	177.000	0.000
A_06_P5561	YMR130W	38.000	0.000
A_06_P5562	YMR131C	10.000	0.000
A_06_P5563	YMR132C	132.000	0.000
A_06_P5564	YMR133W	35.000	0.000
A_06_P5565	YMR134W	150.000	0.000
A_06_P5566	YMR135C	90.000	0.000
A_06_P5567	YMR135W-A	221.000	0.000
A_06_P5568	YMR136W	11.000	0.000
A_06_P5569	YMR137C	39.000	0.000
A_06_P5570	YMR138W	176.000	0.000
A_06_P5571	YMR139W	197.000	0.000
A_06_P5572	YMR140W	192.000	0.000
A_06_P5573	YMR141C	42.000	0.000
A_06_P5574	YMR142C	51.000	0.000
A_06_P5575	YMR143W	177.000	0.000
A_06_P5576	YMR144W	16.000	0.000
A_06_P5577	YMR145C	63.000	0.000
A_06_P5578	YMR146C	14.000	0.000
A_06_P5579	YMR147W	70.000	0.000
A_06_P5580	YMR148W	109.000	0.000
A_06_P5581	YMR149W	27.000	0.000
A_06_P5582	YMR150C	20.000	0.000
A_06_P5583	YMR151W	79.000	0.000
A_06_P5584	YMR152W	21.000	0.000
A_06_P5585	YMR153C-A	28.000	0.000
A_06_P5586	YMR153W	145.000	0.000
A_06_P5587	YMR154C	9.000	0.000
A_06_P5588	YMR155W	133.000	0.000
A_06_P5589	YMR156C	30.000	0.000
A_06_P5590	YMR157C	42.000	0.000
A_06_P5591	YMR158C-A	153.000	0.000
A_06_P5592	YMR158W	24.000	0.000
A_06_P5593	YMR158W-B	18.000	0.000
A_06_P5594	YMR159C	36.000	0.000
A_06_P5595	YMR160W	20.000	0.000
A_06_P5596	YMR161W	44.000	0.000

A_06_P5597	YMR162C	78.000	0.000
A_06_P5598	YMR163C	13.000	0.000
A_06_P5599	YMR164C	7.000	0.000
A_06_P5600	YMR165C	24.000	0.000
A_06_P5601	YMR166C	63.000	0.000
A_06_P5602	YMR167W	35.000	0.000
A_06_P5603	YMR168C	50.000	0.000
A_06_P5604	YMR169C	8.000	0.000
A_06_P5605	YMR170C	34.000	0.000
A_06_P5606	YMR171C	145.000	0.000
A_06_P5607	YMR172C-A	15.000	0.000
A_06_P5608	YMR172W	18.000	0.000
A_06_P5609	YMR173W	112.000	0.000
A_06_P5609	YMR173W-A	18.800	0.447
A_06_P5610	YMR173W-A	0.200	0.447
A_06_P5611	YMR174C	66.000	0.000
A_06_P5612	YMR175W	4.000	0.000
A_06_P5613	YMR176W	72.000	0.000
A_06_P5614	YMR177W	384.000	0.000
A_06_P5615	YMR178W	153.000	0.000
A_06_P5616	YMR179W	31.000	0.000
A_06_P5617	YMR180C	114.000	0.000
A_06_P5618	YMR181C	32.000	0.000
A_06_P5619	YMR182C	18.000	0.000
A_06_P5620	YMR183C	79.000	0.000
A_06_P5621	YMR184W	19.000	0.000
A_06_P5622	YMR185W	41.000	0.000
A_06_P5623	YMR186W	56.000	0.000
A_06_P5624	YMR187C	180.000	0.000
A_06_P5625	YMR188C	23.000	0.000
A_06_P5626	YMR189W	55.000	0.000
A_06_P5627	YMR190C	288.000	0.000
A_06_P5628	YMR191W	380.000	0.000
A_06_P5629	YMR192W	40.000	0.000
A_06_P5630	YMR193C-A	97.000	0.000
A_06_P5631	YMR193W	4.000	0.000
A_06_P5632	YMR194C-A	48.000	0.000
A_06_P5633	YMR194W	33.000	0.000
A_06_P5634	YMR195W	47.000	0.000
A_06_P5635	YMR196W	23.000	0.000
A_06_P5636	YMR197C	68.000	0.000
A_06_P5637	YMR198W	15.000	0.000
A_06_P5638	YMR199W	19.000	0.000
A_06_P5639	YMR200W	44.000	0.000
A_06_P5640	YMR201C	59.000	0.000
A_06_P5641	YMR202W	59.000	0.000
A_06_P5642	YMR203W	339.000	0.000
A_06_P5643	YMR204C	13.000	0.000
A_06_P5644	YMR205C	96.000	0.000
A_06_P5645	YMR206W	26.000	0.000
A_06_P5646	YMR207C	36.000	0.000
A_06_P5647	YMR208W	23.000	0.000
A_06_P5648	YMR209C	69.000	0.000
A_06_P5649	YMR210W	427.000	0.000
A_06_P5650	YMR211W	7.000	0.000

A_06_P5651	YMR212C	29.000	0.000
A_06_P5652	YMR213W	48.000	0.000
A_06_P5653	YMR214W	22.000	0.000
A_06_P5654	YMR215W	4.000	0.000
A_06_P5655	YMR216C	44.000	0.000
A_06_P5656	YMR217W	9.000	0.000
A_06_P5657	YMR218C	13.000	0.000
A_06_P5658	YMR219W	73.000	0.000
A_06_P5659	YMR220W	276.000	0.000
A_06_P5660	YMR221C	10.000	0.000
A_06_P5661	YMR222C	60.000	0.000
A_06_P5662	YMR223W	33.000	0.000
A_06_P5663	YMR224C	16.000	0.000
A_06_P5664	YMR225C	94.000	0.000
A_06_P5665	YMR226C	38.000	0.000
A_06_P5666	YMR227C	45.000	0.000
A_06_P5667	YMR228W	84.000	0.000
A_06_P5668	YMR229C	45.000	0.000
A_06_P5669	YMR230W	373.000	0.000
A_06_P5670	YMR231W	17.000	0.000
A_06_P5671	YMR232W	226.000	0.000
A_06_P5672	YMR233W	36.000	0.000
A_06_P5673	YMR234W	12.000	0.000
A_06_P5674	YMR235C	1000.000	0.000
A_06_P5675	YMR236W	11.000	0.000
A_06_P5676	YMR237W	117.000	0.000
A_06_P5677	YMR238W	120.000	0.000
A_06_P5678	YMR239C	8.000	0.000
A_06_P5679	YMR240C	360.000	0.000
A_06_P5680	YMR241W	139.000	0.000
A_06_P5681	YMR242C	61.000	0.000
A_06_P5682	YMR243C	94.000	0.000
A_06_P5683	YMR244C-A	334.000	0.000
A_06_P5684	YMR244W	41.000	0.000
A_06_P5685	YMR245W	11.000	0.000
A_06_P5686	YMR246W	78.000	0.000
A_06_P5687	YMR247C	35.000	0.000
A_06_P5688	YMR250W	27.000	0.000
A_06_P5689	YMR251W	38.000	0.000
A_06_P5690	YMR251W-A	75.000	0.000
A_06_P5691	YMR252C	22.000	0.000
A_06_P5692	YMR253C	3.000	0.000
A_06_P5693	YMR254C	8.000	0.000
A_06_P5694	YMR255W	37.000	0.000
A_06_P5695	YMR256C	123.000	0.000
A_06_P5696	YMR257C	820.000	0.000
A_06_P5697	YMR258C	62.000	0.000
A_06_P5698	YMR259C	19.000	0.000
A_06_P5699	YMR260C	14.000	0.000
A_06_P5700	YMR261C	9.000	0.000
A_06_P5701	YMR262W	17.000	0.000
A_06_P5702	YMR263W	9.000	0.000
A_06_P5703	YMR264W	6.000	0.000
A_06_P5704	YMR265C	306.000	0.000
A_06_P5705	YMR266W	7.000	0.000

A_06_P5706	YMR267W	61.000	0.000
A_06_P5707	YMR268C	15.000	0.000
A_06_P5708	YMR269W	70.000	0.000
A_06_P5709	YMR270C	43.000	0.000
A_06_P5710	YMR271C	176.000	0.000
A_06_P5711	YMR272C	384.000	0.000
A_06_P5712	YMR273C	42.000	0.000
A_06_P5713	YMR274C	74.000	0.000
A_06_P5714	YMR275C	418.000	0.000
A_06_P5715	YMR276W	20.000	0.000
A_06_P5716	YMR277W	76.000	0.000
A_06_P5717	YMR278W	11.000	0.000
A_06_P5718	YMR279C	256.000	0.000
A_06_P5719	YMR280C	12.000	0.000
A_06_P5720	YMR281W	68.000	0.000
A_06_P5721	YMR282C	133.000	0.000
A_06_P5722	YMR283C	17.000	0.000
A_06_P5723	YMR284W	2.000	0.000
A_06_P5724	YMR285C	94.000	0.000
A_06_P5725	YMR286W	223.000	0.000
A_06_P5726	YMR287C	132.000	0.000
A_06_P5727	YMR288W	246.000	0.000
A_06_P5728	YMR289W	24.000	0.000
A_06_P5729	YMR290C	44.000	0.000
A_06_P5730	YMR290W-A	54.000	0.000
A_06_P5731	YMR291W	10.000	0.000
A_06_P5732	YMR292W	105.000	0.000
A_06_P5733	YMR293C	94.000	0.000
A_06_P5734	YMR294W	48.000	0.000
A_06_P5735	YMR294W-A	13.000	0.000
A_06_P5736	YMR295C	222.000	0.000
A_06_P5737	YMR296C	37.000	0.000
A_06_P5738	YMR297W	28.000	0.000
A_06_P5739	YMR298W	215.000	0.000
A_06_P5740	YMR299C	23.000	0.000
A_06_P5741	YMR300C	104.000	0.000
A_06_P5742	YMR301C	30.000	0.000
A_06_P5743	YMR302C	13.000	0.000
A_06_P5744	YMR303C	314.000	0.000
A_06_P5745	YMR304C-A	24.000	0.000
A_06_P5746	YMR304W	35.000	0.000
A_06_P5747	YMR305C	7.000	0.000
A_06_P5748	YMR306C-A	25.000	0.000
A_06_P5749	YMR306W	29.000	0.000
A_06_P5750	YMR307W	32.000	0.000
A_06_P5751	YMR308C	15.000	0.000
A_06_P5752	YMR309C	17.000	0.000
A_06_P5753	YMR310C	39.000	0.000
A_06_P5754	YMR311C	94.000	0.000
A_06_P5755	YMR312W	90.000	0.000
A_06_P5756	YMR313C	19.000	0.000
A_06_P5757	YMR314W	15.000	0.000
A_06_P5758	YMR315W	99.000	0.000
A_06_P5759	YMR316C-A	111.000	0.000
A_06_P5760	YMR316C-B	23.000	0.000

A_06_P5761	YMR316W	113.000	0.000
A_06_P5762	YMR317W	87.000	0.000
A_06_P5763	YMR318C	37.000	0.000
A_06_P5764	YMR319C	17.000	0.000
A_06_P5765	YMR320W	14.000	0.000
A_06_P5766	YMR321C	37.000	0.000
A_06_P5766	YPL273W	26.400	3.782
A_06_P5767	YMR322C	105.000	1.000
A_06_P5767	YOR391C	1.200	1.095
A_06_P5767	YPL280W	10.600	3.847
A_06_P5768	YMR323W	11.600	2.793
A_06_P5769	YMR324C	9.000	0.000
A_06_P5770	YCR104W	0.200	0.447
A_06_P5770	YMR325W	137.000	1.225
A_06_P5771	YMR326C	49.000	0.000
A_06_P5772	YNL001W	12.000	0.000
A_06_P5773	YNL002C	119.000	0.000
A_06_P5774	YNL003C	33.000	0.000
A_06_P5775	YNL004W	126.000	0.000
A_06_P5776	YNL005C	96.000	0.000
A_06_P5777	YNL006W	126.000	0.000
A_06_P5778	YNL007C	53.000	0.000
A_06_P5779	YNL008C	53.000	0.000
A_06_P5780	YNL009W	45.000	0.000
A_06_P5781	YNL010W	14.000	0.000
A_06_P5782	YNL011C	39.000	0.000
A_06_P5783	YNL012W	6.000	0.000
A_06_P5784	YNL013C	24.000	0.000
A_06_P5785	YNL014W	20.000	0.000
A_06_P5786	YNL015W	150.000	0.000
A_06_P5787	YNL016W	11.000	0.000
A_06_P5788	YNL017C	10.000	0.000
A_06_P5789	YNL018C	21.000	0.000
A_06_P5790	YNL019C	133.600	4.037
A_06_P5790	YNL033W	0.600	0.548
A_06_P5791	YNL020C	779.000	0.000
A_06_P5792	YNL021W	25.000	0.000
A_06_P5793	YNL022C	12.000	0.000
A_06_P5794	YNL023C	44.000	0.000
A_06_P5795	YNL024C	176.000	0.000
A_06_P5796	YNL025C	14.000	0.000
A_06_P5797	YNL026W	346.000	0.000
A_06_P5798	YNL027W	42.000	0.000
A_06_P5799	YNL028W	8.000	0.000
A_06_P5800	YNL029C	15.000	0.000
A_06_P5801	YNL030W	119.800	1.483
A_06_P5802	YNL031C	21.000	0.000
A_06_P5803	YNL032W	7.000	0.000
A_06_P5804	YNL019C	22.400	4.037
A_06_P5804	YNL033W	48.400	0.548
A_06_P5805	YNL034W	2.000	0.000
A_06_P5806	YNL035C	6.000	0.000
A_06_P5807	YNL036W	79.000	0.000
A_06_P5808	YNL037C	33.000	0.000
A_06_P5809	YNL038W	12.000	0.000

A_06_P5810	YNL039W	41.000	0.000
A_06_P5811	YNL040W	105.000	0.000
A_06_P5812	YNL041C	19.000	0.000
A_06_P5813	YNL042W	143.000	0.000
A_06_P5814	YNL043C	40.000	0.000
A_06_P5815	YNL044W	59.000	0.000
A_06_P5816	YNL045W	11.000	0.000
A_06_P5817	YNL046W	65.000	0.000
A_06_P5818	YNL047C	21.000	0.000
A_06_P5819	YNL048W	45.000	0.000
A_06_P5820	YNL049C	74.000	0.000
A_06_P5821	YNL050C	90.000	0.000
A_06_P5822	YNL051W	21.000	0.000
A_06_P5823	YNL052W	71.000	0.000
A_06_P5824	YNL053W	50.000	0.000
A_06_P5825	YNL054W	78.000	0.000
A_06_P5826	YNL055C	75.000	0.000
A_06_P5827	YNL056W	74.000	0.000
A_06_P5828	YNL057W	19.000	0.000
A_06_P5829	YNL058C	5.000	0.000
A_06_P5830	YNL059C	35.000	0.000
A_06_P5831	YNL061W	25.000	0.000
A_06_P5832	YNL062C	54.000	0.000
A_06_P5833	YNL063W	13.000	0.000
A_06_P5834	YNL064C	44.000	0.000
A_06_P5835	YNL065W	75.000	0.000
A_06_P5836	YNL066W	77.000	0.000
A_06_P5837	YNL067W	35.000	0.000
A_06_P5838	YNL067W-A	139.000	0.000
A_06_P5839	YNL068C	8.000	0.000
A_06_P5840	YNL069C	55.000	0.000
A_06_P5841	YNL070W	46.000	0.000
A_06_P5842	YNL071W	86.000	0.000
A_06_P5843	YNL072W	34.000	0.000
A_06_P5844	YNL073W	287.000	0.000
A_06_P5845	YNL074C	47.000	0.000
A_06_P5846	YNL075W	22.000	0.000
A_06_P5847	YNL076W	11.000	0.000
A_06_P5848	YNL077W	165.000	0.000
A_06_P5849	YNL078W	41.000	0.000
A_06_P5850	YNL079C	98.000	0.000
A_06_P5851	YNL080C	37.000	0.000
A_06_P5852	YNL081C	91.000	0.000
A_06_P5853	YNL082W	57.000	0.000
A_06_P5854	YNL083W	170.000	0.000
A_06_P5855	YNL084C	47.000	0.000
A_06_P5856	YNL085W	8.000	0.000
A_06_P5857	YNL086W	522.000	0.000
A_06_P5858	YNL087W	68.000	0.000
A_06_P5859	YNL088W	475.000	0.000
A_06_P5860	YNL089C	135.000	0.000
A_06_P5861	YNL090W	43.000	0.000
A_06_P5862	YNL091W	8.000	0.000
A_06_P5863	YNL092W	26.000	0.000
A_06_P5864	YNL093W	96.000	0.000

A_06_P5865	YNL094W	5.000	0.000
A_06_P5866	YNL095C	14.000	0.000
A_06_P5867	YNL096C	46.000	0.000
A_06_P5868	YNL097C	42.000	0.000
A_06_P5869	YNL098C	84.000	0.000
A_06_P5870	YNL099C	126.000	0.000
A_06_P5871	YNL100W	54.000	0.000
A_06_P5872	YNL101W	44.000	0.000
A_06_P5873	YNL102W	48.000	0.000
A_06_P5874	YNL103W	27.000	0.000
A_06_P5875	YNL104C	229.000	0.000
A_06_P5876	YNL105W	15.000	0.000
A_06_P5877	YNL106C	67.000	0.000
A_06_P5878	YNL107W	100.000	0.000
A_06_P5879	YNL108C	13.000	0.000
A_06_P5880	YNL109W	157.000	0.000
A_06_P5881	YNL110C	60.000	0.000
A_06_P5882	YNL111C	23.000	0.000
A_06_P5883	YNL112W	126.000	0.000
A_06_P5884	YNL113W	69.000	0.000
A_06_P5885	YNL114C	142.000	0.000
A_06_P5886	YNL115C	20.000	0.000
A_06_P5887	YNL116W	76.000	0.000
A_06_P5888	YNL117W	9.000	0.000
A_06_P5889	YNL118C	42.000	0.000
A_06_P5890	YNL119W	85.000	0.000
A_06_P5891	YNL120C	4.000	0.000
A_06_P5892	YNL121C	82.000	0.000
A_06_P5893	YNL122C	106.000	0.000
A_06_P5894	YNL123W	32.000	0.000
A_06_P5895	YNL124W	7.000	0.000
A_06_P5896	YNL125C	221.000	0.000
A_06_P5897	YNL126W	29.000	0.000
A_06_P5898	YNL127W	5.000	0.000
A_06_P5899	YNL128W	110.000	0.000
A_06_P5900	YNL129W	57.000	0.000
A_06_P5901	YNL130C	66.000	0.000
A_06_P5902	YNL131W	39.000	0.000
A_06_P5903	YNL132W	30.000	0.000
A_06_P5904	YNL133C	51.000	0.000
A_06_P5905	YNL134C	559.000	0.000
A_06_P5906	YNL135C	388.000	0.000
A_06_P5907	YNL136W	216.000	0.000
A_06_P5908	YNL137C	42.000	0.000
A_06_P5909	YNL138W	94.000	0.000
A_06_P5911	YNL139C	39.000	0.000
A_06_P5911	YNL140C	20.000	0.000
A_06_P5912	YNL141W	30.000	0.000
A_06_P5913	YNL142W	18.000	0.000
A_06_P5914	YNL143C	248.000	0.000
A_06_P5915	YNL144C	31.000	0.000
A_06_P5916	YNL145W	28.000	0.000
A_06_P5917	YNL146W	47.000	0.000
A_06_P5918	YNL147W	35.000	0.000
A_06_P5919	YNL148C	85.000	0.000

A_06_P5920	YNL149C	97.000	0.000
A_06_P5921	YNL150W	19.000	0.000
A_06_P5922	YNL151C	22.000	0.000
A_06_P5923	YNL152W	105.000	0.000
A_06_P5924	YNL153C	22.000	0.000
A_06_P5925	YNL154C	405.000	0.000
A_06_P5926	YNL155W	92.000	0.000
A_06_P5927	YNL156C	210.000	0.000
A_06_P5928	YNL157W	16.000	0.000
A_06_P5929	YNL158W	22.000	0.000
A_06_P5930	YNL159C	79.000	0.000
A_06_P5931	YNL160W	7.000	0.000
A_06_P5932	YNL161W	43.000	0.000
A_06_P5933	YHR141C	3.600	1.517
A_06_P5933	YNL162W	49.000	0.000
A_06_P5934	YNL162W-A	29.000	0.000
A_06_P5935	YNL163C	303.000	0.000
A_06_P5936	YNL164C	34.000	0.000
A_06_P5937	YNL165W	976.000	0.000
A_06_P5938	YNL166C	6.000	0.000
A_06_P5939	YNL167C	27.000	0.000
A_06_P5940	YNL168C	24.000	0.000
A_06_P5941	YNL169C	9.000	0.000
A_06_P5942	YNL170W	7.000	0.000
A_06_P5943	YNL171C	797.000	0.000
A_06_P5944	YNL172W	339.000	0.000
A_06_P5945	YNL173C	27.000	0.000
A_06_P5946	YNL174W	583.000	0.000
A_06_P5947	YNL175C	37.000	0.000
A_06_P5948	YNL176C	18.000	0.000
A_06_P5949	YNL177C	108.000	0.000
A_06_P5950	YNL178W	252.000	0.000
A_06_P5951	YNL179C	103.000	0.000
A_06_P5952	YNL180C	7.000	0.000
A_06_P5953	YNL181W	8.000	0.000
A_06_P5954	YNL182C	40.000	0.000
A_06_P5955	YNL183C	97.000	0.000
A_06_P5956	YNL184C	73.000	0.000
A_06_P5957	YNL185C	156.000	0.000
A_06_P5958	YNL186W	47.000	0.000
A_06_P5959	YNL187W	175.000	0.000
A_06_P5960	YNL188W	58.000	0.000
A_06_P5961	YNL189W	36.000	0.000
A_06_P5962	YNL190W	18.000	0.000
A_06_P5963	YNL191W	12.000	0.000
A_06_P5964	YNL192W	900.000	0.000
A_06_P5965	YNL193W	19.000	0.000
A_06_P5966	YNL194C	188.000	0.000
A_06_P5967	YNL195C	24.000	0.000
A_06_P5968	YNL196C	72.000	0.000
A_06_P5969	YNL197C	624.000	0.000
A_06_P5970	YNL198C	85.000	0.000
A_06_P5971	YNL199C	318.000	0.000
A_06_P5972	YNL200C	29.000	0.000
A_06_P5973	YNL201C	22.000	0.000

A_06_P5974	YNL202W	42.000	0.000
A_06_P5975	YNL203C	145.000	0.000
A_06_P5976	YNL204C	326.000	0.000
A_06_P5977	YNL205C	767.000	0.000
A_06_P5978	YNL206C	43.000	0.000
A_06_P5979	YNL207W	18.000	0.000
A_06_P5980	YNL208W	55.000	0.000
A_06_P5981	YNL209W	109.000	0.000
A_06_P5982	YNL210W	57.000	0.000
A_06_P5983	YNL211C	39.000	0.000
A_06_P5984	YNL212W	54.000	0.000
A_06_P5985	YNL213C	155.000	0.000
A_06_P5986	YNL214W	288.000	0.000
A_06_P5987	YNL215W	7.000	0.000
A_06_P5988	YNL216W	134.000	0.000
A_06_P5989	YNL217W	28.000	0.000
A_06_P5990	YNL218W	90.000	0.000
A_06_P5991	YNL219C	54.000	0.000
A_06_P5992	YNL220W	345.000	0.000
A_06_P5993	YNL221C	116.000	0.000
A_06_P5994	YNL222W	127.000	0.000
A_06_P5995	YNL223W	130.000	0.000
A_06_P5996	YNL224C	148.000	0.000
A_06_P5997	YNL225C	175.000	0.000
A_06_P5998	YNL226W	53.000	0.000
A_06_P5999	YNL227C	57.000	0.000
A_06_P6000	YNL228W	100.000	0.000
A_06_P6001	YNL229C	61.000	0.000
A_06_P6002	YNL230C	98.000	0.000
A_06_P6003	YNL231C	35.000	0.000
A_06_P6004	YNL232W	192.000	0.000
A_06_P6005	YNL233W	101.000	0.000
A_06_P6006	YNL234W	324.000	0.000
A_06_P6007	YNL235C	484.000	0.000
A_06_P6008	YNL236W	121.000	0.000
A_06_P6009	YNL237W	59.000	0.000
A_06_P6010	YNL238W	50.000	0.000
A_06_P6011	YNL239W	204.000	0.000
A_06_P6012	YNL240C	3.000	0.000
A_06_P6013	YNL241C	11.000	0.000
A_06_P6014	YNL242W	125.000	0.000
A_06_P6015	YNL243W	83.000	0.000
A_06_P6016	YNL244C	93.000	0.000
A_06_P6017	YNL245C	55.000	0.000
A_06_P6018	YNL246W	38.000	0.000
A_06_P6019	YNL247W	560.000	0.000
A_06_P6020	YNL248C	18.000	0.000
A_06_P6021	YNL249C	11.000	0.000
A_06_P6022	YNL250W	43.000	0.000
A_06_P6023	YNL251C	96.000	0.000
A_06_P6024	YNL252C	23.000	0.000
A_06_P6025	YNL253W	30.000	0.000
A_06_P6026	YNL254C	76.000	0.000
A_06_P6027	YNL255C	25.000	0.000
A_06_P6028	YNL256W	12.000	0.000

A_06_P6029	YNL257C	29.000	0.000
A_06_P6030	YNL258C	15.000	0.000
A_06_P6031	YNL259C	24.000	0.000
A_06_P6032	YNL260C	27.000	0.000
A_06_P6033	YNL261W	39.000	0.000
A_06_P6034	YNL262W	28.000	0.000
A_06_P6035	YNL263C	17.000	0.000
A_06_P6036	YNL264C	32.000	0.000
A_06_P6037	YNL265C	122.000	0.000
A_06_P6038	YNL266W	23.000	0.000
A_06_P6039	YNL267W	276.000	0.000
A_06_P6040	YNL268W	108.000	0.000
A_06_P6041	YNL269W	238.000	0.000
A_06_P6042	YNL270C	9.000	0.000
A_06_P6043	YNL271C	25.000	0.000
A_06_P6044	YNL272C	29.000	0.000
A_06_P6045	YNL273W	113.000	0.000
A_06_P6046	YNL274C	15.000	0.000
A_06_P6047	YNL275W	4.000	0.000
A_06_P6048	YNL276C	86.000	0.000
A_06_P6049	YNL277W	2.000	0.000
A_06_P6050	YNL278W	29.000	0.000
A_06_P6051	YNL279W	29.000	0.000
A_06_P6052	YNL280C	472.000	0.000
A_06_P6053	YNL281W	255.000	0.000
A_06_P6054	YNL282W	25.000	0.000
A_06_P6055	YNL283C	43.000	0.000
A_06_P6056	YNL284C	57.000	0.000
A_06_P6057	YNL285W	13.000	0.000
A_06_P6058	YNL286W	3.000	0.000
A_06_P6059	YNL287W	73.000	0.000
A_06_P6060	YNL288W	423.000	0.000
A_06_P6061	YNL289W	37.000	0.000
A_06_P6062	YNL290W	16.000	0.000
A_06_P6063	YNL291C	39.000	0.000
A_06_P6064	YNL292W	22.000	0.000
A_06_P6065	YNL293W	15.000	0.000
A_06_P6066	YNL294C	186.000	0.000
A_06_P6067	YNL295W	19.000	0.000
A_06_P6068	YNL296W	80.000	0.000
A_06_P6069	YNL297C	202.000	0.000
A_06_P6070	YNL298W	111.000	0.000
A_06_P6071	YNL299W	185.000	0.000
A_06_P6072	YNL300W	23.000	0.000
A_06_P6073	YNL301C	124.000	0.000
A_06_P6074	YNL302C	5.000	0.000
A_06_P6075	YNL303W	9.000	0.000
A_06_P6076	YNL304W	52.000	0.000
A_06_P6077	YNL305C	61.000	0.000
A_06_P6078	YNL306W	185.000	0.000
A_06_P6079	YNL307C	72.000	0.000
A_06_P6080	YNL308C	187.000	0.000
A_06_P6081	YNL309W	107.000	0.000
A_06_P6082	YNL310C	29.000	0.000
A_06_P6083	YNL311C	32.000	0.000

A_06_P6084	YNL312W	11.000	0.000
A_06_P6085	YNL313C	16.000	0.000
A_06_P6086	YNL314W	23.000	0.000
A_06_P6087	YNL315C	51.000	0.000
A_06_P6088	YNL316C	79.000	0.000
A_06_P6089	YNL317W	1000.000	0.000
A_06_P6090	YNL318C	98.000	0.000
A_06_P6091	YNL319W	12.000	0.000
A_06_P6092	YNL320W	6.000	0.000
A_06_P6093	YNL321W	46.000	0.000
A_06_P6094	YNL322C	15.000	0.000
A_06_P6095	YNL323W	504.000	0.000
A_06_P6096	YNL324W	119.000	0.000
A_06_P6097	YNL325C	79.000	0.000
A_06_P6098	YNL326C	3.000	0.000
A_06_P6099	YNL327W	94.000	0.000
A_06_P6100	YNL328C	88.000	0.000
A_06_P6101	YNL329C	662.000	0.000
A_06_P6102	YNL330C	89.000	0.000
A_06_P6103	YNL331C	178.000	0.000
A_06_P6104	YFL058W	3.600	1.140
A_06_P6104	YNL332W	103.200	3.114
A_06_P6105	YNL333W	103.200	5.404
A_06_P6106	YNL334C	107.000	1.732
A_06_P6107	YFL061W	4.200	2.588
A_06_P6107	YNL335W	36.000	1.000
A_06_P6108	YNL336W	42.600	0.548
A_06_P6109	YDR543C	0.200	0.447
A_06_P6109	YJR162C	160.000	17.132
A_06_P6109	YNL337W	9.000	0.000
A_06_P6111	YNR001C	26.000	0.000
A_06_P6112	YNR001W-A	14.000	0.000
A_06_P6113	YNR002C	167.000	0.000
A_06_P6114	YNR003C	42.000	0.000
A_06_P6115	YNR004W	21.000	0.000
A_06_P6116	YNR005C	11.000	0.000
A_06_P6117	YNR006W	115.000	0.000
A_06_P6118	YNR007C	32.000	0.000
A_06_P6119	YNR008W	83.000	0.000
A_06_P6120	YNR009W	42.000	0.000
A_06_P6121	YNR010W	25.000	0.000
A_06_P6122	YNR011C	97.000	0.000
A_06_P6123	YNR012W	11.000	0.000
A_06_P6124	YNR013C	8.000	0.000
A_06_P6125	YNR014W	100.000	0.000
A_06_P6126	YNR015W	184.000	0.000
A_06_P6127	YNR016C	25.000	0.000
A_06_P6128	YNR017W	68.000	0.000
A_06_P6129	YNR018W	4.000	0.000
A_06_P6130	YNR019W	7.000	0.000
A_06_P6131	YNR020C	9.000	0.000
A_06_P6132	YNR021W	80.000	0.000
A_06_P6133	YNR022C	461.000	0.000
A_06_P6134	YNR023W	18.000	0.000
A_06_P6135	YNR024W	98.000	0.000

A_06_P6136	YNR025C	76.000	0.000
A_06_P6137	YNR026C	391.000	0.000
A_06_P6138	YNR027W	70.000	0.000
A_06_P6139	YNR028W	143.000	0.000
A_06_P6140	YNR029C	9.000	0.000
A_06_P6141	YNR030W	12.000	0.000
A_06_P6142	YNR031C	93.000	0.000
A_06_P6143	YJL181W	0.200	0.447
A_06_P6143	YNR032C-A	82.000	0.000
A_06_P6144	YNR032W	16.000	0.000
A_06_P6145	YNR033W	156.000	0.000
A_06_P6146	YNR034W	67.000	0.000
A_06_P6147	YNR034W-A	34.000	0.000
A_06_P6148	YNR035C	5.000	0.000
A_06_P6149	YNR036C	26.000	0.000
A_06_P6150	YNR037C	22.000	0.000
A_06_P6151	YNR038W	43.000	0.000
A_06_P6152	YNR039C	18.000	0.000
A_06_P6153	YNR040W	58.000	0.000
A_06_P6154	YNR041C	24.000	0.000
A_06_P6155	YNR042W	95.000	0.000
A_06_P6156	YNR043W	15.000	0.000
A_06_P6157	YNR044W	20.000	0.000
A_06_P6158	YNR045W	34.000	0.000
A_06_P6159	YNR046W	30.000	0.000
A_06_P6160	YNR047W	88.000	0.000
A_06_P6161	YNR048W	98.000	0.000
A_06_P6162	YNR049C	10.000	0.000
A_06_P6163	YNR050C	21.000	0.000
A_06_P6164	YNR051C	11.000	0.000
A_06_P6165	YNR052C	23.000	0.000
A_06_P6166	YNR053C	463.000	0.000
A_06_P6167	YNR054C	193.000	0.000
A_06_P6168	YNR055C	64.000	0.000
A_06_P6169	YNR056C	23.000	0.000
A_06_P6170	YNR057C	38.000	0.000
A_06_P6171	YNR058W	5.000	0.000
A_06_P6172	YNR059W	140.000	0.000
A_06_P6173	YNR060W	22.000	0.000
A_06_P6174	YNR061C	14.000	0.000
A_06_P6175	YNR062C	157.000	0.000
A_06_P6176	YNR063W	148.000	0.000
A_06_P6177	YNR064C	127.000	0.000
A_06_P6178	YNR065C	67.000	0.000
A_06_P6179	YNR066C	37.000	0.000
A_06_P6180	YNR067C	92.000	0.000
A_06_P6181	YNR068C	8.000	0.000
A_06_P6182	YNR069C	48.000	0.000
A_06_P6183	YNR070W	209.000	0.000
A_06_P6184	YNR071C	17.000	0.000
A_06_P6185	YDL245C	0.400	0.548
A_06_P6185	YEL069C	0.200	0.447
A_06_P6185	YJR158W	0.800	0.837
A_06_P6185	YNR072W	21.000	0.000
A_06_P6186	YEL070W	1.400	0.548

A_06_P6186	YNR073C	13.000	0.000
A_06_P6187	YNR074C	6.000	0.000
A_06_P6188	YNR075W	85.000	0.000
A_06_P6189	YLL064C	244.400	6.025
A_06_P6189	YLR461W	0.200	0.447
A_06_P6189	YNR076W	48.200	4.087
A_06_P6190	YAL068W-A	47.400	1.140
A_06_P6190	YER188C-A	6.000	2.000
A_06_P6190	YJR162C	2.400	1.673
A_06_P6190	YNR077C	42.400	0.894
A_06_P6191	YOL001W	125.000	0.000
A_06_P6192	YOL002C	27.000	0.000
A_06_P6193	YOL003C	16.000	0.000
A_06_P6194	YOL004W	50.000	0.000
A_06_P6195	YOL005C	19.000	0.000
A_06_P6196	YOL006C	230.000	0.000
A_06_P6197	YOL007C	10.000	0.000
A_06_P6198	YOL008W	114.000	0.000
A_06_P6199	YOL009C	23.000	0.000
A_06_P6200	YOL010W	243.000	0.000
A_06_P6201	YOL011W	110.000	0.000
A_06_P6202	YOL012C	25.000	0.000
A_06_P6203	YOL013C	93.000	0.000
A_06_P6204	YOL013W-B	70.000	0.000
A_06_P6205	YOL014W	53.000	0.000
A_06_P6206	YOL015W	232.000	0.000
A_06_P6207	YOL016C	16.000	0.000
A_06_P6208	YOL017W	25.000	0.000
A_06_P6209	YOL018C	351.000	0.000
A_06_P6210	YOL019W	7.000	0.000
A_06_P6211	YOL020W	38.000	0.000
A_06_P6212	YOL021C	17.000	0.000
A_06_P6213	YOL022C	109.000	0.000
A_06_P6214	YOL023W	128.000	0.000
A_06_P6215	YOL024W	10.000	0.000
A_06_P6216	YOL025W	83.000	0.000
A_06_P6217	YOL026C	48.000	0.000
A_06_P6218	YOL027C	27.000	0.000
A_06_P6219	YOL028C	465.000	0.000
A_06_P6220	YOL029C	10.000	0.000
A_06_P6221	YOL030W	6.000	0.000
A_06_P6222	YOL031C	6.000	0.000
A_06_P6223	YOL032W	8.000	0.000
A_06_P6224	YOL033W	339.000	0.000
A_06_P6225	YOL034W	23.000	0.000
A_06_P6226	YOL035C	26.000	0.000
A_06_P6227	YOL036W	169.000	0.000
A_06_P6228	YOL037C	13.000	0.000
A_06_P6229	YOL038W	87.000	0.000
A_06_P6230	YOL039W	159.000	0.000
A_06_P6231	YOL040C	252.000	0.000
A_06_P6232	YOL041C	16.000	0.000
A_06_P6233	YOL042W	49.000	0.000
A_06_P6234	YOL043C	351.000	0.000
A_06_P6235	YOL044W	35.000	0.000

A_06_P6236	YOL045W	17.000	0.000
A_06_P6237	YOL046C	9.000	0.000
A_06_P6238	YOL047C	62.000	0.000
A_06_P6239	YOL048C	131.000	0.000
A_06_P6240	YOL049W	10.000	0.000
A_06_P6241	YOL050C	36.000	0.000
A_06_P6242	YOL051W	5.000	0.000
A_06_P6243	YOL052C	426.000	0.000
A_06_P6244	YOL052C-A	29.000	0.000
A_06_P6245	YOL053W	39.000	0.000
A_06_P6246	YOL054W	89.000	0.000
A_06_P6247	YOL055C	16.000	0.000
A_06_P6248	YOL056W	110.000	0.000
A_06_P6249	YOL057W	807.000	0.000
A_06_P6250	YOL058W	24.000	0.000
A_06_P6251	YOL059W	93.000	0.000
A_06_P6252	YOL060C	45.000	0.000
A_06_P6253	YOL061W	43.000	0.000
A_06_P6254	YOL062C	52.000	0.000
A_06_P6255	YOL063C	34.000	0.000
A_06_P6256	YOL064C	57.000	0.000
A_06_P6257	YOL065C	8.000	0.000
A_06_P6258	YOL066C	24.000	0.000
A_06_P6259	YOL067C	7.000	0.000
A_06_P6260	YOL068C	23.000	0.000
A_06_P6261	YOL069W	1000.000	0.000
A_06_P6262	YOL070C	5.000	0.000
A_06_P6263	YOL071W	150.000	0.000
A_06_P6264	YOL072W	13.000	0.000
A_06_P6265	YOL073C	19.000	0.000
A_06_P6266	YOL075C	8.000	0.000
A_06_P6267	YOL076W	108.000	0.000
A_06_P6268	YOL077C	41.000	0.000
A_06_P6269	YOL077W-A	38.000	0.000
A_06_P6270	YOL078W	186.000	0.000
A_06_P6271	YOL079W	224.000	0.000
A_06_P6272	YOL080C	24.000	0.000
A_06_P6273	YOL081W	247.000	0.000
A_06_P6274	YOL082W	148.000	0.000
A_06_P6275	YOL083W	12.000	0.000
A_06_P6276	YOL084W	44.000	0.000
A_06_P6277	YOL085C	119.000	0.000
A_06_P6278	YOL086C	31.000	0.000
A_06_P6279	YOL086W-A	145.000	0.000
A_06_P6280	YOL087C	23.000	0.000
A_06_P6281	YOL088C	182.000	0.000
A_06_P6282	YOL089C	42.000	0.000
A_06_P6283	YOL090W	17.000	0.000
A_06_P6284	YOL091W	54.000	0.000
A_06_P6285	YOL092W	8.000	0.000
A_06_P6286	YOL093W	95.000	0.000
A_06_P6287	YOL094C	347.000	0.000
A_06_P6288	YOL095C	89.000	0.000
A_06_P6289	YOL096C	243.000	0.000
A_06_P6290	YOL097C	68.000	0.000

A_06_P6291	YOL098C	170.000	0.000
A_06_P6292	YOL099C	44.000	0.000
A_06_P6293	YOL100W	9.000	0.000
A_06_P6294	YOL101C	38.000	0.000
A_06_P6295	YOL102C	39.000	0.000
A_06_P6296	YOL103W	65.000	0.000
A_06_P6297	YOL104C	128.000	0.000
A_06_P6298	YOL105C	35.000	0.000
A_06_P6299	YOL106W	24.000	0.000
A_06_P6300	YOL107W	14.000	0.000
A_06_P6301	YOL108C	14.000	0.000
A_06_P6302	YOL109W	119.000	0.000
A_06_P6303	YOL110W	30.000	0.000
A_06_P6304	YOL111C	42.000	0.000
A_06_P6305	YOL112W	37.000	0.000
A_06_P6306	YOL113W	43.000	0.000
A_06_P6307	YOL114C	51.000	0.000
A_06_P6308	YOL115W	31.000	0.000
A_06_P6309	YOL116W	143.000	0.000
A_06_P6310	YOL117W	38.000	0.000
A_06_P6311	YOL118C	34.000	0.000
A_06_P6312	YOL119C	16.000	0.000
A_06_P6313	YOL120C	174.000	0.000
A_06_P6314	YOL121C	70.000	0.000
A_06_P6315	YOL122C	199.000	0.000
A_06_P6316	YOL123W	5.000	0.000
A_06_P6317	YOL124C	45.000	0.000
A_06_P6318	YOL125W	1000.000	0.000
A_06_P6319	YOL126C	23.000	0.000
A_06_P6320	YOL127W	123.000	0.000
A_06_P6321	YOL128C	37.000	0.000
A_06_P6322	YOL129W	70.000	0.000
A_06_P6323	YOL130W	5.000	0.000
A_06_P6324	YOL131W	66.000	0.000
A_06_P6325	YOL132W	29.000	0.000
A_06_P6326	YOL133W	5.000	0.000
A_06_P6327	YOL134C	28.000	0.000
A_06_P6328	YOL135C	36.000	0.000
A_06_P6329	YOL136C	108.000	0.000
A_06_P6330	YOL137W	35.000	0.000
A_06_P6331	YOL138C	44.000	0.000
A_06_P6332	YOL139C	90.000	0.000
A_06_P6333	YOL140W	151.000	0.000
A_06_P6334	YOL141W	3.000	0.000
A_06_P6335	YOL142W	81.000	0.000
A_06_P6336	YOL143C	32.000	0.000
A_06_P6337	YOL144W	199.000	0.000
A_06_P6338	YOL145C	121.000	0.000
A_06_P6339	YOL146W	50.000	0.000
A_06_P6340	YOL147C	4.000	0.000
A_06_P6341	YOL148C	114.000	0.000
A_06_P6342	YOL149W	42.000	0.000
A_06_P6343	YOL150C	20.000	0.000
A_06_P6344	YOL151W	87.000	0.000
A_06_P6345	YOL152W	25.000	0.000

A_06_P6346	YOL153C	60.000	0.000
A_06_P6347	YOL154W	103.000	0.000
A_06_P6348	YOL155C	10.000	0.707
A_06_P6349	YOL156W	28.000	0.000
A_06_P6351	YOL158C	236.000	0.000
A_06_P6352	YOL159C	38.000	0.000
A_06_P6353	YOL159C-A	87.000	0.000
A_06_P6354	YOL160W	130.000	0.000
A_06_P6355	YLL064C	0.200	0.447
A_06_P6355	YLR461W	11.600	4.450
A_06_P6355	YOL161C	4.400	2.302
A_06_P6356	YOL162W	11.000	0.000
A_06_P6357	YOL163W	247.000	0.000
A_06_P6358	YOL164W	57.000	0.000
A_06_P6359	YOL165C	41.000	0.000
A_06_P6360	YOL166C	52.000	0.000
A_06_P6361	YOR001W	23.000	0.000
A_06_P6362	YOR002W	71.000	0.000
A_06_P6363	YOR003W	93.000	0.000
A_06_P6364	YOR004W	11.000	0.000
A_06_P6365	YOR005C	18.000	0.000
A_06_P6366	YOR006C	26.000	0.000
A_06_P6367	YOR007C	148.000	0.000
A_06_P6368	YOR008C	10.000	0.000
A_06_P6369	YOR008C-A	5.000	0.000
A_06_P6370	YOR008W-B	200.000	0.000
A_06_P6371	YOR009W	20.000	0.000
A_06_P6372	YOR010C	14.000	0.000
A_06_P6373	YOR011W	106.000	0.000
A_06_P6374	YOR012W	56.000	0.000
A_06_P6375	YOR013W	80.000	0.000
A_06_P6376	YOR014W	261.000	0.000
A_06_P6377	YOR015W	35.000	0.000
A_06_P6378	YOR016C	45.000	0.000
A_06_P6379	YOR017W	25.000	0.000
A_06_P6380	YOR018W	192.000	0.000
A_06_P6381	YOR019W	275.000	0.000
A_06_P6382	YOR020C	29.000	0.000
A_06_P6383	YOR021C	29.000	0.000
A_06_P6384	YOR022C	160.000	0.000
A_06_P6385	YOR023C	16.000	0.000
A_06_P6386	YOR024W	6.000	0.000
A_06_P6387	YOR025W	20.000	0.000
A_06_P6388	YOR026W	53.000	0.000
A_06_P6389	YOR027W	18.000	0.000
A_06_P6390	YOR028C	66.000	0.000
A_06_P6391	YOR029W	4.000	0.000
A_06_P6392	YOR030W	253.000	0.000
A_06_P6393	YOR031W	6.000	0.000
A_06_P6394	YOR032C	40.000	0.000
A_06_P6395	YOR033C	12.000	0.000
A_06_P6396	YOR034C	48.000	0.000
A_06_P6397	YOR035C	193.000	0.000
A_06_P6398	YOR036W	55.000	0.000
A_06_P6399	YOR037W	29.000	0.000

A_06_P6400	YOR038C	27.000	0.000
A_06_P6401	YOR039W	52.000	0.000
A_06_P6402	YOR040W	185.000	0.000
A_06_P6403	YOR041C	67.000	0.000
A_06_P6404	YOR042W	38.000	0.000
A_06_P6405	YOR043W	84.000	0.000
A_06_P6406	YOR044W	79.000	0.000
A_06_P6407	YOR045W	128.000	0.000
A_06_P6408	YOR046C	53.000	0.000
A_06_P6409	YOR047C	163.000	0.000
A_06_P6410	YOR048C	291.000	0.000
A_06_P6411	YOR049C	84.000	0.000
A_06_P6412	YOR050C	11.000	0.000
A_06_P6413	YOR051C	106.000	0.000
A_06_P6414	YOR052C	26.000	0.000
A_06_P6415	YOR053W	81.000	0.000
A_06_P6416	YOR054C	21.000	0.000
A_06_P6417	YOR055W	19.000	0.000
A_06_P6418	YOR056C	71.000	0.000
A_06_P6419	YOR057W	52.000	0.000
A_06_P6420	YOR058C	115.000	0.000
A_06_P6421	YOR059C	69.000	0.000
A_06_P6422	YOR060C	2.000	0.000
A_06_P6423	YOR061W	5.000	0.000
A_06_P6424	YOR062C	14.000	0.000
A_06_P6425	YOR063W	140.000	0.000
A_06_P6426	YOR064C	16.000	0.000
A_06_P6427	YOR065W	93.000	0.000
A_06_P6428	YOR066W	19.000	0.000
A_06_P6429	YOR067C	13.000	0.000
A_06_P6430	YOR068C	236.000	0.000
A_06_P6431	YOR069W	35.000	0.000
A_06_P6432	YOR070C	31.000	0.000
A_06_P6433	YOR071C	571.000	0.000
A_06_P6434	YOR072W	215.000	0.000
A_06_P6435	YOR073W	79.000	0.000
A_06_P6436	YOR074C	18.000	0.000
A_06_P6437	YOR075W	7.000	0.000
A_06_P6438	YOR076C	344.000	0.000
A_06_P6439	YOR077W	584.000	0.000
A_06_P6440	YOR078W	25.000	0.000
A_06_P6441	YOR079C	16.000	0.000
A_06_P6442	YOR080W	21.000	0.000
A_06_P6443	YOR081C	43.000	0.000
A_06_P6444	YOR082C	24.000	0.000
A_06_P6445	YOR083W	213.000	0.000
A_06_P6446	YOR084W	342.000	0.000
A_06_P6447	YOR085W	15.000	0.000
A_06_P6448	YOR086C	281.000	0.000
A_06_P6449	YOR087W	2.000	0.000
A_06_P6451	YOR089C	106.000	0.000
A_06_P6452	YOR090C	18.000	0.000
A_06_P6453	YOR091W	110.000	0.000
A_06_P6454	YOR092W	78.000	0.000
A_06_P6455	YOR093C	697.000	0.000

A_06_P6456	YOR094W	104.000	0.000
A_06_P6457	YOR095C	234.000	0.000
A_06_P6458	YOR096W	26.000	0.000
A_06_P6459	YOR097C	11.000	0.000
A_06_P6460	YOR098C	70.000	0.000
A_06_P6461	YOR099W	16.000	0.000
A_06_P6462	YOR100C	734.000	0.000
A_06_P6463	YOR101W	141.000	0.000
A_06_P6464	YOR102W	67.000	0.000
A_06_P6465	YOR103C	6.000	0.000
A_06_P6466	YOR104W	125.200	2.387
A_06_P6467	YOR104W	3.800	2.387
A_06_P6467	YOR105W	10.000	0.000
A_06_P6468	YOR106W	22.000	0.000
A_06_P6469	YOR107W	8.000	0.000
A_06_P6470	YOR108W	128.000	0.000
A_06_P6471	YOR109W	32.000	0.000
A_06_P6472	YOR110W	207.000	0.000
A_06_P6473	YOR111W	13.000	0.000
A_06_P6474	YOR112W	30.000	0.000
A_06_P6475	YOR113W	171.000	0.000
A_06_P6476	YOR114W	80.000	0.000
A_06_P6477	YOR115C	5.000	0.000
A_06_P6478	YOR116C	31.000	0.000
A_06_P6479	YOR117W	39.000	0.000
A_06_P6480	YOR118W	42.000	0.000
A_06_P6481	YOR119C	21.000	0.000
A_06_P6482	YOR120W	70.000	0.000
A_06_P6483	YOR121C	8.000	0.000
A_06_P6484	YOR122C	22.000	0.000
A_06_P6485	YOR123C	65.000	0.000
A_06_P6486	YOR124C	56.000	0.000
A_06_P6487	YOR125C	21.000	0.000
A_06_P6488	YOR126C	83.000	0.000
A_06_P6489	YOR127W	46.000	0.000
A_06_P6490	YOR128C	85.000	0.000
A_06_P6491	YOR129C	69.000	0.000
A_06_P6492	YOR130C	243.000	0.000
A_06_P6493	YOR131C	45.000	0.000
A_06_P6494	YOR132W	5.000	0.000
A_06_P6495	YDR385W	23.200	3.033
A_06_P6495	YOR133W	9.000	0.000
A_06_P6496	YOR134W	39.000	0.000
A_06_P6497	YOR135C	15.000	0.000
A_06_P6498	YOR136W	29.000	0.000
A_06_P6499	YOR137C	148.000	0.000
A_06_P6500	YOR138C	146.000	0.000
A_06_P6501	YOR139C	65.000	0.000
A_06_P6502	YOR140W	66.000	0.000
A_06_P6503	YOR141C	65.000	0.000
A_06_P6504	YOR142W	168.000	0.000
A_06_P6505	YOR143C	73.000	0.000
A_06_P6506	YOR144C	25.000	0.000
A_06_P6507	YOR145C	12.000	0.000
A_06_P6508	YOR146W	57.000	0.000

A_06_P6509	YOR147W	7.000	0.000
A_06_P6510	YOR148C	160.000	0.000
A_06_P6511	YOR149C	54.000	0.000
A_06_P6512	YOR150W	41.000	0.000
A_06_P6513	YOR151C	17.000	0.000
A_06_P6514	YOR152C	6.000	0.000
A_06_P6515	YOR153W	4.000	0.000
A_06_P6516	YOR154W	28.000	0.000
A_06_P6517	YOR155C	130.000	0.000
A_06_P6518	YOR156C	6.000	0.000
A_06_P6519	YOR157C	40.000	0.000
A_06_P6520	YOR158W	27.000	0.000
A_06_P6521	YOR159C	21.000	0.000
A_06_P6522	YOR160W	129.000	0.000
A_06_P6523	YMR315W-A	0.200	0.447
A_06_P6523	YOR161C	11.000	0.000
A_06_P6524	YOR162C	53.000	0.000
A_06_P6525	YOR163W	113.000	0.000
A_06_P6526	YOR164C	219.000	0.000
A_06_P6527	YOR165W	187.000	0.000
A_06_P6528	YOR166C	33.000	0.000
A_06_P6529	YOR167C	44.000	0.000
A_06_P6530	YOR168W	70.000	0.000
A_06_P6531	YOR169C	192.000	0.000
A_06_P6532	YOR170W	3.000	0.000
A_06_P6533	YOR171C	19.000	0.000
A_06_P6534	YOR172W	386.000	0.000
A_06_P6535	YOR173W	91.000	0.000
A_06_P6536	YOR174W	31.000	0.000
A_06_P6537	YOR175C	185.000	0.000
A_06_P6538	YOR176W	59.000	0.000
A_06_P6539	YOR177C	115.000	0.000
A_06_P6540	YOR178C	11.000	0.000
A_06_P6541	YOR179C	16.000	0.000
A_06_P6542	YOR180C	164.000	0.000
A_06_P6543	YOR181W	176.000	0.000
A_06_P6544	YOR182C	41.000	0.000
A_06_P6545	YOR183W	27.000	0.000
A_06_P6546	YOR184W	7.000	0.000
A_06_P6547	YOR185C	56.000	0.000
A_06_P6548	YOR186W	47.000	0.000
A_06_P6549	YOR187W	2.000	0.000
A_06_P6550	YOR188W	240.000	0.000
A_06_P6551	YOR189W	49.000	0.000
A_06_P6552	YOR190W	6.000	0.000
A_06_P6553	YOR191W	178.000	0.000
A_06_P6554	YOR192C	45.000	0.000
A_06_P6555	YOR193W	297.000	0.000
A_06_P6556	YOR194C	30.000	0.000
A_06_P6557	YOR195W	22.000	0.000
A_06_P6558	YOR196C	10.000	0.000
A_06_P6559	YOR197W	31.000	0.000
A_06_P6560	YOR198C	18.000	0.000
A_06_P6561	YOR199W	43.000	0.000
A_06_P6562	YOR200W	137.000	0.000

A_06_P6563	YOR201C	17.000	0.000
A_06_P6564	YOR202W	13.000	0.000
A_06_P6565	YOR203W	31.000	0.000
A_06_P6566	YOR204W	28.000	0.000
A_06_P6567	YOR205C	235.000	0.000
A_06_P6568	YOR206W	26.000	0.000
A_06_P6569	YOR207C	27.000	0.000
A_06_P6570	YOR208W	30.000	0.000
A_06_P6571	YOR209C	67.000	0.000
A_06_P6572	YOR210W	17.000	0.000
A_06_P6573	YOR211C	4.000	0.000
A_06_P6574	YOR212W	51.000	0.000
A_06_P6575	YOR213C	47.000	0.000
A_06_P6576	YOR214C	161.000	0.000
A_06_P6577	YOR215C	29.000	0.000
A_06_P6578	YOR216C	24.000	0.000
A_06_P6579	YOR217W	10.000	0.000
A_06_P6580	YOR218C	138.000	0.000
A_06_P6581	YOR219C	134.000	0.000
A_06_P6582	YOR220W	90.000	0.000
A_06_P6583	YOR221C	146.000	0.000
A_06_P6584	YOR222W	14.000	0.000
A_06_P6585	YOR223W	166.000	0.000
A_06_P6586	YOR224C	100.000	0.000
A_06_P6587	YOR225W	28.000	0.000
A_06_P6588	YOR226C	18.000	0.000
A_06_P6589	YOR227W	44.000	0.000
A_06_P6590	YOR228C	18.000	0.000
A_06_P6591	YOR229W	14.000	0.000
A_06_P6592	YOR230W	65.000	0.000
A_06_P6593	YOR231W	54.000	0.000
A_06_P6594	YOR232W	76.000	0.000
A_06_P6595	YOR233W	47.000	0.000
A_06_P6596	YOR234C	65.000	0.000
A_06_P6597	YOR235W	59.000	0.000
A_06_P6598	YOR236W	55.000	0.000
A_06_P6599	YOR237W	17.000	0.000
A_06_P6600	YOR238W	24.000	0.000
A_06_P6601	YOR239W	24.000	0.000
A_06_P6602	YOR241W	46.000	0.000
A_06_P6603	YOR242C	129.000	0.000
A_06_P6604	YOR243C	41.000	0.000
A_06_P6605	YOR244W	20.000	0.000
A_06_P6606	YOR245C	254.000	0.000
A_06_P6607	YOR246C	43.000	0.000
A_06_P6608	YOR247W	32.800	0.447
A_06_P6609	YOR247W	0.200	0.447
A_06_P6609	YOR248W	48.000	0.000
A_06_P6610	YOR249C	239.000	0.000
A_06_P6611	YOR250C	96.000	0.000
A_06_P6612	YOR251C	531.000	0.000
A_06_P6613	YOR252W	31.000	0.000
A_06_P6614	YOR253W	31.000	0.000
A_06_P6615	YOR254C	142.000	0.000
A_06_P6616	YOR255W	44.000	0.000

A_06_P6617	YOR256C	37.000	0.000
A_06_P6618	YOR257W	9.000	0.000
A_06_P6619	YOR258W	144.000	0.000
A_06_P6620	YOR259C	210.000	0.000
A_06_P6621	YOR260W	57.000	0.000
A_06_P6622	YOR261C	27.000	0.000
A_06_P6623	YOR262W	275.000	0.000
A_06_P6624	YOR263C	16.000	0.000
A_06_P6625	YOR264W	109.000	0.000
A_06_P6626	YOR265W	46.000	0.000
A_06_P6627	YOR266W	10.000	0.000
A_06_P6628	YOR267C	24.000	0.000
A_06_P6629	YOR268C	126.000	0.000
A_06_P6630	YOR269W	83.000	0.000
A_06_P6631	YOR270C	197.000	0.000
A_06_P6632	YOR271C	40.000	0.000
A_06_P6633	YOR272W	324.000	0.000
A_06_P6634	YOR273C	22.000	0.000
A_06_P6635	YOR274W	88.000	0.000
A_06_P6636	YOR275C	63.000	0.000
A_06_P6637	YOR276W	72.000	0.000
A_06_P6638	YOR277C	11.000	0.000
A_06_P6639	YOR278W	58.000	0.000
A_06_P6640	YOR279C	34.000	0.000
A_06_P6641	YOR280C	48.000	0.000
A_06_P6642	YOR281C	198.000	0.000
A_06_P6643	YOR282W	87.000	0.000
A_06_P6644	YOR283W	88.000	0.000
A_06_P6645	YOR284W	2.000	0.000
A_06_P6646	YOR285W	6.000	0.000
A_06_P6647	YOR286W	408.000	0.000
A_06_P6648	YOR287C	76.000	0.000
A_06_P6649	YOR288C	12.000	0.000
A_06_P6650	YOR289W	105.000	0.000
A_06_P6651	YOR290C	18.000	0.000
A_06_P6652	YOR291W	196.000	0.000
A_06_P6653	YOR292C	6.000	0.000
A_06_P6654	YOR293W	70.000	0.000
A_06_P6655	YOR294W	128.000	0.000
A_06_P6656	YOR295W	30.000	0.000
A_06_P6657	YOR296W	77.000	0.000
A_06_P6658	YOR297C	52.000	0.000
A_06_P6659	YOR298C-A	33.000	0.000
A_06_P6660	YOR298W	27.000	0.000
A_06_P6661	YOR299W	18.000	0.000
A_06_P6662	YOR300W	23.000	0.000
A_06_P6663	YOR301W	120.000	0.000
A_06_P6664	YOR302W	51.000	0.000
A_06_P6665	YOR303W	15.000	0.000
A_06_P6666	YOR304C-A	79.000	0.000
A_06_P6667	YOR304W	13.000	0.000
A_06_P6668	YOR305W	25.000	0.000
A_06_P6669	YOR306C	74.000	0.000
A_06_P6670	YOR307C	26.000	0.000
A_06_P6671	YOR308C	22.000	0.000

A_06_P6672	YOR309C	69.000	0.000
A_06_P6673	YOR310C	55.000	0.000
A_06_P6674	YOR311C	42.000	0.000
A_06_P6675	YOR312C	24.000	0.000
A_06_P6676	YOR313C	232.000	0.000
A_06_P6677	YOR314W	383.000	0.000
A_06_P6678	YOR314W-A	388.000	0.000
A_06_P6679	YOR315W	35.000	0.000
A_06_P6680	YOR316C	63.000	0.000
A_06_P6681	YOR317W	132.000	0.000
A_06_P6682	YOR318C	147.000	0.000
A_06_P6683	YOR319W	57.000	0.000
A_06_P6684	YOR320C	48.000	0.000
A_06_P6685	YOR321W	51.000	0.000
A_06_P6686	YOR322C	85.000	0.000
A_06_P6687	YOR323C	92.000	0.000
A_06_P6688	YOR324C	48.000	0.000
A_06_P6689	YOR325W	21.000	0.000
A_06_P6690	YOR326W	120.000	0.000
A_06_P6691	YOR327C	28.000	0.000
A_06_P6692	YOR328W	171.000	0.000
A_06_P6693	YOR329C	66.000	0.000
A_06_P6694	YOR330C	7.000	0.000
A_06_P6695	YOR331C	12.000	0.000
A_06_P6696	YOR332W	27.000	0.000
A_06_P6697	YOR333C	38.000	0.000
A_06_P6698	YOR334W	43.000	0.000
A_06_P6699	YOR335C	11.000	0.000
A_06_P6700	YOR336W	27.000	0.000
A_06_P6701	YOR337W	55.000	0.000
A_06_P6702	YOR338W	7.000	0.000
A_06_P6703	YOR339C	29.000	0.000
A_06_P6704	YOR340C	31.000	0.000
A_06_P6705	YOR341W	4.000	0.000
A_06_P6706	YOR342C	3.000	0.000
A_06_P6707	YOR343C	32.000	0.000
A_06_P6708	YOR344C	102.000	0.000
A_06_P6709	YOR345C	108.000	0.000
A_06_P6710	YOR346W	205.000	0.000
A_06_P6711	YOR347C	32.000	0.000
A_06_P6712	YOR348C	15.000	0.000
A_06_P6713	YOR349W	222.000	0.000
A_06_P6714	YOR350C	225.000	0.000
A_06_P6715	YOR351C	27.000	0.000
A_06_P6716	YOR352W	76.000	0.000
A_06_P6717	YOR353C	22.000	0.000
A_06_P6718	YOR354C	183.000	0.000
A_06_P6719	YOR355W	26.000	0.000
A_06_P6720	YOR356W	76.000	0.000
A_06_P6721	YOR357C	82.000	0.000
A_06_P6722	YOR358W	12.000	0.000
A_06_P6723	YOR359W	30.000	0.000
A_06_P6724	YOR360C	39.000	0.000
A_06_P6725	YOR361C	30.000	0.000
A_06_P6726	YOR362C	11.000	0.000

A_06_P6727	YOR363C	9.000	0.000
A_06_P6728	YOR364W	16.000	0.000
A_06_P6729	YOR365C	151.000	0.000
A_06_P6730	YOR366W	35.000	0.000
A_06_P6731	YOR367W	14.000	0.000
A_06_P6732	YOR368W	10.000	0.000
A_06_P6733	YOR369C	25.000	0.000
A_06_P6734	YOR370C	102.000	0.000
A_06_P6735	YOR371C	46.000	0.000
A_06_P6736	YOR372C	34.000	0.000
A_06_P6737	YOR373W	111.000	0.000
A_06_P6738	YOR374W	20.000	0.000
A_06_P6739	YOR375C	108.000	0.000
A_06_P6740	YOR376W	118.000	0.000
A_06_P6741	YOR377W	16.000	0.000
A_06_P6742	YOR378W	29.000	0.000
A_06_P6743	YOR379C	34.000	0.000
A_06_P6744	YOR380W	17.000	0.000
A_06_P6745	YOR381W	105.000	0.000
A_06_P6746	YOR382W	372.000	0.000
A_06_P6747	YOR383C	15.000	0.000
A_06_P6748	YOR384W	202.000	0.000
A_06_P6749	YOR385W	153.000	0.000
A_06_P6750	YOR386W	10.000	0.000
A_06_P6751	YOR387C	52.000	0.000
A_06_P6752	YOR388C	93.400	0.548
A_06_P6753	YOR389W	0.800	0.837
A_06_P6754	YOR390W	13.000	0.000
A_06_P6755	YMR322C	0.200	0.447
A_06_P6755	YOR391C	10.600	1.342
A_06_P6755	YPL280W	3.600	2.074
A_06_P6756	YOR392W	41.000	0.000
A_06_P6757	YMR323W	34.400	3.782
A_06_P6757	YOR393W	84.400	2.408
A_06_P6757	YPL281C	18.400	4.037
A_06_P6758	YOR394W	13.600	2.302
A_06_P6758	YPL282C	1.800	0.837
A_06_P6759	YEL077C	0.200	0.447
A_06_P6760	YPL001W	10.000	0.000
A_06_P6761	YPL002C	59.000	0.000
A_06_P6762	YPL003W	110.000	0.000
A_06_P6763	YPL004C	86.000	0.000
A_06_P6764	YPL005W	56.000	0.000
A_06_P6765	YPL006W	36.000	0.000
A_06_P6766	YPL007C	32.000	0.000
A_06_P6767	YPL008W	71.000	0.000
A_06_P6768	YPL009C	24.000	0.000
A_06_P6769	YPL010W	10.000	0.000
A_06_P6770	YPL011C	14.000	0.000
A_06_P6771	YPL012W	44.000	0.000
A_06_P6772	YPL013C	2.000	0.000
A_06_P6773	YPL014W	263.000	0.000
A_06_P6774	YPL015C	10.000	0.000
A_06_P6775	YPL016W	204.000	0.000
A_06_P6776	YPL017C	60.000	0.000

A_06_P6777	YPL018W	12.000	0.000
A_06_P6778	YPL019C	7.000	0.000
A_06_P6779	YPL020C	66.000	0.000
A_06_P6780	YPL021W	403.000	0.000
A_06_P6781	YPL022W	60.000	0.000
A_06_P6782	YPL023C	96.000	0.000
A_06_P6783	YPL024W	7.000	0.000
A_06_P6784	YPL025C	144.000	0.000
A_06_P6785	YPL026C	231.000	0.000
A_06_P6786	YPL027W	73.000	0.000
A_06_P6787	YPL028W	50.000	0.000
A_06_P6788	YPL029W	29.000	0.000
A_06_P6789	YPL030W	19.000	0.000
A_06_P6790	YPL031C	50.000	0.000
A_06_P6791	YPL032C	46.000	0.000
A_06_P6792	YPL033C	56.000	0.000
A_06_P6793	YPL034W	17.000	0.000
A_06_P6794	YPL035C	17.000	0.000
A_06_P6795	YPL036W	204.000	0.000
A_06_P6796	YPL037C	97.000	0.000
A_06_P6797	YPL038W	11.000	0.000
A_06_P6798	YPL039W	97.000	0.000
A_06_P6799	YPL040C	59.000	0.000
A_06_P6800	YPL041C	5.000	0.000
A_06_P6801	YPL042C	43.000	0.000
A_06_P6802	YPL043W	34.000	0.000
A_06_P6803	YPL044C	185.000	0.000
A_06_P6804	YPL045W	2.000	0.000
A_06_P6805	YPL046C	11.000	0.000
A_06_P6806	YPL047W	27.000	0.000
A_06_P6807	YPL048W	65.000	0.000
A_06_P6808	YPL049C	18.000	0.000
A_06_P6809	YPL050C	14.000	0.000
A_06_P6810	YPL051W	35.000	0.000
A_06_P6811	YPL052W	196.000	0.000
A_06_P6812	YPL053C	355.000	0.000
A_06_P6813	YPL054W	159.000	0.000
A_06_P6814	YPL055C	184.000	0.000
A_06_P6815	YPL056C	53.000	0.000
A_06_P6816	YPL057C	20.000	0.000
A_06_P6817	YPL058C	19.000	0.000
A_06_P6818	YPL059W	39.000	0.000
A_06_P6819	YPL060W	82.000	0.000
A_06_P6820	YPL061W	191.000	0.000
A_06_P6821	YPL062W	24.000	0.000
A_06_P6822	YPL063W	68.000	0.000
A_06_P6823	YPL064C	59.000	0.000
A_06_P6824	YPL065W	43.000	0.000
A_06_P6825	YPL066W	152.000	0.000
A_06_P6826	YPL067C	53.000	0.000
A_06_P6827	YPL068C	148.000	0.000
A_06_P6828	YPL069C	180.000	0.000
A_06_P6829	YPL070W	32.000	0.000
A_06_P6830	YPL071C	5.000	0.000
A_06_P6831	YPL072W	54.000	0.000

A_06_P6832	YPL073C	15.000	0.000
A_06_P6833	YPL074W	103.000	0.000
A_06_P6834	YPL075W	90.000	0.000
A_06_P6835	YPL076W	131.000	0.000
A_06_P6836	YPL077C	63.000	0.000
A_06_P6837	YPL078C	35.000	0.000
A_06_P6838	YPL079W	60.000	0.000
A_06_P6839	YPL080C	107.000	0.000
A_06_P6840	YPL081W	18.000	0.000
A_06_P6841	YPL082C	29.000	0.000
A_06_P6842	YPL083C	34.000	0.000
A_06_P6843	YPL084W	3.000	0.000
A_06_P6844	YPL085W	58.000	0.000
A_06_P6845	YPL086C	11.000	0.000
A_06_P6846	YPL087W	166.000	0.000
A_06_P6847	YPL088W	157.000	0.000
A_06_P6848	YPL089C	18.000	0.000
A_06_P6849	YPL090C	173.400	0.894
A_06_P6850	YPL091W	5.000	0.000
A_06_P6851	YPL092W	67.000	0.000
A_06_P6852	YPL093W	12.000	0.000
A_06_P6853	YPL094C	74.000	0.000
A_06_P6854	YPL095C	50.000	0.000
A_06_P6855	YPL096W	385.000	0.000
A_06_P6856	YPL097W	45.000	0.000
A_06_P6857	YPL098C	53.000	0.000
A_06_P6858	YPL099C	334.000	0.000
A_06_P6859	YPL100W	66.000	0.000
A_06_P6860	YPL101W	4.000	0.000
A_06_P6861	YPL102C	152.000	0.000
A_06_P6862	YPL103C	21.000	0.000
A_06_P6863	YPL104W	69.000	0.000
A_06_P6864	YPL105C	4.000	0.000
A_06_P6865	YPL106C	117.000	0.000
A_06_P6866	YPL107W	51.000	0.000
A_06_P6867	YPL108W	107.000	0.000
A_06_P6868	YPL109C	66.000	0.000
A_06_P6869	YPL110C	11.000	0.000
A_06_P6870	YPL111W	38.000	0.000
A_06_P6871	YPL112C	28.000	0.000
A_06_P6872	YPL113C	35.000	0.000
A_06_P6873	YPL114W	407.000	0.000
A_06_P6874	YPL115C	7.000	0.000
A_06_P6875	YPL116W	19.000	0.000
A_06_P6876	YPL117C	55.000	0.000
A_06_P6877	YPL118W	10.000	0.000
A_06_P6878	YPL119C	3.000	0.000
A_06_P6879	YPL120W	17.000	0.000
A_06_P6880	YPL121C	64.000	0.000
A_06_P6881	YPL122C	30.000	0.000
A_06_P6882	YPL123C	23.000	0.000
A_06_P6883	YPL124W	57.000	0.000
A_06_P6884	YPL125W	14.000	0.000
A_06_P6885	YPL126W	13.000	0.000
A_06_P6886	YPL127C	12.000	0.000

A_06_P6887	YPL128C	56.000	0.000
A_06_P6888	YPL129W	165.000	0.000
A_06_P6889	YPL130W	54.000	0.000
A_06_P6890	YPL131W	253.000	0.000
A_06_P6891	YPL132W	56.000	0.000
A_06_P6892	YPL133C	39.000	0.000
A_06_P6893	YPL134C	89.000	0.000
A_06_P6894	YPL135W	133.000	0.000
A_06_P6895	YPL136W	23.000	0.000
A_06_P6896	YPL137C	13.000	0.000
A_06_P6897	YPL138C	34.000	0.000
A_06_P6898	YPL139C	41.000	0.000
A_06_P6899	YPL140C	60.000	0.000
A_06_P6900	YPL141C	8.000	0.000
A_06_P6901	YPL142C	28.000	0.000
A_06_P6902	YPL143W	39.000	0.000
A_06_P6903	YPL144W	44.000	0.000
A_06_P6904	YPL145C	40.000	0.000
A_06_P6905	YPL146C	94.000	0.000
A_06_P6906	YPL147W	49.000	0.000
A_06_P6907	YPL148C	183.000	0.000
A_06_P6908	YPL149W	493.000	0.000
A_06_P6909	YPL150W	32.000	0.000
A_06_P6910	YPL151C	21.000	0.000
A_06_P6911	YPL152W	30.000	0.000
A_06_P6912	YPL153C	274.000	0.000
A_06_P6913	YPL154C	16.000	0.000
A_06_P6914	YPL155C	12.000	0.000
A_06_P6915	YPL156C	101.000	0.000
A_06_P6916	YPL157W	197.000	0.000
A_06_P6917	YPL158C	26.000	0.000
A_06_P6918	YPL159C	56.000	0.000
A_06_P6919	YPL160W	1.000	0.000
A_06_P6920	YPL161C	43.000	0.000
A_06_P6921	YPL162C	16.000	0.000
A_06_P6922	YPL163C	25.000	0.000
A_06_P6923	YPL164C	19.000	0.000
A_06_P6924	YPL165C	17.000	0.000
A_06_P6925	YPL166W	12.000	0.000
A_06_P6926	YPL167C	179.000	0.000
A_06_P6927	YPL168W	265.000	0.000
A_06_P6928	YPL169C	38.000	0.000
A_06_P6929	YPL170W	16.000	0.000
A_06_P6930	YPL171C	160.000	0.000
A_06_P6931	YPL172C	28.000	0.000
A_06_P6932	YPL173W	21.000	0.000
A_06_P6933	YPL174C	50.000	0.000
A_06_P6934	YPL175W	306.000	0.000
A_06_P6935	YPL176C	10.000	0.000
A_06_P6936	YPL177C	24.000	0.000
A_06_P6937	YPL178W	25.000	0.000
A_06_P6938	YPL179W	11.000	0.000
A_06_P6939	YPL180W	91.000	0.000
A_06_P6940	YPL181W	67.000	0.000
A_06_P6941	YPL182C	1.000	0.000

A_06_P6942	YPL183C	50.000	0.000
A_06_P6943	YPL183W-A	129.000	0.000
A_06_P6944	YPL184C	19.000	0.000
A_06_P6945	YPL185W	13.000	0.000
A_06_P6946	YPL186C	23.000	0.000
A_06_P6947	YPL187W	57.000	0.000
A_06_P6948	YPL188W	210.000	0.000
A_06_P6949	YPL189W	35.000	0.000
A_06_P6950	YPL190C	1.000	0.000
A_06_P6951	YPL191C	50.000	0.000
A_06_P6952	YPL192C	14.000	0.000
A_06_P6953	YPL193W	89.000	0.000
A_06_P6954	YPL194W	49.000	0.000
A_06_P6955	YPL195W	21.000	0.000
A_06_P6956	YPL196W	23.000	0.000
A_06_P6957	YPL197C	27.000	0.000
A_06_P6958	YPL198W	9.000	0.000
A_06_P6959	YPL199C	49.000	0.000
A_06_P6960	YPL200W	56.000	0.000
A_06_P6961	YPL201C	16.000	0.000
A_06_P6962	YPL202C	21.000	0.000
A_06_P6963	YPL203W	207.000	0.000
A_06_P6964	YPL204W	159.000	0.000
A_06_P6965	YPL205C	51.000	0.000
A_06_P6966	YPL206C	230.000	0.000
A_06_P6967	YPL207W	21.000	0.000
A_06_P6968	YPL208W	78.000	0.000
A_06_P6969	YPL209C	27.000	0.000
A_06_P6970	YPL210C	91.000	0.000
A_06_P6971	YPL211W	37.000	0.000
A_06_P6972	YPL212C	95.000	0.000
A_06_P6973	YPL213W	78.000	0.000
A_06_P6974	YPL214C	32.000	0.000
A_06_P6975	YPL215W	16.000	0.000
A_06_P6976	YPL216W	11.000	0.000
A_06_P6977	YPL217C	7.000	0.000
A_06_P6978	YPL218W	21.000	0.000
A_06_P6979	YPL219W	101.000	0.000
A_06_P6980	YPL220W	65.800	3.421
A_06_P6981	YPL221W	77.000	0.000
A_06_P6982	YPL222W	26.000	0.000
A_06_P6983	YPL223C	8.000	0.000
A_06_P6984	YPL224C	424.000	0.000
A_06_P6985	YPL225W	18.000	0.000
A_06_P6986	YPL226W	112.000	0.000
A_06_P6987	YPL227C	51.000	0.000
A_06_P6988	YPL228W	33.000	0.000
A_06_P6989	YPL229W	489.000	0.000
A_06_P6990	YPL230W	13.000	0.000
A_06_P6991	YPL231W	44.000	0.000
A_06_P6992	YPL232W	35.000	0.000
A_06_P6993	YPL233W	10.000	0.000
A_06_P6994	YPL234C	38.000	0.000
A_06_P6995	YPL235W	33.000	0.000
A_06_P6996	YPL236C	19.000	0.000

A_06_P6997	YPL237W	23.000	0.000
A_06_P6998	YPL238C	153.000	0.000
A_06_P6999	YPL239W	13.000	0.000
A_06_P7000	YPL240C	37.000	0.000
A_06_P7001	YPL241C	21.000	0.000
A_06_P7002	YPL242C	108.000	0.000
A_06_P7003	YPL243W	48.000	0.000
A_06_P7004	YPL244C	8.000	0.000
A_06_P7005	YPL245W	13.000	0.000
A_06_P7006	YPL246C	121.000	0.000
A_06_P7007	YPL247C	97.000	0.000
A_06_P7008	YPL248C	87.000	0.000
A_06_P7009	YPL249C	7.000	0.000
A_06_P7010	YPL249C-A	53.000	0.000
A_06_P7011	YPL250C	50.000	0.000
A_06_P7012	YPL251W	13.000	0.000
A_06_P7013	YPL252C	14.000	0.000
A_06_P7014	YPL253C	45.000	0.000
A_06_P7015	YPL254W	25.000	0.000
A_06_P7016	YPL255W	449.000	0.000
A_06_P7017	YPL256C	484.000	0.000
A_06_P7018	YPL257W	372.000	0.000
A_06_P7019	YPL258C	46.000	0.000
A_06_P7020	YPL259C	559.000	0.000
A_06_P7021	YPL260W	85.000	0.000
A_06_P7022	YPL261C	105.000	0.000
A_06_P7023	YPL262W	22.000	0.000
A_06_P7024	YPL263C	142.000	0.000
A_06_P7025	YPL264C	4.000	0.000
A_06_P7026	YPL265W	20.000	0.000
A_06_P7027	YPL266W	1000.000	0.000
A_06_P7028	YPL267W	235.000	0.000
A_06_P7029	YPL268W	30.000	0.000
A_06_P7030	YPL269W	87.000	0.000
A_06_P7031	YPL270W	142.000	0.000
A_06_P7032	YPL271W	105.000	0.000
A_06_P7033	YPL272C	39.000	0.000
A_06_P7034	YPL273W	44.600	3.782
A_06_P7035	YPL274W	9.000	0.000
A_06_P7036	YOR388C	0.200	0.447
A_06_P7036	YPL275W	11.000	0.000
A_06_P7037	YOR388C	1.400	0.548
A_06_P7037	YPL276W	18.000	0.000
A_06_P7038	YPL277C	446.000	0.000
A_06_P7039	YOR389W	6.200	0.837
A_06_P7039	YPL278C	23.000	0.000
A_06_P7040	YPL279C	4.000	0.000
A_06_P7041	YMR322C	0.800	0.837
A_06_P7041	YOR391C	0.200	0.447
A_06_P7041	YPL280W	5.800	2.683
A_06_P7042	YMR323W	36.000	2.236
A_06_P7042	YOR393W	80.600	2.408
A_06_P7042	YPL281C	18.600	4.037
A_06_P7043	YIR041W	0.200	0.447
A_06_P7043	YOR394W	14.400	2.302

A_06_P7043	YPL282C	1.200	0.837
A_06_P7044	YPR204W	0.200	0.447
A_06_P7045	YPR001W	356.000	0.000
A_06_P7046	YPR002C-A	2.200	1.643
A_06_P7047	YPR002W	38.000	0.000
A_06_P7048	YPR003C	84.000	0.000
A_06_P7049	YPR004C	44.000	0.000
A_06_P7050	YPR005C	19.000	0.000
A_06_P7051	YPR006C	16.000	0.000
A_06_P7052	YPR007C	33.000	0.000
A_06_P7053	YPR008W	124.000	0.000
A_06_P7054	YPR009W	56.000	0.000
A_06_P7055	YPR010C	25.000	0.000
A_06_P7056	YPR011C	9.000	0.000
A_06_P7057	YPR012W	32.000	0.000
A_06_P7058	YPR013C	21.000	0.000
A_06_P7059	YPR014C	30.000	0.000
A_06_P7060	YPR015C	5.000	0.000
A_06_P7061	YPR016C	492.000	0.000
A_06_P7062	YPR016W-A	132.000	0.000
A_06_P7063	YPR017C	109.000	0.000
A_06_P7064	YPR018W	44.000	0.000
A_06_P7065	YPR019W	26.000	0.000
A_06_P7066	YPR020W	9.000	0.000
A_06_P7067	YPR021C	36.000	0.000
A_06_P7068	YPR022C	6.000	0.000
A_06_P7069	YPR023C	98.000	0.000
A_06_P7070	YPR024W	9.000	0.000
A_06_P7071	YPR025C	144.000	0.000
A_06_P7072	YPR026W	37.000	0.000
A_06_P7073	YPR027C	237.000	0.000
A_06_P7074	YPR028W	57.000	0.000
A_06_P7075	YPR029C	8.000	0.000
A_06_P7076	YPR030W	162.000	0.000
A_06_P7077	YPR031W	66.000	0.000
A_06_P7078	YPR032W	37.000	0.000
A_06_P7079	YPR033C	38.000	0.000
A_06_P7080	YPR034W	54.000	0.000
A_06_P7081	YPR035W	44.000	0.000
A_06_P7082	YPR036W	117.000	0.000
A_06_P7083	YPR037C	21.000	0.000
A_06_P7084	YPR038W	79.000	0.000
A_06_P7085	YPR039W	30.000	0.000
A_06_P7086	YPR040W	28.000	0.000
A_06_P7087	YPR041W	38.000	0.000
A_06_P7088	YPR042C	64.000	0.000
A_06_P7089	YPR043W	125.000	0.000
A_06_P7090	YPR044C	122.000	0.000
A_06_P7091	YPR045C	31.000	0.000
A_06_P7092	YPR046W	171.000	0.000
A_06_P7093	YPR047W	46.000	0.000
A_06_P7094	YPR048W	76.000	0.000
A_06_P7095	YPR049C	54.000	0.000
A_06_P7096	YPR050C	79.000	0.000
A_06_P7097	YPR051W	56.000	0.000

A_06_P7098	YPR052C	22.000	0.000
A_06_P7099	YPR053C	241.000	0.000
A_06_P7100	YPR054W	3.000	0.000
A_06_P7101	YPR055W	38.000	0.000
A_06_P7102	YPR056W	30.000	0.000
A_06_P7103	YPR057W	32.000	0.000
A_06_P7104	YPR058W	30.000	0.000
A_06_P7105	YPR059C	393.000	0.000
A_06_P7106	YPR060C	4.000	0.000
A_06_P7107	YPR061C	43.000	0.000
A_06_P7108	YPR062W	144.000	0.000
A_06_P7109	YPR063C	148.000	0.000
A_06_P7110	YPR064W	29.000	0.000
A_06_P7111	YPR065W	26.000	0.000
A_06_P7112	YPR066W	59.000	0.000
A_06_P7113	YPR067W	41.000	0.000
A_06_P7114	YPR068C	180.000	0.000
A_06_P7115	YPR069C	22.000	0.000
A_06_P7116	YPR070W	201.000	0.000
A_06_P7117	YPR071W	20.000	0.000
A_06_P7118	YPR072W	30.000	0.000
A_06_P7119	YPR073C	14.000	0.000
A_06_P7120	YPR074C	57.000	0.000
A_06_P7121	YPR074W-A	16.000	0.000
A_06_P7122	YPR075C	9.000	0.000
A_06_P7123	YPR076W	26.000	0.000
A_06_P7124	YPR077C	10.000	0.000
A_06_P7125	YPR078C	270.000	0.000
A_06_P7126	YPR079W	56.000	0.000
A_06_P7127	YPR080W	40.200	2.775
A_06_P7128	YPR081C	5.000	0.000
A_06_P7129	YPR082C	79.000	0.000
A_06_P7130	YPR083W	38.000	0.000
A_06_P7131	YPR084W	15.000	0.000
A_06_P7132	YPR085C	78.000	0.000
A_06_P7133	YPR086W	7.000	0.000
A_06_P7134	YPR087W	11.000	0.000
A_06_P7135	YPR088C	348.000	0.000
A_06_P7136	YPR089W	88.200	10.232
A_06_P7137	YPR089W	280.800	10.232
A_06_P7138	YPR091C	38.000	0.000
A_06_P7139	YPR092W	4.000	0.000
A_06_P7140	YPR093C	46.000	0.000
A_06_P7141	YPR094W	88.000	0.000
A_06_P7142	YPR095C	169.000	0.000
A_06_P7143	YPR096C	332.000	0.000
A_06_P7144	YPR097W	617.000	0.000
A_06_P7145	YPR098C	30.000	0.000
A_06_P7146	YPR099C	9.000	0.000
A_06_P7147	YPR100W	81.000	0.000
A_06_P7148	YPR101W	1000.000	0.000
A_06_P7149	YPR102C	116.000	0.000
A_06_P7150	YPR103W	28.000	0.000
A_06_P7151	YPR104C	29.000	0.000
A_06_P7152	YPR105C	273.000	0.000

A_06_P7153	YPR106W	32.000	0.000
A_06_P7154	YPR107C	30.000	0.000
A_06_P7155	YPR108W	26.000	0.000
A_06_P7156	YPR109W	33.000	0.000
A_06_P7157	YPR110C	6.000	0.000
A_06_P7158	YPR111W	29.000	0.000
A_06_P7159	YPR112C	85.000	0.000
A_06_P7160	YPR113W	287.000	0.000
A_06_P7161	YPR114W	38.000	0.000
A_06_P7162	YPR115W	123.000	0.000
A_06_P7163	YPR116W	55.000	0.000
A_06_P7164	YPR117W	257.000	0.000
A_06_P7165	YPR118W	26.000	0.000
A_06_P7166	YPR119W	24.000	0.000
A_06_P7167	YPR120C	57.000	0.000
A_06_P7168	YPR121W	156.000	0.000
A_06_P7169	YPR122W	70.000	0.000
A_06_P7170	YPR123C	93.000	0.000
A_06_P7171	YPR124W	78.000	0.000
A_06_P7172	YPR125W	25.000	0.000
A_06_P7173	YPR126C	172.000	0.000
A_06_P7174	YPR127W	24.000	0.000
A_06_P7175	YPR128C	8.000	0.000
A_06_P7176	YPR129W	33.000	0.000
A_06_P7177	YPR130C	23.000	0.000
A_06_P7178	YPR131C	32.000	0.000
A_06_P7179	YPR132W	337.000	0.000
A_06_P7180	YPR133C	40.000	0.000
A_06_P7181	YPR133W-A	13.000	0.000
A_06_P7182	YPR134W	51.000	0.000
A_06_P7183	YPR135W	42.000	0.000
A_06_P7184	YPR136C	78.000	0.000
A_06_P7185	YPR137W	76.000	0.000
A_06_P7186	YPR138C	7.000	0.000
A_06_P7187	YPR139C	155.000	0.000
A_06_P7188	YOR108C-A	0.200	0.447
A_06_P7188	YPR140W	9.000	0.000
A_06_P7189	YPR141C	45.000	0.000
A_06_P7190	YPR142C	7.000	0.000
A_06_P7191	YPR143W	43.000	0.000
A_06_P7192	YPR144C	18.000	0.000
A_06_P7193	YPR145W	48.000	0.000
A_06_P7194	YPR146C	40.000	0.000
A_06_P7195	YPR147C	66.000	0.000
A_06_P7196	YPR148C	49.000	0.000
A_06_P7197	YPR149W	3.000	0.000
A_06_P7198	YPR150W	26.000	0.000
A_06_P7199	YPR151C	14.000	0.000
A_06_P7200	YPR152C	107.000	0.000
A_06_P7201	YPR153W	7.000	0.000
A_06_P7202	YPR154W	78.000	0.000
A_06_P7203	YPR155C	64.000	0.000
A_06_P7204	YPR156C	123.000	0.000
A_06_P7205	YPR157W	74.000	0.000
A_06_P7206	YPR158W	17.000	0.000

A_06_P7207	YPR159W	10.000	0.000
A_06_P7208	YPR160W	99.000	0.000
A_06_P7209	YPR161C	20.000	0.000
A_06_P7210	YPR162C	118.000	0.000
A_06_P7211	YPR163C	5.000	0.000
A_06_P7212	YPR164W	358.000	0.000
A_06_P7213	YPR165W	27.000	0.000
A_06_P7214	YPR166C	46.000	0.000
A_06_P7215	YPR167C	45.000	0.000
A_06_P7216	YPR168W	12.000	0.000
A_06_P7217	YPR169W	50.000	0.000
A_06_P7218	YPR170C	30.000	0.000
A_06_P7219	YPR171W	23.000	0.000
A_06_P7220	YPR172W	145.000	0.000
A_06_P7221	YPR173C	240.000	0.000
A_06_P7222	YPR174C	16.000	0.000
A_06_P7223	YPR175W	37.000	0.000
A_06_P7224	YPR176C	173.000	0.000
A_06_P7225	YPR177C	30.000	0.000
A_06_P7226	YPR178W	63.000	0.000
A_06_P7227	YPR179C	331.000	0.000
A_06_P7228	YPR180W	14.000	0.000
A_06_P7229	YPR181C	24.000	0.000
A_06_P7230	YPR182W	44.000	0.000
A_06_P7231	YPR183W	105.000	0.000
A_06_P7232	YPR184W	15.000	0.000
A_06_P7233	YPR185W	37.000	0.000
A_06_P7234	YPR186C	99.000	0.000
A_06_P7235	YPR187W	36.000	0.000
A_06_P7236	YPR188C	27.000	0.000
A_06_P7237	YPR189W	121.000	0.000
A_06_P7238	YPR190C	491.000	0.000
A_06_P7239	YPR191W	131.000	0.000
A_06_P7240	YPR192W	25.000	0.000
A_06_P7241	YPR193C	9.000	0.000
A_06_P7242	YPR194C	80.000	0.000
A_06_P7243	YPR195C	21.000	0.000
A_06_P7244	YPR196W	4.000	0.000
A_06_P7245	YPR197C	33.000	0.000
A_06_P7246	YPR198W	27.000	0.000
A_06_P7247	YPR199C	9.000	0.000
A_06_P7248	YPR200C	41.000	0.000
A_06_P7249	YPR201W	32.000	0.000
A_06_P7250	YPR202W	73.600	2.074
A_06_P7251	YFL065C	0.600	0.548
A_06_P7251	YHL049C	94.600	6.877
A_06_P7251	YPR203W	27.200	3.834
A_06_P7252	YPR204W	0.200	0.447
A_06_P7253	YER087C-B	157.000	0.000
A_06_P7254	YBR255C-A	26.000	0.000
A_06_P7255	YDR381C-A	5.000	0.000
A_06_P7256	YER074W-A	23.000	0.000
