# Supplementary material for: An efficient algorithm for the stochastic simulation of the hybridization of DNA to microarrays
Source: BMC Bioinformatics. 2009 Dec 10;10:411. doi: 10.1186/1471-2105-10-411 (PMC2805644; doi:10.1186/1471-2105-10-411)
Supplement: Additional file 3 — Arslan_Laurenzi_Supplemental. This file contains the populations of all hybrids (X) at equilibrium for the experiments described in section "Characterization of Cross Hybridization". Simulations of the hybridization of full length yeast cDNA to the Agilent probe set were conducted at 65°C. Initial cDNA populations (prior to hybridization) are specified in the worksheet "Initial Target Populations". There are 1000 copies of each of the 6256 Agilent probes per feature. The simulation is conducted at 0.275 nL. The results of five replicate simulations are provided - differences between the results of each run are due to the probabilistic nature of chemical reaction. Data are organized by row and column: for instance, there are thirteen hybrids between A_06_P1002 and Q0010 at the end of the first replicate simulation (Run number 1) [file 1471-2105-10-411-S3.PDF]

This file contains a list of Agilent reporter names and the yeast ORFs targeted by each. The sequences of the Agilent probes may be obtained from https://earray.chem.agilent.com/earray/

Agilent Probe	Targeted Yeast ORF
A_06_P1001	Q0010
A_06_P1002	Q0017
A_06_P1003	Q0045
A_06_P1004	Q0050
A_06_P1005	Q0055
A_06_P1006	Q0060
A_06_P1007	Q0065
A_06_P1008	Q0070
A_06_P1009	Q0075
A_06_P1010	Q0080
A_06_P1011	Q0085
A_06_P1012	Q0105
A_06_P1013	Q0110
A_06_P1014	Q0115
A_06_P1015	Q0120
A_06_P1016	Q0130
A_06_P1017	Q0140
A_06_P1018	Q0160
A_06_P1019	Q0182
A_06_P1020	Q0250
A_06_P1021	Q0255
A_06_P1022	Q0275
A_06_P1023	Q0297
A_06_P1024	YAL001C
A_06_P1025	YAL002W
A_06_P1026	YAL003W
A_06_P1027	YAL004W
A_06_P1028	YAL005C
A_06_P1029	YAL007C
A_06_P1030	YAL008W
A_06_P1031	YAL009W
A_06_P1032	YAL010C
A_06_P1033	YAL011W
A_06_P1034	YAL012W
A_06_P1035	YAL013W
A_06_P1036	YAL014C
A_06_P1037	YAL015C
A_06_P1038	YAL016W
A_06_P1039	YAL017W
A_06_P1040	YAL018C
A_06_P1041	YAL019W
A_06_P1042	YAL020C
A_06_P1043	YAL021C
A_06_P1044	YAL022C
A_06_P1045	YAL023C
A_06_P1046	YAL024C
A_06_P1047	YAL025C
A_06_P1048	YAL026C
A_06_P1049	YAL027W
A_06_P1050	YAL028W
A_06_P1051	YAL029C
A_06_P1052	YAL030W
A_06_P1053	YAL031C
A_06_P1054	YAL032C

A_06_P1055	YAL033W
A_06_P1056	YAL034C
A_06_P1058	YAL034C-B
A_06_P1057	YAL034W-A
A_06_P1059	YAL035W
A_06_P1060	YAL036C
A_06_P1061	YAL037W
A_06_P1062	YAL038W
A_06_P1063	YAL039C
A_06_P1064	YAL040C
A_06_P1065	YAL041W
A_06_P1068	YAL042C-A
A_06_P1066	YAL042W
A_06_P1067	YAL043C
A_06_P1069	YAL044C
A_06_P1070	YAL044W-A
A_06_P1071	YAL045C
A_06_P1072	YAL046C
A_06_P1073	YAL047C
A_06_P1074	YAL048C
A_06_P1075	YAL049C
A_06_P1076	YAL051W
A_06_P1077	YAL053W
A_06_P1078	YAL054C
A_06_P1079	YAL055W
A_06_P1081	YAL056C-A
A_06_P1080	YAL056W
A_06_P1082	YAL058W
A_06_P1083	YAL059W
A_06_P1084	YAL060W
A_06_P1085	YAL061W
A_06_P1086	YAL062W
A_06_P1087	YAL063C
A_06_P1088	YAL064C-A
A_06_P1089	YAL064W
A_06_P1090	YAL064W-B
A_06_P1091	YAL065C
A_06_P1092	YAL066W
A_06_P1093	YAL067C
A_06_P1094	YAL068C
A_06_P1095	YAL069W
A_06_P1096	YAR002C-A
A_06_P1097	YAR002W
A_06_P1098	YAR003W
A_06_P1099	YAR007C
A_06_P1100	YAR008W
A_06_P1101	YAR014C
A_06_P1102	YAR015W
A_06_P1103	YAR018C
A_06_P1104	YAR019C
A_06_P1105	YAR020C
A_06_P1106	YAR023C
A_06_P1107	YAR027W
A_06_P1108	YAR028W
A_06_P1109	YAR029W

A_06_P1110	YAR030C
A_06_P1111	YAR031W
A_06_P1112	YAR033W
A_06_P1113	YAR035W
A_06_P1114	YAR042W
A_06_P1115	YAR042W
A_06_P1116	YAR047C
A_06_P1117	YAR050W
A_06_P1118	YAR053W
A_06_P1119	YAR060C
A_06_P1120	YAR061W
A_06_P1121	YAR062W
A_06_P1122	YAR064W
A_06_P1123	YAR066W
A_06_P1124	YAR068W
A_06_P1125	YAR069C
A_06_P1126	YAR070C
A_06_P1127	YAR071W
A_06_P1128	YAR073W
A_06_P1129	YAR075W
A_06_P1130	YBL001C
A_06_P1131	YBL002W
A_06_P1132	YBL003C
A_06_P1133	YBL004W
A_06_P1134	YBL005W
A_06_P1135	YBL006C
A_06_P1136	YBL007C
A_06_P1137	YBL008W
A_06_P1138	YBL009W
A_06_P1139	YBL010C
A_06_P1140	YBL011W
A_06_P1141	YBL012C
A_06_P1142	YBL013W
A_06_P1143	YBL014C
A_06_P1144	YBL015W
A_06_P1145	YBL016W
A_06_P1146	YBL017C
A_06_P1147	YBL018C
A_06_P1148	YBL019W
A_06_P1149	YBL020W
A_06_P1150	YBL021C
A_06_P1151	YBL022C
A_06_P1152	YBL023C
A_06_P1153	YBL024W
A_06_P1154	YBL025W
A_06_P1155	YBL026W
A_06_P1156	YBL027W
A_06_P1157	YBL028C
A_06_P1158	YBL029C-A
A_06_P1159	YBL029W
A_06_P1160	YBL030C
A_06_P1161	YBL031W
A_06_P1162	YBL032W
A_06_P1163	YBL033C
A_06_P1164	YBL034C

A_06_P1165	YBL035C
A_06_P1166	YBL036C
A_06_P1167	YBL037W
A_06_P1168	YBL038W
A_06_P1169	YBL039C
A_06_P1170	YBL040C
A_06_P1171	YBL041W
A_06_P1172	YBL042C
A_06_P1173	YBL043W
A_06_P1174	YBL044W
A_06_P1175	YBL045C
A_06_P1176	YBL046W
A_06_P1177	YBL047C
A_06_P1178	YBL048W
A_06_P1179	YBL049W
A_06_P1180	YBL050W
A_06_P1181	YBL051C
A_06_P1182	YBL052C
A_06_P1183	YBL053W
A_06_P1184	YBL054W
A_06_P1185	YBL055C
A_06_P1186	YBL056W
A_06_P1187	YBL057C
A_06_P1188	YBL058W
A_06_P1189	YBL059C-A
A_06_P1190	YBL059W
A_06_P1191	YBL060W
A_06_P1192	YBL061C
A_06_P1193	YBL062W
A_06_P1194	YBL063W
A_06_P1195	YBL064C
A_06_P1196	YBL065W
A_06_P1197	YBL066C
A_06_P1198	YBL067C
A_06_P1199	YBL068W
A_06_P1200	YBL069W
A_06_P1201	YBL070C
A_06_P1202	YBL071C
A_06_P1203	YBL071W-A
A_06_P1204	YBL072C
A_06_P1205	YBL073W
A_06_P1206	YBL074C
A_06_P1207	YBL075C
A_06_P1208	YBL076C
A_06_P1209	YBL077W
A_06_P1210	YBL078C
A_06_P1211	YBL079W
A_06_P1212	YBL080C
A_06_P1213	YBL081W
A_06_P1214	YBL082C
A_06_P1215	YBL083C
A_06_P1216	YBL084C
A_06_P1217	YBL085W
A_06_P1218	YBL086C
A_06_P1219	YBL087C

A_06_P1220	YBL088C
A_06_P1221	YBL089W
A_06_P1222	YBL090W
A_06_P1223	YBL091C
A_06_P1224	YBL091C-A
A_06_P1225	YBL092W
A_06_P1226	YBL093C
A_06_P1227	YBL094C
A_06_P1228	YBL095W
A_06_P1229	YBL096C
A_06_P1230	YBL097W
A_06_P1231	YBL098W
A_06_P1232	YBL099W
A_06_P1233	YBL100C
A_06_P1234	YBL101C
A_06_P1235	YBL102W
A_06_P1236	YBL103C
A_06_P1237	YBL104C
A_06_P1238	YBL105C
A_06_P1239	YBL106C
A_06_P1240	YBL107C
A_06_P1241	YBL107W-A
A_06_P1242	YBL108C-A
A_06_P1243	YBL108W
A_06_P1244	YBL109W
A_06_P1245	YBL111C
A_06_P1246	YBL112C
A_06_P1247	YBL113C
A_06_P1248	YBR001C
A_06_P1249	YBR002C
A_06_P1250	YBR003W
A_06_P1251	YBR004C
A_06_P1252	YBR005W
A_06_P1253	YBR006W
A_06_P1254	YBR007C
A_06_P1255	YBR008C
A_06_P1256	YBR009C
A_06_P1257	YBR010W
A_06_P1258	YBR011C
A_06_P1259	YBR012C
A_06_P1260	YBR013C
A_06_P1261	YBR014C
A_06_P1262	YBR015C
A_06_P1263	YBR016W
A_06_P1264	YBR017C
A_06_P1265	YBR018C
A_06_P1266	YBR019C
A_06_P1267	YBR020W
A_06_P1268	YBR021W
A_06_P1269	YBR022W
A_06_P1270	YBR023C
A_06_P1271	YBR024W
A_06_P1272	YBR025C
A_06_P1273	YBR026C
A_06_P1274	YBR027C

A_06_P1275	YBR028C
A_06_P1276	YBR029C
A_06_P1277	YBR030W
A_06_P1278	YBR031W
A_06_P1279	YBR032W
A_06_P1280	YBR033W
A_06_P1281	YBR034C
A_06_P1282	YBR035C
A_06_P1283	YBR036C
A_06_P1284	YBR037C
A_06_P1285	YBR038W
A_06_P1286	YBR039W
A_06_P1287	YBR040W
A_06_P1288	YBR041W
A_06_P1289	YBR042C
A_06_P1290	YBR043C
A_06_P1291	YBR044C
A_06_P1292	YBR045C
A_06_P1293	YBR046C
A_06_P1294	YBR047W
A_06_P1295	YBR048W
A_06_P1296	YBR049C
A_06_P1297	YBR050C
A_06_P1298	YBR051W
A_06_P1299	YBR052C
A_06_P1300	YBR053C
A_06_P1301	YBR054W
A_06_P1302	YBR055C
A_06_P1303	YBR056W
A_06_P1304	YBR057C
A_06_P1305	YBR058C
A_06_P1306	YBR058C-A
A_06_P1307	YBR059C
A_06_P1308	YBR060C
A_06_P1309	YBR061C
A_06_P1310	YBR062C
A_06_P1311	YBR063C
A_06_P1312	YBR064W
A_06_P1313	YBR065C
A_06_P1314	YBR066C
A_06_P1315	YBR067C
A_06_P1316	YBR068C
A_06_P1317	YBR069C
A_06_P1318	YBR070C
A_06_P1319	YBR071W
A_06_P1320	YBR072W
A_06_P1321	YBR073W
A_06_P1322	YBR074W
A_06_P1323	YBR074W
A_06_P1324	YBR076W
A_06_P1325	YBR077C
A_06_P1326	YBR078W
A_06_P1327	YBR079C
A_06_P1328	YBR080C
A_06_P1329	YBR081C

A_06_P1330	YBR082C
A_06_P1331	YBR083W
A_06_P1332	YBR084C-A
A_06_P1333	YBR084W
A_06_P1334	YBR085C-A
A_06_P1335	YBR085W
A_06_P1336	YBR086C
A_06_P1337	YBR087W
A_06_P1338	YBR088C
A_06_P1339	YBR089C-A
A_06_P1340	YBR089W
A_06_P1341	YBR090C
A_06_P1342	YBR091C
A_06_P1343	YBR092C
A_06_P1344	YBR093C
A_06_P1345	YBR094W
A_06_P1346	YBR095C
A_06_P1347	YBR096W
A_06_P1348	YBR097W
A_06_P1349	YBR098W
A_06_P1350	YBR099C
A_06_P1351	YBR098W
A_06_P1352	YBR101C
A_06_P1353	YBR102C
A_06_P1354	YBR103C-A
A_06_P1355	YBR103W
A_06_P1356	YBR104W
A_06_P1357	YBR105C
A_06_P1358	YBR106W
A_06_P1359	YBR107C
A_06_P1360	YBR108W
A_06_P1361	YBR109C
A_06_P1362	YBR110W
A_06_P1363	YBR111C
A_06_P1364	YBR112C
A_06_P1365	YBR113W
A_06_P1366	YBR114W
A_06_P1367	YBR115C
A_06_P1368	YBR116C
A_06_P1369	YBR117C
A_06_P1370	YBR118W
A_06_P1371	YBR119W
A_06_P1372	YBR120C
A_06_P1373	YBR121C
A_06_P1374	YBR122C
A_06_P1375	YBR123C
A_06_P1376	YBR124W
A_06_P1377	YBR125C
A_06_P1378	YBR126C
A_06_P1379	YBR127C
A_06_P1380	YBR128C
A_06_P1381	YBR129C
A_06_P1382	YBR130C
A_06_P1383	YBR131W
A_06_P1384	YBR132C

A_06_P1385	YBR133C
A_06_P1386	YBR134W
A_06_P1387	YBR135W
A_06_P1388	YBR136W
A_06_P1389	YBR137W
A_06_P1390	YBR138C
A_06_P1391	YBR139W
A_06_P1392	YBR140C
A_06_P1393	YBR141C
A_06_P1394	YBR142W
A_06_P1395	YBR143C
A_06_P1396	YBR144C
A_06_P1397	YBR145W
A_06_P1398	YBR146W
A_06_P1399	YBR147W
A_06_P1400	YBR148W
A_06_P1401	YBR149W
A_06_P1402	YBR150C
A_06_P1403	YBR151W
A_06_P1404	YBR152W
A_06_P1405	YBR153W
A_06_P1406	YBR154C
A_06_P1407	YBR155W
A_06_P1408	YBR156C
A_06_P1409	YBR157C
A_06_P1410	YBR158W
A_06_P1411	YBR159W
A_06_P1412	YBR160W
A_06_P1413	YBR161W
A_06_P1414	YBR162C
A_06_P1415	YBR162W-A
A_06_P1416	YBR163W
A_06_P1417	YBR164C
A_06_P1418	YBR165W
A_06_P1419	YBR166C
A_06_P1420	YBR167C
A_06_P1421	YBR168W
A_06_P1422	YBR169C
A_06_P1423	YBR170C
A_06_P1424	YBR171W
A_06_P1425	YBR172C
A_06_P1426	YBR173C
A_06_P1427	YBR174C
A_06_P1428	YBR175W
A_06_P1429	YBR176W
A_06_P1430	YBR177C
A_06_P1431	YBR178W
A_06_P1432	YBR179C
A_06_P1433	YBR180W
A_06_P1434	YBR181C
A_06_P1435	YBR182C
A_06_P1436	YBR183W
A_06_P1437	YBR184W
A_06_P1438	YBR185C
A_06_P1439	YBR186W

A_06_P1440	YBR187W
A_06_P1441	YBR188C
A_06_P1442	YBR189W
A_06_P1443	YBR190W
A_06_P1444	YBR191W
A_06_P1445	YBR192W
A_06_P1446	YBR193C
A_06_P1447	YBR194W
A_06_P1448	YBR195C
A_06_P1449	YBR196C
A_06_P1450	YBR197C
A_06_P1451	YBR198C
A_06_P1452	YBR199W
A_06_P1453	YBR200W
A_06_P1454	YBR201W
A_06_P1455	YBR202W
A_06_P1456	YBR203W
A_06_P1457	YBR204C
A_06_P1458	YBR205W
A_06_P1459	YBR206W
A_06_P1460	YBR207W
A_06_P1461	YBR208C
A_06_P1462	YBR209W
A_06_P1463	YBR210W
A_06_P1464	YBR211C
A_06_P1465	YBR212W
A_06_P1466	YBR213W
A_06_P1467	YBR214W
A_06_P1468	YBR215W
A_06_P1469	YBR216C
A_06_P1470	YBR217W
A_06_P1471	YBR218C
A_06_P1472	YBR219C
A_06_P1473	YBR220C
A_06_P1474	YBR221C
A_06_P1475	YBR222C
A_06_P1476	YBR223C
A_06_P1477	YBR224W
A_06_P1478	YBR225W
A_06_P1479	YBR226C
A_06_P1480	YBR227C
A_06_P1481	YBR228W
A_06_P1482	YBR229C
A_06_P1483	YBR230C
A_06_P1484	YBR231C
A_06_P1485	YBR232C
A_06_P1486	YBR233W
A_06_P1487	YBR233W-A
A_06_P1488	YBR234C
A_06_P1489	YBR235W
A_06_P1490	YBR236C
A_06_P1491	YBR237W
A_06_P1492	YBR238C
A_06_P1493	YBR239C
A_06_P1494	YBR240C

A_06_P1495	YBR241C
A_06_P1496	YBR242W
A_06_P1497	YBR243C
A_06_P1498	YBR244W
A_06_P1499	YBR245C
A_06_P1500	YBR246W
A_06_P1501	YBR247C
A_06_P1502	YBR248C
A_06_P1503	YBR249C
A_06_P1504	YBR250W
A_06_P1505	YBR251W
A_06_P1506	YBR252W
A_06_P1507	YBR253W
A_06_P1508	YBR254C
A_06_P7254	YBR255C-A
A_06_P1509	YBR255W
A_06_P1510	YBR256C
A_06_P1511	YBR257W
A_06_P1512	YBR258C
A_06_P1513	YBR259W
A_06_P1514	YBR260C
A_06_P1515	YBR261C
A_06_P1516	YBR262C
A_06_P1517	YBR263W
A_06_P1518	YBR264C
A_06_P1519	YBR265W
A_06_P1520	YBR266C
A_06_P1521	YBR267W
A_06_P1522	YBR268W
A_06_P1523	YBR269C
A_06_P1524	YBR270C
A_06_P1525	YBR271W
A_06_P1526	YBR272C
A_06_P1527	YBR273C
A_06_P1528	YBR274W
A_06_P1529	YBR275C
A_06_P1530	YBR276C
A_06_P1531	YBR277C
A_06_P1532	YBR278W
A_06_P1533	YBR279W
A_06_P1534	YBR280C
A_06_P1535	YBR281C
A_06_P1536	YBR282W
A_06_P1537	YBR283C
A_06_P1538	YBR284W
A_06_P1539	YBR285W
A_06_P1540	YBR286W
A_06_P1541	YBR287W
A_06_P1542	YBR288C
A_06_P1543	YBR289W
A_06_P1544	YBR290W
A_06_P1545	YBR291C
A_06_P1546	YBR292C
A_06_P1547	YBR293W
A_06_P1548	YBR294W

A_06_P1549	YBR295W
A_06_P1550	YBR296C
A_06_P1551	YBR297W
A_06_P1552	YBR298C
A_06_P1553	YBR299W
A_06_P1554	YBR300C
A_06_P1555	YBR301W
A_06_P1556	YBR302C
A_06_P1557	YCL001W
A_06_P1558	YCL001W-A
A_06_P1559	YCL001W-B
A_06_P1560	YCL002C
A_06_P1561	YCL004W
A_06_P1562	YCL005W
A_06_P1563	YCL007C
A_06_P1564	YCL008C
A_06_P1565	YCL009C
A_06_P1566	YCL010C
A_06_P1567	YCL011C
A_06_P1568	YCL014W
A_06_P1569	YCL016C
A_06_P1570	YCL017C
A_06_P1571	YCL018W
A_06_P1572	YCL021W-A
A_06_P1573	YCL022C
A_06_P1574	YCL023C
A_06_P1575	YCL024W
A_06_P1576	YCL025C
A_06_P1577	YCL026C-A
A_06_P1578	YCL026C-B
A_06_P1579	YCL027W
A_06_P1580	YCL028W
A_06_P1581	YCL029C
A_06_P1582	YCL030C
A_06_P1583	YCL031C
A_06_P1584	YCL032W
A_06_P1585	YCL033C
A_06_P1586	YCL034W
A_06_P1587	YCL035C
A_06_P1588	YCL036W
A_06_P1589	YCL037C
A_06_P1590	YCL038C
A_06_P1591	YCL039W
A_06_P1592	YCL040W
A_06_P1593	YCL041C
A_06_P1594	YCL042W
A_06_P1595	YCL043C
A_06_P1596	YCL044C
A_06_P1597	YCL045C
A_06_P1598	YCL046W
A_06_P1599	YCL047C
A_06_P1600	YCL048W
A_06_P1601	YCL049C
A_06_P1602	YCL050C
A_06_P1603	YCL051W

A_06_P1604	YCL052C
A_06_P1605	YCL054W
A_06_P1606	YCL055W
A_06_P1607	YCL056C
A_06_P1608	YCL057C-A
A_06_P1609	YCL057W
A_06_P1610	YCL058C
A_06_P1611	YCL059C
A_06_P1612	YCL061C
A_06_P1613	YCL063W
A_06_P1614	YCL064C
A_06_P1615	YCL065W
A_06_P1616	YCL066W
A_06_P1617	YCL067C
A_06_P1618	YCL068C
A_06_P1619	YCL069W
A_06_P1620	YCL073C
A_06_P1621	YCL076W
A_06_P1622	YCR001W
A_06_P1623	YCR002C
A_06_P1624	YCR003W
A_06_P1625	YCR004C
A_06_P1626	YCR005C
A_06_P1627	YCR006C
A_06_P1628	YCR007C
A_06_P1629	YCR008W
A_06_P1630	YCR009C
A_06_P1631	YCR010C
A_06_P1632	YCR011C
A_06_P1633	YCR012W
A_06_P1634	YCR013C
A_06_P1635	YCR014C
A_06_P1636	YCR015C
A_06_P1637	YCR016W
A_06_P1638	YCR017C
A_06_P1639	YCR018C
A_06_P1640	YCR018C-A
A_06_P1641	YCR019W
A_06_P1642	YCR020C
A_06_P1643	YCR020C-A
A_06_P1644	YCR020W-B
A_06_P1645	YCR021C
A_06_P1646	YCR022C
A_06_P1647	YCR023C
A_06_P1648	YCR024C
A_06_P1649	YCR024C-A
A_06_P1650	YCR025C
A_06_P1651	YCR026C
A_06_P1652	YCR027C
A_06_P1653	YCR028C
A_06_P1654	YCR028C-A
A_06_P1655	YCR030C
A_06_P1656	YCR031C
A_06_P1657	YCR032W
A_06_P1658	YCR033W

A_06_P1659	YCR034W
A_06_P1660	YCR035C
A_06_P1661	YCR036W
A_06_P1662	YCR037C
A_06_P1663	YCR038C
A_06_P1664	YCR038W-A
A_06_P1665	YCR039C
A_06_P1666	YCR040W
A_06_P1667	YCR041W
A_06_P1668	YCR042C
A_06_P1669	YCR043C
A_06_P1670	YCR044C
A_06_P1671	YCR045C
A_06_P1672	YCR046C
A_06_P1673	YCR047C
A_06_P1674	YCR048W
A_06_P1675	YCR049C
A_06_P1676	YCR050C
A_06_P1677	YCR051W
A_06_P1678	YCR052W
A_06_P1679	YCR053W
A_06_P1680	YCR054C
A_06_P1681	YCR057C
A_06_P1682	YCR059C
A_06_P1683	YCR060W
A_06_P1684	YCR061W
A_06_P1685	YCR063W
A_06_P1686	YCR064C
A_06_P1687	YCR065W
A_06_P1688	YCR066W
A_06_P1689	YCR067C
A_06_P1690	YCR068W
A_06_P1691	YCR069W
A_06_P1692	YCR071C
A_06_P1693	YCR072C
A_06_P1694	YCR073C
A_06_P1695	YCR073W-A
A_06_P1696	YCR075C
A_06_P1697	YCR076C
A_06_P1698	YCR077C
A_06_P1699	YCR079W
A_06_P1700	YCR081W
A_06_P1701	YCR082W
A_06_P1702	YCR083W
A_06_P1703	YCR084C
A_06_P1704	YCR085W
A_06_P1705	YCR086W
A_06_P1706	YCR087C-A
A_06_P1707	YCR087W
A_06_P1708	YCR088W
A_06_P1709	YCR089W
A_06_P1710	YCR090C
A_06_P1711	YCR091W
A_06_P1712	YCR092C
A_06_P1713	YCR093W

A_06_P1714	YCR094W
A_06_P1715	YCR095C
A_06_P1716	YCR096C
A_06_P1717	YCR097W
A_06_P1718	YCR097W-A
A_06_P1719	YCR098C
A_06_P1720	YCR099C
A_06_P1721	YCR100C
A_06_P1722	YCR101C
A_06_P1723	YCR102C
A_06_P1724	YCR102W-A
A_06_P1725	YCR104W
A_06_P1726	YCR105W
A_06_P1727	YCR106W
A_06_P1728	YCR107W
A_06_P1729	YDL001W
A_06_P1730	YDL002C
A_06_P1731	YDL003W
A_06_P1732	YDL004W
A_06_P1733	YDL005C
A_06_P1734	YDL006W
A_06_P1735	YDL007W
A_06_P1736	YDL008W
A_06_P1737	YDL009C
A_06_P1738	YDL010W
A_06_P1739	YDL011C
A_06_P1740	YDL012C
A_06_P1741	YDL013W
A_06_P1742	YDL014W
A_06_P1743	YDL015C
A_06_P1744	YDL016C
A_06_P1745	YDL017W
A_06_P1746	YDL018C
A_06_P1747	YDL019C
A_06_P1748	YDL020C
A_06_P1749	YDL021W
A_06_P1750	YDL022W
A_06_P1751	YDL023C
A_06_P1752	YDL024C
A_06_P1753	YDL025C
A_06_P1754	YDL026W
A_06_P1755	YDL027C
A_06_P1756	YDL028C
A_06_P1757	YDL029W
A_06_P1758	YDL030W
A_06_P1759	YDL031W
A_06_P1760	YDL032W
A_06_P1761	YDL033C
A_06_P1762	YDL034W
A_06_P1763	YDL035C
A_06_P1764	YDL036C
A_06_P1765	YDL037C
A_06_P1766	YDL038C
A_06_P1767	YDL039C
A_06_P1768	YDL040C

A_06_P1769	YDL041W
A_06_P1770	YDL042C
A_06_P1771	YDL043C
A_06_P1772	YDL044C
A_06_P1773	YDL045C
A_06_P1774	YDL045W-A
A_06_P1775	YDL046W
A_06_P1776	YDL047W
A_06_P1777	YDL048C
A_06_P1778	YDL049C
A_06_P1779	YDL050C
A_06_P1780	YDL051W
A_06_P1781	YDL052C
A_06_P1782	YDL053C
A_06_P1783	YDL054C
A_06_P1784	YDL055C
A_06_P1785	YDL056W
A_06_P1786	YDL057W
A_06_P1787	YDL058W
A_06_P1788	YDL059C
A_06_P1789	YDL060W
A_06_P1790	YDL061C
A_06_P1791	YDL062W
A_06_P1792	YDL063C
A_06_P1793	YDL064W
A_06_P1794	YDL065C
A_06_P1795	YDL066W
A_06_P1796	YDL067C
A_06_P1797	YDL068W
A_06_P1798	YDL069C
A_06_P1799	YDL070W
A_06_P1800	YDL071C
A_06_P1801	YDL072C
A_06_P1802	YDL073W
A_06_P1803	YDL074C
A_06_P1804	YDL075W
A_06_P1805	YDL076C
A_06_P1806	YDL077C
A_06_P1807	YDL078C
A_06_P1808	YDL079C
A_06_P1809	YDL080C
A_06_P1810	YDL081C
A_06_P1811	YDL082W
A_06_P1812	YDL083C
A_06_P1813	YDL084W
A_06_P1814	YDL085C-A
A_06_P1815	YDL085W
A_06_P1816	YDL086W
A_06_P1817	YDL087C
A_06_P1818	YDL088C
A_06_P1819	YDL089W
A_06_P1820	YDL090C
A_06_P1821	YDL091C
A_06_P1822	YDL092W
A_06_P1823	YDL093W

A_06_P1824	YDL094C
A_06_P1825	YDL095W
A_06_P1826	YDL096C
A_06_P1827	YDL097C
A_06_P1828	YDL098C
A_06_P1829	YDL099W
A_06_P1830	YDL100C
A_06_P1831	YDL101C
A_06_P1832	YDL102W
A_06_P1833	YDL103C
A_06_P1834	YDL104C
A_06_P1835	YDL105W
A_06_P1836	YDL106C
A_06_P1837	YDL107W
A_06_P1838	YDL108W
A_06_P1839	YDL109C
A_06_P1840	YDL110C
A_06_P1841	YDL111C
A_06_P1842	YDL112W
A_06_P1843	YDL113C
A_06_P1844	YDL114W
A_06_P1845	YDL114W-A
A_06_P1846	YDL115C
A_06_P1847	YDL116W
A_06_P1848	YDL117W
A_06_P1849	YDL118W
A_06_P1850	YDL119C
A_06_P1851	YDL120W
A_06_P1852	YDL121C
A_06_P1853	YDL122W
A_06_P1854	YDL123W
A_06_P1855	YDL124W
A_06_P1856	YDL125C
A_06_P1857	YDL126C
A_06_P1858	YDL127W
A_06_P1859	YDL128W
A_06_P1860	YDL129W
A_06_P1861	YDL130W
A_06_P1862	YDL130W-A
A_06_P1863	YDL131W
A_06_P1864	YDL132W
A_06_P1865	YDL133C-A
A_06_P1866	YDL133W
A_06_P1867	YDL134C
A_06_P1868	YDL135C
A_06_P1869	YDL136W
A_06_P1870	YDL137W
A_06_P1871	YDL138W
A_06_P1872	YDL139C
A_06_P1873	YDL140C
A_06_P1874	YDL141W
A_06_P1875	YDL142C
A_06_P1876	YDL143W
A_06_P1877	YDL144C
A_06_P1878	YDL145C

A_06_P1879	YDL146W
A_06_P1880	YDL147W
A_06_P1881	YDL148C
A_06_P1882	YDL149W
A_06_P1883	YDL150W
A_06_P1884	YDL151C
A_06_P1885	YDL152W
A_06_P1886	YDL153C
A_06_P1887	YDL154W
A_06_P1888	YDL155W
A_06_P1889	YDL156W
A_06_P1890	YDL157C
A_06_P1891	YDL158C
A_06_P1892	YDL159W
A_06_P1893	YDL159W-A
A_06_P1894	YDL160C
A_06_P1895	YDL161W
A_06_P1896	YDL162C
A_06_P1897	YDL163W
A_06_P1898	YDL164C
A_06_P1899	YDL165W
A_06_P1900	YDL166C
A_06_P1901	YDL167C
A_06_P1902	YDL168W
A_06_P1903	YDL169C
A_06_P1904	YDL170W
A_06_P1905	YDL171C
A_06_P1906	YDL172C
A_06_P1907	YDL173W
A_06_P1908	YDL174C
A_06_P1909	YDL175C
A_06_P1910	YDL176W
A_06_P1911	YDL177C
A_06_P1912	YDL178W
A_06_P1913	YDL179W
A_06_P1914	YDL180W
A_06_P1915	YDL181W
A_06_P1916	YDL182W
A_06_P1917	YDL183C
A_06_P1918	YDL184C
A_06_P1919	YDL185C-A
A_06_P1920	YDL185W
A_06_P1921	YDL186W
A_06_P1922	YDL187C
A_06_P1923	YDL188C
A_06_P1924	YDL189W
A_06_P1925	YDL190C
A_06_P1926	YDL191W
A_06_P1927	YDL192W
A_06_P1928	YDL193W
A_06_P1929	YDL194W
A_06_P1930	YDL195W
A_06_P1931	YDL196W
A_06_P1932	YDL197C
A_06_P1933	YDL198C

A_06_P1934	YDL199C
A_06_P1935	YDL200C
A_06_P1936	YDL201W
A_06_P1937	YDL202W
A_06_P1938	YDL203C
A_06_P1939	YDL204W
A_06_P1940	YDL205C
A_06_P1941	YDL206W
A_06_P1942	YDL207W
A_06_P1943	YDL208W
A_06_P1944	YDL209C
A_06_P1945	YDL210W
A_06_P1946	YDL211C
A_06_P1947	YDL212W
A_06_P1948	YDL213C
A_06_P1949	YDL214C
A_06_P1950	YDL215C
A_06_P1951	YDL216C
A_06_P1952	YDL217C
A_06_P1953	YDL218W
A_06_P1954	YDL219W
A_06_P1955	YDL220C
A_06_P1956	YDL221W
A_06_P1957	YDL222C
A_06_P1958	YDL223C
A_06_P1959	YDL224C
A_06_P1960	YDL225W
A_06_P1961	YDL226C
A_06_P1962	YDL227C
A_06_P1963	YDL228C
A_06_P1964	YDL229W
A_06_P1965	YDL230W
A_06_P1966	YDL231C
A_06_P1967	YDL232W
A_06_P1968	YDL233W
A_06_P1969	YDL234C
A_06_P1970	YDL235C
A_06_P1971	YDL236W
A_06_P1972	YDL237W
A_06_P1973	YDL238C
A_06_P1974	YDL239C
A_06_P1975	YDL240C-A
A_06_P1976	YDL240W
A_06_P1977	YDL241W
A_06_P1978	YDL242W
A_06_P1979	YDL243C
A_06_P1980	YDL244W
A_06_P1981	YDL245C
A_06_P1982	YDL246C
A_06_P1983	YDL247W
A_06_P1984	YDL247W-A
A_06_P1985	YDL248W
A_06_P1986	YDR001C
A_06_P1987	YDR002W
A_06_P1988	YDR003W

A_06_P1989	YDR004W
A_06_P1990	YDR005C
A_06_P1991	YDR006C
A_06_P1992	YDR007W
A_06_P1993	YDR008C
A_06_P1994	YDR009W
A_06_P1995	YDR010C
A_06_P1996	YDR011W
A_06_P1997	YDR012W
A_06_P1998	YDR013W
A_06_P1999	YDR014W
A_06_P2000	YDR015C
A_06_P2001	YDR016C
A_06_P2002	YDR017C
A_06_P2003	YDR018C
A_06_P2004	YDR019C
A_06_P2005	YDR020C
A_06_P2006	YDR021W
A_06_P2007	YDR022C
A_06_P2008	YDR023W
A_06_P2009	YDR024W
A_06_P2010	YDR025W
A_06_P2011	YDR026C
A_06_P2012	YDR027C
A_06_P2013	YDR028C
A_06_P2014	YDR029W
A_06_P2015	YDR030C
A_06_P2016	YDR031W
A_06_P2017	YDR032C
A_06_P2018	YDR033W
A_06_P2019	YDR034C
A_06_P2020	YDR034C-A
A_06_P2021	YDR034W-B
A_06_P2022	YDR035W
A_06_P2023	YDR036C
A_06_P2024	YDR037W
A_06_P2025	YDR038C
A_06_P2026	YDR039C
A_06_P2027	YDR040C
A_06_P2028	YDR041W
A_06_P2029	YDR042C
A_06_P2030	YDR043C
A_06_P2031	YDR044W
A_06_P2032	YDR045C
A_06_P2033	YDR046C
A_06_P2034	YDR047W
A_06_P2035	YDR048C
A_06_P2036	YDR049W
A_06_P2037	YDR050C
A_06_P2038	YDR051C
A_06_P2039	YDR052C
A_06_P2040	YDR053W
A_06_P2041	YDR054C
A_06_P2042	YDR055W
A_06_P2043	YDR056C

A_06_P2044	YDR057W
A_06_P2045	YDR058C
A_06_P2046	YDR059C
A_06_P2047	YDR060W
A_06_P2048	YDR061W
A_06_P2049	YDR062W
A_06_P2050	YDR063W
A_06_P2051	YDR064W
A_06_P2052	YDR065W
A_06_P2053	YDR066C
A_06_P2054	YDR067C
A_06_P2055	YDR068W
A_06_P2056	YDR069C
A_06_P2057	YDR070C
A_06_P2058	YDR071C
A_06_P2059	YDR072C
A_06_P2060	YDR073W
A_06_P2061	YDR074W
A_06_P2062	YDR075W
A_06_P2063	YDR076W
A_06_P2064	YDR077W
A_06_P2065	YDR078C
A_06_P2066	YDR079C-A
A_06_P2067	YDR079W
A_06_P2068	YDR080W
A_06_P2069	YDR081C
A_06_P2070	YDR082W
A_06_P2071	YDR083W
A_06_P2072	YDR084C
A_06_P2073	YDR085C
A_06_P2074	YDR086C
A_06_P2075	YDR087C
A_06_P2076	YDR088C
A_06_P2077	YDR089W
A_06_P2078	YDR090C
A_06_P2079	YDR091C
A_06_P2080	YDR092W
A_06_P2081	YDR093W
A_06_P2082	YDR094W
A_06_P2083	YDR095C
A_06_P2084	YDR096W
A_06_P2085	YDR097C
A_06_P2086	YDR098C
A_06_P2087	YDR099W
A_06_P2088	YDR100W
A_06_P2089	YDR101C
A_06_P2090	YDR102C
A_06_P2091	YDR103W
A_06_P2092	YDR104C
A_06_P2093	YDR105C
A_06_P2094	YDR106W
A_06_P2095	YDR107C
A_06_P2096	YDR108W
A_06_P2097	YDR109C
A_06_P2098	YDR110W

A_06_P2099	YDR111C
A_06_P2100	YDR112W
A_06_P2101	YDR113C
A_06_P2102	YDR114C
A_06_P2103	YDR115W
A_06_P2104	YDR116C
A_06_P2105	YDR117C
A_06_P2106	YDR118W
A_06_P2107	YDR119W
A_06_P2108	YDR120C
A_06_P2109	YDR121W
A_06_P2110	YDR122W
A_06_P2111	YDR123C
A_06_P2112	YDR124W
A_06_P2113	YDR125C
A_06_P2114	YDR126W
A_06_P2115	YDR127W
A_06_P2116	YDR128W
A_06_P2117	YDR129C
A_06_P2118	YDR130C
A_06_P2119	YDR131C
A_06_P2120	YDR132C
A_06_P2121	YDR133C
A_06_P2122	YDR134C
A_06_P2123	YDR135C
A_06_P2124	YDR136C
A_06_P2125	YDR137W
A_06_P2126	YDR138W
A_06_P2127	YDR139C
A_06_P2128	YDR140W
A_06_P2129	YDR141C
A_06_P2130	YDR142C
A_06_P2131	YDR143C
A_06_P2132	YDR144C
A_06_P2133	YDR145W
A_06_P2134	YDR146C
A_06_P2135	YDR147W
A_06_P2136	YDR148C
A_06_P2137	YDR149C
A_06_P2138	YDR150W
A_06_P2139	YDR151C
A_06_P2140	YDR152W
A_06_P2141	YDR153C
A_06_P2142	YDR154C
A_06_P2143	YDR155C
A_06_P2144	YDR156W
A_06_P2145	YDR157W
A_06_P2146	YDR158W
A_06_P2147	YDR159W
A_06_P2148	YDR160W
A_06_P2149	YDR161W
A_06_P2150	YDR162C
A_06_P2151	YDR163W
A_06_P2152	YDR164C
A_06_P2153	YDR165W

A_06_P2154	YDR166C
A_06_P2155	YDR167W
A_06_P2156	YDR168W
A_06_P2157	YDR169C
A_06_P2158	YDR170C
A_06_P2159	YDR171W
A_06_P2160	YDR172W
A_06_P2161	YDR173C
A_06_P2162	YDR174W
A_06_P2163	YDR175C
A_06_P2164	YDR176W
A_06_P2165	YDR177W
A_06_P2166	YDR178W
A_06_P2167	YDR179C
A_06_P2168	YDR179W-A
A_06_P2169	YDR180W
A_06_P2170	YDR181C
A_06_P2171	YDR182W
A_06_P2172	YDR183W
A_06_P2173	YDR184C
A_06_P2174	YDR185C
A_06_P2175	YDR186C
A_06_P2176	YDR187C
A_06_P2177	YDR188W
A_06_P2178	YDR189W
A_06_P2179	YDR190C
A_06_P2180	YDR191W
A_06_P2181	YDR192C
A_06_P2182	YDR193W
A_06_P2183	YDR194C
A_06_P2184	YDR195W
A_06_P2185	YDR196C
A_06_P2186	YDR197W
A_06_P2187	YDR198C
A_06_P2188	YDR199W
A_06_P2189	YDR200C
A_06_P2190	YDR201W
A_06_P2191	YDR202C
A_06_P2192	YDR203W
A_06_P2193	YDR204W
A_06_P2194	YDR205W
A_06_P2195	YDR206W
A_06_P2196	YDR207C
A_06_P2197	YDR208W
A_06_P2198	YDR209C
A_06_P2199	YDR210W
A_06_P2200	YDR211W
A_06_P2201	YDR212W
A_06_P2202	YDR213W
A_06_P2203	YDR214W
A_06_P2204	YDR215C
A_06_P2205	YDR216W
A_06_P2206	YDR217C
A_06_P2207	YDR218C
A_06_P2208	YDR219C

A_06_P2209	YDR220C
A_06_P2210	YDR221W
A_06_P2211	YDR222W
A_06_P2212	YDR223W
A_06_P2213	YDR224C
A_06_P2214	YDR225W
A_06_P2215	YDR226W
A_06_P2216	YDR227W
A_06_P2217	YDR228C
A_06_P2218	YDR229W
A_06_P2219	YDR230W
A_06_P2220	YDR231C
A_06_P2221	YDR232W
A_06_P2222	YDR233C
A_06_P2223	YDR234W
A_06_P2224	YDR235W
A_06_P2225	YDR236C
A_06_P2226	YDR237W
A_06_P2227	YDR238C
A_06_P2228	YDR239C
A_06_P2229	YDR240C
A_06_P2230	YDR241W
A_06_P2231	YDR242W
A_06_P2232	YDR243C
A_06_P2233	YDR244W
A_06_P2234	YDR245W
A_06_P2235	YDR246W
A_06_P2236	YDR247W
A_06_P2237	YDR248C
A_06_P2238	YDR249C
A_06_P2239	YDR250C
A_06_P2240	YDR251W
A_06_P2241	YDR252W
A_06_P2242	YDR253C
A_06_P2243	YDR254W
A_06_P2244	YDR255C
A_06_P2245	YDR256C
A_06_P2246	YDR257C
A_06_P2247	YDR258C
A_06_P2248	YDR259C
A_06_P2249	YDR260C
A_06_P2250	YDR261C
A_06_P2251	YDR262W
A_06_P2252	YDR263C
A_06_P2253	YDR264C
A_06_P2254	YDR265W
A_06_P2255	YDR266C
A_06_P2256	YDR267C
A_06_P2257	YDR268W
A_06_P2258	YDR269C
A_06_P2259	YDR270W
A_06_P2260	YDR271C
A_06_P2261	YDR272W
A_06_P2262	YDR273W
A_06_P2263	YDR274C

A_06_P2264	YDR275W
A_06_P2265	YDR276C
A_06_P2266	YDR277C
A_06_P2267	YDR278C
A_06_P2268	YDR279W
A_06_P2269	YDR280W
A_06_P2270	YDR281C
A_06_P2271	YDR282C
A_06_P2272	YDR283C
A_06_P2273	YDR284C
A_06_P2274	YDR285W
A_06_P2275	YDR286C
A_06_P2276	YDR287W
A_06_P2277	YDR288W
A_06_P2278	YDR289C
A_06_P2279	YDR290W
A_06_P2280	YDR291W
A_06_P2281	YDR292C
A_06_P2282	YDR293C
A_06_P2283	YDR294C
A_06_P2284	YDR295C
A_06_P2285	YDR296W
A_06_P2286	YDR297W
A_06_P2287	YDR298C
A_06_P2288	YDR299W
A_06_P2289	YDR300C
A_06_P2290	YDR301W
A_06_P2291	YDR302W
A_06_P2292	YDR303C
A_06_P2293	YDR304C
A_06_P2294	YDR305C
A_06_P2295	YDR306C
A_06_P2296	YDR307W
A_06_P2297	YDR308C
A_06_P2298	YDR309C
A_06_P2299	YDR310C
A_06_P2300	YDR311W
A_06_P2301	YDR312W
A_06_P2302	YDR313C
A_06_P2303	YDR314C
A_06_P2304	YDR315C
A_06_P2305	YDR316W
A_06_P2306	YDR317W
A_06_P2307	YDR318W
A_06_P2308	YDR319C
A_06_P2309	YDR320C
A_06_P2310	YDR320C-A
A_06_P2311	YDR321W
A_06_P2312	YDR322C-A
A_06_P2313	YDR322W
A_06_P2314	YDR323C
A_06_P2315	YDR324C
A_06_P2316	YDR325W
A_06_P2317	YDR326C
A_06_P2318	YDR327W

A_06_P2319	YDR328C
A_06_P2320	YDR329C
A_06_P2321	YDR330W
A_06_P2322	YDR331W
A_06_P2323	YDR332W
A_06_P2324	YDR333C
A_06_P2325	YDR334W
A_06_P2326	YDR335W
A_06_P2327	YDR336W
A_06_P2328	YDR337W
A_06_P2329	YDR338C
A_06_P2330	YDR339C
A_06_P2331	YDR340W
A_06_P2332	YDR341C
A_06_P2333	YDR342C
A_06_P2334	YDR343C
A_06_P2335	YDR344C
A_06_P2336	YDR345C
A_06_P2337	YDR346C
A_06_P2338	YDR347W
A_06_P2339	YDR348C
A_06_P2340	YDR349C
A_06_P2341	YDR350C
A_06_P2342	YDR351W
A_06_P2343	YDR352W
A_06_P2344	YDR353W
A_06_P2345	YDR354W
A_06_P2346	YDR355C
A_06_P2347	YDR356W
A_06_P2348	YDR357C
A_06_P2349	YDR358W
A_06_P2350	YDR359C
A_06_P2351	YDR360W
A_06_P2352	YDR361C
A_06_P2353	YDR362C
A_06_P2354	YDR363W
A_06_P2355	YDR363W-A
A_06_P2356	YDR364C
A_06_P2357	YDR365C
A_06_P2358	YDR366C
A_06_P2359	YDR367W
A_06_P2360	YDR368W
A_06_P2361	YDR369C
A_06_P2362	YDR370C
A_06_P2363	YDR371W
A_06_P2364	YDR372C
A_06_P2365	YDR373W
A_06_P2366	YDR374C
A_06_P2367	YDR375C
A_06_P2368	YDR376W
A_06_P2369	YDR377W
A_06_P2370	YDR378C
A_06_P2371	YDR379C-A
A_06_P2372	YDR379W
A_06_P2373	YDR380W

A_06_P7255	YDR381C-A
A_06_P2374	YDR381W
A_06_P2375	YDR382W
A_06_P2376	YDR383C
A_06_P2377	YDR384C
A_06_P2378	YDR385W
A_06_P2379	YDR386W
A_06_P2380	YDR387C
A_06_P2381	YDR388W
A_06_P2382	YDR389W
A_06_P2383	YDR390C
A_06_P2384	YDR391C
A_06_P2385	YDR392W
A_06_P2386	YDR393W
A_06_P2387	YDR394W
A_06_P2388	YDR395W
A_06_P2389	YDR396W
A_06_P2390	YDR397C
A_06_P2391	YDR398W
A_06_P2392	YDR399W
A_06_P2393	YDR400W
A_06_P2394	YDR401W
A_06_P2395	YDR402C
A_06_P2396	YDR403W
A_06_P2397	YDR404C
A_06_P2398	YDR405W
A_06_P2399	YDR406W
A_06_P2400	YDR407C
A_06_P2401	YDR408C
A_06_P2402	YDR409W
A_06_P2403	YDR410C
A_06_P2404	YDR411C
A_06_P2405	YDR412W
A_06_P2406	YDR413C
A_06_P2407	YDR414C
A_06_P2408	YDR415C
A_06_P2409	YDR416W
A_06_P2410	YDR417C
A_06_P2411	YDR418W
A_06_P2412	YDR419W
A_06_P2413	YDR420W
A_06_P2414	YDR421W
A_06_P2415	YDR422C
A_06_P2416	YDR423C
A_06_P2417	YDR424C
A_06_P2418	YDR425W
A_06_P2419	YDR426C
A_06_P2420	YDR427W
A_06_P2421	YDR428C
A_06_P2422	YDR429C
A_06_P2423	YDR430C
A_06_P2424	YDR431W
A_06_P2425	YDR432W
A_06_P2426	YDR433W
A_06_P2427	YDR434W

A_06_P2428	YDR435C
A_06_P2429	YDR436W
A_06_P2430	YDR437W
A_06_P2431	YDR438W
A_06_P2432	YDR439W
A_06_P2433	YDR440W
A_06_P2434	YDR441C
A_06_P2435	YDR442W
A_06_P2436	YDR443C
A_06_P2437	YDR444W
A_06_P2438	YDR445C
A_06_P2439	YDR446W
A_06_P2440	YDR447C
A_06_P2441	YDR448W
A_06_P2442	YDR449C
A_06_P2443	YDR450W
A_06_P2444	YDR451C
A_06_P2445	YDR452W
A_06_P2446	YDR453C
A_06_P2447	YDR454C
A_06_P2448	YDR455C
A_06_P2449	YDR456W
A_06_P2450	YDR457W
A_06_P2451	YDR458C
A_06_P2452	YDR459C
A_06_P2453	YDR460W
A_06_P2454	YDR461W
A_06_P2455	YDR462W
A_06_P2456	YDR463W
A_06_P2457	YDR464W
A_06_P2458	YDR465C
A_06_P2459	YDR466W
A_06_P2460	YDR467C
A_06_P2461	YDR468C
A_06_P2462	YDR469W
A_06_P2463	YDR470C
A_06_P2464	YDR471W
A_06_P2465	YDR472W
A_06_P2466	YDR473C
A_06_P2467	YDR475C
A_06_P2468	YDR475C
A_06_P2469	YDR476C
A_06_P2470	YDR477W
A_06_P2471	YDR478W
A_06_P2472	YDR479C
A_06_P2473	YDR480W
A_06_P2474	YDR481C
A_06_P2475	YDR482C
A_06_P2476	YDR483W
A_06_P2477	YDR484W
A_06_P2478	YDR485C
A_06_P2479	YDR486C
A_06_P2480	YDR487C
A_06_P2481	YDR488C
A_06_P2482	YDR489W

A_06_P2483	YDR490C
A_06_P2484	YDR491C
A_06_P2485	YDR492W
A_06_P2486	YDR493W
A_06_P2487	YDR494W
A_06_P2488	YDR495C
A_06_P2489	YDR496C
A_06_P2490	YDR497C
A_06_P2491	YDR498C
A_06_P2492	YDR499W
A_06_P2493	YDR500C
A_06_P2494	YDR501W
A_06_P2495	YDR502C
A_06_P2496	YDR503C
A_06_P2497	YDR504C
A_06_P2498	YDR505C
A_06_P2499	YDR506C
A_06_P2500	YDR507C
A_06_P2501	YDR508C
A_06_P2502	YDR509W
A_06_P2503	YDR510W
A_06_P2504	YDR511W
A_06_P2505	YDR512C
A_06_P2506	YDR513W
A_06_P2507	YDR514C
A_06_P2508	YDR515W
A_06_P2509	YDR516C
A_06_P2510	YDR517W
A_06_P2511	YDR518W
A_06_P2512	YDR519W
A_06_P2513	YDR520C
A_06_P2514	YDR521W
A_06_P2515	YDR522C
A_06_P2516	YDR523C
A_06_P2517	YDR524C
A_06_P2518	YDR525W
A_06_P2519	YDR525W-A
A_06_P2520	YDR526C
A_06_P2521	YDR527W
A_06_P2522	YDR528W
A_06_P2523	YDR529C
A_06_P2524	YDR530C
A_06_P2525	YDR531W
A_06_P2526	YDR532C
A_06_P2527	YDR533C
A_06_P2528	YDR534C
A_06_P2529	YDR535C
A_06_P2530	YDR536W
A_06_P2531	YDR537C
A_06_P2532	YDR538W
A_06_P2533	YDR539W
A_06_P2534	YDR540C
A_06_P2535	YDR541C
A_06_P2536	YDR542W
A_06_P2537	YDR543C

A_06_P2538	YDR544C
A_06_P2539	YDR545W
A_06_P2540	YEL001C
A_06_P2541	YEL002C
A_06_P2542	YEL003W
A_06_P2543	YEL004W
A_06_P2544	YEL005C
A_06_P2545	YEL006W
A_06_P2546	YEL007W
A_06_P2547	YEL008W
A_06_P2548	YEL009C
A_06_P2549	YEL010W
A_06_P2550	YEL011W
A_06_P2551	YEL012W
A_06_P2552	YEL013W
A_06_P2553	YEL014C
A_06_P2554	YEL015W
A_06_P2555	YEL016C
A_06_P2556	YEL017C-A
A_06_P2557	YEL017W
A_06_P2558	YEL018W
A_06_P2559	YEL019C
A_06_P2560	YEL020C
A_06_P2561	YEL020W-A
A_06_P2562	YEL021W
A_06_P2563	YEL022W
A_06_P2564	YEL023C
A_06_P2565	YEL024W
A_06_P2566	YEL025C
A_06_P2567	YEL026W
A_06_P2568	YEL027W
A_06_P2569	YEL028W
A_06_P2570	YEL029C
A_06_P2571	YEL030W
A_06_P2572	YEL031W
A_06_P2573	YEL032W
A_06_P2574	YEL033W
A_06_P2575	YEL034W
A_06_P2576	YEL035C
A_06_P2577	YEL036C
A_06_P2578	YEL037C
A_06_P2579	YEL038W
A_06_P2580	YEL039C
A_06_P2581	YEL040W
A_06_P2582	YEL041W
A_06_P2583	YEL042W
A_06_P2584	YEL043W
A_06_P2585	YEL044W
A_06_P2586	YEL045C
A_06_P2587	YEL046C
A_06_P2588	YEL047C
A_06_P2589	YEL048C
A_06_P2590	YEL049W
A_06_P2591	YEL050C
A_06_P2592	YEL051W

A_06_P2593	YEL052W
A_06_P2594	YEL053C
A_06_P2595	YEL054C
A_06_P2596	YEL055C
A_06_P2597	YEL056W
A_06_P2598	YEL057C
A_06_P2599	YEL058W
A_06_P2600	YEL059C-A
A_06_P2601	YEL059W
A_06_P2602	YEL060C
A_06_P2603	YEL061C
A_06_P2604	YEL062W
A_06_P2605	YEL063C
A_06_P2606	YEL064C
A_06_P2607	YEL065W
A_06_P2608	YEL066W
A_06_P2609	YEL067C
A_06_P2610	YEL068C
A_06_P2611	YEL069C
A_06_P2612	YEL070W
A_06_P2613	YEL071W
A_06_P2614	YEL072W
A_06_P2615	YEL073C
A_06_P2616	YEL074W
A_06_P2617	YEL075C
A_06_P2620	YEL075W-A
A_06_P2618	YEL076C
A_06_P2619	YEL076C-A
A_06_P2621	YEL077C
A_06_P2622	YER001W
A_06_P2623	YER002W
A_06_P2624	YER003C
A_06_P2625	YER004W
A_06_P2626	YER005W
A_06_P2627	YER006W
A_06_P2628	YER007C-A
A_06_P2629	YER007W
A_06_P2630	YER008C
A_06_P2631	YER009W
A_06_P2632	YER010C
A_06_P2633	YER011W
A_06_P2634	YER012W
A_06_P2635	YER013W
A_06_P2636	YER014C-A
A_06_P2637	YER014W
A_06_P2638	YER015W
A_06_P2639	YER016W
A_06_P2640	YER017C
A_06_P2641	YER018C
A_06_P2642	YER019C-A
A_06_P2643	YER019W
A_06_P2644	YER020W
A_06_P2645	YER021W
A_06_P2646	YER022W
A_06_P2647	YER023W

A_06_P2648	YER024W
A_06_P2649	YER025W
A_06_P2650	YER026C
A_06_P2651	YER027C
A_06_P2652	YER028C
A_06_P2653	YER029C
A_06_P2654	YER030W
A_06_P2655	YER031C
A_06_P2656	YER032W
A_06_P2657	YER033C
A_06_P2658	YER034W
A_06_P2659	YER035W
A_06_P2660	YER036C
A_06_P2661	YER037W
A_06_P2662	YER038C
A_06_P2663	YER039C
A_06_P2664	YER039C-A
A_06_P2665	YER040W
A_06_P2666	YER041W
A_06_P2667	YER042W
A_06_P2668	YER043C
A_06_P2669	YER044C
A_06_P2670	YER044C-A
A_06_P2671	YER045C
A_06_P2672	YER046W
A_06_P2673	YER047C
A_06_P2674	YER048C
A_06_P2675	YER048W-A
A_06_P2676	YER049W
A_06_P2677	YER050C
A_06_P2678	YER051W
A_06_P2679	YER052C
A_06_P2680	YER053C
A_06_P2681	YER053C-A
A_06_P2682	YER054C
A_06_P2683	YER055C
A_06_P2684	YER056C
A_06_P2685	YER056C-A
A_06_P2686	YER057C
A_06_P2687	YER058W
A_06_P2688	YER059W
A_06_P2689	YER060W
A_06_P2690	YER060W-A
A_06_P2691	YER061C
A_06_P2692	YER062C
A_06_P2693	YER063W
A_06_P2694	YER064C
A_06_P2695	YER065C
A_06_P2696	YER066C-A
A_06_P2697	YER066W
A_06_P2698	YER067W
A_06_P2699	YER068W
A_06_P2700	YER069W
A_06_P2701	YER070W
A_06_P2702	YER071C

A_06_P2703	YER072W
A_06_P2704	YER073W
A_06_P2705	YER074W
A_06_P7256	YER074W-A
A_06_P2706	YER075C
A_06_P2707	YER076C
A_06_P2708	YER077C
A_06_P2709	YER078C
A_06_P2710	YER079W
A_06_P2711	YER080W
A_06_P2712	YER081W
A_06_P2713	YER082C
A_06_P2714	YER083C
A_06_P2715	YER084W
A_06_P2716	YER085C
A_06_P2717	YER086W
A_06_P7253	YER087C-B
A_06_P2718	YER087W
A_06_P2719	YER088C
A_06_P2720	YER089C
A_06_P2721	YER090W
A_06_P2722	YER091C
A_06_P2723	YER091C-A
A_06_P2724	YER092W
A_06_P2725	YER093C
A_06_P2726	YER093C-A
A_06_P2727	YER094C
A_06_P2728	YER095W
A_06_P2729	YER096W
A_06_P2730	YER097W
A_06_P2731	YER098W
A_06_P2732	YER099C
A_06_P2733	YER100W
A_06_P2734	YER101C
A_06_P2735	YER102W
A_06_P2736	YER103W
A_06_P2737	YER104W
A_06_P2738	YER105C
A_06_P2739	YER106W
A_06_P2740	YER107C
A_06_P2741	YER109C
A_06_P2742	YER110C
A_06_P2743	YER111C
A_06_P2744	YER112W
A_06_P2745	YER113C
A_06_P2746	YER114C
A_06_P2747	YER115C
A_06_P2748	YER116C
A_06_P2749	YER117W
A_06_P2750	YER118C
A_06_P2751	YER119C
A_06_P2752	YER119C-A
A_06_P2753	YER120W
A_06_P2754	YER121W
A_06_P2755	YER122C

A_06_P2756	YER123W
A_06_P2757	YER124C
A_06_P2758	YER125W
A_06_P2759	YER126C
A_06_P2760	YER127W
A_06_P2761	YER128W
A_06_P2762	YER129W
A_06_P2763	YER130C
A_06_P2764	YER131W
A_06_P2765	YER132C
A_06_P2766	YER133W
A_06_P2767	YER134C
A_06_P2768	YER135C
A_06_P2769	YER136W
A_06_P2770	YER137C
A_06_P2771	YER138W-A
A_06_P2772	YER139C
A_06_P2773	YER140W
A_06_P2774	YER141W
A_06_P2775	YER142C
A_06_P2776	YER143W
A_06_P2777	YER144C
A_06_P2778	YER145C
A_06_P2779	YER146W
A_06_P2780	YER147C
A_06_P2781	YER148W
A_06_P2782	YER149C
A_06_P2783	YER150W
A_06_P2784	YER151C
A_06_P2785	YER152C
A_06_P2786	YER153C
A_06_P2787	YER154W
A_06_P2788	YER155C
A_06_P2789	YER156C
A_06_P2790	YER157W
A_06_P2791	YER158C
A_06_P2792	YER159C
A_06_P2793	YER161C
A_06_P2794	YER162C
A_06_P2795	YER163C
A_06_P2796	YER164W
A_06_P2797	YER165W
A_06_P2798	YER166W
A_06_P2799	YER167W
A_06_P2800	YER168C
A_06_P2801	YER169W
A_06_P2802	YER170W
A_06_P2803	YER171W
A_06_P2804	YER172C
A_06_P2805	YER173W
A_06_P2806	YER174C
A_06_P2807	YER175C
A_06_P2808	YER176W
A_06_P2809	YER177W
A_06_P2810	YER178W

A_06_P2811	YER179W
A_06_P2812	YER180C
A_06_P2813	YER181C
A_06_P2814	YER182W
A_06_P2815	YER183C
A_06_P2816	YER184C
A_06_P2817	YER185W
A_06_P2818	YER186C
A_06_P2819	YER187W
A_06_P2820	YER187W
A_06_P2821	YER188W
A_06_P2822	YER189W
A_06_P2823	YER190W
A_06_P2824	YFL001W
A_06_P2825	YFL002C
A_06_P2826	YFL003C
A_06_P2827	YFL004W
A_06_P2828	YFL005W
A_06_P2829	YFL007W
A_06_P2830	YFL007W
A_06_P2831	YFL008W
A_06_P2832	YFL009W
A_06_P2833	YFL010C
A_06_P2834	YFL010W-A
A_06_P2835	YFL011W
A_06_P2836	YFL012W
A_06_P2838	YFL012W-A
A_06_P2837	YFL013C
A_06_P2839	YFL014W
A_06_P2840	YFL015C
A_06_P2841	YFL016C
A_06_P2842	YFL017C
A_06_P2843	YFL017W-A
A_06_P2844	YFL018C
A_06_P2845	YFL019C
A_06_P2846	YFL020C
A_06_P2847	YFL021W
A_06_P2848	YFL022C
A_06_P2849	YFL023W
A_06_P2850	YFL024C
A_06_P2851	YFL025C
A_06_P2852	YFL026W
A_06_P2853	YFL027C
A_06_P2854	YFL028C
A_06_P2855	YFL029C
A_06_P2856	YFL030W
A_06_P2857	YFL031W
A_06_P2858	YFL032W
A_06_P2859	YFL033C
A_06_P2860	YFL034C-A
A_06_P2861	YFL034C-B
A_06_P2862	YFL034W
A_06_P2863	YFL036W
A_06_P2864	YFL037W
A_06_P2865	YFL038C

A_06_P2866	YFL039C
A_06_P2867	YFL040W
A_06_P2868	YFL041W
A_06_P2869	YFL042C
A_06_P2870	YFL044C
A_06_P2871	YFL045C
A_06_P2872	YFL046W
A_06_P2873	YFL047W
A_06_P2874	YFL048C
A_06_P2875	YFL049W
A_06_P2876	YFL050C
A_06_P2877	YFL051C
A_06_P2878	YFL052W
A_06_P2879	YFL053W
A_06_P2880	YFL054C
A_06_P2881	YFL055W
A_06_P2882	YFL056C
A_06_P2883	YFL057C
A_06_P2884	YFL058W
A_06_P2885	YFL059W
A_06_P2886	YFL060C
A_06_P2887	YFL061W
A_06_P2888	YFL062W
A_06_P2889	YFL063W
A_06_P2890	YFL064C
A_06_P2891	YFL065C
A_06_P2892	YFL066C
A_06_P2893	YFL067W
A_06_P2894	YFL068W
A_06_P2895	YFR001W
A_06_P2896	YFR002W
A_06_P2897	YFR003C
A_06_P2898	YFR004W
A_06_P2899	YFR005C
A_06_P2900	YFR006W
A_06_P2901	YFR007W
A_06_P2902	YFR008W
A_06_P2903	YFR009W
A_06_P2904	YFR010W
A_06_P2905	YFR011C
A_06_P2906	YFR012W
A_06_P2907	YFR012W-A
A_06_P2908	YFR013W
A_06_P2909	YFR014C
A_06_P2910	YFR015C
A_06_P2911	YFR016C
A_06_P2912	YFR017C
A_06_P2913	YFR018C
A_06_P2914	YFR019W
A_06_P2915	YFR020W
A_06_P2916	YFR021W
A_06_P2917	YFR022W
A_06_P2918	YFR023W
A_06_P2919	YFR024C-A
A_06_P2920	YFR024C-A

A_06_P2921	YFR025C
A_06_P2922	YFR026C
A_06_P2923	YFR027W
A_06_P2924	YFR028C
A_06_P2925	YFR029W
A_06_P2926	YFR030W
A_06_P2927	YFR031C
A_06_P2928	YFR031C-A
A_06_P2929	YFR032C
A_06_P2930	YFR032C-A
A_06_P2931	YFR033C
A_06_P2932	YFR034C
A_06_P2933	YFR035C
A_06_P2934	YFR036W
A_06_P2935	YFR037C
A_06_P2936	YFR038W
A_06_P2937	YFR039C
A_06_P2938	YFR040W
A_06_P2939	YFR041C
A_06_P2940	YFR042W
A_06_P2941	YFR043C
A_06_P2942	YFR044C
A_06_P2943	YFR045W
A_06_P2944	YFR046C
A_06_P2945	YFR047C
A_06_P2946	YFR048W
A_06_P2947	YFR049W
A_06_P2948	YFR050C
A_06_P2949	YFR051C
A_06_P2950	YFR052W
A_06_P2951	YFR053C
A_06_P2952	YFR054C
A_06_P2953	YFR055W
A_06_P2954	YFR056C
A_06_P2955	YFR057W
A_06_P2956	YGL001C
A_06_P2957	YGL002W
A_06_P2958	YGL003C
A_06_P2959	YGL004C
A_06_P2960	YGL005C
A_06_P2961	YGL006W
A_06_P2962	YGL007W
A_06_P2963	YGL008C
A_06_P2964	YGL009C
A_06_P2965	YGL010W
A_06_P2966	YGL011C
A_06_P2967	YGL012W
A_06_P2968	YGL013C
A_06_P2969	YGL014W
A_06_P2970	YGL015C
A_06_P2971	YGL016W
A_06_P2972	YGL017W
A_06_P2973	YGL018C
A_06_P2974	YGL019W
A_06_P2975	YGL020C

A_06_P2976	YGL021W
A_06_P2977	YGL022W
A_06_P2978	YGL023C
A_06_P2979	YGL024W
A_06_P2980	YGL025C
A_06_P2981	YGL026C
A_06_P2982	YGL027C
A_06_P2983	YGL028C
A_06_P2984	YGL029W
A_06_P2985	YGL030W
A_06_P2986	YGL031C
A_06_P2987	YGL032C
A_06_P2988	YGL033W
A_06_P2989	YGL034C
A_06_P2990	YGL035C
A_06_P2991	YGL036W
A_06_P2992	YGL037C
A_06_P2993	YGL038C
A_06_P2994	YGL039W
A_06_P2995	YGL040C
A_06_P2996	YGL041C
A_06_P2997	YGL042C
A_06_P2998	YGL043W
A_06_P2999	YGL044C
A_06_P3000	YGL045W
A_06_P3001	YGL045W
A_06_P3002	YGL047W
A_06_P3003	YGL048C
A_06_P3004	YGL049C
A_06_P3005	YGL050W
A_06_P3006	YGL051W
A_06_P3007	YGL052W
A_06_P3008	YGL053W
A_06_P3009	YGL054C
A_06_P3010	YGL055W
A_06_P3011	YGL056C
A_06_P3012	YGL057C
A_06_P3013	YGL058W
A_06_P3014	YGL059W
A_06_P3015	YGL060W
A_06_P3016	YGL061C
A_06_P3017	YGL062W
A_06_P3018	YGL063W
A_06_P3019	YGL064C
A_06_P3020	YGL065C
A_06_P3021	YGL066W
A_06_P3022	YGL067W
A_06_P3023	YGL068W
A_06_P3024	YGL069C
A_06_P3025	YGL070C
A_06_P3026	YGL071W
A_06_P3027	YGL072C
A_06_P3028	YGL073W
A_06_P3029	YGL074C
A_06_P3030	YGL075C

A_06_P3031	YGL076C
A_06_P3032	YGL077C
A_06_P3033	YGL078C
A_06_P3034	YGL079W
A_06_P3035	YGL080W
A_06_P3036	YGL081W
A_06_P3037	YGL082W
A_06_P3038	YGL083W
A_06_P3039	YGL084C
A_06_P3040	YGL085W
A_06_P3041	YGL086W
A_06_P3042	YGL087C
A_06_P3043	YGL088W
A_06_P3044	YGL089C
A_06_P3045	YGL090W
A_06_P3046	YGL091C
A_06_P3047	YGL092W
A_06_P3048	YGL093W
A_06_P3049	YGL094C
A_06_P3050	YGL095C
A_06_P3051	YGL096W
A_06_P3052	YGL097W
A_06_P3053	YGL098W
A_06_P3054	YGL099W
A_06_P3055	YGL100W
A_06_P3056	YGL101W
A_06_P3057	YGL102C
A_06_P3058	YGL103W
A_06_P3059	YGL104C
A_06_P3060	YGL105W
A_06_P3061	YGL106W
A_06_P3062	YGL107C
A_06_P3063	YGL108C
A_06_P3064	YGL109W
A_06_P3065	YGL110C
A_06_P3066	YGL111W
A_06_P3067	YGL112C
A_06_P3068	YGL113W
A_06_P3069	YGL114W
A_06_P3070	YGL115W
A_06_P3071	YGL116W
A_06_P3072	YGL117W
A_06_P3073	YGL118C
A_06_P3074	YGL119W
A_06_P3075	YGL120C
A_06_P3076	YGL121C
A_06_P3077	YGL122C
A_06_P3078	YGL123W
A_06_P3079	YGL124C
A_06_P3080	YGL125W
A_06_P3081	YGL126W
A_06_P3082	YGL127C
A_06_P3083	YGL128C
A_06_P3084	YGL129C
A_06_P3085	YGL130W

A_06_P3086	YGL131C
A_06_P3087	YGL132W
A_06_P3088	YGL133W
A_06_P3089	YGL134W
A_06_P3090	YGL135W
A_06_P3091	YGL136C
A_06_P3092	YGL137W
A_06_P3093	YGL138C
A_06_P3094	YGL139W
A_06_P3095	YGL140C
A_06_P3096	YGL141W
A_06_P3097	YGL142C
A_06_P3098	YGL143C
A_06_P3099	YGL144C
A_06_P3100	YGL145W
A_06_P3101	YGL146C
A_06_P3102	YGL147C
A_06_P3103	YGL148W
A_06_P3104	YGL149W
A_06_P3105	YGL150C
A_06_P3106	YGL151W
A_06_P3107	YGL152C
A_06_P3108	YGL153W
A_06_P3109	YGL154C
A_06_P3110	YGL155W
A_06_P3111	YGL156W
A_06_P3112	YGL157W
A_06_P3113	YGL158W
A_06_P3114	YGL159W
A_06_P3115	YGL160W
A_06_P3116	YGL161C
A_06_P3117	YGL162W
A_06_P3118	YGL163C
A_06_P3119	YGL164C
A_06_P3120	YGL165C
A_06_P3121	YGL166W
A_06_P3122	YGL167C
A_06_P3123	YGL168W
A_06_P3124	YGL169W
A_06_P3125	YGL170C
A_06_P3126	YGL171W
A_06_P3127	YGL172W
A_06_P3128	YGL173C
A_06_P3129	YGL174W
A_06_P3130	YGL175C
A_06_P3131	YGL176C
A_06_P3132	YGL177W
A_06_P3133	YGL178W
A_06_P3134	YGL179C
A_06_P3135	YGL180W
A_06_P3136	YGL181W
A_06_P3137	YGL182C
A_06_P3138	YGL183C
A_06_P3139	YGL184C
A_06_P3140	YGL185C

A_06_P3141	YGL186C
A_06_P3142	YGL187C
A_06_P3143	YGL188C
A_06_P3144	YGL189C
A_06_P3145	YGL190C
A_06_P3146	YGL191W
A_06_P3147	YGL192W
A_06_P3148	YGL193C
A_06_P3149	YGL194C
A_06_P3150	YGL195W
A_06_P3151	YGL196W
A_06_P3152	YGL197W
A_06_P3153	YGL198W
A_06_P3154	YGL199C
A_06_P3155	YGL200C
A_06_P3156	YGL201C
A_06_P3157	YGL202W
A_06_P3158	YGL203C
A_06_P3159	YGL204C
A_06_P3160	YGL205W
A_06_P3161	YGL206C
A_06_P3162	YGL207W
A_06_P3163	YGL208W
A_06_P3164	YGL209W
A_06_P3165	YGL210W
A_06_P3166	YGL211W
A_06_P3167	YGL212W
A_06_P3168	YGL213C
A_06_P3169	YGL214W
A_06_P3170	YGL215W
A_06_P3171	YGL216W
A_06_P3172	YGL217C
A_06_P3173	YGL218W
A_06_P3174	YGL219C
A_06_P3175	YGL220W
A_06_P3176	YGL221C
A_06_P3177	YGL222C
A_06_P3178	YGL223C
A_06_P3179	YGL224C
A_06_P3180	YGL225W
A_06_P3181	YGL226C-A
A_06_P3182	YGL226W
A_06_P3183	YGL227W
A_06_P3184	YGL228W
A_06_P3185	YGL229C
A_06_P3186	YGL230C
A_06_P3187	YGL231C
A_06_P3188	YGL232W
A_06_P3189	YGL233W
A_06_P3190	YGL234W
A_06_P3191	YGL235W
A_06_P3192	YGL236C
A_06_P3193	YGL237C
A_06_P3194	YGL238W
A_06_P3195	YGL239C

A_06_P3196	YGL240W
A_06_P3197	YGL241W
A_06_P3198	YGL242C
A_06_P3199	YGL243W
A_06_P3200	YGL244W
A_06_P3201	YGL245W
A_06_P3202	YGL246C
A_06_P3203	YGL247W
A_06_P3204	YGL248W
A_06_P3205	YGL249W
A_06_P3206	YGL250W
A_06_P3207	YGL251C
A_06_P3208	YGL252C
A_06_P3209	YGL253W
A_06_P3210	YGL254W
A_06_P3211	YGL255W
A_06_P3212	YGL256W
A_06_P3213	YGL257C
A_06_P3214	YGL258W
A_06_P3215	YGL258W-A
A_06_P3216	YGL259W
A_06_P3217	YGL260W
A_06_P3218	YGL261C
A_06_P3219	YGL262W
A_06_P3220	YGL263W
A_06_P3221	YGR001C
A_06_P3222	YGR002C
A_06_P3223	YGR003W
A_06_P3224	YGR004W
A_06_P3225	YGR005C
A_06_P3226	YGR006W
A_06_P3227	YGR007W
A_06_P3228	YGR008C
A_06_P3229	YGR009C
A_06_P3230	YGR010W
A_06_P3231	YGR011W
A_06_P3232	YGR012W
A_06_P3233	YGR013W
A_06_P3234	YGR014W
A_06_P3235	YGR015C
A_06_P3236	YGR016W
A_06_P3237	YGR017W
A_06_P3238	YGR018C
A_06_P3239	YGR019W
A_06_P3240	YGR020C
A_06_P3241	YGR021W
A_06_P3242	YGR022C
A_06_P3243	YGR023W
A_06_P3244	YGR024C
A_06_P3245	YGR025W
A_06_P3246	YGR026W
A_06_P3247	YGR027C
A_06_P3248	YGR028W
A_06_P3249	YGR029W
A_06_P3250	YGR030C

A_06_P3251	YGR031W
A_06_P3252	YGR032W
A_06_P3253	YGR033C
A_06_P3254	YGR034W
A_06_P3255	YGR035C
A_06_P3256	YGR036C
A_06_P3257	YGR037C
A_06_P3258	YGR038W
A_06_P3259	YGR039W
A_06_P3260	YGR040W
A_06_P3261	YGR041W
A_06_P3262	YGR042W
A_06_P3263	YGR043C
A_06_P3264	YGR044C
A_06_P3265	YGR045C
A_06_P3266	YGR046W
A_06_P3267	YGR047C
A_06_P3268	YGR048W
A_06_P3269	YGR049W
A_06_P3270	YGR050C
A_06_P3271	YGR051C
A_06_P3272	YGR052W
A_06_P3273	YGR053C
A_06_P3274	YGR054W
A_06_P3275	YGR055W
A_06_P3276	YGR056W
A_06_P3277	YGR057C
A_06_P3278	YGR058W
A_06_P3279	YGR059W
A_06_P3280	YGR060W
A_06_P3281	YGR061C
A_06_P3282	YGR062C
A_06_P3283	YGR063C
A_06_P3284	YGR064W
A_06_P3285	YGR065C
A_06_P3286	YGR066C
A_06_P3287	YGR067C
A_06_P3288	YGR068C
A_06_P3289	YGR069W
A_06_P3290	YGR070W
A_06_P3291	YGR071C
A_06_P3292	YGR072W
A_06_P3293	YGR073C
A_06_P3294	YGR074W
A_06_P3295	YGR075C
A_06_P3296	YGR076C
A_06_P3297	YGR077C
A_06_P3298	YGR078C
A_06_P3299	YGR079W
A_06_P3300	YGR080W
A_06_P3301	YGR081C
A_06_P3302	YGR082W
A_06_P3303	YGR083C
A_06_P3304	YGR084C
A_06_P3305	YGR085C

A_06_P3306	YGR086C
A_06_P3307	YGR087C
A_06_P3308	YGR088W
A_06_P3309	YGR089W
A_06_P3310	YGR090W
A_06_P3311	YGR091W
A_06_P3312	YGR092W
A_06_P3313	YGR093W
A_06_P3314	YGR094W
A_06_P3315	YGR095C
A_06_P3316	YGR096W
A_06_P3317	YGR097W
A_06_P3318	YGR098C
A_06_P3319	YGR099W
A_06_P3320	YGR100W
A_06_P3321	YGR101W
A_06_P3322	YGR102C
A_06_P3323	YGR103W
A_06_P3324	YGR104C
A_06_P3325	YGR105W
A_06_P3326	YGR106C
A_06_P3327	YGR107W
A_06_P3328	YGR108W
A_06_P3329	YGR109C
A_06_P3330	YGR110W
A_06_P3331	YGR111W
A_06_P3332	YGR112W
A_06_P3333	YGR113W
A_06_P3334	YGR114C
A_06_P3335	YGR115C
A_06_P3336	YGR116W
A_06_P3337	YGR117C
A_06_P3338	YGR118W
A_06_P3339	YGR119C
A_06_P3340	YGR120C
A_06_P3341	YGR121C
A_06_P3342	YGR122C-A
A_06_P3343	YGR122W
A_06_P3344	YGR123C
A_06_P3345	YGR124W
A_06_P3346	YGR125W
A_06_P3347	YGR126W
A_06_P3348	YGR127W
A_06_P3349	YGR128C
A_06_P3350	YGR129W
A_06_P3351	YGR130C
A_06_P3352	YGR131W
A_06_P3353	YGR132C
A_06_P3354	YGR133W
A_06_P3355	YGR134W
A_06_P3356	YGR135W
A_06_P3357	YGR136W
A_06_P3358	YGR137W
A_06_P3359	YGR138C
A_06_P3360	YGR139W

A_06_P3361	YGR140W
A_06_P3362	YGR141W
A_06_P3363	YGR142W
A_06_P3364	YGR143W
A_06_P3365	YGR144W
A_06_P3366	YGR145W
A_06_P3367	YGR146C
A_06_P3368	YGR147C
A_06_P3369	YGR148C
A_06_P3370	YGR149W
A_06_P3371	YGR150C
A_06_P3372	YGR151C
A_06_P3373	YGR152C
A_06_P3374	YGR153W
A_06_P3375	YGR154C
A_06_P3376	YGR155W
A_06_P3377	YGR156W
A_06_P3378	YGR157W
A_06_P3379	YGR158C
A_06_P3380	YGR159C
A_06_P3381	YGR160W
A_06_P3382	YGR161C
A_06_P3383	YGR162W
A_06_P3384	YGR163W
A_06_P3385	YGR164W
A_06_P3386	YGR165W
A_06_P3387	YGR166W
A_06_P3388	YGR167W
A_06_P3389	YGR168C
A_06_P3390	YGR169C
A_06_P3391	YGR170W
A_06_P3392	YGR171C
A_06_P3393	YGR172C
A_06_P3394	YGR173W
A_06_P3395	YGR174C
A_06_P3396	YGR175C
A_06_P3397	YGR176W
A_06_P3398	YGR177C
A_06_P3399	YGR178C
A_06_P3400	YGR179C
A_06_P3401	YGR180C
A_06_P3402	YGR181W
A_06_P3403	YGR182C
A_06_P3404	YGR183C
A_06_P3405	YGR184C
A_06_P3406	YGR185C
A_06_P3407	YGR186W
A_06_P3408	YGR187C
A_06_P3409	YGR188C
A_06_P3410	YGR189C
A_06_P3411	YGR190C
A_06_P3412	YGR191W
A_06_P3413	YGR192C
A_06_P3414	YGR193C
A_06_P3415	YGR194C

A_06_P3416	YGR195W
A_06_P3417	YGR196C
A_06_P3418	YGR197C
A_06_P3419	YGR198W
A_06_P3420	YGR199W
A_06_P3421	YGR200C
A_06_P3422	YGR201C
A_06_P3423	YGR202C
A_06_P3424	YGR203W
A_06_P3425	YGR204W
A_06_P3426	YGR205W
A_06_P3427	YGR206W
A_06_P3428	YGR207C
A_06_P3429	YGR208W
A_06_P3430	YGR209C
A_06_P3431	YGR210C
A_06_P3432	YGR211W
A_06_P3433	YGR212W
A_06_P3434	YGR213C
A_06_P3435	YGR214W
A_06_P3436	YGR215W
A_06_P3437	YGR216C
A_06_P3438	YGR217W
A_06_P3439	YGR218W
A_06_P3440	YGR219W
A_06_P3441	YGR220C
A_06_P3442	YGR221C
A_06_P3443	YGR222W
A_06_P3444	YGR223C
A_06_P3445	YGR224W
A_06_P3446	YGR225W
A_06_P3447	YGR226C
A_06_P3448	YGR227W
A_06_P3449	YGR228W
A_06_P3450	YGR229C
A_06_P3451	YGR230W
A_06_P3452	YGR231C
A_06_P3453	YGR232W
A_06_P3454	YGR233C
A_06_P3455	YGR234W
A_06_P3456	YGR235C
A_06_P3457	YGR236C
A_06_P3458	YGR237C
A_06_P3459	YGR238C
A_06_P3460	YGR239C
A_06_P3461	YGR240C
A_06_P3462	YGR241C
A_06_P3463	YGR242W
A_06_P3464	YGR243W
A_06_P3465	YGR244C
A_06_P3466	YGR245C
A_06_P3467	YGR246C
A_06_P3468	YGR247W
A_06_P3469	YGR248W
A_06_P3470	YGR249W

A_06_P3471	YGR250C
A_06_P3472	YGR251W
A_06_P3473	YGR252W
A_06_P3474	YGR253C
A_06_P3475	YGR254W
A_06_P3476	YGR255C
A_06_P3477	YGR256W
A_06_P3478	YGR257C
A_06_P3479	YGR258C
A_06_P3480	YGR259C
A_06_P3481	YGR260W
A_06_P3482	YGR261C
A_06_P3483	YGR262C
A_06_P3484	YGR263C
A_06_P3485	YGR264C
A_06_P3486	YGR265W
A_06_P3487	YGR266W
A_06_P3488	YGR267C
A_06_P3489	YGR268C
A_06_P3490	YGR269W
A_06_P3491	YGR270W
A_06_P3492	YGR271C-A
A_06_P3493	YGR271W
A_06_P3494	YGR271C-A
A_06_P3495	YGR273C
A_06_P3496	YGR274C
A_06_P3497	YGR275W
A_06_P3498	YGR276C
A_06_P3499	YGR277C
A_06_P3500	YGR278W
A_06_P3501	YGR279C
A_06_P3502	YGR280C
A_06_P3503	YGR281W
A_06_P3504	YGR282C
A_06_P3505	YGR283C
A_06_P3506	YGR284C
A_06_P3507	YGR285C
A_06_P3508	YGR286C
A_06_P3509	YGR287C
A_06_P3510	YGR288W
A_06_P3511	YGR289C
A_06_P3512	YGR290W
A_06_P3513	YGR291C
A_06_P3514	YGR292W
A_06_P3515	YGR293C
A_06_P3516	YGR294W
A_06_P3517	YGR295C
A_06_P3518	YGR296W
A_06_P3519	YHL001W
A_06_P3520	YHL002W
A_06_P3521	YHL003C
A_06_P3522	YHL004W
A_06_P3523	YHL005C
A_06_P3524	YHL006C
A_06_P3525	YHL007C

A_06_P3526	YHL008C
A_06_P3527	YHL009C
A_06_P3528	YHL010C
A_06_P3529	YHL011C
A_06_P3530	YHL012W
A_06_P3531	YHL013C
A_06_P3532	YHL014C
A_06_P3533	YHL015W
A_06_P3534	YHL016C
A_06_P3535	YHL017W
A_06_P3536	YHL018W
A_06_P3537	YHL019C
A_06_P3538	YHL020C
A_06_P3539	YHL021C
A_06_P3540	YHL022C
A_06_P3541	YHL023C
A_06_P3542	YHL024W
A_06_P3543	YHL025W
A_06_P3544	YHL026C
A_06_P3545	YHL027W
A_06_P3546	YHL028W
A_06_P3547	YHL029C
A_06_P3548	YHL030W
A_06_P3549	YHL031C
A_06_P3550	YHL032C
A_06_P3551	YHL033C
A_06_P3552	YHL034C
A_06_P3553	YHL035C
A_06_P3554	YHL036W
A_06_P3555	YHL037C
A_06_P3556	YHL038C
A_06_P3557	YHL039W
A_06_P3558	YHL040C
A_06_P3559	YHL041W
A_06_P3560	YHL042W
A_06_P3561	YHL043W
A_06_P3562	YHL044W
A_06_P3563	YHL045W
A_06_P3564	YHL046C
A_06_P3565	YHL047C
A_06_P3566	YHL048W
A_06_P3567	YHL049C
A_06_P3568	YHL050C
A_06_P3569	YHR001W
A_06_P3570	YHR001W-A
A_06_P3571	YHR002W
A_06_P3572	YHR003C
A_06_P3573	YHR004C
A_06_P3574	YHR005C
A_06_P3575	YHR005C-A
A_06_P3576	YHR006W
A_06_P3577	YHR007C
A_06_P3578	YHR008C
A_06_P3579	YHR009C
A_06_P3580	YHR010W

A_06_P3581	YHR011W
A_06_P3582	YHR012W
A_06_P3583	YHR013C
A_06_P3584	YHR014W
A_06_P3585	YHR015W
A_06_P3586	YHR016C
A_06_P3587	YHR017W
A_06_P3588	YHR018C
A_06_P3589	YHR019C
A_06_P3590	YHR020W
A_06_P3591	YHR021C
A_06_P3592	YHR021W-A
A_06_P3593	YHR022C
A_06_P3594	YHR023W
A_06_P3595	YHR024C
A_06_P3596	YHR025W
A_06_P3597	YHR026W
A_06_P3598	YHR027C
A_06_P3599	YHR028C
A_06_P3600	YHR029C
A_06_P3601	YHR030C
A_06_P3602	YHR031C
A_06_P3603	YHR032W
A_06_P3604	YHR033W
A_06_P3605	YHR034C
A_06_P3606	YHR035W
A_06_P3607	YHR036W
A_06_P3608	YHR037W
A_06_P3609	YHR038W
A_06_P3610	YHR039C
A_06_P3611	YHR039C-A
A_06_P3612	YHR040W
A_06_P3613	YHR041C
A_06_P3614	YHR042W
A_06_P3615	YHR043C
A_06_P3616	YHR044C
A_06_P3617	YHR045W
A_06_P3618	YHR046C
A_06_P3619	YHR047C
A_06_P3620	YHR048W
A_06_P3621	YHR049C-A
A_06_P3622	YHR049W
A_06_P3623	YHR050W
A_06_P3624	YHR051W
A_06_P3625	YHR052W
A_06_P3626	YHR053C
A_06_P3627	YHR054C
A_06_P3628	YHR055C
A_06_P3629	YHR056C
A_06_P3630	YHR057C
A_06_P3631	YHR058C
A_06_P3632	YHR059W
A_06_P3633	YHR060W
A_06_P3634	YHR061C
A_06_P3635	YHR062C

A_06_P3636	YHR063C
A_06_P3637	YHR064C
A_06_P3638	YHR065C
A_06_P3639	YHR066W
A_06_P3640	YHR067W
A_06_P3641	YHR068W
A_06_P3642	YHR069C
A_06_P3643	YHR070W
A_06_P3644	YHR071W
A_06_P3645	YHR072W
A_06_P3646	YHR072W-A
A_06_P3647	YHR073W
A_06_P3648	YHR074W
A_06_P3649	YHR075C
A_06_P3650	YHR076W
A_06_P3651	YHR077C
A_06_P3652	YHR078W
A_06_P3653	YHR079C
A_06_P3654	YHR079C-A
A_06_P3655	YHR080C
A_06_P3656	YHR081W
A_06_P3657	YHR082C
A_06_P3658	YHR083W
A_06_P3659	YHR084W
A_06_P3660	YHR085W
A_06_P3661	YHR086W
A_06_P3662	YHR087W
A_06_P3663	YHR088W
A_06_P3664	YHR089C
A_06_P3665	YHR090C
A_06_P3666	YHR091C
A_06_P3667	YHR092C
A_06_P3668	YHR093W
A_06_P3669	YHR094C
A_06_P3670	YHR095W
A_06_P3671	YHR096C
A_06_P3672	YHR097C
A_06_P3673	YHR098C
A_06_P3674	YHR099W
A_06_P3675	YHR100C
A_06_P3676	YHR101C
A_06_P3677	YHR102W
A_06_P3678	YHR103W
A_06_P3679	YHR104W
A_06_P3680	YHR105W
A_06_P3681	YHR106W
A_06_P3682	YHR107C
A_06_P3683	YHR108W
A_06_P3684	YHR109W
A_06_P3685	YHR110W
A_06_P3686	YHR111W
A_06_P3687	YHR112C
A_06_P3688	YHR113W
A_06_P3689	YHR114W
A_06_P3690	YHR115C

A_06_P3691	YHR116W
A_06_P3692	YHR117W
A_06_P3693	YHR118C
A_06_P3694	YHR119W
A_06_P3695	YHR120W
A_06_P3696	YHR121W
A_06_P3697	YHR122W
A_06_P3698	YHR123W
A_06_P3699	YHR124W
A_06_P3700	YHR125W
A_06_P3701	YHR126C
A_06_P3702	YHR127W
A_06_P3703	YHR128W
A_06_P3704	YHR129C
A_06_P3705	YHR130C
A_06_P3706	YHR131C
A_06_P3707	YHR132C
A_06_P3708	YHR132W-A
A_06_P3709	YHR133C
A_06_P3710	YHR134W
A_06_P3711	YHR135C
A_06_P3712	YHR136C
A_06_P3713	YHR137W
A_06_P3714	YHR138C
A_06_P3715	YHR139C
A_06_P3716	YHR139C-A
A_06_P3717	YHR140W
A_06_P3718	YHR141C
A_06_P3719	YHR142W
A_06_P3720	YHR143W
A_06_P3721	YHR143W-A
A_06_P3722	YHR144C
A_06_P3723	YHR145C
A_06_P3724	YHR146W
A_06_P3725	YHR147C
A_06_P3726	YHR148W
A_06_P3727	YHR149C
A_06_P3728	YHR150W
A_06_P3729	YHR151C
A_06_P3730	YHR152W
A_06_P3731	YHR153C
A_06_P3732	YHR154W
A_06_P3733	YHR155W
A_06_P3734	YHR156C
A_06_P3735	YHR157W
A_06_P3736	YHR158C
A_06_P3737	YHR159W
A_06_P3738	YHR160C
A_06_P3739	YHR161C
A_06_P3740	YHR162W
A_06_P3741	YHR163W
A_06_P3742	YHR164C
A_06_P3743	YHR165C
A_06_P3744	YHR166C
A_06_P3745	YHR167W

A_06_P3746	YHR168W
A_06_P3747	YHR169W
A_06_P3748	YHR170W
A_06_P3749	YHR171W
A_06_P3750	YHR172W
A_06_P3751	YHR173C
A_06_P3752	YHR174W
A_06_P3753	YHR175W
A_06_P3754	YHR176W
A_06_P3755	YHR177W
A_06_P3756	YHR178W
A_06_P3757	YHR179W
A_06_P3758	YHR180W
A_06_P3759	YHR181W
A_06_P3760	YHR182W
A_06_P3761	YHR183W
A_06_P3762	YHR184W
A_06_P3763	YHR185C
A_06_P3764	YHR186C
A_06_P3765	YHR187W
A_06_P3766	YHR188C
A_06_P3767	YHR189W
A_06_P3768	YHR190W
A_06_P3769	YHR191C
A_06_P3770	YHR192W
A_06_P3771	YHR193C
A_06_P3772	YHR194W
A_06_P3773	YHR195W
A_06_P3774	YHR196W
A_06_P3775	YHR197W
A_06_P3776	YHR198C
A_06_P3777	YHR199C
A_06_P3778	YHR200W
A_06_P3779	YHR201C
A_06_P3780	YHR202W
A_06_P3781	YHR203C
A_06_P3782	YHR204W
A_06_P3783	YHR205W
A_06_P3784	YHR206W
A_06_P3785	YHR207C
A_06_P3786	YHR208W
A_06_P3787	YHR209W
A_06_P3788	YHR210C
A_06_P3789	YHR211W
A_06_P3790	YHR212C
A_06_P3791	YHR213W
A_06_P3792	YHR214W
A_06_P3793	YHR214W-A
A_06_P3794	YHR215W
A_06_P3795	YHR216W
A_06_P3796	YHR217C
A_06_P3797	YHR218W
A_06_P3798	YHR219W
A_06_P3799	YIL001W
A_06_P3800	YIL002C

A_06_P3801	YIL003W
A_06_P3802	YIL004C
A_06_P3803	YIL005W
A_06_P3804	YIL006W
A_06_P3805	YIL007C
A_06_P3806	YIL008W
A_06_P3807	YIL009C-A
A_06_P3808	YIL009W
A_06_P3809	YIL010W
A_06_P3810	YIL011W
A_06_P3811	YIL012W
A_06_P3812	YIL013C
A_06_P3814	YIL014C-A
A_06_P3813	YIL014W
A_06_P3815	YIL015W
A_06_P3816	YIL016W
A_06_P3817	YIL017C
A_06_P3818	YIL018W
A_06_P3819	YIL019W
A_06_P3820	YIL020C
A_06_P3821	YIL021W
A_06_P3822	YIL022W
A_06_P3823	YIL023C
A_06_P3824	YIL024C
A_06_P3825	YIL025C
A_06_P3826	YIL026C
A_06_P3827	YIL027C
A_06_P3828	YIL028W
A_06_P3829	YIL029C
A_06_P3830	YIL030C
A_06_P3831	YIL031W
A_06_P3832	YIL032C
A_06_P3833	YIL033C
A_06_P3834	YIL034C
A_06_P3835	YIL035C
A_06_P3836	YIL036W
A_06_P3837	YIL037C
A_06_P3838	YIL038C
A_06_P3839	YIL039W
A_06_P3840	YIL040W
A_06_P3841	YIL041W
A_06_P3842	YIL042C
A_06_P3843	YIL043C
A_06_P3844	YIL044C
A_06_P3845	YIL045W
A_06_P3846	YIL046W
A_06_P3847	YIL047C
A_06_P3848	YIL048W
A_06_P3849	YIL049W
A_06_P3850	YIL050W
A_06_P3851	YIL051C
A_06_P3852	YIL052C
A_06_P3853	YIL053W
A_06_P3854	YIL054W
A_06_P3855	YIL055C

A_06_P3856	YIL056W
A_06_P3857	YIL057C
A_06_P3858	YIL058W
A_06_P3859	YIL059C
A_06_P3860	YIL060W
A_06_P3861	YIL061C
A_06_P3862	YIL062C
A_06_P3863	YIL063C
A_06_P3864	YIL064W
A_06_P3865	YIL065C
A_06_P3866	YIL066C
A_06_P3867	YIL067C
A_06_P3868	YIL068C
A_06_P3869	YIL069C
A_06_P3870	YIL070C
A_06_P3871	YIL071C
A_06_P3872	YIL072W
A_06_P3873	YIL073C
A_06_P3874	YIL074C
A_06_P3875	YIL075C
A_06_P3876	YIL076W
A_06_P3877	YIL077C
A_06_P3878	YIL078W
A_06_P3879	YIL079C
A_06_P3880	YIL082W
A_06_P3881	YIL082W-A
A_06_P3882	YIL083C
A_06_P3883	YIL084C
A_06_P3884	YIL085C
A_06_P3885	YIL086C
A_06_P3886	YIL087C
A_06_P3887	YIL088C
A_06_P3888	YIL089W
A_06_P3889	YIL090W
A_06_P3890	YIL091C
A_06_P3891	YIL092W
A_06_P3892	YIL093C
A_06_P3893	YIL094C
A_06_P3894	YIL095W
A_06_P3895	YIL096C
A_06_P3896	YIL097W
A_06_P3897	YIL098C
A_06_P3898	YIL099W
A_06_P3899	YIL100W
A_06_P3900	YIL101C
A_06_P3901	YIL102C
A_06_P3902	YIL103W
A_06_P3903	YIL104C
A_06_P3904	YIL105C
A_06_P3905	YIL106W
A_06_P3906	YIL107C
A_06_P3907	YIL108W
A_06_P3908	YIL109C
A_06_P3909	YIL110W
A_06_P3910	YIL111W

A_06_P3911	YIL112W
A_06_P3912	YIL113W
A_06_P3913	YIL114C
A_06_P3914	YIL115C
A_06_P3915	YIL116W
A_06_P3916	YIL117C
A_06_P3917	YIL118W
A_06_P3918	YIL119C
A_06_P3919	YIL120W
A_06_P3920	YIL121W
A_06_P3921	YIL122W
A_06_P3922	YIL123W
A_06_P3923	YIL124W
A_06_P3924	YIL125W
A_06_P3925	YIL126W
A_06_P3926	YIL127C
A_06_P3927	YIL128W
A_06_P3928	YIL129C
A_06_P3929	YIL130W
A_06_P3930	YIL131C
A_06_P3931	YIL132C
A_06_P3932	YIL133C
A_06_P3933	YIL134W
A_06_P3934	YIL135C
A_06_P3935	YIL136W
A_06_P3936	YIL137C
A_06_P3937	YIL138C
A_06_P3938	YIL139C
A_06_P3939	YIL140W
A_06_P3940	YIL141W
A_06_P3941	YIL142W
A_06_P3942	YIL143C
A_06_P3943	YIL144W
A_06_P3944	YIL145C
A_06_P3945	YIL146C
A_06_P3946	YIL147C
A_06_P3947	YIL148W
A_06_P3948	YIL149C
A_06_P3949	YIL150C
A_06_P3950	YIL151C
A_06_P3951	YIL152W
A_06_P3952	YIL153W
A_06_P3953	YIL154C
A_06_P3954	YIL155C
A_06_P3955	YIL156W
A_06_P3956	YIL157C
A_06_P3957	YIL158W
A_06_P3958	YIL159W
A_06_P3959	YIL160C
A_06_P3960	YIL161W
A_06_P3961	YIL162W
A_06_P3962	YIL163C
A_06_P3963	YIL164C
A_06_P3964	YIL165C
A_06_P3965	YIL166C

A_06_P3966	YIL167W
A_06_P3967	YIL168W
A_06_P3968	YIL169C
A_06_P3969	YIL170W
A_06_P3970	YIL171W
A_06_P3971	YIL172C
A_06_P3972	YIL173W
A_06_P3973	YIL174W
A_06_P3974	YIL175W
A_06_P3975	YIL176C
A_06_P3976	YIL177C
A_06_P3977	YIR001C
A_06_P3978	YIR002C
A_06_P3979	YIR003W
A_06_P3980	YIR004W
A_06_P3981	YIR005W
A_06_P3982	YIR006C
A_06_P3983	YIR007W
A_06_P3984	YIR008C
A_06_P3985	YIR009W
A_06_P3986	YIR010W
A_06_P3987	YIR011C
A_06_P3988	YIR012W
A_06_P3989	YIR013C
A_06_P3990	YIR014W
A_06_P3991	YIR015W
A_06_P3992	YIR016W
A_06_P3993	YIR017C
A_06_P3994	YIR018W
A_06_P3995	YIR019C
A_06_P3996	YIR020C
A_06_P3997	YIR020W-A
A_06_P3998	YIR021W
A_06_P3999	YIR022W
A_06_P4000	YIR023W
A_06_P4001	YIR024C
A_06_P4002	YIR025W
A_06_P4003	YIR026C
A_06_P4004	YIR027C
A_06_P4005	YIR028W
A_06_P4006	YIR029W
A_06_P4007	YIR030C
A_06_P4008	YIR031C
A_06_P4009	YIR032C
A_06_P4010	YIR033W
A_06_P4011	YIR034C
A_06_P4012	YIR035C
A_06_P4013	YIR036C
A_06_P4014	YIR037W
A_06_P4015	YIR038C
A_06_P4016	YIR039C
A_06_P4017	YIR040C
A_06_P4018	YIR041W
A_06_P4019	YIR042C
A_06_P4020	YIR043C

A_06_P4021	YIR044C
A_06_P4022	YJL001W
A_06_P4023	YJL002C
A_06_P4024	YJL003W
A_06_P4025	YJL004C
A_06_P4026	YJL005W
A_06_P4027	YJL006C
A_06_P4028	YJL007C
A_06_P4029	YJL008C
A_06_P4030	YJL009W
A_06_P4031	YJL010C
A_06_P4032	YJL011C
A_06_P4033	YJL012C
A_06_P4034	YJL012C
A_06_P4035	YJL013C
A_06_P4036	YJL014W
A_06_P4037	YJL015C
A_06_P4038	YJL016W
A_06_P4039	YJL012C
A_06_P4040	YJL019W
A_06_P4041	YJL019W
A_06_P4042	YJL020C
A_06_P4043	YJL022W
A_06_P4044	YJL023C
A_06_P4045	YJL024C
A_06_P4046	YJL025W
A_06_P4047	YJL026W
A_06_P4048	YJL027C
A_06_P4049	YJL028W
A_06_P4050	YJL029C
A_06_P4051	YJL030W
A_06_P4052	YJL031C
A_06_P4053	YJL032W
A_06_P4054	YJL033W
A_06_P4055	YJL034W
A_06_P4056	YJL035C
A_06_P4057	YJL036W
A_06_P4058	YJL037W
A_06_P4059	YJL038C
A_06_P4060	YJL039C
A_06_P4061	YJL041W
A_06_P4062	YJL042W
A_06_P4063	YJL043W
A_06_P4064	YJL044C
A_06_P4065	YJL045W
A_06_P4066	YJL046W
A_06_P4067	YJL047C
A_06_P4068	YJL048C
A_06_P4069	YJL049W
A_06_P4070	YJL050W
A_06_P4071	YJL051W
A_06_P4072	YJL052C-A
A_06_P4073	YJL052W
A_06_P4074	YJL053W
A_06_P4075	YJL054W

A_06_P4076	YJL055W
A_06_P4077	YJL056C
A_06_P4078	YJL057C
A_06_P4079	YJL058C
A_06_P4080	YJL059W
A_06_P4081	YJL060W
A_06_P4082	YJL061W
A_06_P4083	YJL062W
A_06_P4084	YJL062W-A
A_06_P4085	YJL063C
A_06_P4086	YJL064W
A_06_P4087	YJL065C
A_06_P4088	YJL066C
A_06_P4089	YJL067W
A_06_P4090	YJL068C
A_06_P4091	YJL069C
A_06_P4092	YJL070C
A_06_P4093	YJL071W
A_06_P4094	YJL072C
A_06_P4095	YJL073W
A_06_P4096	YJL074C
A_06_P4097	YJL075C
A_06_P4098	YJL076W
A_06_P4099	YJL077C
A_06_P4100	YJL078C
A_06_P4101	YJL079C
A_06_P4102	YJL080C
A_06_P4103	YJL081C
A_06_P4104	YJL082W
A_06_P4105	YJL083W
A_06_P4106	YJL084C
A_06_P4107	YJL085W
A_06_P4108	YJL086C
A_06_P4109	YJL087C
A_06_P4110	YJL088W
A_06_P4111	YJL089W
A_06_P4112	YJL090C
A_06_P4113	YJL091C
A_06_P4114	YJL092W
A_06_P4115	YJL093C
A_06_P4116	YJL094C
A_06_P4117	YJL095W
A_06_P4118	YJL096W
A_06_P4119	YJL097W
A_06_P4120	YJL098W
A_06_P4121	YJL099W
A_06_P4122	YJL100W
A_06_P4123	YJL101C
A_06_P4124	YJL102W
A_06_P4125	YJL103C
A_06_P4126	YJL104W
A_06_P4127	YJL105W
A_06_P4128	YJL106W
A_06_P4129	YJL107C
A_06_P4130	YJL108C

A_06_P4131	YJL109C
A_06_P4132	YJL110C
A_06_P4133	YJL111W
A_06_P4134	YJL112W
A_06_P4135	YJL115W
A_06_P4136	YJL116C
A_06_P4137	YJL117W
A_06_P4138	YJL118W
A_06_P4139	YJL119C
A_06_P4140	YJL120W
A_06_P4141	YJL121C
A_06_P4142	YJL122W
A_06_P4143	YJL123C
A_06_P4144	YJL124C
A_06_P4145	YJL125C
A_06_P4146	YJL126W
A_06_P4147	YJL127C
A_06_P4148	YJL127W-A
A_06_P4149	YJL128C
A_06_P4150	YJL129C
A_06_P4151	YJL130C
A_06_P4152	YJL131C
A_06_P4153	YJL132W
A_06_P4154	YJL133W
A_06_P4155	YJL134W
A_06_P4156	YJL135W
A_06_P4157	YJL136C
A_06_P4158	YJL137C
A_06_P4159	YJL138C
A_06_P4160	YJL139C
A_06_P4161	YJL140W
A_06_P4162	YJL141C
A_06_P4163	YJL142C
A_06_P4164	YJL143W
A_06_P4165	YJL144W
A_06_P4166	YJL145W
A_06_P4167	YJL146W
A_06_P4168	YJL147C
A_06_P4169	YJL148W
A_06_P4170	YJL149W
A_06_P4171	YJL150W
A_06_P4172	YJL151C
A_06_P4173	YJL152W
A_06_P4174	YJL153C
A_06_P4175	YJL154C
A_06_P4176	YJL155C
A_06_P4177	YJL156C
A_06_P4178	YJL156W-A
A_06_P4179	YJL157C
A_06_P4180	YJL158C
A_06_P4181	YJL159W
A_06_P4182	YJL160C
A_06_P4183	YJL161W
A_06_P4184	YJL162C
A_06_P4185	YJL163C

A_06_P4186	YJL164C
A_06_P4187	YJL165C
A_06_P4188	YJL166W
A_06_P4189	YJL167W
A_06_P4190	YJL168C
A_06_P4191	YJL169W
A_06_P4192	YJL170C
A_06_P4193	YJL171C
A_06_P4194	YJL172W
A_06_P4195	YJL173C
A_06_P4196	YJL174W
A_06_P4197	YJL175W
A_06_P4198	YJL176C
A_06_P4199	YJL177W
A_06_P4200	YJL178C
A_06_P4201	YJL179W
A_06_P4202	YJL180C
A_06_P4203	YJL181W
A_06_P4204	YJL182C
A_06_P4205	YJL183W
A_06_P4206	YJL184W
A_06_P4207	YJL185C
A_06_P4208	YJL186W
A_06_P4209	YJL187C
A_06_P4210	YJL188C
A_06_P4211	YJL189W
A_06_P4212	YJL190C
A_06_P4213	YJL191W
A_06_P4214	YJL192C
A_06_P4215	YJL193W
A_06_P4216	YJL194W
A_06_P4217	YJL195C
A_06_P4218	YJL196C
A_06_P4219	YJL197W
A_06_P4220	YJL198W
A_06_P4221	YJL199C
A_06_P4222	YJL200C
A_06_P4223	YJL201W
A_06_P4224	YJL202C
A_06_P4225	YJL203W
A_06_P4226	YJL204C
A_06_P4227	YJL205C
A_06_P4228	YJL206C
A_06_P4229	YJL207C
A_06_P4230	YJL208C
A_06_P4231	YJL209W
A_06_P4232	YJL210W
A_06_P4233	YJL211C
A_06_P4234	YJL212C
A_06_P4235	YJL213W
A_06_P4236	YJL214W
A_06_P4237	YJL215C
A_06_P4238	YJL216C
A_06_P4239	YJL217W
A_06_P4240	YJL218W

A_06_P4241	YJL219W
A_06_P4242	YJL220W
A_06_P4243	YJL221C
A_06_P4244	YJL222W
A_06_P4245	YJL223C
A_06_P4246	YJL225C
A_06_P4247	YJR001W
A_06_P4248	YJR002W
A_06_P4249	YJR003C
A_06_P4250	YJR004C
A_06_P4251	YJR005W
A_06_P4252	YJR006W
A_06_P4253	YJR007W
A_06_P4254	YJR008W
A_06_P4255	YJR009C
A_06_P4256	YJR010C-A
A_06_P4257	YJR010W
A_06_P4258	YJR011C
A_06_P4259	YJR012C
A_06_P4260	YJR013W
A_06_P4261	YJR014W
A_06_P4262	YJR015W
A_06_P4263	YJR016C
A_06_P4264	YJR017C
A_06_P4265	YJR018W
A_06_P4266	YJR019C
A_06_P4267	YJR020W
A_06_P4268	YJR021C
A_06_P4269	YJR022W
A_06_P4270	YJR023C
A_06_P4271	YJR024C
A_06_P4272	YJR025C
A_06_P4273	YJR030C
A_06_P4274	YJR031C
A_06_P4275	YJR032W
A_06_P4276	YJR033C
A_06_P4277	YJR034W
A_06_P4278	YJR035W
A_06_P4279	YJR036C
A_06_P4280	YJR037W
A_06_P4281	YJR038C
A_06_P4282	YJR039W
A_06_P4283	YJR040W
A_06_P4284	YJR041C
A_06_P4285	YJR042W
A_06_P4286	YJR043C
A_06_P4287	YJR044C
A_06_P4288	YJR045C
A_06_P4289	YJR046W
A_06_P4290	YJR047C
A_06_P4291	YJR048W
A_06_P4292	YJR049C
A_06_P4293	YJR050W
A_06_P4294	YJR051W
A_06_P4295	YJR052W

A_06_P4296	YJR053W
A_06_P4297	YJR054W
A_06_P4298	YJR055W
A_06_P4299	YJR056C
A_06_P4300	YJR057W
A_06_P4301	YJR058C
A_06_P4302	YJR059W
A_06_P4303	YJR060W
A_06_P4304	YJR061W
A_06_P4305	YJR062C
A_06_P4306	YJR063W
A_06_P4307	YJR064W
A_06_P4308	YJR065C
A_06_P4309	YJR066W
A_06_P4310	YJR067C
A_06_P4311	YJR068W
A_06_P4312	YJR069C
A_06_P4313	YJR070C
A_06_P4314	YJR071W
A_06_P4315	YJR072C
A_06_P4316	YJR073C
A_06_P4317	YJR074W
A_06_P4318	YJR075W
A_06_P4319	YJR076C
A_06_P4320	YJR077C
A_06_P4321	YJR078W
A_06_P4322	YJR079W
A_06_P4323	YJR080C
A_06_P4324	YJR082C
A_06_P4325	YJR083C
A_06_P4326	YJR084W
A_06_P4327	YJR085C
A_06_P4328	YJR086W
A_06_P4329	YJR087W
A_06_P4330	YJR088C
A_06_P4331	YJR089W
A_06_P4332	YJR090C
A_06_P4333	YJR091C
A_06_P4334	YJR092W
A_06_P4335	YJR093C
A_06_P4336	YJR094C
A_06_P4337	YJR094W-A
A_06_P4338	YJR095W
A_06_P4339	YJR096W
A_06_P4340	YJR097W
A_06_P4341	YJR098C
A_06_P4342	YJR099W
A_06_P4343	YJR100C
A_06_P4344	YJR101W
A_06_P4345	YJR102C
A_06_P4346	YJR103W
A_06_P4347	YJR104C
A_06_P4348	YJR105W
A_06_P4349	YJR106W
A_06_P4350	YJR107W

A_06_P4351	YJR108W
A_06_P4352	YJR109C
A_06_P4353	YJR110W
A_06_P4354	YJR111C
A_06_P4355	YJR112W
A_06_P4356	YJR113C
A_06_P4357	YJR114W
A_06_P4358	YJR115W
A_06_P4359	YJR116W
A_06_P4360	YJR117W
A_06_P4361	YJR118C
A_06_P4362	YJR119C
A_06_P4363	YJR120W
A_06_P4364	YJR121W
A_06_P4365	YJR122W
A_06_P4366	YJR123W
A_06_P4367	YJR124C
A_06_P4368	YJR125C
A_06_P4369	YJR126C
A_06_P4370	YJR127C
A_06_P4371	YJR128W
A_06_P4372	YJR129C
A_06_P4373	YJR130C
A_06_P4374	YJR131W
A_06_P4375	YJR132W
A_06_P4376	YJR133W
A_06_P4377	YJR134C
A_06_P4378	YJR135C
A_06_P4379	YJR135W-A
A_06_P4380	YJR136C
A_06_P4381	YJR137C
A_06_P4382	YJR138W
A_06_P4383	YJR139C
A_06_P4384	YJR140C
A_06_P4385	YJR141W
A_06_P4386	YJR142W
A_06_P4387	YJR143C
A_06_P4388	YJR144W
A_06_P4389	YJR145C
A_06_P4390	YJR146W
A_06_P4391	YJR147W
A_06_P4392	YJR148W
A_06_P4393	YJR149W
A_06_P4394	YJR150C
A_06_P4395	YJR151C
A_06_P4396	YJR152W
A_06_P4397	YJR153W
A_06_P4398	YJR154W
A_06_P4399	YJR155W
A_06_P4400	YJR156C
A_06_P4401	YJR157W
A_06_P4402	YJR158W
A_06_P4403	YJR159W
A_06_P4404	YJR160C
A_06_P4405	YJR161C

A_06_P4406	YJR162C
A_06_P4407	YKL001C
A_06_P4408	YKL002W
A_06_P4409	YKL003C
A_06_P4410	YKL004W
A_06_P4411	YKL005C
A_06_P4412	YKL006C-A
A_06_P4413	YKL006W
A_06_P4414	YKL007W
A_06_P4415	YKL008C
A_06_P4416	YKL009W
A_06_P4417	YKL010C
A_06_P4418	YKL011C
A_06_P4419	YKL012W
A_06_P4420	YKL013C
A_06_P4421	YKL014C
A_06_P4422	YKL015W
A_06_P4423	YKL016C
A_06_P4424	YKL017C
A_06_P4425	YKL018C-A
A_06_P4426	YKL018W
A_06_P4427	YKL019W
A_06_P4428	YKL020C
A_06_P4429	YKL021C
A_06_P4430	YKL022C
A_06_P4431	YKL023W
A_06_P4432	YKL024C
A_06_P4433	YKL025C
A_06_P4434	YKL026C
A_06_P4435	YKL027W
A_06_P4436	YKL028W
A_06_P4437	YKL029C
A_06_P4438	YKL030W
A_06_P4439	YKL031W
A_06_P4440	YKL032C
A_06_P4441	YKL033W
A_06_P4442	YKL033W-A
A_06_P4443	YKL034W
A_06_P4444	YKL035W
A_06_P4445	YKL036C
A_06_P4446	YKL037W
A_06_P4447	YKL038W
A_06_P4448	YKL039W
A_06_P4449	YKL040C
A_06_P4450	YKL041W
A_06_P4451	YKL042W
A_06_P4452	YKL043W
A_06_P4453	YKL044W
A_06_P4454	YKL045W
A_06_P4455	YKL046C
A_06_P4456	YKL047W
A_06_P4457	YKL048C
A_06_P4458	YKL049C
A_06_P4459	YKL050C
A_06_P4460	YKL051W

A_06_P4461	YKL052C
A_06_P4462	YKL053C-A
A_06_P4463	YKL053W
A_06_P4464	YKL054C
A_06_P4465	YKL055C
A_06_P4466	YKL056C
A_06_P4467	YKL057C
A_06_P4468	YKL058W
A_06_P4469	YKL059C
A_06_P4470	YKL060C
A_06_P4471	YKL061W
A_06_P4472	YKL062W
A_06_P4473	YKL063C
A_06_P4474	YKL064W
A_06_P4475	YKL065C
A_06_P4476	YKL066W
A_06_P4477	YKL067W
A_06_P4478	YKL068W
A_06_P4479	YKL069W
A_06_P4480	YKL070W
A_06_P4481	YKL071W
A_06_P4482	YKL072W
A_06_P4483	YKL073W
A_06_P4484	YKL074C
A_06_P4485	YKL075C
A_06_P4486	YKL076C
A_06_P4487	YKL077W
A_06_P4488	YKL078W
A_06_P4489	YKL079W
A_06_P4490	YKL080W
A_06_P4491	YKL081W
A_06_P4492	YKL082C
A_06_P4493	YKL083W
A_06_P4494	YKL084W
A_06_P4495	YKL085W
A_06_P4496	YKL086W
A_06_P4497	YKL087C
A_06_P4498	YKL088W
A_06_P4499	YKL089W
A_06_P4500	YKL090W
A_06_P4501	YKL091C
A_06_P4502	YKL092C
A_06_P4503	YKL093W
A_06_P4504	YKL094W
A_06_P4505	YKL095W
A_06_P4506	YKL096W
A_06_P4507	YKL096W-A
A_06_P4508	YKL097C
A_06_P4509	YKL098W
A_06_P4510	YKL099C
A_06_P4511	YKL100C
A_06_P4512	YKL101W
A_06_P4513	YKL102C
A_06_P4514	YKL103C
A_06_P4515	YKL104C

A_06_P4516	YKL105C
A_06_P4517	YKL106C-A
A_06_P4518	YKL106W
A_06_P4519	YKL107W
A_06_P4520	YKL108W
A_06_P4521	YKL109W
A_06_P4522	YKL110C
A_06_P4523	YKL111C
A_06_P4524	YKL112W
A_06_P4525	YKL113C
A_06_P4526	YKL114C
A_06_P4527	YKL115C
A_06_P4528	YKL116C
A_06_P4529	YKL117W
A_06_P4530	YKL118W
A_06_P4531	YKL119C
A_06_P4532	YKL120W
A_06_P4533	YKL121W
A_06_P4534	YKL122C
A_06_P4535	YKL123W
A_06_P4536	YKL124W
A_06_P4537	YKL125W
A_06_P4538	YKL126W
A_06_P4539	YKL127W
A_06_P4540	YKL128C
A_06_P4541	YKL129C
A_06_P4542	YKL130C
A_06_P4543	YKL131W
A_06_P4544	YKL132C
A_06_P4545	YKL133C
A_06_P4546	YKL134C
A_06_P4547	YKL135C
A_06_P4548	YKL136W
A_06_P4549	YKL137W
A_06_P4550	YKL138C
A_06_P4551	YKL139W
A_06_P4552	YKL140W
A_06_P4553	YKL141W
A_06_P4554	YKL142W
A_06_P4555	YKL143W
A_06_P4556	YKL144C
A_06_P4557	YKL145W
A_06_P4558	YKL146W
A_06_P4559	YKL147C
A_06_P4560	YKL148C
A_06_P4561	YKL149C
A_06_P4562	YKL150W
A_06_P4563	YKL151C
A_06_P4564	YKL152C
A_06_P4565	YKL153W
A_06_P4566	YKL154W
A_06_P4567	YKL155C
A_06_P4568	YKL156W
A_06_P4569	YKL157W
A_06_P4570	YKL159C

A_06_P4571	YKL160W
A_06_P4572	YKL161C
A_06_P4573	YKL162C
A_06_P4574	YKL162C-A
A_06_P4575	YKL163W
A_06_P4576	YKL164C
A_06_P4577	YKL165C
A_06_P4578	YKL165C-A
A_06_P4579	YKL166C
A_06_P4580	YKL167C
A_06_P4581	YKL168C
A_06_P4582	YKL169C
A_06_P4583	YKL170W
A_06_P4584	YKL171W
A_06_P4585	YKL172W
A_06_P4586	YKL173W
A_06_P4587	YKL174C
A_06_P4588	YKL175W
A_06_P4589	YKL176C
A_06_P4590	YKL177W
A_06_P4591	YKL178C
A_06_P4592	YKL179C
A_06_P4593	YKL180W
A_06_P4594	YKL181W
A_06_P4595	YKL182W
A_06_P4596	YKL183W
A_06_P4597	YKL184W
A_06_P4598	YKL185W
A_06_P4599	YKL186C
A_06_P4600	YKL187C
A_06_P4601	YKL188C
A_06_P4602	YKL189W
A_06_P4603	YKL190W
A_06_P4604	YKL191W
A_06_P4605	YKL192C
A_06_P4606	YKL193C
A_06_P4607	YKL194C
A_06_P4608	YKL195W
A_06_P4609	YKL196C
A_06_P4610	YKL197C
A_06_P4611	YKL198C
A_06_P4612	YKL198C
A_06_P4613	YKL201C
A_06_P4614	YKL202W
A_06_P4615	YKL203C
A_06_P4616	YKL204W
A_06_P4617	YKL205W
A_06_P4618	YKL206C
A_06_P4619	YKL207W
A_06_P4620	YKL208W
A_06_P4621	YKL209C
A_06_P4622	YKL210W
A_06_P4623	YKL211C
A_06_P4624	YKL212W
A_06_P4625	YKL213C

A_06_P4626	YKL214C
A_06_P4627	YKL215C
A_06_P4628	YKL216W
A_06_P4629	YKL217W
A_06_P4630	YKL218C
A_06_P4631	YKL219W
A_06_P4632	YKL220C
A_06_P4633	YKL221W
A_06_P4634	YKL222C
A_06_P4635	YKL223W
A_06_P4636	YKL224C
A_06_P4637	YKL225W
A_06_P4638	YKR001C
A_06_P4639	YKR002W
A_06_P4640	YKR003W
A_06_P4641	YKR004C
A_06_P4642	YKR005C
A_06_P4643	YKR006C
A_06_P4644	YKR007W
A_06_P4645	YKR008W
A_06_P4646	YKR009C
A_06_P4647	YKR010C
A_06_P4648	YKR011C
A_06_P4649	YKR012C
A_06_P4650	YKR013W
A_06_P4651	YKR014C
A_06_P4652	YKR015C
A_06_P4653	YKR016W
A_06_P4654	YKR017C
A_06_P4655	YKR018C
A_06_P4656	YKR019C
A_06_P4657	YKR020W
A_06_P4658	YKR021W
A_06_P4659	YKR022C
A_06_P4660	YKR023W
A_06_P4661	YKR024C
A_06_P4662	YKR025W
A_06_P4663	YKR026C
A_06_P4664	YKR027W
A_06_P4665	YKR028W
A_06_P4666	YKR029C
A_06_P4667	YKR030W
A_06_P4668	YKR031C
A_06_P4669	YKR032W
A_06_P4670	YKR033C
A_06_P4671	YKR034W
A_06_P4672	YKR035C
A_06_P4673	YKR035W-A
A_06_P4674	YKR036C
A_06_P4675	YKR037C
A_06_P4676	YKR038C
A_06_P4677	YKR039W
A_06_P4678	YKR040C
A_06_P4679	YKR041W
A_06_P4680	YKR042W

A_06_P4681	YKR043C
A_06_P4682	YKR044W
A_06_P4683	YKR045C
A_06_P4684	YKR046C
A_06_P4685	YKR047W
A_06_P4686	YKR048C
A_06_P4687	YKR049C
A_06_P4688	YKR050W
A_06_P4689	YKR051W
A_06_P4690	YKR052C
A_06_P4691	YKR053C
A_06_P4692	YKR054C
A_06_P4693	YKR055W
A_06_P4694	YKR056W
A_06_P4695	YKR057W
A_06_P4696	YKR058W
A_06_P4697	YKR059W
A_06_P4698	YKR060W
A_06_P4699	YKR061W
A_06_P4700	YKR062W
A_06_P4701	YKR063C
A_06_P4702	YKR064W
A_06_P4703	YKR065C
A_06_P4704	YKR066C
A_06_P4705	YKR067W
A_06_P4706	YKR068C
A_06_P4707	YKR069W
A_06_P4708	YKR070W
A_06_P4709	YKR071C
A_06_P4710	YKR072C
A_06_P4711	YKR073C
A_06_P4712	YKR074W
A_06_P4713	YKR075C
A_06_P4714	YKR076W
A_06_P4715	YKR077W
A_06_P4716	YKR078W
A_06_P4717	YKR079C
A_06_P4718	YKR080W
A_06_P4719	YKR081C
A_06_P4720	YKR082W
A_06_P4721	YKR083C
A_06_P4722	YKR084C
A_06_P4723	YKR085C
A_06_P4724	YKR086W
A_06_P4725	YKR087C
A_06_P4726	YKR088C
A_06_P4727	YKR089C
A_06_P4728	YKR090W
A_06_P4729	YKR091W
A_06_P4730	YKR092C
A_06_P4731	YKR093W
A_06_P4732	YKR094C
A_06_P4733	YKR095W
A_06_P4734	YKR096W
A_06_P4735	YKR097W

A_06_P4736	YKR098C
A_06_P4737	YKR099W
A_06_P4738	YKR100C
A_06_P4739	YKR101W
A_06_P4740	YKR102W
A_06_P4741	YKR103W
A_06_P4742	YKR104W
A_06_P4743	YKR105C
A_06_P4744	YKR106W
A_06_P4745	YLL001W
A_06_P4746	YLL002W
A_06_P4747	YLL003W
A_06_P4748	YLL004W
A_06_P4749	YLL005C
A_06_P4750	YLL006W
A_06_P4751	YLL007C
A_06_P4752	YLL008W
A_06_P4753	YLL009C
A_06_P4754	YLL010C
A_06_P4755	YLL011W
A_06_P4756	YLL012W
A_06_P4757	YLL013C
A_06_P4758	YLL014W
A_06_P4759	YLL015W
A_06_P4760	YLL016W
A_06_P4761	YLL017W
A_06_P4762	YLL018C
A_06_P4763	YLL018C-A
A_06_P4764	YLL019C
A_06_P4765	YLL020C
A_06_P4766	YLL021W
A_06_P4767	YLL022C
A_06_P4768	YLL023C
A_06_P4769	YLL024C
A_06_P4770	YLL025W
A_06_P4771	YLL026W
A_06_P4772	YLL027W
A_06_P4773	YLL028W
A_06_P4774	YLL029W
A_06_P4775	YLL030C
A_06_P4776	YLL031C
A_06_P4777	YLL032C
A_06_P4778	YLL033W
A_06_P4779	YLL034C
A_06_P4780	YLL035W
A_06_P4781	YLL036C
A_06_P4782	YLL037W
A_06_P4783	YLL038C
A_06_P4784	YLL039C
A_06_P4785	YLL040C
A_06_P4786	YLL041C
A_06_P4787	YLL042C
A_06_P4788	YLL043W
A_06_P4789	YLL044W
A_06_P4790	YLL045C

A_06_P4791	YLL046C
A_06_P4792	YLL047W
A_06_P4793	YLL048C
A_06_P4794	YLL049W
A_06_P4795	YLL050C
A_06_P4796	YLL051C
A_06_P4797	YLL052C
A_06_P4798	YLL053C
A_06_P4799	YLL054C
A_06_P4800	YLL055W
A_06_P4801	YLL056C
A_06_P4802	YLL057C
A_06_P4803	YLL058W
A_06_P4804	YLL059C
A_06_P4805	YLL060C
A_06_P4806	YLL061W
A_06_P4807	YLL062C
A_06_P4808	YLL063C
A_06_P4809	YLL064C
A_06_P4810	YLL065W
A_06_P4811	YLL066C
A_06_P4812	YLL067C
A_06_P4813	YLR001C
A_06_P4814	YLR002C
A_06_P4815	YLR003C
A_06_P4816	YLR004C
A_06_P4817	YLR005W
A_06_P4818	YLR006C
A_06_P4819	YLR007W
A_06_P4820	YLR008C
A_06_P4821	YLR009W
A_06_P4822	YLR010C
A_06_P4823	YLR011W
A_06_P4824	YLR012C
A_06_P4825	YLR013W
A_06_P4826	YLR014C
A_06_P4827	YLR015W
A_06_P4828	YLR016C
A_06_P4829	YLR017W
A_06_P4830	YLR018C
A_06_P4831	YLR019W
A_06_P4832	YLR020C
A_06_P4833	YLR021W
A_06_P4834	YLR022C
A_06_P4835	YLR023C
A_06_P4836	YLR024C
A_06_P4837	YLR025W
A_06_P4838	YLR026C
A_06_P4839	YLR027C
A_06_P4840	YLR028C
A_06_P4841	YLR029C
A_06_P4842	YLR030W
A_06_P4843	YLR031W
A_06_P4844	YLR032W
A_06_P4845	YLR033W

A_06_P4846	YLR034C
A_06_P4847	YLR035C
A_06_P4848	YLR036C
A_06_P4849	YLR037C
A_06_P4850	YLR038C
A_06_P4851	YLR039C
A_06_P4852	YLR040C
A_06_P4853	YLR041W
A_06_P4854	YLR042C
A_06_P4855	YLR043C
A_06_P4856	YLR044C
A_06_P4857	YLR045C
A_06_P4858	YLR046C
A_06_P4859	YLR047C
A_06_P4860	YLR048W
A_06_P4861	YLR049C
A_06_P4862	YLR050C
A_06_P4863	YLR051C
A_06_P4864	YLR052W
A_06_P4865	YLR053C
A_06_P4866	YLR054C
A_06_P4867	YLR055C
A_06_P4868	YLR056W
A_06_P4869	YLR057W
A_06_P4870	YLR058C
A_06_P4871	YLR059C
A_06_P4872	YLR060W
A_06_P4873	YLR061W
A_06_P4874	YLR062C
A_06_P4875	YLR063W
A_06_P4876	YLR064W
A_06_P4877	YLR065C
A_06_P4878	YLR066W
A_06_P4879	YLR067C
A_06_P4880	YLR068W
A_06_P4881	YLR069C
A_06_P4882	YLR070C
A_06_P4883	YLR071C
A_06_P4884	YLR072W
A_06_P4885	YLR073C
A_06_P4886	YLR074C
A_06_P4887	YLR075W
A_06_P4888	YLR076C
A_06_P4889	YLR077W
A_06_P4890	YLR078C
A_06_P4891	YLR079W
A_06_P4892	YLR080W
A_06_P4893	YLR081W
A_06_P4894	YLR082C
A_06_P4895	YLR083C
A_06_P4896	YLR084C
A_06_P4897	YLR085C
A_06_P4898	YLR086W
A_06_P4899	YLR087C
A_06_P4900	YLR088W

A_06_P4901	YLR089C
A_06_P4902	YLR090W
A_06_P4903	YLR091W
A_06_P4904	YLR092W
A_06_P4905	YLR093C
A_06_P4906	YLR094C
A_06_P4907	YLR095C
A_06_P4908	YLR096W
A_06_P4909	YLR097C
A_06_P4910	YLR098C
A_06_P4911	YLR099C
A_06_P4912	YLR099W-A
A_06_P4913	YLR100W
A_06_P4914	YLR101C
A_06_P4915	YLR102C
A_06_P4916	YLR103C
A_06_P4917	YLR104W
A_06_P4918	YLR105C
A_06_P4919	YLR106C
A_06_P4920	YLR107W
A_06_P4921	YLR108C
A_06_P4922	YLR109W
A_06_P4923	YLR110C
A_06_P4924	YLR111W
A_06_P4925	YLR112W
A_06_P4926	YLR113W
A_06_P4927	YLR114C
A_06_P4928	YLR115W
A_06_P4929	YLR116W
A_06_P4930	YLR117C
A_06_P4931	YLR118C
A_06_P4932	YLR119W
A_06_P4933	YLR120C
A_06_P4934	YLR121C
A_06_P4935	YLR122C
A_06_P4936	YLR123C
A_06_P4937	YLR124W
A_06_P4938	YLR125W
A_06_P4939	YLR126C
A_06_P4940	YLR127C
A_06_P4941	YLR128W
A_06_P4942	YLR129W
A_06_P4943	YLR130C
A_06_P4944	YLR131C
A_06_P4945	YLR132C
A_06_P4946	YLR133W
A_06_P4947	YLR134W
A_06_P4948	YLR135W
A_06_P4949	YLR136C
A_06_P4950	YLR137W
A_06_P4951	YLR138W
A_06_P4952	YLR139C
A_06_P4953	YLR140W
A_06_P4954	YLR141W
A_06_P4955	YLR142W

A_06_P4956	YLR143W
A_06_P4957	YLR144C
A_06_P4958	YLR145W
A_06_P4959	YLR146C
A_06_P4960	YLR147C
A_06_P4961	YLR148W
A_06_P4962	YLR149C
A_06_P4963	YLR149C-A
A_06_P4964	YLR150W
A_06_P4965	YLR151C
A_06_P4966	YLR152C
A_06_P4967	YLR153C
A_06_P4968	YLR154C
A_06_P4969	YLR155C
A_06_P4970	YLR156W
A_06_P4971	YLR157C
A_06_P4972	YLR158C
A_06_P4973	YLR159W
A_06_P4974	YLR160C
A_06_P4975	YLR161W
A_06_P4976	YLR162W
A_06_P4977	YLR163C
A_06_P4978	YLR164W
A_06_P4979	YLR165C
A_06_P4980	YLR166C
A_06_P4981	YLR167W
A_06_P4982	YLR168C
A_06_P4983	YLR169W
A_06_P4984	YLR170C
A_06_P4985	YLR171W
A_06_P4986	YLR172C
A_06_P4987	YLR173W
A_06_P4988	YLR174W
A_06_P4989	YLR175W
A_06_P4990	YLR176C
A_06_P4991	YLR177W
A_06_P4992	YLR178C
A_06_P4993	YLR179C
A_06_P4994	YLR180W
A_06_P4995	YLR181C
A_06_P4996	YLR182W
A_06_P4997	YLR183C
A_06_P4998	YLR184W
A_06_P4999	YLR185W
A_06_P5000	YLR186W
A_06_P5001	YLR187W
A_06_P5002	YLR188W
A_06_P5003	YLR189C
A_06_P5004	YLR190W
A_06_P5005	YLR191W
A_06_P5006	YLR192C
A_06_P5007	YLR193C
A_06_P5008	YLR194C
A_06_P5009	YLR195C
A_06_P5010	YLR196W

A_06_P5011	YLR197W
A_06_P5012	YLR198C
A_06_P5013	YLR199C
A_06_P5014	YLR200W
A_06_P5015	YLR201C
A_06_P5016	YLR202C
A_06_P5017	YLR203C
A_06_P5018	YLR204W
A_06_P5019	YLR205C
A_06_P5020	YLR206W
A_06_P5021	YLR207W
A_06_P5022	YLR208W
A_06_P5023	YLR209C
A_06_P5024	YLR210W
A_06_P5025	YLR211C
A_06_P5026	YLR212C
A_06_P5027	YLR213C
A_06_P5028	YLR214W
A_06_P5029	YLR215C
A_06_P5030	YLR216C
A_06_P5031	YLR217W
A_06_P5032	YLR218C
A_06_P5033	YLR219W
A_06_P5034	YLR220W
A_06_P5035	YLR221C
A_06_P5036	YLR222C
A_06_P5037	YLR223C
A_06_P5038	YLR224W
A_06_P5039	YLR225C
A_06_P5040	YLR226W
A_06_P5041	YLR227C
A_06_P5042	YLR228C
A_06_P5043	YLR229C
A_06_P5044	YLR230W
A_06_P5045	YLR231C
A_06_P5046	YLR232W
A_06_P5047	YLR233C
A_06_P5048	YLR234W
A_06_P5049	YLR235C
A_06_P5050	YLR236C
A_06_P5051	YLR237W
A_06_P5052	YLR238W
A_06_P5053	YLR239C
A_06_P5054	YLR240W
A_06_P5055	YLR241W
A_06_P5056	YLR242C
A_06_P5057	YLR243W
A_06_P5058	YLR244C
A_06_P5059	YLR245C
A_06_P5060	YLR246W
A_06_P5061	YLR247C
A_06_P5062	YLR248W
A_06_P5063	YLR249W
A_06_P5064	YLR250W
A_06_P5065	YLR251W

A_06_P5066	YLR252W
A_06_P5067	YLR253W
A_06_P5068	YLR254C
A_06_P5069	YLR255C
A_06_P5070	YLR256W
A_06_P5071	YLR257W
A_06_P5072	YLR258W
A_06_P5073	YLR259C
A_06_P5074	YLR260W
A_06_P5075	YLR261C
A_06_P5076	YLR262C
A_06_P5077	YLR262C-A
A_06_P5078	YLR263W
A_06_P5079	YLR264W
A_06_P5080	YLR265C
A_06_P5081	YLR266C
A_06_P5082	YLR267W
A_06_P5083	YLR268W
A_06_P5084	YLR269C
A_06_P5085	YLR270W
A_06_P5086	YLR271W
A_06_P5087	YLR272C
A_06_P5088	YLR273C
A_06_P5089	YLR274W
A_06_P5090	YLR275W
A_06_P5091	YLR276C
A_06_P5092	YLR277C
A_06_P5093	YLR278C
A_06_P5094	YLR279W
A_06_P5095	YLR280C
A_06_P5096	YLR281C
A_06_P5097	YLR282C
A_06_P5098	YLR283W
A_06_P5099	YLR284C
A_06_P5100	YLR285W
A_06_P5101	YLR286C
A_06_P5102	YLR287C
A_06_P5103	YLR287C-A
A_06_P5104	YLR288C
A_06_P5105	YLR289W
A_06_P5106	YLR290C
A_06_P5107	YLR291C
A_06_P5108	YLR292C
A_06_P5109	YLR293C
A_06_P5110	YLR294C
A_06_P5111	YLR295C
A_06_P5112	YLR296W
A_06_P5113	YLR297W
A_06_P5114	YLR298C
A_06_P5115	YLR299W
A_06_P5116	YLR300W
A_06_P5117	YLR301W
A_06_P5118	YLR302C
A_06_P5119	YLR303W
A_06_P5120	YLR304C

A_06_P5121	YLR305C
A_06_P5122	YLR306W
A_06_P5123	YLR307W
A_06_P5124	YLR308W
A_06_P5125	YLR309C
A_06_P5126	YLR310C
A_06_P5127	YLR311C
A_06_P5128	YLR312C
A_06_P5129	YLR312W-A
A_06_P5130	YLR313C
A_06_P5131	YLR314C
A_06_P5132	YLR315W
A_06_P5133	YLR316C
A_06_P5134	YLR317W
A_06_P5135	YLR318W
A_06_P5136	YLR319C
A_06_P5137	YLR320W
A_06_P5138	YLR321C
A_06_P5139	YLR322W
A_06_P5140	YLR323C
A_06_P5141	YLR324W
A_06_P5142	YLR325C
A_06_P5143	YLR326W
A_06_P5144	YLR327C
A_06_P5145	YLR328W
A_06_P5146	YLR329W
A_06_P5147	YLR330W
A_06_P5148	YLR331C
A_06_P5149	YLR332W
A_06_P5150	YLR333C
A_06_P5151	YLR334C
A_06_P5152	YLR335W
A_06_P5153	YLR336C
A_06_P5154	YLR337C
A_06_P5155	YLR338W
A_06_P5156	YLR339C
A_06_P5157	YLR340W
A_06_P5158	YLR341W
A_06_P5159	YLR342W
A_06_P5160	YLR343W
A_06_P5161	YLR344W
A_06_P5162	YLR345W
A_06_P5163	YLR346C
A_06_P5164	YLR347C
A_06_P5165	YLR348C
A_06_P5166	YLR349W
A_06_P5167	YLR350W
A_06_P5168	YLR351C
A_06_P5169	YLR352W
A_06_P5170	YLR353W
A_06_P5171	YLR354C
A_06_P5172	YLR355C
A_06_P5173	YLR356W
A_06_P5174	YLR357W
A_06_P5175	YLR358C

A_06_P5176	YLR359W
A_06_P5177	YLR360W
A_06_P5178	YLR361C
A_06_P5179	YLR362W
A_06_P5180	YLR363C
A_06_P5181	YLR363W-A
A_06_P5182	YLR364W
A_06_P5183	YLR365W
A_06_P5184	YLR366W
A_06_P5185	YLR367W
A_06_P5186	YLR368W
A_06_P5187	YLR369W
A_06_P5188	YLR370C
A_06_P5189	YLR371W
A_06_P5190	YLR372W
A_06_P5191	YLR373C
A_06_P5192	YLR374C
A_06_P5193	YLR375W
A_06_P5194	YLR376C
A_06_P5195	YLR377C
A_06_P5196	YLR378C
A_06_P5197	YLR379W
A_06_P5198	YLR380W
A_06_P5199	YLR381W
A_06_P5200	YLR382C
A_06_P5201	YLR383W
A_06_P5202	YLR384C
A_06_P5203	YLR385C
A_06_P5204	YLR386W
A_06_P5205	YLR387C
A_06_P5206	YLR388W
A_06_P5207	YLR389C
A_06_P5208	YLR390W
A_06_P5209	YLR390W-A
A_06_P5210	YLR392C
A_06_P5211	YLR393W
A_06_P5212	YLR394W
A_06_P5213	YLR395C
A_06_P5214	YLR396C
A_06_P5215	YLR397C
A_06_P5216	YLR398C
A_06_P5217	YLR399C
A_06_P5218	YLR400W
A_06_P5219	YLR401C
A_06_P5220	YLR402W
A_06_P5221	YLR403W
A_06_P5222	YLR404W
A_06_P5223	YLR405W
A_06_P5224	YLR406C
A_06_P5225	YLR407W
A_06_P5226	YLR408C
A_06_P5227	YLR409C
A_06_P5228	YLR410W
A_06_P5229	YLR411W
A_06_P5230	YLR412W

A_06_P5231	YLR413W
A_06_P5232	YLR414C
A_06_P5233	YLR415C
A_06_P5234	YLR416C
A_06_P5235	YLR417W
A_06_P5236	YLR418C
A_06_P5237	YLR419W
A_06_P5238	YLR420W
A_06_P5239	YLR421C
A_06_P5240	YLR422W
A_06_P5241	YLR423C
A_06_P5242	YLR424W
A_06_P5243	YLR425W
A_06_P5244	YLR426W
A_06_P5245	YLR427W
A_06_P5246	YLR428C
A_06_P5247	YLR429W
A_06_P5248	YLR430W
A_06_P5249	YLR431C
A_06_P5250	YLR432W
A_06_P5251	YLR433C
A_06_P5252	YLR434C
A_06_P5253	YLR435W
A_06_P5254	YLR436C
A_06_P5255	YLR437C
A_06_P5256	YLR438C-A
A_06_P5257	YLR438W
A_06_P5258	YLR439W
A_06_P5259	YLR440C
A_06_P5260	YLR441C
A_06_P5261	YLR442C
A_06_P5262	YLR443W
A_06_P5263	YLR444C
A_06_P5264	YLR445W
A_06_P5265	YLR446W
A_06_P5266	YLR447C
A_06_P5267	YLR448W
A_06_P5268	YLR449W
A_06_P5269	YLR450W
A_06_P5270	YLR451W
A_06_P5271	YLR452C
A_06_P5272	YLR453C
A_06_P5273	YLR454W
A_06_P5274	YLR455W
A_06_P5275	YLR456W
A_06_P5276	YLR457C
A_06_P5277	YLR458W
A_06_P5278	YLR459W
A_06_P5279	YLR460C
A_06_P5280	YLR461W
A_06_P5281	YLR462W
A_06_P5282	YLR463C
A_06_P5283	YLR464W
A_06_P5284	YLR465C
A_06_P5285	YLR466W

A_06_P5286	YLR467W
A_06_P5287	YML001W
A_06_P5288	YML002W
A_06_P5289	YML003W
A_06_P5290	YML004C
A_06_P5291	YML005W
A_06_P5292	YML006C
A_06_P5293	YML007C-A
A_06_P5294	YML007W
A_06_P5295	YML008C
A_06_P5296	YML009C
A_06_P5297	YML009C-A
A_06_P5299	YML009W-B
A_06_P5298	YML010W
A_06_P5300	YML011C
A_06_P5302	YML012C-A
A_06_P5301	YML012W
A_06_P5303	YML013W
A_06_P5304	YML014W
A_06_P5305	YML015C
A_06_P5306	YML016C
A_06_P5307	YML017W
A_06_P5308	YML018C
A_06_P5309	YML019W
A_06_P5310	YML020W
A_06_P5311	YML021C
A_06_P5312	YML022W
A_06_P5313	YML023C
A_06_P5314	YML024W
A_06_P5315	YML025C
A_06_P5316	YML026C
A_06_P5317	YML027W
A_06_P5318	YML028W
A_06_P5319	YML029W
A_06_P5320	YML030W
A_06_P5321	YML031W
A_06_P5322	YML032C
A_06_P5325	YML034C-A
A_06_P5323	YML034W
A_06_P5324	YML035C
A_06_P5326	YML036W
A_06_P5327	YML037C
A_06_P5328	YML038C
A_06_P5329	YML041C
A_06_P5330	YML042W
A_06_P5331	YML043C
A_06_P5332	YML046W
A_06_P5333	YML047C
A_06_P5335	YML047W-A
A_06_P5334	YML048W
A_06_P5336	YML049C
A_06_P5337	YML050W
A_06_P5338	YML051W
A_06_P5339	YML052W
A_06_P5340	YML053C

A_06_P5341	YML054C
A_06_P5342	YML055W
A_06_P5343	YML056C
A_06_P5345	YML057C-A
A_06_P5344	YML057W
A_06_P5346	YML058W
A_06_P5347	YML058W-A
A_06_P5348	YML059C
A_06_P5349	YML060W
A_06_P5350	YML061C
A_06_P5351	YML062C
A_06_P5352	YML063W
A_06_P5353	YML064C
A_06_P5354	YML065W
A_06_P5355	YML066C
A_06_P5356	YML067C
A_06_P5357	YML068W
A_06_P5358	YML069W
A_06_P5359	YML070W
A_06_P5360	YML071C
A_06_P5361	YML072C
A_06_P5362	YML073C
A_06_P5363	YML074C
A_06_P5364	YML075C
A_06_P5365	YML076C
A_06_P5366	YML077W
A_06_P5367	YML078W
A_06_P5368	YML079W
A_06_P5369	YML080W
A_06_P5370	YML081C-A
A_06_P5371	YML081W
A_06_P5372	YML082W
A_06_P5373	YML083C
A_06_P5374	YML084W
A_06_P5375	YML085C
A_06_P5376	YML086C
A_06_P5377	YML087C
A_06_P5378	YML088W
A_06_P5379	YML089C
A_06_P5380	YML090W
A_06_P5381	YML091C
A_06_P5382	YML092C
A_06_P5383	YML093W
A_06_P5386	YML094C-A
A_06_P5384	YML094W
A_06_P5385	YML095C
A_06_P5387	YML096W
A_06_P5388	YML097C
A_06_P5389	YML098W
A_06_P5390	YML099C
A_06_P5392	YML099W-A
A_06_P5391	YML100W
A_06_P5393	YML101C
A_06_P5394	YML101C-A
A_06_P5395	YML102W

A_06_P5396	YML103C
A_06_P5397	YML104C
A_06_P5398	YML105C
A_06_P5399	YML106W
A_06_P5400	YML107C
A_06_P5401	YML108W
A_06_P5402	YML109W
A_06_P5403	YML110C
A_06_P5404	YML111W
A_06_P5405	YML112W
A_06_P5406	YML113W
A_06_P5407	YML114C
A_06_P5408	YML115C
A_06_P5409	YML116W
A_06_P5411	YML116W-A
A_06_P5410	YML117W
A_06_P5412	YML118W
A_06_P5413	YML119W
A_06_P5414	YML120C
A_06_P5415	YML121W
A_06_P5416	YML122C
A_06_P5417	YML123C
A_06_P5418	YML124C
A_06_P5419	YML125C
A_06_P5420	YML126C
A_06_P5421	YML127W
A_06_P5422	YML128C
A_06_P5423	YML129C
A_06_P5424	YML130C
A_06_P5425	YML131W
A_06_P5426	YML132W
A_06_P5427	YML133C
A_06_P5428	YMR001C
A_06_P5429	YMR002W
A_06_P5430	YMR003W
A_06_P5431	YMR004W
A_06_P5432	YMR005W
A_06_P5433	YMR006C
A_06_P5434	YMR007W
A_06_P5435	YMR008C
A_06_P5436	YMR009W
A_06_P5437	YMR010W
A_06_P5438	YMR011W
A_06_P5439	YMR012W
A_06_P5440	YMR013C
A_06_P5441	YMR013W-A
A_06_P5442	YMR014W
A_06_P5443	YMR015C
A_06_P5444	YMR016C
A_06_P5445	YMR017W
A_06_P5446	YMR018W
A_06_P5447	YMR019W
A_06_P5448	YMR020W
A_06_P5449	YMR021C
A_06_P5450	YMR022W

A_06_P5451	YMR023C
A_06_P5452	YMR024W
A_06_P5453	YMR025W
A_06_P5454	YMR026C
A_06_P5455	YMR027W
A_06_P5456	YMR028W
A_06_P5457	YMR029C
A_06_P5458	YMR030W
A_06_P5459	YMR031C
A_06_P5460	YMR031W-A
A_06_P5461	YMR032W
A_06_P5462	YMR033W
A_06_P5463	YMR034C
A_06_P5464	YMR035W
A_06_P5465	YMR036C
A_06_P5466	YMR037C
A_06_P5467	YMR038C
A_06_P5468	YMR039C
A_06_P5469	YMR040W
A_06_P5470	YMR041C
A_06_P5471	YMR042W
A_06_P5472	YMR043W
A_06_P5473	YMR044W
A_06_P5474	YMR046W-A
A_06_P5475	YMR047C
A_06_P5476	YMR048W
A_06_P5477	YMR049C
A_06_P5478	YMR052C-A
A_06_P5479	YMR052W
A_06_P5480	YMR053C
A_06_P5481	YMR054W
A_06_P5482	YMR055C
A_06_P5483	YMR056C
A_06_P5484	YMR057C
A_06_P5485	YMR058W
A_06_P5486	YMR059W
A_06_P5487	YMR060C
A_06_P5488	YMR061W
A_06_P5489	YMR062C
A_06_P5490	YMR063W
A_06_P5491	YMR064W
A_06_P5492	YMR065W
A_06_P5493	YMR066W
A_06_P5494	YMR067C
A_06_P5495	YMR068W
A_06_P5496	YMR069W
A_06_P5497	YMR070W
A_06_P5498	YMR071C
A_06_P5499	YMR072W
A_06_P5500	YMR073C
A_06_P5501	YMR074C
A_06_P5502	YMR075C-A
A_06_P5503	YMR075W
A_06_P5504	YMR076C
A_06_P5505	YMR077C

A_06_P5506	YMR078C
A_06_P5507	YMR079W
A_06_P5508	YMR080C
A_06_P5509	YMR081C
A_06_P5510	YMR082C
A_06_P5511	YMR083W
A_06_P5512	YMR084W
A_06_P5513	YMR085W
A_06_P5514	YMR086C-A
A_06_P5515	YMR086W
A_06_P5516	YMR087W
A_06_P5517	YMR088C
A_06_P5518	YMR089C
A_06_P5519	YMR090W
A_06_P5520	YMR091C
A_06_P5521	YMR092C
A_06_P5522	YMR093W
A_06_P5523	YMR094W
A_06_P5524	YMR095C
A_06_P5525	YMR096W
A_06_P5526	YMR097C
A_06_P5527	YMR098C
A_06_P5528	YMR099C
A_06_P5529	YMR100W
A_06_P5530	YMR101C
A_06_P5531	YMR102C
A_06_P5532	YMR103C
A_06_P5533	YMR104C
A_06_P5534	YMR105C
A_06_P5535	YMR106C
A_06_P5536	YMR107W
A_06_P5537	YMR108W
A_06_P5538	YMR109W
A_06_P5539	YMR110C
A_06_P5540	YMR111C
A_06_P5541	YMR112C
A_06_P5542	YMR113W
A_06_P5543	YMR114C
A_06_P5544	YMR115W
A_06_P5545	YMR116C
A_06_P5546	YMR117C
A_06_P5547	YMR118C
A_06_P5548	YMR119W
A_06_P5549	YMR119W-A
A_06_P5550	YMR120C
A_06_P5551	YMR121C
A_06_P5552	YMR122C
A_06_P5553	YMR122W-A
A_06_P5554	YMR123W
A_06_P5555	YMR124W
A_06_P5556	YMR125W
A_06_P5557	YMR126C
A_06_P5558	YMR127C
A_06_P5559	YMR128W
A_06_P5560	YMR129W

A_06_P5561	YMR130W
A_06_P5562	YMR131C
A_06_P5563	YMR132C
A_06_P5564	YMR133W
A_06_P5565	YMR134W
A_06_P5566	YMR135C
A_06_P5567	YMR135W-A
A_06_P5568	YMR136W
A_06_P5569	YMR137C
A_06_P5570	YMR138W
A_06_P5571	YMR139W
A_06_P5572	YMR140W
A_06_P5573	YMR141C
A_06_P5574	YMR142C
A_06_P5575	YMR143W
A_06_P5576	YMR144W
A_06_P5577	YMR145C
A_06_P5578	YMR146C
A_06_P5579	YMR147W
A_06_P5580	YMR148W
A_06_P5581	YMR149W
A_06_P5582	YMR150C
A_06_P5583	YMR151W
A_06_P5584	YMR152W
A_06_P5585	YMR153C-A
A_06_P5586	YMR153W
A_06_P5587	YMR154C
A_06_P5588	YMR155W
A_06_P5589	YMR156C
A_06_P5590	YMR157C
A_06_P5591	YMR158C-A
A_06_P5592	YMR158W
A_06_P5593	YMR158W-B
A_06_P5594	YMR159C
A_06_P5595	YMR160W
A_06_P5596	YMR161W
A_06_P5597	YMR162C
A_06_P5598	YMR163C
A_06_P5599	YMR164C
A_06_P5600	YMR165C
A_06_P5601	YMR166C
A_06_P5602	YMR167W
A_06_P5603	YMR168C
A_06_P5604	YMR169C
A_06_P5605	YMR170C
A_06_P5606	YMR171C
A_06_P5607	YMR172C-A
A_06_P5608	YMR172W
A_06_P5609	YMR173W
A_06_P5610	YMR173W-A
A_06_P5611	YMR174C
A_06_P5612	YMR175W
A_06_P5613	YMR176W
A_06_P5614	YMR177W
A_06_P5615	YMR178W

A_06_P5616	YMR179W
A_06_P5617	YMR180C
A_06_P5618	YMR181C
A_06_P5619	YMR182C
A_06_P5620	YMR183C
A_06_P5621	YMR184W
A_06_P5622	YMR185W
A_06_P5623	YMR186W
A_06_P5624	YMR187C
A_06_P5625	YMR188C
A_06_P5626	YMR189W
A_06_P5627	YMR190C
A_06_P5628	YMR191W
A_06_P5629	YMR192W
A_06_P5630	YMR193C-A
A_06_P5631	YMR193W
A_06_P5632	YMR194C-A
A_06_P5633	YMR194W
A_06_P5634	YMR195W
A_06_P5635	YMR196W
A_06_P5636	YMR197C
A_06_P5637	YMR198W
A_06_P5638	YMR199W
A_06_P5639	YMR200W
A_06_P5640	YMR201C
A_06_P5641	YMR202W
A_06_P5642	YMR203W
A_06_P5643	YMR204C
A_06_P5644	YMR205C
A_06_P5645	YMR206W
A_06_P5646	YMR207C
A_06_P5647	YMR208W
A_06_P5648	YMR209C
A_06_P5649	YMR210W
A_06_P5650	YMR211W
A_06_P5651	YMR212C
A_06_P5652	YMR213W
A_06_P5653	YMR214W
A_06_P5654	YMR215W
A_06_P5655	YMR216C
A_06_P5656	YMR217W
A_06_P5657	YMR218C
A_06_P5658	YMR219W
A_06_P5659	YMR220W
A_06_P5660	YMR221C
A_06_P5661	YMR222C
A_06_P5662	YMR223W
A_06_P5663	YMR224C
A_06_P5664	YMR225C
A_06_P5665	YMR226C
A_06_P5666	YMR227C
A_06_P5667	YMR228W
A_06_P5668	YMR229C
A_06_P5669	YMR230W
A_06_P5670	YMR231W

A_06_P5671	YMR232W
A_06_P5672	YMR233W
A_06_P5673	YMR234W
A_06_P5674	YMR235C
A_06_P5675	YMR236W
A_06_P5676	YMR237W
A_06_P5677	YMR238W
A_06_P5678	YMR239C
A_06_P5679	YMR240C
A_06_P5680	YMR241W
A_06_P5681	YMR242C
A_06_P5682	YMR243C
A_06_P5683	YMR244C-A
A_06_P5684	YMR244W
A_06_P5685	YMR245W
A_06_P5686	YMR246W
A_06_P5687	YMR247C
A_06_P5688	YMR250W
A_06_P5689	YMR251W
A_06_P5690	YMR251W-A
A_06_P5691	YMR252C
A_06_P5692	YMR253C
A_06_P5693	YMR254C
A_06_P5694	YMR255W
A_06_P5695	YMR256C
A_06_P5696	YMR257C
A_06_P5697	YMR258C
A_06_P5698	YMR259C
A_06_P5699	YMR260C
A_06_P5700	YMR261C
A_06_P5701	YMR262W
A_06_P5702	YMR263W
A_06_P5703	YMR264W
A_06_P5704	YMR265C
A_06_P5705	YMR266W
A_06_P5706	YMR267W
A_06_P5707	YMR268C
A_06_P5708	YMR269W
A_06_P5709	YMR270C
A_06_P5710	YMR271C
A_06_P5711	YMR272C
A_06_P5712	YMR273C
A_06_P5713	YMR274C
A_06_P5714	YMR275C
A_06_P5715	YMR276W
A_06_P5716	YMR277W
A_06_P5717	YMR278W
A_06_P5718	YMR279C
A_06_P5719	YMR280C
A_06_P5720	YMR281W
A_06_P5721	YMR282C
A_06_P5722	YMR283C
A_06_P5723	YMR284W
A_06_P5724	YMR285C
A_06_P5725	YMR286W

A_06_P5726	YMR287C
A_06_P5727	YMR288W
A_06_P5728	YMR289W
A_06_P5729	YMR290C
A_06_P5730	YMR290W-A
A_06_P5731	YMR291W
A_06_P5732	YMR292W
A_06_P5733	YMR293C
A_06_P5734	YMR294W
A_06_P5735	YMR294W-A
A_06_P5736	YMR295C
A_06_P5737	YMR296C
A_06_P5738	YMR297W
A_06_P5739	YMR298W
A_06_P5740	YMR299C
A_06_P5741	YMR300C
A_06_P5742	YMR301C
A_06_P5743	YMR302C
A_06_P5744	YMR303C
A_06_P5745	YMR304C-A
A_06_P5746	YMR304W
A_06_P5747	YMR305C
A_06_P5748	YMR306C-A
A_06_P5749	YMR306W
A_06_P5750	YMR307W
A_06_P5751	YMR308C
A_06_P5752	YMR309C
A_06_P5753	YMR310C
A_06_P5754	YMR311C
A_06_P5755	YMR312W
A_06_P5756	YMR313C
A_06_P5757	YMR314W
A_06_P5758	YMR315W
A_06_P5759	YMR316C-A
A_06_P5760	YMR316C-B
A_06_P5761	YMR316W
A_06_P5762	YMR317W
A_06_P5763	YMR318C
A_06_P5764	YMR319C
A_06_P5765	YMR320W
A_06_P5766	YMR321C
A_06_P5767	YMR322C
A_06_P5768	YMR323W
A_06_P5769	YMR324C
A_06_P5770	YMR325W
A_06_P5771	YMR326C
A_06_P5772	YNL001W
A_06_P5773	YNL002C
A_06_P5774	YNL003C
A_06_P5775	YNL004W
A_06_P5776	YNL005C
A_06_P5777	YNL006W
A_06_P5778	YNL007C
A_06_P5779	YNL008C
A_06_P5780	YNL009W

A_06_P5781	YNL010W
A_06_P5782	YNL011C
A_06_P5783	YNL012W
A_06_P5784	YNL013C
A_06_P5785	YNL014W
A_06_P5786	YNL015W
A_06_P5787	YNL016W
A_06_P5788	YNL017C
A_06_P5789	YNL018C
A_06_P5790	YNL019C
A_06_P5791	YNL020C
A_06_P5792	YNL021W
A_06_P5793	YNL022C
A_06_P5794	YNL023C
A_06_P5795	YNL024C
A_06_P5796	YNL025C
A_06_P5797	YNL026W
A_06_P5798	YNL027W
A_06_P5799	YNL028W
A_06_P5800	YNL029C
A_06_P5801	YNL030W
A_06_P5802	YNL031C
A_06_P5803	YNL032W
A_06_P5804	YNL033W
A_06_P5805	YNL034W
A_06_P5806	YNL035C
A_06_P5807	YNL036W
A_06_P5808	YNL037C
A_06_P5809	YNL038W
A_06_P5810	YNL039W
A_06_P5811	YNL040W
A_06_P5812	YNL041C
A_06_P5813	YNL042W
A_06_P5814	YNL043C
A_06_P5815	YNL044W
A_06_P5816	YNL045W
A_06_P5817	YNL046W
A_06_P5818	YNL047C
A_06_P5819	YNL048W
A_06_P5820	YNL049C
A_06_P5821	YNL050C
A_06_P5822	YNL051W
A_06_P5823	YNL052W
A_06_P5824	YNL053W
A_06_P5825	YNL054W
A_06_P5826	YNL055C
A_06_P5827	YNL056W
A_06_P5828	YNL057W
A_06_P5829	YNL058C
A_06_P5830	YNL059C
A_06_P5831	YNL061W
A_06_P5832	YNL062C
A_06_P5833	YNL063W
A_06_P5834	YNL064C
A_06_P5835	YNL065W

A_06_P5836	YNL066W
A_06_P5837	YNL067W
A_06_P5838	YNL067W-A
A_06_P5839	YNL068C
A_06_P5840	YNL069C
A_06_P5841	YNL070W
A_06_P5842	YNL071W
A_06_P5843	YNL072W
A_06_P5844	YNL073W
A_06_P5845	YNL074C
A_06_P5846	YNL075W
A_06_P5847	YNL076W
A_06_P5848	YNL077W
A_06_P5849	YNL078W
A_06_P5850	YNL079C
A_06_P5851	YNL080C
A_06_P5852	YNL081C
A_06_P5853	YNL082W
A_06_P5854	YNL083W
A_06_P5855	YNL084C
A_06_P5856	YNL085W
A_06_P5857	YNL086W
A_06_P5858	YNL087W
A_06_P5859	YNL088W
A_06_P5860	YNL089C
A_06_P5861	YNL090W
A_06_P5862	YNL091W
A_06_P5863	YNL092W
A_06_P5864	YNL093W
A_06_P5865	YNL094W
A_06_P5866	YNL095C
A_06_P5867	YNL096C
A_06_P5868	YNL097C
A_06_P5869	YNL098C
A_06_P5870	YNL099C
A_06_P5871	YNL100W
A_06_P5872	YNL101W
A_06_P5873	YNL102W
A_06_P5874	YNL103W
A_06_P5875	YNL104C
A_06_P5876	YNL105W
A_06_P5877	YNL106C
A_06_P5878	YNL107W
A_06_P5879	YNL108C
A_06_P5880	YNL109W
A_06_P5881	YNL110C
A_06_P5882	YNL111C
A_06_P5883	YNL112W
A_06_P5884	YNL113W
A_06_P5885	YNL114C
A_06_P5886	YNL115C
A_06_P5887	YNL116W
A_06_P5888	YNL117W
A_06_P5889	YNL118C
A_06_P5890	YNL119W

A_06_P5891	YNL120C
A_06_P5892	YNL121C
A_06_P5893	YNL122C
A_06_P5894	YNL123W
A_06_P5895	YNL124W
A_06_P5896	YNL125C
A_06_P5897	YNL126W
A_06_P5898	YNL127W
A_06_P5899	YNL128W
A_06_P5900	YNL129W
A_06_P5901	YNL130C
A_06_P5902	YNL131W
A_06_P5903	YNL132W
A_06_P5904	YNL133C
A_06_P5905	YNL134C
A_06_P5906	YNL135C
A_06_P5907	YNL136W
A_06_P5908	YNL137C
A_06_P5909	YNL138W
A_06_P5910	YNL139C
A_06_P5911	YNL140C
A_06_P5912	YNL141W
A_06_P5913	YNL142W
A_06_P5914	YNL143C
A_06_P5915	YNL144C
A_06_P5916	YNL145W
A_06_P5917	YNL146W
A_06_P5918	YNL147W
A_06_P5919	YNL148C
A_06_P5920	YNL149C
A_06_P5921	YNL150W
A_06_P5922	YNL151C
A_06_P5923	YNL152W
A_06_P5924	YNL153C
A_06_P5925	YNL154C
A_06_P5926	YNL155W
A_06_P5927	YNL156C
A_06_P5928	YNL157W
A_06_P5929	YNL158W
A_06_P5930	YNL159C
A_06_P5931	YNL160W
A_06_P5932	YNL161W
A_06_P5933	YNL162W
A_06_P5934	YNL162W-A
A_06_P5935	YNL163C
A_06_P5936	YNL164C
A_06_P5937	YNL165W
A_06_P5938	YNL166C
A_06_P5939	YNL167C
A_06_P5940	YNL168C
A_06_P5941	YNL169C
A_06_P5942	YNL170W
A_06_P5943	YNL171C
A_06_P5944	YNL172W
A_06_P5945	YNL173C

A_06_P5946	YNL174W
A_06_P5947	YNL175C
A_06_P5948	YNL176C
A_06_P5949	YNL177C
A_06_P5950	YNL178W
A_06_P5951	YNL179C
A_06_P5952	YNL180C
A_06_P5953	YNL181W
A_06_P5954	YNL182C
A_06_P5955	YNL183C
A_06_P5956	YNL184C
A_06_P5957	YNL185C
A_06_P5958	YNL186W
A_06_P5959	YNL187W
A_06_P5960	YNL188W
A_06_P5961	YNL189W
A_06_P5962	YNL190W
A_06_P5963	YNL191W
A_06_P5964	YNL192W
A_06_P5965	YNL193W
A_06_P5966	YNL194C
A_06_P5967	YNL195C
A_06_P5968	YNL196C
A_06_P5969	YNL197C
A_06_P5970	YNL198C
A_06_P5971	YNL199C
A_06_P5972	YNL200C
A_06_P5973	YNL201C
A_06_P5974	YNL202W
A_06_P5975	YNL203C
A_06_P5976	YNL204C
A_06_P5977	YNL205C
A_06_P5978	YNL206C
A_06_P5979	YNL207W
A_06_P5980	YNL208W
A_06_P5981	YNL209W
A_06_P5982	YNL210W
A_06_P5983	YNL211C
A_06_P5984	YNL212W
A_06_P5985	YNL213C
A_06_P5986	YNL214W
A_06_P5987	YNL215W
A_06_P5988	YNL216W
A_06_P5989	YNL217W
A_06_P5990	YNL218W
A_06_P5991	YNL219C
A_06_P5992	YNL220W
A_06_P5993	YNL221C
A_06_P5994	YNL222W
A_06_P5995	YNL223W
A_06_P5996	YNL224C
A_06_P5997	YNL225C
A_06_P5998	YNL226W
A_06_P5999	YNL227C
A_06_P6000	YNL228W

A_06_P6001	YNL229C
A_06_P6002	YNL230C
A_06_P6003	YNL231C
A_06_P6004	YNL232W
A_06_P6005	YNL233W
A_06_P6006	YNL234W
A_06_P6007	YNL235C
A_06_P6008	YNL236W
A_06_P6009	YNL237W
A_06_P6010	YNL238W
A_06_P6011	YNL239W
A_06_P6012	YNL240C
A_06_P6013	YNL241C
A_06_P6014	YNL242W
A_06_P6015	YNL243W
A_06_P6016	YNL244C
A_06_P6017	YNL245C
A_06_P6018	YNL246W
A_06_P6019	YNL247W
A_06_P6020	YNL248C
A_06_P6021	YNL249C
A_06_P6022	YNL250W
A_06_P6023	YNL251C
A_06_P6024	YNL252C
A_06_P6025	YNL253W
A_06_P6026	YNL254C
A_06_P6027	YNL255C
A_06_P6028	YNL256W
A_06_P6029	YNL257C
A_06_P6030	YNL258C
A_06_P6031	YNL259C
A_06_P6032	YNL260C
A_06_P6033	YNL261W
A_06_P6034	YNL262W
A_06_P6035	YNL263C
A_06_P6036	YNL264C
A_06_P6037	YNL265C
A_06_P6038	YNL266W
A_06_P6039	YNL267W
A_06_P6040	YNL268W
A_06_P6041	YNL269W
A_06_P6042	YNL270C
A_06_P6043	YNL271C
A_06_P6044	YNL272C
A_06_P6045	YNL273W
A_06_P6046	YNL274C
A_06_P6047	YNL275W
A_06_P6048	YNL276C
A_06_P6049	YNL277W
A_06_P6050	YNL278W
A_06_P6051	YNL279W
A_06_P6052	YNL280C
A_06_P6053	YNL281W
A_06_P6054	YNL282W
A_06_P6055	YNL283C

A_06_P6056	YNL284C
A_06_P6057	YNL285W
A_06_P6058	YNL286W
A_06_P6059	YNL287W
A_06_P6060	YNL288W
A_06_P6061	YNL289W
A_06_P6062	YNL290W
A_06_P6063	YNL291C
A_06_P6064	YNL292W
A_06_P6065	YNL293W
A_06_P6066	YNL294C
A_06_P6067	YNL295W
A_06_P6068	YNL296W
A_06_P6069	YNL297C
A_06_P6070	YNL298W
A_06_P6071	YNL299W
A_06_P6072	YNL300W
A_06_P6073	YNL301C
A_06_P6074	YNL302C
A_06_P6075	YNL303W
A_06_P6076	YNL304W
A_06_P6077	YNL305C
A_06_P6078	YNL306W
A_06_P6079	YNL307C
A_06_P6080	YNL308C
A_06_P6081	YNL309W
A_06_P6082	YNL310C
A_06_P6083	YNL311C
A_06_P6084	YNL312W
A_06_P6085	YNL313C
A_06_P6086	YNL314W
A_06_P6087	YNL315C
A_06_P6088	YNL316C
A_06_P6089	YNL317W
A_06_P6090	YNL318C
A_06_P6091	YNL319W
A_06_P6092	YNL320W
A_06_P6093	YNL321W
A_06_P6094	YNL322C
A_06_P6095	YNL323W
A_06_P6096	YNL324W
A_06_P6097	YNL325C
A_06_P6098	YNL326C
A_06_P6099	YNL327W
A_06_P6100	YNL328C
A_06_P6101	YNL329C
A_06_P6102	YNL330C
A_06_P6103	YNL331C
A_06_P6104	YNL332W
A_06_P6105	YNL333W
A_06_P6106	YNL334C
A_06_P6107	YNL335W
A_06_P6108	YNL336W
A_06_P6109	YNL337W
A_06_P6110	YNL339C

A_06_P6111	YNR001C
A_06_P6112	YNR001W-A
A_06_P6113	YNR002C
A_06_P6114	YNR003C
A_06_P6115	YNR004W
A_06_P6116	YNR005C
A_06_P6117	YNR006W
A_06_P6118	YNR007C
A_06_P6119	YNR008W
A_06_P6120	YNR009W
A_06_P6121	YNR010W
A_06_P6122	YNR011C
A_06_P6123	YNR012W
A_06_P6124	YNR013C
A_06_P6125	YNR014W
A_06_P6126	YNR015W
A_06_P6127	YNR016C
A_06_P6128	YNR017W
A_06_P6129	YNR018W
A_06_P6130	YNR019W
A_06_P6131	YNR020C
A_06_P6132	YNR021W
A_06_P6133	YNR022C
A_06_P6134	YNR023W
A_06_P6135	YNR024W
A_06_P6136	YNR025C
A_06_P6137	YNR026C
A_06_P6138	YNR027W
A_06_P6139	YNR028W
A_06_P6140	YNR029C
A_06_P6141	YNR030W
A_06_P6142	YNR031C
A_06_P6143	YNR032C-A
A_06_P6144	YNR032W
A_06_P6145	YNR033W
A_06_P6146	YNR034W
A_06_P6147	YNR034W-A
A_06_P6148	YNR035C
A_06_P6149	YNR036C
A_06_P6150	YNR037C
A_06_P6151	YNR038W
A_06_P6152	YNR039C
A_06_P6153	YNR040W
A_06_P6154	YNR041C
A_06_P6155	YNR042W
A_06_P6156	YNR043W
A_06_P6157	YNR044W
A_06_P6158	YNR045W
A_06_P6159	YNR046W
A_06_P6160	YNR047W
A_06_P6161	YNR048W
A_06_P6162	YNR049C
A_06_P6163	YNR050C
A_06_P6164	YNR051C
A_06_P6165	YNR052C

A_06_P6166	YNR053C
A_06_P6167	YNR054C
A_06_P6168	YNR055C
A_06_P6169	YNR056C
A_06_P6170	YNR057C
A_06_P6171	YNR058W
A_06_P6172	YNR059W
A_06_P6173	YNR060W
A_06_P6174	YNR061C
A_06_P6175	YNR062C
A_06_P6176	YNR063W
A_06_P6177	YNR064C
A_06_P6178	YNR065C
A_06_P6179	YNR066C
A_06_P6180	YNR067C
A_06_P6181	YNR068C
A_06_P6182	YNR069C
A_06_P6183	YNR070W
A_06_P6184	YNR071C
A_06_P6185	YNR072W
A_06_P6186	YNR073C
A_06_P6187	YNR074C
A_06_P6188	YNR075W
A_06_P6189	YNR076W
A_06_P6190	YNR077C
A_06_P6191	YOL001W
A_06_P6192	YOL002C
A_06_P6193	YOL003C
A_06_P6194	YOL004W
A_06_P6195	YOL005C
A_06_P6196	YOL006C
A_06_P6197	YOL007C
A_06_P6198	YOL008W
A_06_P6199	YOL009C
A_06_P6200	YOL010W
A_06_P6201	YOL011W
A_06_P6202	YOL012C
A_06_P6203	YOL013C
A_06_P6204	YOL013W-A
A_06_P6205	YOL014W
A_06_P6206	YOL015W
A_06_P6207	YOL016C
A_06_P6208	YOL017W
A_06_P6209	YOL018C
A_06_P6210	YOL019W
A_06_P6211	YOL020W
A_06_P6212	YOL021C
A_06_P6213	YOL022C
A_06_P6214	YOL023W
A_06_P6215	YOL024W
A_06_P6216	YOL025W
A_06_P6217	YOL026C
A_06_P6218	YOL027C
A_06_P6219	YOL028C
A_06_P6220	YOL029C

A_06_P6221	YOL030W
A_06_P6222	YOL031C
A_06_P6223	YOL032W
A_06_P6224	YOL033W
A_06_P6225	YOL034W
A_06_P6226	YOL035C
A_06_P6227	YOL036W
A_06_P6228	YOL037C
A_06_P6229	YOL038W
A_06_P6230	YOL039W
A_06_P6231	YOL040C
A_06_P6232	YOL041C
A_06_P6233	YOL042W
A_06_P6234	YOL043C
A_06_P6235	YOL044W
A_06_P6236	YOL045W
A_06_P6237	YOL046C
A_06_P6238	YOL047C
A_06_P6239	YOL048C
A_06_P6240	YOL049W
A_06_P6241	YOL050C
A_06_P6242	YOL051W
A_06_P6243	YOL052C
A_06_P6244	YOL052C-A
A_06_P6245	YOL053W
A_06_P6246	YOL054W
A_06_P6247	YOL055C
A_06_P6248	YOL056W
A_06_P6249	YOL057W
A_06_P6250	YOL058W
A_06_P6251	YOL059W
A_06_P6252	YOL060C
A_06_P6253	YOL061W
A_06_P6254	YOL062C
A_06_P6255	YOL063C
A_06_P6256	YOL064C
A_06_P6257	YOL065C
A_06_P6258	YOL066C
A_06_P6259	YOL067C
A_06_P6260	YOL068C
A_06_P6261	YOL069W
A_06_P6262	YOL070C
A_06_P6263	YOL071W
A_06_P6264	YOL072W
A_06_P6265	YOL073C
A_06_P6266	YOL075C
A_06_P6267	YOL076W
A_06_P6268	YOL077C
A_06_P6269	YOL077W-A
A_06_P6270	YOL078W
A_06_P6271	YOL079W
A_06_P6272	YOL080C
A_06_P6273	YOL081W
A_06_P6274	YOL082W
A_06_P6275	YOL083W

A_06_P6276	YOL084W
A_06_P6277	YOL085C
A_06_P6278	YOL086C
A_06_P6279	YOL086W-A
A_06_P6280	YOL087C
A_06_P6281	YOL088C
A_06_P6282	YOL089C
A_06_P6283	YOL090W
A_06_P6284	YOL091W
A_06_P6285	YOL092W
A_06_P6286	YOL093W
A_06_P6287	YOL094C
A_06_P6288	YOL095C
A_06_P6289	YOL096C
A_06_P6290	YOL097C
A_06_P6291	YOL098C
A_06_P6292	YOL099C
A_06_P6293	YOL100W
A_06_P6294	YOL101C
A_06_P6295	YOL102C
A_06_P6296	YOL103W
A_06_P6297	YOL104C
A_06_P6298	YOL105C
A_06_P6299	YOL106W
A_06_P6300	YOL107W
A_06_P6301	YOL108C
A_06_P6302	YOL109W
A_06_P6303	YOL110W
A_06_P6304	YOL111C
A_06_P6305	YOL112W
A_06_P6306	YOL113W
A_06_P6307	YOL114C
A_06_P6308	YOL115W
A_06_P6309	YOL116W
A_06_P6310	YOL117W
A_06_P6311	YOL118C
A_06_P6312	YOL119C
A_06_P6313	YOL120C
A_06_P6314	YOL121C
A_06_P6315	YOL122C
A_06_P6316	YOL123W
A_06_P6317	YOL124C
A_06_P6318	YOL125W
A_06_P6319	YOL126C
A_06_P6320	YOL127W
A_06_P6321	YOL128C
A_06_P6322	YOL129W
A_06_P6323	YOL130W
A_06_P6324	YOL131W
A_06_P6325	YOL132W
A_06_P6326	YOL133W
A_06_P6327	YOL134C
A_06_P6328	YOL135C
A_06_P6329	YOL136C
A_06_P6330	YOL137W

A_06_P6331	YOL138C
A_06_P6332	YOL139C
A_06_P6333	YOL140W
A_06_P6334	YOL141W
A_06_P6335	YOL142W
A_06_P6336	YOL143C
A_06_P6337	YOL144W
A_06_P6338	YOL145C
A_06_P6339	YOL146W
A_06_P6340	YOL147C
A_06_P6341	YOL148C
A_06_P6342	YOL149W
A_06_P6343	YOL150C
A_06_P6344	YOL151W
A_06_P6345	YOL152W
A_06_P6346	YOL153C
A_06_P6347	YOL154W
A_06_P6348	YOL155C
A_06_P6349	YOL156W
A_06_P6350	YOL157C
A_06_P6351	YOL158C
A_06_P6352	YOL159C
A_06_P6353	YOL159C-A
A_06_P6354	YOL160W
A_06_P6355	YOL161C
A_06_P6356	YOL162W
A_06_P6357	YOL163W
A_06_P6358	YOL164W
A_06_P6359	YOL165C
A_06_P6360	YOL166C
A_06_P6361	YOR001W
A_06_P6362	YOR002W
A_06_P6363	YOR003W
A_06_P6364	YOR004W
A_06_P6365	YOR005C
A_06_P6366	YOR006C
A_06_P6367	YOR007C
A_06_P6368	YOR008C
A_06_P6369	YOR008C-A
A_06_P6370	YOR008W-B
A_06_P6371	YOR009W
A_06_P6372	YOR010C
A_06_P6373	YOR011W
A_06_P6374	YOR012W
A_06_P6375	YOR013W
A_06_P6376	YOR014W
A_06_P6377	YOR015W
A_06_P6378	YOR016C
A_06_P6379	YOR017W
A_06_P6380	YOR018W
A_06_P6381	YOR019W
A_06_P6382	YOR020C
A_06_P6383	YOR021C
A_06_P6384	YOR022C
A_06_P6385	YOR023C

A_06_P6386	YOR024W
A_06_P6387	YOR025W
A_06_P6388	YOR026W
A_06_P6389	YOR027W
A_06_P6390	YOR028C
A_06_P6391	YOR029W
A_06_P6392	YOR030W
A_06_P6393	YOR031W
A_06_P6394	YOR032C
A_06_P6395	YOR033C
A_06_P6396	YOR034C
A_06_P6397	YOR035C
A_06_P6398	YOR036W
A_06_P6399	YOR037W
A_06_P6400	YOR038C
A_06_P6401	YOR039W
A_06_P6402	YOR040W
A_06_P6403	YOR041C
A_06_P6404	YOR042W
A_06_P6405	YOR043W
A_06_P6406	YOR044W
A_06_P6407	YOR045W
A_06_P6408	YOR046C
A_06_P6409	YOR047C
A_06_P6410	YOR048C
A_06_P6411	YOR049C
A_06_P6412	YOR050C
A_06_P6413	YOR051C
A_06_P6414	YOR052C
A_06_P6415	YOR053W
A_06_P6416	YOR054C
A_06_P6417	YOR055W
A_06_P6418	YOR056C
A_06_P6419	YOR057W
A_06_P6420	YOR058C
A_06_P6421	YOR059C
A_06_P6422	YOR060C
A_06_P6423	YOR061W
A_06_P6424	YOR062C
A_06_P6425	YOR063W
A_06_P6426	YOR064C
A_06_P6427	YOR065W
A_06_P6428	YOR066W
A_06_P6429	YOR067C
A_06_P6430	YOR068C
A_06_P6431	YOR069W
A_06_P6432	YOR070C
A_06_P6433	YOR071C
A_06_P6434	YOR072W
A_06_P6435	YOR073W
A_06_P6436	YOR074C
A_06_P6437	YOR075W
A_06_P6438	YOR076C
A_06_P6439	YOR077W
A_06_P6440	YOR078W

A_06_P6441	YOR079C
A_06_P6442	YOR080W
A_06_P6443	YOR081C
A_06_P6444	YOR082C
A_06_P6445	YOR083W
A_06_P6446	YOR084W
A_06_P6447	YOR085W
A_06_P6448	YOR086C
A_06_P6449	YOR087W
A_06_P6450	YOR087W
A_06_P6451	YOR089C
A_06_P6452	YOR090C
A_06_P6453	YOR091W
A_06_P6454	YOR092W
A_06_P6455	YOR093C
A_06_P6456	YOR094W
A_06_P6457	YOR095C
A_06_P6458	YOR096W
A_06_P6459	YOR097C
A_06_P6460	YOR098C
A_06_P6461	YOR099W
A_06_P6462	YOR100C
A_06_P6463	YOR101W
A_06_P6464	YOR102W
A_06_P6465	YOR103C
A_06_P6466	YOR104W
A_06_P6467	YOR105W
A_06_P6468	YOR106W
A_06_P6469	YOR107W
A_06_P6470	YOR108W
A_06_P6471	YOR109W
A_06_P6472	YOR110W
A_06_P6473	YOR111W
A_06_P6474	YOR112W
A_06_P6475	YOR113W
A_06_P6476	YOR114W
A_06_P6477	YOR115C
A_06_P6478	YOR116C
A_06_P6479	YOR117W
A_06_P6480	YOR118W
A_06_P6481	YOR119C
A_06_P6482	YOR120W
A_06_P6483	YOR121C
A_06_P6484	YOR122C
A_06_P6485	YOR123C
A_06_P6486	YOR124C
A_06_P6487	YOR125C
A_06_P6488	YOR126C
A_06_P6489	YOR127W
A_06_P6490	YOR128C
A_06_P6491	YOR129C
A_06_P6492	YOR130C
A_06_P6493	YOR131C
A_06_P6494	YOR132W
A_06_P6495	YOR133W

A_06_P6496	YOR134W
A_06_P6497	YOR135C
A_06_P6498	YOR136W
A_06_P6499	YOR137C
A_06_P6500	YOR138C
A_06_P6501	YOR139C
A_06_P6502	YOR140W
A_06_P6503	YOR141C
A_06_P6504	YOR142W
A_06_P6505	YOR143C
A_06_P6506	YOR144C
A_06_P6507	YOR145C
A_06_P6508	YOR146W
A_06_P6509	YOR147W
A_06_P6510	YOR148C
A_06_P6511	YOR149C
A_06_P6512	YOR150W
A_06_P6513	YOR151C
A_06_P6514	YOR152C
A_06_P6515	YOR153W
A_06_P6516	YOR154W
A_06_P6517	YOR155C
A_06_P6518	YOR156C
A_06_P6519	YOR157C
A_06_P6520	YOR158W
A_06_P6521	YOR159C
A_06_P6522	YOR160W
A_06_P6523	YOR161C
A_06_P6524	YOR162C
A_06_P6525	YOR163W
A_06_P6526	YOR164C
A_06_P6527	YOR165W
A_06_P6528	YOR166C
A_06_P6529	YOR167C
A_06_P6530	YOR168W
A_06_P6531	YOR169C
A_06_P6532	YOR170W
A_06_P6533	YOR171C
A_06_P6534	YOR172W
A_06_P6535	YOR173W
A_06_P6536	YOR174W
A_06_P6537	YOR175C
A_06_P6538	YOR176W
A_06_P6539	YOR177C
A_06_P6540	YOR178C
A_06_P6541	YOR179C
A_06_P6542	YOR180C
A_06_P6543	YOR181W
A_06_P6544	YOR182C
A_06_P6545	YOR183W
A_06_P6546	YOR184W
A_06_P6547	YOR185C
A_06_P6548	YOR186W
A_06_P6549	YOR187W
A_06_P6550	YOR188W

A_06_P6551	YOR189W
A_06_P6552	YOR190W
A_06_P6553	YOR191W
A_06_P6554	YOR192C
A_06_P6555	YOR193W
A_06_P6556	YOR194C
A_06_P6557	YOR195W
A_06_P6558	YOR196C
A_06_P6559	YOR197W
A_06_P6560	YOR198C
A_06_P6561	YOR199W
A_06_P6562	YOR200W
A_06_P6563	YOR201C
A_06_P6564	YOR202W
A_06_P6565	YOR203W
A_06_P6566	YOR204W
A_06_P6567	YOR205C
A_06_P6568	YOR206W
A_06_P6569	YOR207C
A_06_P6570	YOR208W
A_06_P6571	YOR209C
A_06_P6572	YOR210W
A_06_P6573	YOR211C
A_06_P6574	YOR212W
A_06_P6575	YOR213C
A_06_P6576	YOR214C
A_06_P6577	YOR215C
A_06_P6578	YOR216C
A_06_P6579	YOR217W
A_06_P6580	YOR218C
A_06_P6581	YOR219C
A_06_P6582	YOR220W
A_06_P6583	YOR221C
A_06_P6584	YOR222W
A_06_P6585	YOR223W
A_06_P6586	YOR224C
A_06_P6587	YOR225W
A_06_P6588	YOR226C
A_06_P6589	YOR227W
A_06_P6590	YOR228C
A_06_P6591	YOR229W
A_06_P6592	YOR230W
A_06_P6593	YOR231W
A_06_P6594	YOR232W
A_06_P6595	YOR233W
A_06_P6596	YOR234C
A_06_P6597	YOR235W
A_06_P6598	YOR236W
A_06_P6599	YOR237W
A_06_P6600	YOR238W
A_06_P6601	YOR239W
A_06_P6602	YOR241W
A_06_P6603	YOR242C
A_06_P6604	YOR243C
A_06_P6605	YOR244W

A_06_P6606	YOR245C
A_06_P6607	YOR246C
A_06_P6608	YOR247W
A_06_P6609	YOR248W
A_06_P6610	YOR249C
A_06_P6611	YOR250C
A_06_P6612	YOR251C
A_06_P6613	YOR252W
A_06_P6614	YOR253W
A_06_P6615	YOR254C
A_06_P6616	YOR255W
A_06_P6617	YOR256C
A_06_P6618	YOR257W
A_06_P6619	YOR258W
A_06_P6620	YOR259C
A_06_P6621	YOR260W
A_06_P6622	YOR261C
A_06_P6623	YOR262W
A_06_P6624	YOR263C
A_06_P6625	YOR264W
A_06_P6626	YOR265W
A_06_P6627	YOR266W
A_06_P6628	YOR267C
A_06_P6629	YOR268C
A_06_P6630	YOR269W
A_06_P6631	YOR270C
A_06_P6632	YOR271C
A_06_P6633	YOR272W
A_06_P6634	YOR273C
A_06_P6635	YOR274W
A_06_P6636	YOR275C
A_06_P6637	YOR276W
A_06_P6638	YOR277C
A_06_P6639	YOR278W
A_06_P6640	YOR279C
A_06_P6641	YOR280C
A_06_P6642	YOR281C
A_06_P6643	YOR282W
A_06_P6644	YOR283W
A_06_P6645	YOR284W
A_06_P6646	YOR285W
A_06_P6647	YOR286W
A_06_P6648	YOR287C
A_06_P6649	YOR288C
A_06_P6650	YOR289W
A_06_P6651	YOR290C
A_06_P6652	YOR291W
A_06_P6653	YOR292C
A_06_P6654	YOR293W
A_06_P6655	YOR294W
A_06_P6656	YOR295W
A_06_P6657	YOR296W
A_06_P6658	YOR297C
A_06_P6659	YOR298C-A
A_06_P6660	YOR298W

A_06_P6661	YOR299W
A_06_P6662	YOR300W
A_06_P6663	YOR301W
A_06_P6664	YOR302W
A_06_P6665	YOR303W
A_06_P6666	YOR304C-A
A_06_P6667	YOR304W
A_06_P6668	YOR305W
A_06_P6669	YOR306C
A_06_P6670	YOR307C
A_06_P6671	YOR308C
A_06_P6672	YOR309C
A_06_P6673	YOR310C
A_06_P6674	YOR311C
A_06_P6675	YOR312C
A_06_P6676	YOR313C
A_06_P6677	YOR314W
A_06_P6678	YOR314W-A
A_06_P6679	YOR315W
A_06_P6680	YOR316C
A_06_P6681	YOR317W
A_06_P6682	YOR318C
A_06_P6683	YOR319W
A_06_P6684	YOR320C
A_06_P6685	YOR321W
A_06_P6686	YOR322C
A_06_P6687	YOR323C
A_06_P6688	YOR324C
A_06_P6689	YOR325W
A_06_P6690	YOR326W
A_06_P6691	YOR327C
A_06_P6692	YOR328W
A_06_P6693	YOR329C
A_06_P6694	YOR330C
A_06_P6695	YOR331C
A_06_P6696	YOR332W
A_06_P6697	YOR333C
A_06_P6698	YOR334W
A_06_P6699	YOR335C
A_06_P6700	YOR336W
A_06_P6701	YOR337W
A_06_P6702	YOR338W
A_06_P6703	YOR339C
A_06_P6704	YOR340C
A_06_P6705	YOR341W
A_06_P6706	YOR342C
A_06_P6707	YOR343C
A_06_P6708	YOR344C
A_06_P6709	YOR345C
A_06_P6710	YOR346W
A_06_P6711	YOR347C
A_06_P6712	YOR348C
A_06_P6713	YOR349W
A_06_P6714	YOR350C
A_06_P6715	YOR351C

A_06_P6716	YOR352W
A_06_P6717	YOR353C
A_06_P6718	YOR354C
A_06_P6719	YOR355W
A_06_P6720	YOR356W
A_06_P6721	YOR357C
A_06_P6722	YOR358W
A_06_P6723	YOR359W
A_06_P6724	YOR360C
A_06_P6725	YOR361C
A_06_P6726	YOR362C
A_06_P6727	YOR363C
A_06_P6728	YOR364W
A_06_P6729	YOR365C
A_06_P6730	YOR366W
A_06_P6731	YOR367W
A_06_P6732	YOR368W
A_06_P6733	YOR369C
A_06_P6734	YOR370C
A_06_P6735	YOR371C
A_06_P6736	YOR372C
A_06_P6737	YOR373W
A_06_P6738	YOR374W
A_06_P6739	YOR375C
A_06_P6740	YOR376W
A_06_P6741	YOR377W
A_06_P6742	YOR378W
A_06_P6743	YOR379C
A_06_P6744	YOR380W
A_06_P6745	YOR381W
A_06_P6746	YOR382W
A_06_P6747	YOR383C
A_06_P6748	YOR384W
A_06_P6749	YOR385W
A_06_P6750	YOR386W
A_06_P6751	YOR387C
A_06_P6752	YOR388C
A_06_P6753	YOR389W
A_06_P6754	YOR390W
A_06_P6755	YOR391C
A_06_P6756	YOR392W
A_06_P6757	YOR393W
A_06_P6758	YOR394W
A_06_P6759	YOR396W
A_06_P6760	YPL001W
A_06_P6761	YPL002C
A_06_P6762	YPL003W
A_06_P6763	YPL004C
A_06_P6764	YPL005W
A_06_P6765	YPL006W
A_06_P6766	YPL007C
A_06_P6767	YPL008W
A_06_P6768	YPL009C
A_06_P6769	YPL010W
A_06_P6770	YPL011C

A_06_P6771	YPL012W
A_06_P6772	YPL013C
A_06_P6773	YPL014W
A_06_P6774	YPL015C
A_06_P6775	YPL016W
A_06_P6776	YPL017C
A_06_P6777	YPL018W
A_06_P6778	YPL019C
A_06_P6779	YPL020C
A_06_P6780	YPL021W
A_06_P6781	YPL022W
A_06_P6782	YPL023C
A_06_P6783	YPL024W
A_06_P6784	YPL025C
A_06_P6785	YPL026C
A_06_P6786	YPL027W
A_06_P6787	YPL028W
A_06_P6788	YPL029W
A_06_P6789	YPL030W
A_06_P6790	YPL031C
A_06_P6791	YPL032C
A_06_P6792	YPL033C
A_06_P6793	YPL034W
A_06_P6794	YPL035C
A_06_P6795	YPL036W
A_06_P6796	YPL037C
A_06_P6797	YPL038W
A_06_P6798	YPL039W
A_06_P6799	YPL040C
A_06_P6800	YPL041C
A_06_P6801	YPL042C
A_06_P6802	YPL043W
A_06_P6803	YPL044C
A_06_P6804	YPL045W
A_06_P6805	YPL046C
A_06_P6806	YPL047W
A_06_P6807	YPL048W
A_06_P6808	YPL049C
A_06_P6809	YPL050C
A_06_P6810	YPL051W
A_06_P6811	YPL052W
A_06_P6812	YPL053C
A_06_P6813	YPL054W
A_06_P6814	YPL055C
A_06_P6815	YPL056C
A_06_P6816	YPL057C
A_06_P6817	YPL058C
A_06_P6818	YPL059W
A_06_P6819	YPL060W
A_06_P6820	YPL061W
A_06_P6821	YPL062W
A_06_P6822	YPL063W
A_06_P6823	YPL064C
A_06_P6824	YPL065W
A_06_P6825	YPL066W

A_06_P6826	YPL067C
A_06_P6827	YPL068C
A_06_P6828	YPL069C
A_06_P6829	YPL070W
A_06_P6830	YPL071C
A_06_P6831	YPL072W
A_06_P6832	YPL073C
A_06_P6833	YPL074W
A_06_P6834	YPL075W
A_06_P6835	YPL076W
A_06_P6836	YPL077C
A_06_P6837	YPL078C
A_06_P6838	YPL079W
A_06_P6839	YPL080C
A_06_P6840	YPL081W
A_06_P6841	YPL082C
A_06_P6842	YPL083C
A_06_P6843	YPL084W
A_06_P6844	YPL085W
A_06_P6845	YPL086C
A_06_P6846	YPL087W
A_06_P6847	YPL088W
A_06_P6848	YPL089C
A_06_P6849	YPL090C
A_06_P6850	YPL091W
A_06_P6851	YPL092W
A_06_P6852	YPL093W
A_06_P6853	YPL094C
A_06_P6854	YPL095C
A_06_P6855	YPL096W
A_06_P6856	YPL097W
A_06_P6857	YPL098C
A_06_P6858	YPL099C
A_06_P6859	YPL100W
A_06_P6860	YPL101W
A_06_P6861	YPL102C
A_06_P6862	YPL103C
A_06_P6863	YPL104W
A_06_P6864	YPL105C
A_06_P6865	YPL106C
A_06_P6866	YPL107W
A_06_P6867	YPL108W
A_06_P6868	YPL109C
A_06_P6869	YPL110C
A_06_P6870	YPL111W
A_06_P6871	YPL112C
A_06_P6872	YPL113C
A_06_P6873	YPL114W
A_06_P6874	YPL115C
A_06_P6875	YPL116W
A_06_P6876	YPL117C
A_06_P6877	YPL118W
A_06_P6878	YPL119C
A_06_P6879	YPL120W
A_06_P6880	YPL121C

A_06_P6881	YPL122C
A_06_P6882	YPL123C
A_06_P6883	YPL124W
A_06_P6884	YPL125W
A_06_P6885	YPL126W
A_06_P6886	YPL127C
A_06_P6887	YPL128C
A_06_P6888	YPL129W
A_06_P6889	YPL130W
A_06_P6890	YPL131W
A_06_P6891	YPL132W
A_06_P6892	YPL133C
A_06_P6893	YPL134C
A_06_P6894	YPL135W
A_06_P6895	YPL136W
A_06_P6896	YPL137C
A_06_P6897	YPL138C
A_06_P6898	YPL139C
A_06_P6899	YPL140C
A_06_P6900	YPL141C
A_06_P6901	YPL142C
A_06_P6902	YPL143W
A_06_P6903	YPL144W
A_06_P6904	YPL145C
A_06_P6905	YPL146C
A_06_P6906	YPL147W
A_06_P6907	YPL148C
A_06_P6908	YPL149W
A_06_P6909	YPL150W
A_06_P6910	YPL151C
A_06_P6911	YPL152W
A_06_P6912	YPL153C
A_06_P6913	YPL154C
A_06_P6914	YPL155C
A_06_P6915	YPL156C
A_06_P6916	YPL157W
A_06_P6917	YPL158C
A_06_P6918	YPL159C
A_06_P6919	YPL160W
A_06_P6920	YPL161C
A_06_P6921	YPL162C
A_06_P6922	YPL163C
A_06_P6923	YPL164C
A_06_P6924	YPL165C
A_06_P6925	YPL166W
A_06_P6926	YPL167C
A_06_P6927	YPL168W
A_06_P6928	YPL169C
A_06_P6929	YPL170W
A_06_P6930	YPL171C
A_06_P6931	YPL172C
A_06_P6932	YPL173W
A_06_P6933	YPL174C
A_06_P6934	YPL175W
A_06_P6935	YPL176C

A_06_P6936	YPL177C
A_06_P6937	YPL178W
A_06_P6938	YPL179W
A_06_P6939	YPL180W
A_06_P6940	YPL181W
A_06_P6941	YPL182C
A_06_P6942	YPL183C
A_06_P6943	YPL183W-A
A_06_P6944	YPL184C
A_06_P6945	YPL185W
A_06_P6946	YPL186C
A_06_P6947	YPL187W
A_06_P6948	YPL188W
A_06_P6949	YPL189W
A_06_P6950	YPL190C
A_06_P6951	YPL191C
A_06_P6952	YPL192C
A_06_P6953	YPL193W
A_06_P6954	YPL194W
A_06_P6955	YPL195W
A_06_P6956	YPL196W
A_06_P6957	YPL197C
A_06_P6958	YPL198W
A_06_P6959	YPL199C
A_06_P6960	YPL200W
A_06_P6961	YPL201C
A_06_P6962	YPL202C
A_06_P6963	YPL203W
A_06_P6964	YPL204W
A_06_P6965	YPL205C
A_06_P6966	YPL206C
A_06_P6967	YPL207W
A_06_P6968	YPL208W
A_06_P6969	YPL209C
A_06_P6970	YPL210C
A_06_P6971	YPL211W
A_06_P6972	YPL212C
A_06_P6973	YPL213W
A_06_P6974	YPL214C
A_06_P6975	YPL215W
A_06_P6976	YPL216W
A_06_P6977	YPL217C
A_06_P6978	YPL218W
A_06_P6979	YPL219W
A_06_P6980	YPL220W
A_06_P6981	YPL221W
A_06_P6982	YPL222W
A_06_P6983	YPL223C
A_06_P6984	YPL224C
A_06_P6985	YPL225W
A_06_P6986	YPL226W
A_06_P6987	YPL227C
A_06_P6988	YPL228W
A_06_P6989	YPL229W
A_06_P6990	YPL230W

A_06_P6991	YPL231W
A_06_P6992	YPL232W
A_06_P6993	YPL233W
A_06_P6994	YPL234C
A_06_P6995	YPL235W
A_06_P6996	YPL236C
A_06_P6997	YPL237W
A_06_P6998	YPL238C
A_06_P6999	YPL239W
A_06_P7000	YPL240C
A_06_P7001	YPL241C
A_06_P7002	YPL242C
A_06_P7003	YPL243W
A_06_P7004	YPL244C
A_06_P7005	YPL245W
A_06_P7006	YPL246C
A_06_P7007	YPL247C
A_06_P7008	YPL248C
A_06_P7009	YPL249C
A_06_P7010	YPL249C-A
A_06_P7011	YPL250C
A_06_P7012	YPL251W
A_06_P7013	YPL252C
A_06_P7014	YPL253C
A_06_P7015	YPL254W
A_06_P7016	YPL255W
A_06_P7017	YPL256C
A_06_P7018	YPL257W
A_06_P7019	YPL258C
A_06_P7020	YPL259C
A_06_P7021	YPL260W
A_06_P7022	YPL261C
A_06_P7023	YPL262W
A_06_P7024	YPL263C
A_06_P7025	YPL264C
A_06_P7026	YPL265W
A_06_P7027	YPL266W
A_06_P7028	YPL267W
A_06_P7029	YPL268W
A_06_P7030	YPL269W
A_06_P7031	YPL270W
A_06_P7032	YPL271W
A_06_P7033	YPL272C
A_06_P7034	YPL273W
A_06_P7035	YPL274W
A_06_P7036	YPL275W
A_06_P7037	YPL276W
A_06_P7038	YPL277C
A_06_P7039	YPL278C
A_06_P7040	YPL279C
A_06_P7041	YPL280W
A_06_P7042	YPL281C
A_06_P7043	YPL282C
A_06_P7044	YPL283C
A_06_P7045	YPR001W

A_06_P7046	YPR002C-A
A_06_P7047	YPR002W
A_06_P7048	YPR003C
A_06_P7049	YPR004C
A_06_P7050	YPR005C
A_06_P7051	YPR006C
A_06_P7052	YPR007C
A_06_P7053	YPR008W
A_06_P7054	YPR009W
A_06_P7055	YPR010C
A_06_P7056	YPR011C
A_06_P7057	YPR012W
A_06_P7058	YPR013C
A_06_P7059	YPR014C
A_06_P7060	YPR015C
A_06_P7061	YPR016C
A_06_P7062	YPR016W-A
A_06_P7063	YPR017C
A_06_P7064	YPR018W
A_06_P7065	YPR019W
A_06_P7066	YPR020W
A_06_P7067	YPR021C
A_06_P7068	YPR022C
A_06_P7069	YPR023C
A_06_P7070	YPR024W
A_06_P7071	YPR025C
A_06_P7072	YPR026W
A_06_P7073	YPR027C
A_06_P7074	YPR028W
A_06_P7075	YPR029C
A_06_P7076	YPR030W
A_06_P7077	YPR031W
A_06_P7078	YPR032W
A_06_P7079	YPR033C
A_06_P7080	YPR034W
A_06_P7081	YPR035W
A_06_P7082	YPR036W
A_06_P7083	YPR037C
A_06_P7084	YPR038W
A_06_P7085	YPR039W
A_06_P7086	YPR040W
A_06_P7087	YPR041W
A_06_P7088	YPR042C
A_06_P7089	YPR043W
A_06_P7090	YPR044C
A_06_P7091	YPR045C
A_06_P7092	YPR046W
A_06_P7093	YPR047W
A_06_P7094	YPR048W
A_06_P7095	YPR049C
A_06_P7096	YPR050C
A_06_P7097	YPR051W
A_06_P7098	YPR052C
A_06_P7099	YPR053C
A_06_P7100	YPR054W

A_06_P7101	YPR055W
A_06_P7102	YPR056W
A_06_P7103	YPR057W
A_06_P7104	YPR058W
A_06_P7105	YPR059C
A_06_P7106	YPR060C
A_06_P7107	YPR061C
A_06_P7108	YPR062W
A_06_P7109	YPR063C
A_06_P7110	YPR064W
A_06_P7111	YPR065W
A_06_P7112	YPR066W
A_06_P7113	YPR067W
A_06_P7114	YPR068C
A_06_P7115	YPR069C
A_06_P7116	YPR070W
A_06_P7117	YPR071W
A_06_P7118	YPR072W
A_06_P7119	YPR073C
A_06_P7120	YPR074C
A_06_P7121	YPR074W-A
A_06_P7122	YPR075C
A_06_P7123	YPR076W
A_06_P7124	YPR077C
A_06_P7125	YPR078C
A_06_P7126	YPR079W
A_06_P7127	YPR080W
A_06_P7128	YPR081C
A_06_P7129	YPR082C
A_06_P7130	YPR083W
A_06_P7131	YPR084W
A_06_P7132	YPR085C
A_06_P7133	YPR086W
A_06_P7134	YPR087W
A_06_P7135	YPR088C
A_06_P7136	YPR089W
A_06_P7137	YPR089W
A_06_P7138	YPR091C
A_06_P7139	YPR092W
A_06_P7140	YPR093C
A_06_P7141	YPR094W
A_06_P7142	YPR095C
A_06_P7143	YPR096C
A_06_P7144	YPR097W
A_06_P7145	YPR098C
A_06_P7146	YPR099C
A_06_P7147	YPR100W
A_06_P7148	YPR101W
A_06_P7149	YPR102C
A_06_P7150	YPR103W
A_06_P7151	YPR104C
A_06_P7152	YPR105C
A_06_P7153	YPR106W
A_06_P7154	YPR107C
A_06_P7155	YPR108W

A_06_P7156	YPR109W
A_06_P7157	YPR110C
A_06_P7158	YPR111W
A_06_P7159	YPR112C
A_06_P7160	YPR113W
A_06_P7161	YPR114W
A_06_P7162	YPR115W
A_06_P7163	YPR116W
A_06_P7164	YPR117W
A_06_P7165	YPR118W
A_06_P7166	YPR119W
A_06_P7167	YPR120C
A_06_P7168	YPR121W
A_06_P7169	YPR122W
A_06_P7170	YPR123C
A_06_P7171	YPR124W
A_06_P7172	YPR125W
A_06_P7173	YPR126C
A_06_P7174	YPR127W
A_06_P7175	YPR128C
A_06_P7176	YPR129W
A_06_P7177	YPR130C
A_06_P7178	YPR131C
A_06_P7179	YPR132W
A_06_P7180	YPR133C
A_06_P7181	YPR133W-A
A_06_P7182	YPR134W
A_06_P7183	YPR135W
A_06_P7184	YPR136C
A_06_P7185	YPR137W
A_06_P7186	YPR138C
A_06_P7187	YPR139C
A_06_P7188	YPR140W
A_06_P7189	YPR141C
A_06_P7190	YPR142C
A_06_P7191	YPR143W
A_06_P7192	YPR144C
A_06_P7193	YPR145W
A_06_P7194	YPR146C
A_06_P7195	YPR147C
A_06_P7196	YPR148C
A_06_P7197	YPR149W
A_06_P7198	YPR150W
A_06_P7199	YPR151C
A_06_P7200	YPR152C
A_06_P7201	YPR153W
A_06_P7202	YPR154W
A_06_P7203	YPR155C
A_06_P7204	YPR156C
A_06_P7205	YPR157W
A_06_P7206	YPR158W
A_06_P7207	YPR159W
A_06_P7208	YPR160W
A_06_P7209	YPR161C
A_06_P7210	YPR162C

A_06_P7211	YPR163C
A_06_P7212	YPR164W
A_06_P7213	YPR165W
A_06_P7214	YPR166C
A_06_P7215	YPR167C
A_06_P7216	YPR168W
A_06_P7217	YPR169W
A_06_P7218	YPR170C
A_06_P7219	YPR171W
A_06_P7220	YPR172W
A_06_P7221	YPR173C
A_06_P7222	YPR174C
A_06_P7223	YPR175W
A_06_P7224	YPR176C
A_06_P7225	YPR177C
A_06_P7226	YPR178W
A_06_P7227	YPR179C
A_06_P7228	YPR180W
A_06_P7229	YPR181C
A_06_P7230	YPR182W
A_06_P7231	YPR183W
A_06_P7232	YPR184W
A_06_P7233	YPR185W
A_06_P7234	YPR186C
A_06_P7235	YPR187W
A_06_P7236	YPR188C
A_06_P7237	YPR189W
A_06_P7238	YPR190C
A_06_P7239	YPR191W
A_06_P7240	YPR192W
A_06_P7241	YPR193C
A_06_P7242	YPR194C
A_06_P7243	YPR195C
A_06_P7244	YPR196W
A_06_P7245	YPR197C
A_06_P7246	YPR198W
A_06_P7247	YPR199C
A_06_P7248	YPR200C
A_06_P7249	YPR201W
A_06_P7250	YPR202W
A_06_P7251	YPR203W
A_06_P7252	YPR204W
