# Supplementary material for: An efficient algorithm for the stochastic simulation of the hybridization of DNA to microarrays
Source: BMC Bioinformatics. 2009 Dec 10;10:411. doi: 10.1186/1471-2105-10-411 (PMC2805644; doi:10.1186/1471-2105-10-411)
Supplement: Additional file 4 — Arslan_Laurenzi_Supplemental. The average (and standard deviation) of the populations of each hybrid are provided in this worksheet, as calculated from the results provided in Supplemental Table 3. [file 1471-2105-10-411-S4.PDF]

This file lists the populations of full length cDNA molecules used in all simulations conducted with N = 1000 probe molecules per feature. The concentrations of each cDNA species may be calculated (in number of molecules/nL) by dividing these populations by the hybridization volume corresponding to this probe population (0.275 nL).

The sequences of these cDNA molecules may be obtained from the Saccharomyces Genome Database (www.yeastgenome.org); we have utilized the November 10, 2006 version of the yeast genome.

ORF Name	Population
YAL001C	258
YAL002W	53
YAL003W	43
YAL004W	13
YAL005C	152
YAL007C	28
YAL008W	79
YAL009W	97
YAL010C	26
YAL011W	26
YAL012W	6
YAL013W	97
YAL014C	22
YAL015C	121
YAL016C-A	226
YAL016C-B	28
YAL016W	58
YAL017W	22
YAL018C	100
YAL019W	2
YAL019W-A	39
YAL020C	16
YAL021C	54
YAL022C	220
YAL023C	78
YAL024C	178
YAL025C	99
YAL026C	23
YAL026C-A	96
YAL027W	6
YAL028W	236
YAL029C	102
YAL030W	44
YAL031C	13
YAL031W-A	30
YAL032C	31
YAL033W	12
YAL034C	22
YAL034C-B	36
YAL034W-A	41
YAL035W	76
YAL036C	40
YAL037C-A	14
YAL037C-B	23
YAL037W	77
YAL038W	26
YAL039C	53
YAL040C	12
YAL041W	40
YAL042C-A	742
YAL042W	117
YAL043C	232
YAL044C	27
YAL044W-A	7

YAL045C	418
YAL046C	65
YAL047C	13
YAL047W-A	20
YAL048C	22
YAL049C	8
YAL051W	11
YAL053W	400
YAL054C	2
YAL055W	25
YAL056C-A	41
YAL056W	116
YAL058W	155
YAL059C-A	7
YAL059W	56
YAL060W	29
YAL061W	13
YAL062W	37
YAL063C	11
YAL063C-A	22
YAL064C-A	3
YAL064W	24
YAL064W-B	37
YAL065C	71
YAL066W	106
YAL067C	32
YAL067W-A	44
YAL068C	32
YAL068W-A	49
YAL069W	52
YAR002C-A	80
YAR002W	124
YAR003W	43
YAR007C	187
YAR008W	103
YAR009C	30
YAR010C	93
YAR014C	228
YAR015W	226
YAR018C	36
YAR019C	58
YAR019W-A	55
YAR020C	138
YAR023C	8
YAR027W	28
YAR028W	9
YAR029W	112
YAR030C	41
YAR031W	46
YAR033W	8
YAR035C-A	17
YAR035W	13
YAR042W	29
YAR047C	15
YAR050W	36

YAR053W	21
YAR060C	35
YAR061W	60
YAR062W	114
YAR064W	10
YAR066W	66
YAR068W	17
YAR069C	50
YAR070C	266
YAR071W	38
YAR073W	89
YAR075W	180
YBL001C	276
YBL002W	10
YBL003C	7
YBL004W	8
YBL005W	23
YBL005W-A	55
YBL005W-B	27
YBL006C	9
YBL006W-A	16
YBL007C	117
YBL008W	38
YBL008W-A	83
YBL009W	159
YBL010C	345
YBL011W	19
YBL012C	117
YBL013W	11
YBL014C	35
YBL015W	4
YBL016W	28
YBL017C	13
YBL018C	19
YBL019W	30
YBL020W	54
YBL021C	31
YBL022C	10
YBL023C	3
YBL024W	66
YBL025W	39
YBL026W	6
YBL027W	26
YBL028C	129
YBL029C-A	265
YBL029W	9
YBL030C	101
YBL031W	47
YBL032W	66
YBL033C	35
YBL034C	31
YBL035C	49
YBL036C	25
YBL037W	409
YBL038W	27

YBL039C	110
YBL039C-A	40
YBL039W-B	9
YBL040C	4
YBL041W	20
YBL042C	27
YBL043W	62
YBL044W	72
YBL045C	562
YBL046W	33
YBL047C	61
YBL048W	27
YBL049W	15
YBL050W	7
YBL051C	70
YBL052C	33
YBL053W	23
YBL054W	21
YBL055C	180
YBL056W	52
YBL057C	18
YBL058W	56
YBL059C-A	5
YBL059W	166
YBL060W	21
YBL061C	7
YBL062W	35
YBL063W	552
YBL064C	870
YBL065W	112
YBL066C	101
YBL067C	27
YBL068W	162
YBL068W-A	86
YBL069W	39
YBL070C	94
YBL071C	9
YBL071C-B	10
YBL071W-A	68
YBL072C	11
YBL073W	12
YBL074C	147
YBL075C	35
YBL076C	98
YBL077W	188
YBL078C	22
YBL079W	128
YBL080C	70
YBL081W	494
YBL082C	2
YBL083C	36
YBL084C	53
YBL085W	218
YBL086C	154
YBL087C	4

YBL088C	52
YBL089W	3
YBL090W	42
YBL091C	125
YBL091C-A	216
YBL092W	72
YBL093C	13
YBL094C	69
YBL095W	385
YBL096C	46
YBL097W	10
YBL098W	4
YBL099W	196
YBL100C	41
YBL100W-A	162
YBL100W-B	91
YBL100W-C	26
YBL101C	39
YBL102W	291
YBL103C	94
YBL104C	10
YBL105C	286
YBL106C	135
YBL107C	121
YBL107W-A	5
YBL108C-A	56
YBL108W	54
YBL109W	100
YBL111C	70
YBL112C	44
YBL113C	8
YBL113W-A	99
YBR001C	40
YBR002C	74
YBR003W	43
YBR004C	50
YBR005W	21
YBR006W	53
YBR007C	53
YBR008C	67
YBR009C	40
YBR010W	9
YBR011C	188
YBR012C	6
YBR012W-A	61
YBR012W-B	11
YBR013C	39
YBR014C	27
YBR015C	264
YBR016W	19
YBR017C	104
YBR018C	54
YBR019C	218
YBR020W	70
YBR021W	292

YBR022W	5
YBR023C	53
YBR024W	4
YBR025C	88
YBR026C	18
YBR027C	11
YBR028C	23
YBR029C	8
YBR030W	101
YBR031W	12
YBR032W	251
YBR033W	194
YBR034C	10
YBR035C	19
YBR036C	36
YBR037C	76
YBR038W	142
YBR039W	393
YBR040W	49
YBR041W	274
YBR042C	20
YBR043C	82
YBR044C	251
YBR045C	171
YBR046C	7
YBR047W	63
YBR048W	15
YBR049C	25
YBR050C	11
YBR051W	82
YBR052C	18
YBR053C	21
YBR054W	81
YBR055C	447
YBR056C-B	149
YBR056W	5
YBR056W-A	55
YBR057C	4
YBR058C	30
YBR058C-A	68
YBR059C	3
YBR060C	23
YBR061C	16
YBR062C	21
YBR063C	55
YBR064W	20
YBR065C	197
YBR066C	20
YBR067C	84
YBR068C	146
YBR069C	19
YBR070C	5
YBR071W	94
YBR072C-A	13
YBR072W	98

YBR073W	8
YBR074W	88
YBR076C-A	116
YBR076W	81
YBR077C	15
YBR078W	106
YBR079C	320
YBR080C	4
YBR081C	174
YBR082C	25
YBR083W	10
YBR084C-A	4
YBR084W	5
YBR085C-A	37
YBR085W	17
YBR086C	667
YBR087W	11
YBR088C	147
YBR089C-A	151
YBR089W	31
YBR090C	173
YBR091C	41
YBR092C	73
YBR093C	6
YBR094W	131
YBR095C	40
YBR096W	29
YBR097W	73
YBR098W	36
YBR099C	30
YBR101C	66
YBR102C	13
YBR103C-A	23
YBR103W	28
YBR104W	41
YBR105C	193
YBR106W	39
YBR107C	41
YBR108W	85
YBR109C	62
YBR109W-A	208
YBR110W	55
YBR111C	39
YBR111W-A	27
YBR112C	67
YBR113W	835
YBR114W	56
YBR115C	22
YBR116C	77
YBR117C	31
YBR118W	15
YBR119W	5
YBR120C	20
YBR121C	142
YBR121C-A	51

YBR122C	29
YBR123C	10
YBR124W	21
YBR125C	51
YBR126C	238
YBR126W-A	28
YBR126W-B	51
YBR127C	636
YBR128C	110
YBR129C	17
YBR130C	20
YBR131C-A	52
YBR131W	39
YBR132C	148
YBR133C	147
YBR134W	47
YBR135W	589
YBR136W	56
YBR137W	110
YBR138C	41
YBR139W	26
YBR140C	23
YBR141C	12
YBR141W-A	17
YBR142W	119
YBR143C	51
YBR144C	51
YBR145W	14
YBR146W	27
YBR147W	17
YBR148W	13
YBR149W	43
YBR150C	78
YBR151W	412
YBR152W	26
YBR153W	402
YBR154C	61
YBR155W	33
YBR156C	14
YBR157C	1
YBR158W	47
YBR159W	62
YBR160W	180
YBR161W	39
YBR162C	13
YBR162W-A	41
YBR163W	19
YBR164C	11
YBR165W	35
YBR166C	70
YBR167C	20
YBR168W	47
YBR169C	52
YBR170C	55
YBR171W	14

YBR172C	14
YBR173C	195
YBR174C	407
YBR175W	29
YBR176W	174
YBR177C	39
YBR178W	44
YBR179C	353
YBR180W	27
YBR181C	22
YBR182C	57
YBR182C-A	19
YBR183W	8
YBR184W	5
YBR185C	264
YBR186W	21
YBR187W	164
YBR188C	53
YBR189W	138
YBR190W	63
YBR191W	18
YBR191W-A	22
YBR192W	143
YBR193C	29
YBR194W	394
YBR195C	29
YBR196C	30
YBR196C-A	32
YBR196C-B	41
YBR197C	75
YBR198C	36
YBR199W	67
YBR200W	64
YBR200W-A	100
YBR201C-A	24
YBR201W	110
YBR202W	13
YBR203W	13
YBR204C	19
YBR205W	6
YBR206W	41
YBR207W	15
YBR208C	200
YBR209W	217
YBR210W	13
YBR211C	65
YBR212W	59
YBR213W	170
YBR214W	15
YBR215W	100
YBR216C	87
YBR217W	5
YBR218C	576
YBR219C	13
YBR220C	17

YBR221C	164
YBR221W-A	39
YBR222C	10
YBR223C	34
YBR223W-A	91
YBR224W	6
YBR225W	92
YBR226C	14
YBR227C	34
YBR228W	14
YBR229C	11
YBR230C	16
YBR230W-A	19
YBR231C	80
YBR232C	8
YBR233W	30
YBR233W-A	46
YBR234C	3
YBR235W	38
YBR236C	5
YBR237W	408
YBR238C	9
YBR239C	8
YBR240C	22
YBR241C	64
YBR242W	151
YBR243C	68
YBR244W	46
YBR245C	24
YBR246W	430
YBR247C	176
YBR248C	111
YBR249C	43
YBR250W	22
YBR251W	26
YBR252W	25
YBR253W	5
YBR254C	17
YBR255C-A	26
YBR255W	39
YBR256C	85
YBR257W	40
YBR258C	164
YBR259W	29
YBR260C	200
YBR261C	12
YBR262C	159
YBR263W	159
YBR264C	81
YBR265W	26
YBR266C	64
YBR267W	157
YBR268W	27
YBR269C	14
YBR270C	12

YBR271W	22
YBR272C	29
YBR273C	125
YBR274W	41
YBR275C	57
YBR276C	170
YBR277C	102
YBR278W	231
YBR279W	720
YBR280C	25
YBR281C	39
YBR282W	142
YBR283C	7
YBR284W	33
YBR285W	106
YBR286W	66
YBR287W	24
YBR288C	105
YBR289W	4
YBR290W	29
YBR291C	107
YBR292C	20
YBR293W	36
YBR294W	4
YBR295W	101
YBR296C	746
YBR296C-A	23
YBR297W	276
YBR298C	58
YBR298C-A	12
YBR299W	39
YBR300C	52
YBR301W	26
YBR302C	12
YCL001W	451
YCL001W-A	3
YCL001W-B	62
YCL002C	33
YCL004W	30
YCL005W	41
YCL005W-A	2
YCL007C	36
YCL008C	67
YCL009C	11
YCL010C	17
YCL011C	26
YCL012C	12
YCL014W	29
YCL016C	5
YCL017C	58
YCL018W	43
YCL019W	170
YCL020W	29
YCL021W-A	79
YCL022C	64

YCL023C	13
YCL024W	16
YCL025C	181
YCL026C-A	40
YCL026C-B	56
YCL027W	158
YCL028W	27
YCL029C	741
YCL030C	35
YCL031C	170
YCL032W	23
YCL033C	205
YCL034W	97
YCL035C	303
YCL036W	137
YCL037C	33
YCL038C	288
YCL039W	63
YCL040W	12
YCL041C	26
YCL042W	50
YCL043C	124
YCL044C	23
YCL045C	10
YCL046W	688
YCL047C	110
YCL048W	183
YCL048W-A	172
YCL049C	32
YCL050C	28
YCL051W	13
YCL052C	365
YCL054W	5
YCL055W	23
YCL056C	36
YCL057C-A	85
YCL057W	246
YCL058C	22
YCL058W-A	26
YCL059C	16
YCL061C	404
YCL063W	169
YCL064C	22
YCL065W	164
YCL066W	398
YCL067C	257
YCL068C	16
YCL069W	5
YCL073C	67
YCL074W	88
YCL075W	229
YCL076W	57
YCR001W	18
YCR002C	264
YCR003W	21

YCR004C	38
YCR005C	10
YCR006C	58
YCR007C	29
YCR008W	43
YCR009C	46
YCR010C	53
YCR011C	62
YCR012W	23
YCR013C	7
YCR014C	45
YCR015C	293
YCR016W	317
YCR017C	3
YCR018C	51
YCR018C-A	46
YCR019W	325
YCR020C	38
YCR020C-A	10
YCR020W-B	9
YCR021C	33
YCR022C	14
YCR023C	34
YCR024C	62
YCR024C-A	23
YCR024C-B	756
YCR025C	45
YCR026C	31
YCR027C	26
YCR028C	42
YCR028C-A	36
YCR030C	159
YCR031C	13
YCR032W	104
YCR033W	160
YCR034W	251
YCR035C	125
YCR036W	33
YCR037C	197
YCR038C	235
YCR038W-A	21
YCR039C	11
YCR040W	299
YCR041W	53
YCR042C	37
YCR043C	7
YCR044C	240
YCR045C	225
YCR045W-A	345
YCR046C	6
YCR047C	8
YCR047W-A	16
YCR048W	5
YCR049C	11
YCR050C	21

YCR051W	103
YCR052W	17
YCR053W	11
YCR054C	43
YCR057C	23
YCR059C	18
YCR060W	19
YCR061W	22
YCR063W	5
YCR064C	107
YCR065W	108
YCR066W	455
YCR067C	78
YCR068W	49
YCR069W	72
YCR071C	56
YCR072C	8
YCR073C	150
YCR073W-A	24
YCR075C	27
YCR075W-A	259
YCR076C	24
YCR077C	28
YCR079W	26
YCR081C-A	298
YCR081W	90
YCR082W	35
YCR083W	80
YCR084C	2
YCR085W	18
YCR086W	36
YCR087C-A	13
YCR087W	16
YCR088W	4
YCR089W	34
YCR090C	96
YCR091W	88
YCR092C	416
YCR093W	34
YCR094W	61
YCR095C	21
YCR095W-A	146
YCR096C	51
YCR097W	51
YCR097W-A	30
YCR098C	40
YCR099C	19
YCR100C	131
YCR101C	294
YCR102C	157
YCR102W-A	153
YCR104W	96
YCR105W	72
YCR106W	18
YCR107W	87

YCR108C	24
YDL001W	13
YDL002C	160
YDL003W	5
YDL004W	8
YDL005C	72
YDL006W	30
YDL007C-A	18
YDL007W	17
YDL008W	119
YDL009C	38
YDL010W	229
YDL011C	29
YDL012C	4
YDL013W	3
YDL014W	9
YDL015C	37
YDL016C	26
YDL017W	75
YDL018C	16
YDL019C	10
YDL020C	269
YDL021W	32
YDL022C-A	94
YDL022W	4
YDL023C	53
YDL024C	198
YDL025C	36
YDL025W-A	154
YDL026W	7
YDL027C	15
YDL028C	31
YDL029W	59
YDL030W	70
YDL031W	25
YDL032W	90
YDL033C	93
YDL034W	97
YDL035C	90
YDL036C	131
YDL037C	60
YDL038C	17
YDL039C	20
YDL040C	57
YDL041W	173
YDL042C	62
YDL043C	108
YDL044C	34
YDL045C	30
YDL045W-A	49
YDL046W	61
YDL047W	250
YDL048C	3
YDL049C	25
YDL050C	101

YDL051W	714
YDL052C	37
YDL053C	20
YDL054C	69
YDL055C	67
YDL056W	215
YDL057W	9
YDL058W	21
YDL059C	222
YDL060W	566
YDL061C	41
YDL062W	5
YDL063C	38
YDL064W	228
YDL065C	61
YDL066W	8
YDL067C	18
YDL068W	53
YDL069C	7
YDL070W	13
YDL071C	135
YDL072C	42
YDL073W	11
YDL074C	149
YDL075W	8
YDL076C	23
YDL077C	54
YDL078C	331
YDL079C	6
YDL080C	5
YDL081C	16
YDL082W	63
YDL083C	33
YDL084W	12
YDL085C-A	71
YDL085W	90
YDL086C-A	47
YDL086W	21
YDL087C	39
YDL088C	36
YDL089W	24
YDL090C	102
YDL091C	26
YDL092W	15
YDL093W	228
YDL094C	54
YDL095W	103
YDL096C	101
YDL097C	47
YDL098C	12
YDL099W	74
YDL100C	12
YDL101C	21
YDL102W	15
YDL103C	85

YDL104C	37
YDL105W	740
YDL106C	10
YDL107W	10
YDL108W	178
YDL109C	180
YDL110C	7
YDL111C	16
YDL112W	33
YDL113C	9
YDL114W	7
YDL114W-A	93
YDL115C	28
YDL116W	17
YDL117W	130
YDL118W	45
YDL119C	29
YDL120W	17
YDL121C	31
YDL122W	29
YDL123W	188
YDL124W	48
YDL125C	90
YDL126C	16
YDL127W	69
YDL128W	60
YDL129W	6
YDL130W	41
YDL130W-A	83
YDL131W	53
YDL132W	16
YDL133C-A	257
YDL133W	159
YDL134C	41
YDL135C	33
YDL136W	79
YDL137W	114
YDL138W	356
YDL139C	24
YDL140C	32
YDL141W	10
YDL142C	34
YDL143W	11
YDL144C	20
YDL145C	354
YDL146W	59
YDL147W	4
YDL148C	9
YDL149W	63
YDL150W	25
YDL151C	6
YDL152W	59
YDL153C	33
YDL154W	73
YDL155W	25

YDL156W	15
YDL157C	114
YDL158C	41
YDL159C-B	6
YDL159W	19
YDL159W-A	20
YDL160C	523
YDL160C-A	220
YDL161W	2
YDL162C	247
YDL163W	259
YDL164C	140
YDL165W	19
YDL166C	21
YDL167C	7
YDL168W	153
YDL169C	59
YDL170W	291
YDL171C	154
YDL172C	45
YDL173W	119
YDL174C	38
YDL175C	14
YDL176W	88
YDL177C	324
YDL178W	81
YDL179W	49
YDL180W	48
YDL181W	260
YDL182W	19
YDL183C	264
YDL184C	110
YDL185C-A	11
YDL185W	23
YDL186W	7
YDL187C	9
YDL188C	44
YDL189W	371
YDL190C	78
YDL191W	58
YDL192W	409
YDL193W	89
YDL194W	13
YDL195W	71
YDL196W	61
YDL197C	45
YDL198C	33
YDL199C	41
YDL200C	44
YDL201W	19
YDL202W	51
YDL203C	6
YDL204W	580
YDL205C	222
YDL206W	78

YDL207W	12
YDL208W	151
YDL209C	53
YDL210W	738
YDL211C	23
YDL212W	317
YDL213C	237
YDL214C	50
YDL215C	21
YDL216C	45
YDL217C	13
YDL218W	12
YDL219W	128
YDL220C	842
YDL221W	7
YDL222C	74
YDL223C	336
YDL224C	27
YDL225W	8
YDL226C	82
YDL227C	4
YDL228C	24
YDL229W	139
YDL230W	51
YDL231C	61
YDL232W	16
YDL233W	15
YDL234C	121
YDL235C	91
YDL236W	30
YDL237W	97
YDL238C	38
YDL239C	332
YDL240C-A	277
YDL240W	250
YDL241W	55
YDL242W	31
YDL243C	6
YDL244W	40
YDL245C	26
YDL246C	88
YDL247W	80
YDL247W-A	166
YDL248W	45
YDR001C	13
YDR002W	212
YDR003W	3
YDR003W-A	63
YDR004W	8
YDR005C	1672
YDR006C	121
YDR007W	105
YDR008C	57
YDR009W	53
YDR010C	29

YDR011W	27
YDR012W	10
YDR013W	797
YDR014W	16
YDR014W-A	4
YDR015C	24
YDR016C	28
YDR017C	63
YDR018C	68
YDR019C	31
YDR020C	102
YDR021W	3
YDR022C	23
YDR023W	64
YDR024W	26
YDR025W	45
YDR026C	6
YDR027C	18
YDR028C	29
YDR029W	17
YDR030C	26
YDR031W	69
YDR032C	50
YDR033W	18
YDR034C	11
YDR034C-A	35
YDR034C-C	24
YDR034C-D	6
YDR034W-B	97
YDR035W	147
YDR036C	52
YDR037W	12
YDR038C	31
YDR039C	20
YDR040C	111
YDR041W	261
YDR042C	62
YDR043C	19
YDR044W	53
YDR045C	56
YDR046C	39
YDR047W	22
YDR048C	24
YDR049W	26
YDR050C	12
YDR051C	21
YDR052C	171
YDR053W	35
YDR054C	90
YDR055W	8
YDR056C	58
YDR057W	15
YDR058C	15
YDR059C	99
YDR060W	165

YDR061W	20
YDR062W	15
YDR063W	37
YDR064W	123
YDR065W	261
YDR066C	137
YDR067C	95
YDR068W	29
YDR069C	13
YDR070C	56
YDR071C	33
YDR072C	26
YDR073W	3
YDR074W	203
YDR075W	55
YDR076W	92
YDR077W	31
YDR078C	7
YDR079C-A	87
YDR079W	109
YDR080W	607
YDR081C	7
YDR082W	378
YDR083W	57
YDR084C	39
YDR085C	342
YDR086C	177
YDR087C	38
YDR088C	226
YDR089W	61
YDR090C	36
YDR091C	24
YDR092W	402
YDR093W	34
YDR094W	16
YDR095C	5
YDR096W	36
YDR097C	60
YDR098C	144
YDR098C-A	463
YDR098C-B	138
YDR099W	24
YDR100W	85
YDR101C	121
YDR102C	218
YDR103W	11
YDR104C	1
YDR105C	39
YDR106W	287
YDR107C	41
YDR108W	19
YDR109C	85
YDR110W	192
YDR111C	66
YDR112W	203

YDR113C	14
YDR114C	51
YDR115W	11
YDR116C	47
YDR117C	23
YDR118W	20
YDR118W-A	19
YDR119W	17
YDR119W-A	33
YDR120C	50
YDR121W	21
YDR122W	23
YDR123C	3
YDR124W	72
YDR125C	39
YDR126W	5
YDR127W	7
YDR128W	179
YDR129C	140
YDR130C	28
YDR131C	34
YDR132C	13
YDR133C	17
YDR134C	17
YDR135C	6
YDR136C	37
YDR137W	11
YDR138W	34
YDR139C	331
YDR140W	37
YDR141C	6
YDR142C	35
YDR143C	20
YDR144C	87
YDR145W	58
YDR146C	27
YDR147W	96
YDR148C	45
YDR149C	138
YDR150W	56
YDR151C	135
YDR152W	198
YDR153C	52
YDR154C	45
YDR155C	15
YDR156W	32
YDR157W	3
YDR158W	27
YDR159W	43
YDR160W	47
YDR161W	51
YDR162C	41
YDR163W	10
YDR164C	45
YDR165W	27

YDR166C	74
YDR167W	51
YDR168W	50
YDR169C	42
YDR169C-A	5
YDR170C	61
YDR170W-A	140
YDR171W	364
YDR172W	25
YDR173C	137
YDR174W	72
YDR175C	192
YDR176W	51
YDR177W	51
YDR178W	23
YDR179C	97
YDR179W-A	81
YDR180W	2
YDR181C	9
YDR182W	35
YDR182W-A	34
YDR183C-A	102
YDR183W	247
YDR184C	20
YDR185C	25
YDR186C	121
YDR187C	76
YDR188W	493
YDR189W	76
YDR190C	11
YDR191W	223
YDR192C	79
YDR193W	1
YDR194C	123
YDR194W-A	104
YDR195W	95
YDR196C	56
YDR197W	18
YDR198C	120
YDR199W	14
YDR200C	36
YDR201W	227
YDR202C	214
YDR203W	3
YDR204W	259
YDR205W	23
YDR206W	277
YDR207C	9
YDR208W	11
YDR209C	38
YDR210C-C	107
YDR210C-D	37
YDR210W	20
YDR210W-A	22
YDR210W-B	54

YDR211W	139
YDR212W	5
YDR213W	17
YDR214W	94
YDR215C	14
YDR216W	79
YDR217C	184
YDR218C	41
YDR219C	16
YDR220C	147
YDR221W	10
YDR222W	110
YDR223W	335
YDR224C	80
YDR225W	23
YDR226W	651
YDR227W	11
YDR228C	61
YDR229W	17
YDR230W	44
YDR231C	69
YDR232W	138
YDR233C	70
YDR234W	8
YDR235W	30
YDR236C	8
YDR237W	20
YDR238C	54
YDR239C	27
YDR240C	28
YDR241W	91
YDR242W	143
YDR243C	63
YDR244W	10
YDR245W	14
YDR246W	98
YDR246W-A	212
YDR247W	94
YDR248C	21
YDR249C	18
YDR250C	91
YDR251W	44
YDR252W	295
YDR253C	30
YDR254W	35
YDR255C	319
YDR256C	105
YDR257C	2
YDR258C	103
YDR259C	31
YDR260C	8
YDR261C	107
YDR261C-C	53
YDR261C-D	25
YDR261W-A	59

YDR261W-B	126
YDR262W	22
YDR263C	5
YDR264C	21
YDR265W	26
YDR266C	63
YDR267C	19
YDR268W	16
YDR269C	51
YDR270W	237
YDR271C	175
YDR272W	39
YDR273W	66
YDR274C	135
YDR275W	20
YDR276C	35
YDR277C	221
YDR278C	83
YDR279W	24
YDR280W	28
YDR281C	164
YDR282C	427
YDR283C	60
YDR284C	85
YDR285W	80
YDR286C	11
YDR287W	78
YDR288W	20
YDR289C	4
YDR290W	173
YDR291W	24
YDR292C	30
YDR293C	237
YDR294C	49
YDR295C	32
YDR296W	26
YDR297W	55
YDR298C	44
YDR299W	39
YDR300C	186
YDR301W	15
YDR302W	5
YDR303C	254
YDR304C	104
YDR305C	5
YDR306C	104
YDR307W	17
YDR308C	63
YDR309C	76
YDR310C	13
YDR311W	24
YDR312W	10
YDR313C	18
YDR314C	28
YDR315C	10

YDR316W	28
YDR316W-A	8
YDR316W-B	290
YDR317W	67
YDR318W	110
YDR319C	59
YDR320C	6
YDR320C-A	20
YDR320W-B	52
YDR321W	1
YDR322C-A	6
YDR322W	87
YDR323C	22
YDR324C	51
YDR325W	473
YDR326C	2
YDR327W	418
YDR328C	20
YDR329C	15
YDR330W	638
YDR331W	190
YDR332W	567
YDR333C	37
YDR334W	67
YDR335W	124
YDR336W	113
YDR337W	19
YDR338C	40
YDR339C	51
YDR340W	3
YDR341C	14
YDR342C	28
YDR343C	150
YDR344C	93
YDR345C	10
YDR346C	13
YDR347W	23
YDR348C	21
YDR349C	46
YDR350C	58
YDR351W	243
YDR352W	212
YDR353W	169
YDR354C-A	83
YDR354W	102
YDR355C	9
YDR356W	47
YDR357C	95
YDR358W	146
YDR359C	163
YDR360W	74
YDR361C	21
YDR362C	26
YDR363W	299
YDR363W-A	45

YDR364C	90
YDR365C	31
YDR365W-A	10
YDR365W-B	316
YDR366C	24
YDR367W	111
YDR368W	116
YDR369C	70
YDR370C	53
YDR371C-A	263
YDR371W	83
YDR372C	3
YDR373W	30
YDR374C	69
YDR374W-A	27
YDR375C	11
YDR376W	99
YDR377W	111
YDR378C	6
YDR379C-A	5
YDR379W	31
YDR380W	24
YDR381C-A	5
YDR381W	235
YDR382W	125
YDR383C	112
YDR384C	106
YDR385W	44
YDR386W	141
YDR387C	63
YDR388W	75
YDR389W	21
YDR390C	793
YDR391C	90
YDR392W	23
YDR393W	41
YDR394W	62
YDR395W	113
YDR396W	131
YDR397C	31
YDR398W	90
YDR399W	121
YDR400W	288
YDR401W	24
YDR402C	50
YDR403W	206
YDR404C	82
YDR405W	40
YDR406W	8
YDR406W-A	63
YDR407C	138
YDR408C	23
YDR409W	86
YDR410C	28
YDR411C	357

YDR412W	70
YDR413C	56
YDR414C	35
YDR415C	68
YDR416W	102
YDR417C	60
YDR418W	76
YDR419W	109
YDR420W	27
YDR421W	24
YDR422C	59
YDR423C	5
YDR424C	88
YDR425W	131
YDR426C	27
YDR427W	38
YDR428C	10
YDR429C	167
YDR430C	48
YDR431W	29
YDR432W	52
YDR433W	44
YDR434W	96
YDR435C	32
YDR436W	19
YDR437W	23
YDR438W	103
YDR439W	10
YDR440W	91
YDR441C	61
YDR442W	8
YDR443C	17
YDR444W	35
YDR445C	25
YDR446W	25
YDR447C	114
YDR448W	45
YDR449C	64
YDR450W	10
YDR451C	121
YDR452W	12
YDR453C	38
YDR454C	13
YDR455C	9
YDR456W	9
YDR457W	79
YDR458C	23
YDR459C	9
YDR460W	28
YDR461C-A	40
YDR461W	288
YDR462W	488
YDR463W	181
YDR464C-A	18
YDR464W	118

YDR465C	85
YDR466W	11
YDR467C	88
YDR468C	22
YDR469W	28
YDR470C	49
YDR471W	97
YDR472W	196
YDR473C	36
YDR475C	105
YDR476C	63
YDR477W	21
YDR478W	192
YDR479C	17
YDR480W	20
YDR481C	137
YDR482C	47
YDR483W	152
YDR484W	20
YDR485C	9
YDR486C	14
YDR487C	27
YDR488C	26
YDR489W	182
YDR490C	63
YDR491C	500
YDR492W	22
YDR493W	375
YDR494W	62
YDR495C	21
YDR496C	14
YDR497C	342
YDR498C	31
YDR499W	33
YDR500C	27
YDR501W	35
YDR502C	18
YDR503C	122
YDR504C	32
YDR505C	52
YDR506C	36
YDR507C	29
YDR508C	469
YDR509W	108
YDR510C-A	28
YDR510W	50
YDR511W	76
YDR512C	5
YDR513W	101
YDR514C	68
YDR515W	13
YDR516C	183
YDR517W	24
YDR518W	54
YDR519W	21

YDR520C	36
YDR521W	164
YDR522C	122
YDR523C	58
YDR524C	49
YDR524C-A	18
YDR524C-B	8
YDR524W-C	78
YDR525W	4
YDR525W-A	512
YDR526C	51
YDR527W	84
YDR528W	551
YDR529C	80
YDR530C	63
YDR531W	24
YDR532C	49
YDR533C	22
YDR534C	70
YDR535C	240
YDR536W	5
YDR537C	29
YDR538W	5
YDR539W	15
YDR540C	44
YDR541C	70
YDR542W	21
YDR543C	14
YDR544C	433
YDR545C-A	74
YDR545W	125
YEL001C	237
YEL002C	328
YEL003W	8
YEL004W	84
YEL005C	10
YEL006W	18
YEL007W	23
YEL008C-A	11
YEL008W	56
YEL009C	25
YEL009C-A	13
YEL010W	72
YEL011W	10
YEL012W	33
YEL013W	36
YEL014C	10
YEL015W	15
YEL016C	268
YEL017C-A	46
YEL017W	26
YEL018C-A	119
YEL018W	84
YEL019C	17
YEL020C	68

YEL020C-B	27
YEL020W-A	127
YEL021W	5
YEL022W	350
YEL023C	16
YEL024W	4
YEL025C	105
YEL026W	35
YEL027W	10
YEL028W	112
YEL029C	220
YEL030C-A	114
YEL030W	8
YEL031W	728
YEL032C-A	41
YEL032W	75
YEL033W	37
YEL034C-A	49
YEL034W	29
YEL035C	44
YEL036C	36
YEL037C	77
YEL038W	124
YEL039C	10
YEL040W	93
YEL041W	250
YEL042W	58
YEL043W	45
YEL044W	35
YEL045C	28
YEL046C	89
YEL047C	289
YEL048C	82
YEL049W	252
YEL050C	492
YEL050W-A	293
YEL051W	11
YEL052W	26
YEL053C	53
YEL053W-A	22
YEL054C	25
YEL055C	120
YEL056W	17
YEL057C	150
YEL058W	36
YEL059C-A	24
YEL059W	17
YEL060C	16
YEL061C	32
YEL062W	77
YEL063C	82
YEL064C	155
YEL065W	112
YEL066W	19
YEL067C	16

YEL068C	17
YEL069C	100
YEL070W	170
YEL071W	14
YEL072W	45
YEL073C	57
YEL074W	131
YEL075C	538
YEL075W-A	65
YEL076C	32
YEL076C-A	23
YEL077C	341
YEL077W-A	41
YER001W	18
YER002W	32
YER003C	76
YER004W	19
YER005W	165
YER006C-A	10
YER006W	65
YER007C-A	84
YER007W	6
YER008C	39
YER009W	38
YER010C	21
YER011W	92
YER012W	12
YER013W	37
YER014C-A	151
YER014W	288
YER015W	23
YER016W	91
YER017C	37
YER018C	149
YER019C-A	56
YER019W	11
YER020W	21
YER021W	159
YER022W	17
YER023C-A	84
YER023W	3
YER024W	5
YER025W	72
YER026C	318
YER027C	3
YER028C	96
YER029C	52
YER030W	61
YER031C	73
YER032W	107
YER033C	29
YER034W	17
YER035W	4
YER036C	46
YER037W	23

YER038C	121
YER038W-A	142
YER039C	11
YER039C-A	313
YER040W	16
YER041W	60
YER042W	6
YER043C	42
YER044C	6
YER044C-A	41
YER045C	74
YER046W	74
YER046W-A	46
YER047C	57
YER048C	33
YER048W-A	123
YER049W	26
YER050C	26
YER051W	87
YER052C	74
YER053C	77
YER053C-A	10
YER054C	20
YER055C	18
YER056C	15
YER056C-A	5
YER057C	36
YER058W	57
YER059W	22
YER060W	242
YER060W-A	28
YER061C	32
YER062C	118
YER063W	12
YER064C	14
YER065C	28
YER066C-A	206
YER066W	83
YER067C-A	146
YER067W	41
YER068C-A	64
YER068W	28
YER069W	2
YER070W	33
YER071C	183
YER072W	238
YER073W	55
YER074W	297
YER074W-A	23
YER075C	13
YER076C	34
YER076W-A	10
YER077C	75
YER078C	64
YER078W-A	31

YER079C-A	39
YER079W	13
YER080W	123
YER081W	222
YER082C	194
YER083C	13
YER084W	34
YER084W-A	108
YER085C	229
YER086W	77
YER087C-A	17
YER087C-B	157
YER087W	12
YER088C	14
YER088C-A	50
YER088W-B	24
YER089C	15
YER090C-A	37
YER090W	222
YER091C	39
YER091C-A	50
YER092W	38
YER093C	266
YER093C-A	27
YER094C	131
YER095W	31
YER096W	539
YER097W	84
YER098W	3
YER099C	120
YER100W	15
YER101C	139
YER102W	14
YER103W	100
YER104W	235
YER105C	75
YER106W	22
YER107C	56
YER107W-A	32
YER109C	57
YER110C	25
YER111C	16
YER112W	47
YER113C	290
YER114C	975
YER115C	59
YER116C	91
YER117W	149
YER118C	96
YER119C	204
YER119C-A	37
YER120W	10
YER121W	62
YER122C	6
YER123W	116

YER124C	65
YER125W	232
YER126C	5
YER127W	117
YER128W	26
YER129W	4
YER130C	5
YER131W	909
YER132C	70
YER133W	179
YER133W-A	1
YER134C	204
YER135C	125
YER136W	7
YER137C	17
YER137C-A	41
YER137W-A	13
YER138C	24
YER138W-A	12
YER139C	122
YER140W	20
YER141W	39
YER142C	10
YER143W	25
YER144C	59
YER145C	99
YER145C-A	86
YER146W	34
YER147C	6
YER147C-A	328
YER148W	9
YER148W-A	79
YER149C	23
YER150W	41
YER151C	45
YER152C	52
YER152W-A	139
YER153C	155
YER154W	48
YER155C	850
YER156C	32
YER157W	66
YER158C	11
YER158W-A	91
YER159C	87
YER159C-A	20
YER160C	69
YER161C	49
YER162C	72
YER163C	336
YER164W	101
YER165C-A	81
YER165W	83
YER166W	52
YER167W	172

YER168C	15
YER169W	18
YER170W	239
YER171W	23
YER172C	107
YER172C-A	117
YER173W	29
YER174C	25
YER175C	42
YER175W-A	27
YER176W	30
YER177W	4
YER178W	59
YER179W	34
YER180C	16
YER180C-A	14
YER181C	24
YER182W	74
YER183C	34
YER184C	11
YER185W	39
YER186C	14
YER187W	42
YER188C-A	474
YER188W	37
YER189W	19
YER190C-A	52
YER190C-B	137
YER190W	154
YFL001W	143
YFL002C	53
YFL002W-A	20
YFL002W-B	37
YFL003C	203
YFL004W	47
YFL005W	6
YFL007W	145
YFL008W	8
YFL009W	26
YFL010C	99
YFL010W-A	115
YFL011W	27
YFL012W	157
YFL012W-A	43
YFL013C	31
YFL013W-A	17
YFL014W	15
YFL015C	12
YFL015W-A	39
YFL016C	62
YFL017C	104
YFL017W-A	2
YFL018C	16
YFL019C	534
YFL020C	42

YFL021C-A	67
YFL021W	97
YFL022C	67
YFL023W	25
YFL024C	33
YFL025C	22
YFL026W	12
YFL027C	165
YFL028C	11
YFL029C	14
YFL030W	367
YFL031C-A	105
YFL031W	21
YFL032W	46
YFL033C	67
YFL034C-A	28
YFL034C-B	23
YFL034W	40
YFL036W	134
YFL037W	42
YFL038C	581
YFL039C	71
YFL040W	181
YFL041W	229
YFL041W-A	573
YFL042C	21
YFL044C	93
YFL045C	19
YFL046W	21
YFL047W	12
YFL048C	3
YFL049W	40
YFL050C	27
YFL051C	9
YFL052W	150
YFL053W	9
YFL054C	21
YFL055W	10
YFL056C	16
YFL057C	50
YFL058W	430
YFL059W	16
YFL060C	125
YFL061W	54
YFL062W	115
YFL063W	30
YFL064C	156
YFL065C	18
YFL066C	5
YFL067W	160
YFL068W	32
YFR001W	63
YFR002W	67
YFR003C	60
YFR004W	208

YFR005C	38
YFR006W	53
YFR007W	126
YFR008W	5
YFR009W	34
YFR009W-A	27
YFR010W	129
YFR010W-A	15
YFR011C	99
YFR012W	9
YFR012W-A	32
YFR013W	30
YFR014C	7
YFR015C	61
YFR016C	12
YFR017C	42
YFR018C	88
YFR019W	15
YFR020W	36
YFR021W	111
YFR022W	26
YFR023W	27
YFR024C-A	24
YFR025C	10
YFR026C	80
YFR027W	9
YFR028C	5
YFR029W	34
YFR030W	207
YFR031C	74
YFR031C-A	24
YFR032C	14
YFR032C-A	68
YFR032C-B	22
YFR033C	85
YFR034C	118
YFR034W-A	145
YFR035C	11
YFR036W	69
YFR036W-A	37
YFR037C	115
YFR038W	206
YFR039C	90
YFR040W	101
YFR041C	483
YFR042W	98
YFR043C	112
YFR044C	12
YFR045W	9
YFR046C	62
YFR047C	96
YFR048W	50
YFR049W	48
YFR050C	113
YFR051C	38

YFR052C-A	181
YFR052W	12
YFR053C	35
YFR054C	425
YFR055W	10
YFR056C	61
YFR057W	334
YGL001C	30
YGL002W	1
YGL003C	37
YGL004C	10
YGL005C	10
YGL006W	10
YGL006W-A	22
YGL007C-A	81
YGL007W	9
YGL008C	4
YGL009C	46
YGL010W	307
YGL011C	169
YGL012W	19
YGL013C	14
YGL014C-A	85
YGL014W	14
YGL015C	648
YGL016W	131
YGL017W	72
YGL018C	15
YGL019W	147
YGL020C	21
YGL021W	368
YGL022W	74
YGL023C	25
YGL024W	63
YGL025C	15
YGL026C	188
YGL027C	44
YGL028C	48
YGL029W	26
YGL030W	32
YGL031C	11
YGL032C	3
YGL033W	140
YGL034C	152
YGL035C	42
YGL036W	14
YGL037C	28
YGL038C	167
YGL039W	25
YGL040C	67
YGL041C	121
YGL041C-B	20
YGL041W-A	39
YGL042C	14
YGL043W	48

YGL044C	11
YGL045W	38
YGL047W	64
YGL048C	60
YGL049C	81
YGL050W	129
YGL051W	144
YGL052W	115
YGL053W	480
YGL054C	24
YGL055W	4
YGL056C	44
YGL057C	45
YGL058W	24
YGL059W	260
YGL060W	29
YGL061C	25
YGL062W	5
YGL063C-A	71
YGL063W	107
YGL064C	75
YGL065C	34
YGL066W	29
YGL067W	28
YGL068W	27
YGL069C	67
YGL070C	5
YGL071W	17
YGL072C	8
YGL073W	31
YGL074C	84
YGL075C	44
YGL076C	46
YGL077C	21
YGL078C	106
YGL079W	136
YGL080W	22
YGL081W	22
YGL082W	11
YGL083W	71
YGL084C	19
YGL085W	55
YGL086W	270
YGL087C	4
YGL088W	21
YGL089C	30
YGL090W	24
YGL091C	430
YGL092W	186
YGL093W	118
YGL094C	33
YGL095C	86
YGL096W	22
YGL097W	71
YGL098W	10

YGL099W	201
YGL100W	31
YGL101W	29
YGL102C	4
YGL103W	17
YGL104C	6
YGL105W	88
YGL106W	48
YGL107C	3
YGL108C	9
YGL109W	24
YGL110C	22
YGL111W	43
YGL112C	42
YGL113W	485
YGL114W	3
YGL115W	7
YGL116W	24
YGL117W	57
YGL118C	165
YGL119W	164
YGL120C	15
YGL121C	15
YGL122C	36
YGL123C-A	128
YGL123W	92
YGL124C	39
YGL125W	36
YGL126W	8
YGL127C	29
YGL128C	1084
YGL129C	349
YGL130W	28
YGL131C	82
YGL132W	626
YGL133W	169
YGL134W	12
YGL135W	83
YGL136C	92
YGL137W	10
YGL138C	10
YGL139W	6
YGL140C	20
YGL141W	3
YGL142C	29
YGL143C	81
YGL144C	85
YGL145W	44
YGL146C	12
YGL147C	193
YGL148W	345
YGL149W	473
YGL150C	15
YGL151W	38
YGL152C	20

YGL153W	47
YGL154C	3
YGL155W	100
YGL156W	56
YGL157W	97
YGL158W	342
YGL159W	7
YGL160W	36
YGL161C	67
YGL162W	15
YGL163C	108
YGL164C	34
YGL165C	4
YGL166W	37
YGL167C	51
YGL168W	27
YGL169W	128
YGL170C	23
YGL171W	8
YGL172W	38
YGL173C	27
YGL174W	89
YGL175C	41
YGL176C	12
YGL177W	117
YGL178W	43
YGL179C	43
YGL180W	27
YGL181W	75
YGL182C	48
YGL183C	74
YGL184C	11
YGL185C	7
YGL186C	148
YGL187C	19
YGL188C	4
YGL188C-A	293
YGL189C	357
YGL190C	204
YGL191W	202
YGL192W	60
YGL193C	33
YGL194C	56
YGL194C-A	14
YGL195W	9
YGL196W	57
YGL197W	195
YGL198W	135
YGL199C	115
YGL200C	12
YGL201C	59
YGL202W	60
YGL203C	168
YGL204C	46
YGL205W	85

YGL206C	73
YGL207W	12
YGL208W	21
YGL209W	32
YGL210W	70
YGL211W	47
YGL212W	200
YGL213C	8
YGL214W	4
YGL215W	3
YGL216W	333
YGL217C	89
YGL218W	5
YGL219C	41
YGL220W	17
YGL221C	154
YGL222C	61
YGL223C	38
YGL224C	184
YGL225W	29
YGL226C-A	22
YGL226W	56
YGL227W	966
YGL228W	1
YGL229C	89
YGL230C	30
YGL231C	66
YGL232W	25
YGL233W	502
YGL234W	194
YGL235W	318
YGL236C	19
YGL237C	19
YGL238W	108
YGL239C	79
YGL240W	81
YGL241W	106
YGL242C	41
YGL243W	65
YGL244W	21
YGL245W	96
YGL246C	91
YGL247W	95
YGL248W	28
YGL249W	276
YGL250W	8
YGL251C	242
YGL252C	36
YGL253W	19
YGL254W	120
YGL255W	296
YGL256W	5
YGL257C	277
YGL258W	28
YGL258W-A	63

YGL259W	240
YGL260W	42
YGL261C	14
YGL262W	344
YGL263W	106
YGR001C	52
YGR002C	53
YGR003W	38
YGR004W	23
YGR005C	164
YGR006W	23
YGR007W	64
YGR008C	66
YGR009C	111
YGR010W	640
YGR011W	6
YGR012W	120
YGR013W	45
YGR014W	42
YGR015C	29
YGR016W	2452
YGR017W	171
YGR018C	14
YGR019W	57
YGR020C	217
YGR021W	44
YGR022C	224
YGR023W	19
YGR024C	125
YGR025W	16
YGR026W	85
YGR027C	29
YGR027W-A	166
YGR027W-B	103
YGR028W	90
YGR029W	39
YGR030C	32
YGR031C-A	40
YGR031W	37
YGR032W	20
YGR033C	28
YGR034W	12
YGR035C	25
YGR035W-A	105
YGR036C	20
YGR037C	89
YGR038C-A	24
YGR038C-B	65
YGR038W	52
YGR039W	106
YGR040W	11
YGR041W	16
YGR042W	51
YGR043C	123
YGR044C	44

YGR045C	15
YGR046W	46
YGR047C	69
YGR048W	2546
YGR049W	19
YGR050C	85
YGR051C	154
YGR052W	16
YGR053C	238
YGR054W	69
YGR055W	191
YGR056W	32
YGR057C	263
YGR058W	32
YGR059W	72
YGR060W	145
YGR061C	57
YGR062C	315
YGR063C	62
YGR064W	28
YGR065C	11
YGR066C	85
YGR067C	11
YGR068C	230
YGR068W-A	106
YGR069W	31
YGR070W	19
YGR071C	61
YGR072W	33
YGR073C	47
YGR074W	3
YGR075C	30
YGR076C	440
YGR077C	11
YGR078C	17
YGR079W	305
YGR080W	39
YGR081C	625
YGR082W	116
YGR083C	66
YGR084C	30
YGR085C	25
YGR086C	65
YGR087C	7
YGR088W	151
YGR089W	319
YGR090W	14
YGR091W	48
YGR092W	105
YGR093W	31
YGR094W	24
YGR095C	37
YGR096W	25
YGR097W	157
YGR098C	35

YGR099W	83
YGR100W	18
YGR101W	72
YGR102C	108
YGR103W	41
YGR104C	111
YGR105W	46
YGR106C	341
YGR107W	12
YGR108W	22
YGR109C	50
YGR109W-A	9
YGR109W-B	46
YGR110W	124
YGR111W	621
YGR112W	36
YGR113W	59
YGR114C	22
YGR115C	18
YGR116W	261
YGR117C	14
YGR118W	18
YGR119C	12
YGR120C	86
YGR121C	205
YGR121W-A	35
YGR122C-A	5
YGR122W	8
YGR123C	27
YGR124W	8
YGR125W	35
YGR126W	256
YGR127W	58
YGR128C	31
YGR129W	70
YGR130C	22
YGR131W	10
YGR132C	52
YGR133W	170
YGR134W	44
YGR135W	147
YGR136W	19
YGR137W	21
YGR138C	18
YGR139W	18
YGR140W	69
YGR141W	94
YGR142W	17
YGR143W	68
YGR144W	59
YGR145W	249
YGR146C	109
YGR146C-A	30
YGR147C	58
YGR148C	8

YGR149W	23
YGR150C	1137
YGR151C	32
YGR152C	12
YGR153W	60
YGR154C	22
YGR155W	149
YGR156W	81
YGR157W	107
YGR158C	87
YGR159C	30
YGR160W	53
YGR161C	262
YGR161C-C	59
YGR161C-D	14
YGR161W-A	41
YGR161W-B	160
YGR161W-C	207
YGR162W	10
YGR163W	7
YGR164W	101
YGR165W	18
YGR166W	243
YGR167W	75
YGR168C	63
YGR169C	28
YGR169C-A	61
YGR170W	65
YGR171C	41
YGR172C	18
YGR173W	18
YGR174C	155
YGR174W-A	129
YGR175C	21
YGR176W	41
YGR177C	36
YGR178C	198
YGR179C	52
YGR180C	120
YGR181W	18
YGR182C	81
YGR183C	48
YGR184C	303
YGR185C	66
YGR186W	3
YGR187C	69
YGR188C	14
YGR189C	27
YGR190C	440
YGR191W	81
YGR192C	125
YGR193C	16
YGR194C	4
YGR195W	43
YGR196C	20

YGR197C	144
YGR198W	214
YGR199W	9
YGR200C	95
YGR201C	46
YGR202C	144
YGR203W	19
YGR204C-A	11
YGR204W	81
YGR205W	57
YGR206W	50
YGR207C	57
YGR208W	22
YGR209C	16
YGR210C	20
YGR211W	96
YGR212W	39
YGR213C	104
YGR214W	330
YGR215W	31
YGR216C	56
YGR217W	15
YGR218W	7
YGR219W	137
YGR220C	2
YGR221C	7
YGR222W	107
YGR223C	40
YGR224W	32
YGR225W	76
YGR226C	84
YGR227W	35
YGR228W	17
YGR229C	19
YGR230W	99
YGR231C	41
YGR232W	41
YGR233C	38
YGR234W	142
YGR235C	20
YGR236C	56
YGR237C	67
YGR238C	77
YGR239C	73
YGR240C	60
YGR240C-A	150
YGR241C	593
YGR242W	46
YGR243W	9
YGR244C	50
YGR245C	7
YGR246C	9
YGR247W	20
YGR248W	122
YGR249W	62

YGR250C	59
YGR251W	13
YGR252W	7
YGR253C	143
YGR254W	61
YGR255C	73
YGR256W	71
YGR257C	21
YGR258C	21
YGR259C	261
YGR260W	487
YGR261C	23
YGR262C	131
YGR263C	93
YGR264C	31
YGR265W	278
YGR266W	68
YGR267C	1
YGR268C	17
YGR269W	78
YGR270C-A	336
YGR270W	902
YGR271C-A	55
YGR271W	38
YGR272C	232
YGR273C	127
YGR274C	311
YGR275W	76
YGR276C	24
YGR277C	65
YGR278W	269
YGR279C	3
YGR280C	36
YGR281W	147
YGR282C	26
YGR283C	43
YGR284C	15
YGR285C	64
YGR286C	125
YGR287C	30
YGR288W	188
YGR289C	73
YGR290W	46
YGR291C	7
YGR292W	114
YGR293C	124
YGR294W	72
YGR295C	439
YGR296C-A	59
YGR296C-B	7
YGR296W	41
YHL001W	38
YHL002C-A	111
YHL002W	120
YHL003C	40

YHL004W	163
YHL005C	54
YHL006C	287
YHL006W-A	152
YHL007C	72
YHL008C	115
YHL009C	147
YHL009W-A	21
YHL009W-B	94
YHL010C	120
YHL011C	47
YHL012W	23
YHL013C	12
YHL014C	176
YHL015W	30
YHL015W-A	61
YHL016C	53
YHL017W	226
YHL018W	70
YHL019C	22
YHL019W-A	176
YHL020C	18
YHL021C	9
YHL022C	43
YHL023C	10
YHL024W	20
YHL025W	74
YHL026C	87
YHL027W	4
YHL028W	3
YHL029C	20
YHL030W	31
YHL030W-A	44
YHL031C	131
YHL032C	89
YHL033C	44
YHL034C	49
YHL034W-A	18
YHL035C	116
YHL036W	92
YHL037C	33
YHL038C	27
YHL039W	80
YHL040C	4
YHL041W	70
YHL042W	41
YHL043W	45
YHL044W	23
YHL045W	9
YHL046C	28
YHL046W-A	61
YHL047C	217
YHL048C-A	51
YHL048W	92
YHL049C	195

YHL050C	5
YHL050W-A	132
YHR001W	65
YHR001W-A	900
YHR002W	64
YHR003C	22
YHR004C	20
YHR005C	228
YHR005C-A	28
YHR006W	100
YHR007C	90
YHR007C-A	27
YHR008C	17
YHR009C	105
YHR010W	13
YHR011W	65
YHR012W	35
YHR013C	5
YHR014W	13
YHR015W	57
YHR016C	45
YHR017W	13
YHR018C	25
YHR019C	187
YHR020W	166
YHR021C	35
YHR021W-A	32
YHR022C	49
YHR022C-A	26
YHR023W	27
YHR024C	161
YHR025W	70
YHR026W	59
YHR027C	12
YHR028C	47
YHR028W-A	90
YHR029C	54
YHR030C	325
YHR031C	28
YHR032C-A	190
YHR032W	53
YHR032W-A	138
YHR033W	13
YHR034C	7
YHR035W	106
YHR036W	29
YHR037W	51
YHR038W	21
YHR039C	26
YHR039C-A	29
YHR040W	136
YHR041C	38
YHR042W	25
YHR043C	54
YHR044C	51

YHR045W	33
YHR046C	19
YHR047C	70
YHR048W	172
YHR049C-A	34
YHR049W	14
YHR050W	198
YHR050W-A	237
YHR051W	70
YHR052W	18
YHR052W-A	41
YHR053C	71
YHR054C	41
YHR054W-A	190
YHR055C	49
YHR056C	15
YHR056W-A	5
YHR057C	58
YHR058C	9
YHR059W	201
YHR060W	14
YHR061C	44
YHR062C	33
YHR063C	183
YHR063W-A	17
YHR064C	129
YHR065C	20
YHR066W	39
YHR067W	11
YHR068W	42
YHR069C	35
YHR069C-A	4
YHR070C-A	175
YHR070W	71
YHR071C-A	9
YHR071W	49
YHR072W	5
YHR072W-A	18
YHR073C-B	33
YHR073W	35
YHR073W-A	24
YHR074W	285
YHR075C	41
YHR076W	284
YHR077C	203
YHR078W	54
YHR079C	121
YHR079C-A	28
YHR080C	13
YHR081W	38
YHR082C	88
YHR083W	64
YHR084W	17
YHR085W	43
YHR086W	48

YHR086W-A	13
YHR087W	23
YHR088W	6
YHR089C	85
YHR090C	4
YHR091C	78
YHR092C	16
YHR093W	29
YHR094C	96
YHR095W	9
YHR096C	96
YHR097C	23
YHR098C	10
YHR099W	18
YHR100C	31
YHR101C	159
YHR102W	34
YHR103W	152
YHR104W	4
YHR105W	239
YHR106W	5
YHR107C	62
YHR108W	172
YHR109W	13
YHR110W	18
YHR111W	1076
YHR112C	14
YHR113W	68
YHR114W	94
YHR115C	39
YHR116W	14
YHR117W	18
YHR118C	52
YHR119W	26
YHR120W	25
YHR121W	169
YHR122W	46
YHR123W	66
YHR124W	76
YHR125W	9
YHR126C	150
YHR127W	12
YHR128W	19
YHR129C	555
YHR130C	125
YHR131C	16
YHR131W-A	8
YHR132C	53
YHR132W-A	201
YHR133C	192
YHR134W	25
YHR135C	159
YHR136C	65
YHR137C-A	37
YHR137W	31

YHR138C	12
YHR139C	18
YHR139C-A	13
YHR140W	234
YHR141C	96
YHR142W	332
YHR143W	126
YHR143W-A	48
YHR144C	18
YHR145C	24
YHR146W	38
YHR147C	65
YHR148W	26
YHR149C	182
YHR150W	412
YHR151C	124
YHR152W	5
YHR153C	19
YHR154W	25
YHR155W	107
YHR156C	71
YHR157W	23
YHR158C	91
YHR159W	405
YHR160C	192
YHR161C	5
YHR162W	18
YHR163W	88
YHR164C	294
YHR165C	134
YHR165W-A	15
YHR166C	98
YHR167W	35
YHR168W	6
YHR169W	23
YHR170W	14
YHR171W	23
YHR172W	297
YHR173C	103
YHR174W	103
YHR175W	95
YHR175W-A	15
YHR176W	31
YHR177W	25
YHR178W	17
YHR179W	407
YHR180C-B	17
YHR180W	12
YHR180W-A	102
YHR181W	78
YHR182C-A	119
YHR182W	25
YHR183W	46
YHR184W	942
YHR185C	66

YHR186C	24
YHR187W	35
YHR188C	458
YHR189W	41
YHR190W	37
YHR191C	121
YHR192W	27
YHR193C	42
YHR193C-A	53
YHR194W	15
YHR195W	80
YHR196W	9
YHR197W	78
YHR198C	286
YHR199C	18
YHR199C-A	18
YHR200W	52
YHR201C	52
YHR202W	83
YHR203C	137
YHR204W	97
YHR205W	255
YHR206W	467
YHR207C	15
YHR208W	94
YHR209W	47
YHR210C	47
YHR211W	13
YHR212C	29
YHR212W-A	21
YHR213W	45
YHR213W-A	4
YHR213W-B	18
YHR214C-B	340
YHR214C-C	54
YHR214C-D	10
YHR214C-E	12
YHR214W	66
YHR214W-A	52
YHR215W	14
YHR216W	58
YHR217C	14
YHR218W	75
YHR218W-A	4
YHR219C-A	266
YHR219W	1
YIL001W	11
YIL002C	57
YIL002W-A	19
YIL003W	92
YIL004C	44
YIL005W	373
YIL006W	12
YIL007C	26
YIL008W	21

YIL009C-A	49
YIL009W	74
YIL010W	265
YIL011W	51
YIL012W	23
YIL013C	9
YIL014C-A	690
YIL014W	62
YIL015W	63
YIL016W	102
YIL017C	125
YIL018W	61
YIL019W	31
YIL020C	41
YIL020C-A	68
YIL021C-A	74
YIL021W	93
YIL022W	60
YIL023C	195
YIL024C	12
YIL025C	78
YIL026C	71
YIL027C	40
YIL028W	68
YIL029C	37
YIL029W-A	13
YIL030C	8
YIL030W-A	63
YIL031W	8
YIL032C	73
YIL033C	61
YIL034C	473
YIL035C	99
YIL036W	60
YIL037C	39
YIL038C	22
YIL039W	57
YIL040W	63
YIL041W	28
YIL042C	11
YIL043C	381
YIL044C	19
YIL045W	26
YIL046W	7
YIL046W-A	32
YIL047C	39
YIL047C-A	17
YIL048W	42
YIL049W	177
YIL050W	8
YIL051C	117
YIL052C	23
YIL053W	167
YIL054W	51
YIL055C	63

YIL056W	25
YIL057C	26
YIL058W	85
YIL059C	93
YIL060W	112
YIL061C	30
YIL062C	22
YIL063C	141
YIL064W	141
YIL065C	53
YIL066C	130
YIL066W-A	266
YIL067C	113
YIL068C	109
YIL068W-A	41
YIL069C	11
YIL070C	248
YIL071C	14
YIL071W-A	38
YIL072W	19
YIL073C	23
YIL074C	65
YIL075C	298
YIL076W	418
YIL077C	353
YIL078W	94
YIL079C	57
YIL080W	1
YIL082W	12
YIL082W-A	26
YIL083C	51
YIL084C	19
YIL085C	121
YIL086C	129
YIL087C	49
YIL088C	127
YIL089W	12
YIL090W	122
YIL091C	31
YIL092W	24
YIL093C	355
YIL094C	29
YIL095W	9
YIL096C	11
YIL097W	12
YIL098C	298
YIL099W	66
YIL100C-A	201
YIL100W	49
YIL101C	5
YIL102C	114
YIL102C-A	120
YIL103W	48
YIL104C	278
YIL105C	26

YIL105W-A	38
YIL106W	172
YIL107C	75
YIL108W	81
YIL109C	6
YIL110W	157
YIL111W	66
YIL112W	71
YIL113W	38
YIL114C	103
YIL115C	29
YIL115W-A	113
YIL116W	105
YIL117C	14
YIL118W	72
YIL119C	162
YIL120W	20
YIL121W	41
YIL122W	12
YIL123W	217
YIL124W	92
YIL125W	39
YIL126W	82
YIL127C	5
YIL128W	215
YIL129C	189
YIL130W	30
YIL131C	18
YIL132C	29
YIL133C	32
YIL134C-A	173
YIL134W	30
YIL135C	239
YIL136W	100
YIL137C	694
YIL138C	19
YIL139C	87
YIL140W	221
YIL141W	7
YIL142C-A	12
YIL142W	108
YIL143C	546
YIL144W	183
YIL145C	143
YIL146C	62
YIL147C	38
YIL148W	8
YIL149C	52
YIL150C	76
YIL151C	2
YIL152W	154
YIL153W	5
YIL154C	50
YIL155C	112
YIL156W	55

YIL156W-A	43
YIL156W-B	17
YIL157C	51
YIL158W	94
YIL159W	27
YIL160C	136
YIL161W	15
YIL162W	174
YIL163C	10
YIL164C	28
YIL165C	40
YIL166C	23
YIL167W	27
YIL168W	51
YIL169C	75
YIL170W	26
YIL171W	362
YIL171W-A	275
YIL172C	83
YIL173W	20
YIL174W	60
YIL175W	16
YIL176C	800
YIL177C	8
YIL177W-A	13
YIR001C	32
YIR002C	174
YIR003W	55
YIR004W	35
YIR005W	15
YIR006C	188
YIR007W	23
YIR008C	104
YIR009W	49
YIR010W	36
YIR011C	86
YIR012W	59
YIR013C	13
YIR014W	27
YIR015W	173
YIR016W	80
YIR017C	98
YIR017W-A	29
YIR018C-A	4
YIR018W	33
YIR019C	5
YIR020C	7
YIR020C-B	216
YIR020W-A	30
YIR021W	322
YIR021W-A	10
YIR022W	119
YIR023C-A	399
YIR023W	27
YIR024C	206

YIR025W	70
YIR026C	53
YIR027C	33
YIR028W	26
YIR029W	41
YIR030C	112
YIR030W-A	218
YIR031C	99
YIR032C	6
YIR033W	7
YIR034C	34
YIR035C	341
YIR036C	19
YIR036W-A	13
YIR037W	45
YIR038C	5
YIR039C	381
YIR040C	13
YIR041W	39
YIR042C	56
YIR043C	27
YIR044C	37
YJL001W	36
YJL002C	38
YJL003W	692
YJL004C	48
YJL005W	4
YJL006C	26
YJL007C	217
YJL008C	152
YJL009W	66
YJL010C	13
YJL011C	4
YJL012C	466
YJL013C	22
YJL014W	35
YJL015C	91
YJL016W	54
YJL019W	26
YJL020C	32
YJL020W-A	24
YJL022W	122
YJL023C	89
YJL024C	7
YJL025W	28
YJL026C-A	26
YJL026W	397
YJL027C	201
YJL028W	19
YJL029C	149
YJL030W	95
YJL031C	63
YJL032W	13
YJL033W	14
YJL034W	17

YJL035C	81
YJL036W	84
YJL037W	17
YJL038C	1172
YJL039C	45
YJL041W	29
YJL042W	97
YJL043W	28
YJL044C	80
YJL045W	172
YJL046W	132
YJL047C	71
YJL047C-A	139
YJL048C	7
YJL049W	118
YJL050W	407
YJL051W	28
YJL052C-A	294
YJL052W	46
YJL053W	19
YJL054W	126
YJL055W	11
YJL056C	18
YJL057C	48
YJL058C	199
YJL059W	58
YJL060W	22
YJL061W	17
YJL062W	188
YJL062W-A	32
YJL063C	12
YJL064W	54
YJL065C	46
YJL066C	12
YJL067W	20
YJL068C	48
YJL069C	80
YJL070C	82
YJL071W	68
YJL072C	82
YJL073W	15
YJL074C	7
YJL075C	74
YJL076W	2
YJL077C	10
YJL077W-A	77
YJL077W-B	194
YJL078C	90
YJL079C	18
YJL080C	67
YJL081C	32
YJL082W	32
YJL083W	7
YJL084C	5
YJL085W	14

YJL086C	9
YJL087C	4
YJL088W	76
YJL089W	29
YJL090C	29
YJL091C	73
YJL092W	67
YJL093C	59
YJL094C	126
YJL095W	20
YJL096W	48
YJL097W	79
YJL098W	122
YJL099W	11
YJL100W	139
YJL101C	145
YJL102W	15
YJL103C	192
YJL104W	68
YJL105W	112
YJL106W	5
YJL107C	191
YJL108C	90
YJL109C	81
YJL110C	205
YJL111W	68
YJL112W	29
YJL113W	36
YJL114W	20
YJL115W	32
YJL116C	109
YJL117W	127
YJL118W	82
YJL119C	218
YJL120W	60
YJL121C	12
YJL122W	95
YJL123C	104
YJL124C	152
YJL125C	135
YJL126W	94
YJL127C	42
YJL127C-B	92
YJL127W-A	159
YJL128C	179
YJL129C	48
YJL130C	117
YJL131C	27
YJL132W	172
YJL133C-A	28
YJL133W	13
YJL134W	32
YJL135W	65
YJL136C	13
YJL136W-A	15

YJL137C	166
YJL138C	19
YJL139C	11
YJL140W	59
YJL141C	7
YJL142C	126
YJL143W	43
YJL144W	38
YJL145W	332
YJL146W	266
YJL147C	29
YJL148W	32
YJL149W	12
YJL150W	68
YJL151C	8
YJL152W	9
YJL153C	41
YJL154C	7
YJL155C	67
YJL156C	12
YJL156W-A	35
YJL157C	141
YJL158C	21
YJL159W	173
YJL160C	62
YJL161W	578
YJL162C	130
YJL163C	6
YJL164C	163
YJL165C	79
YJL166W	116
YJL167W	10
YJL168C	17
YJL169W	91
YJL170C	3
YJL171C	153
YJL172W	80
YJL173C	40
YJL174W	12
YJL175W	48
YJL176C	42
YJL177W	107
YJL178C	1
YJL179W	40
YJL180C	36
YJL181W	2038
YJL182C	271
YJL183W	764
YJL184W	17
YJL185C	9
YJL186W	24
YJL187C	26
YJL188C	17
YJL189W	306
YJL190C	52

YJL191W	55
YJL192C	42
YJL193W	39
YJL194W	42
YJL195C	18
YJL196C	62
YJL197C-A	94
YJL197W	830
YJL198W	24
YJL199C	45
YJL200C	103
YJL201W	157
YJL202C	323
YJL203W	36
YJL204C	9
YJL205C	516
YJL206C	32
YJL207C	12
YJL208C	7
YJL209W	46
YJL210W	19
YJL211C	171
YJL212C	273
YJL213W	101
YJL214W	13
YJL215C	35
YJL216C	29
YJL217W	35
YJL218W	56
YJL219W	66
YJL220W	307
YJL221C	41
YJL222W	244
YJL222W-A	12
YJL222W-B	21
YJL223C	26
YJL225C	73
YJL225W-A	35
YJR001W	126
YJR002W	227
YJR003C	37
YJR004C	53
YJR005C-A	66
YJR005W	8
YJR006W	117
YJR007W	70
YJR008W	130
YJR009C	29
YJR010C-A	165
YJR010W	13
YJR011C	145
YJR012C	251
YJR013W	13
YJR014W	24
YJR015W	45

YJR016C	92
YJR017C	65
YJR018W	139
YJR019C	64
YJR020W	54
YJR021C	93
YJR022W	355
YJR023C	70
YJR024C	38
YJR025C	39
YJR026W	14
YJR027W	130
YJR028W	56
YJR029W	118
YJR030C	233
YJR031C	51
YJR032W	45
YJR033C	78
YJR034W	211
YJR035W	45
YJR036C	151
YJR037W	37
YJR038C	128
YJR039W	9
YJR040W	159
YJR041C	130
YJR042W	59
YJR043C	114
YJR044C	103
YJR045C	39
YJR046W	39
YJR047C	75
YJR048W	249
YJR049C	255
YJR050W	53
YJR051W	17
YJR052W	44
YJR053W	61
YJR054W	105
YJR055W	293
YJR056C	45
YJR057W	117
YJR058C	18
YJR059W	52
YJR060W	84
YJR061W	53
YJR062C	32
YJR063W	112
YJR064W	95
YJR065C	10
YJR066W	117
YJR067C	17
YJR068W	27
YJR069C	24
YJR070C	70

YJR071W	499
YJR072C	32
YJR073C	26
YJR074W	109
YJR075W	45
YJR076C	47
YJR077C	51
YJR078W	64
YJR079W	34
YJR080C	45
YJR082C	30
YJR083C	35
YJR084W	224
YJR085C	50
YJR086W	16
YJR087W	105
YJR088C	199
YJR089W	190
YJR090C	11
YJR091C	20
YJR092W	140
YJR093C	116
YJR094C	21
YJR094W-A	9
YJR095W	15
YJR096W	67
YJR097W	54
YJR098C	55
YJR099W	75
YJR100C	38
YJR101W	14
YJR102C	27
YJR103W	11
YJR104C	2
YJR105W	137
YJR106W	8
YJR107W	30
YJR108W	56
YJR109C	47
YJR110W	198
YJR111C	3
YJR112W	126
YJR112W-A	116
YJR113C	7
YJR114W	8
YJR115W	19
YJR116W	124
YJR117W	65
YJR118C	229
YJR119C	236
YJR120W	8
YJR121W	79
YJR122W	17
YJR123W	82
YJR124C	28

YJR125C	42
YJR126C	81
YJR127C	61
YJR128W	119
YJR129C	122
YJR130C	33
YJR131W	109
YJR132W	65
YJR133W	43
YJR134C	34
YJR135C	75
YJR135W-A	12
YJR136C	40
YJR137C	88
YJR138W	17
YJR139C	61
YJR140C	22
YJR140W-A	37
YJR141W	60
YJR142W	101
YJR143C	13
YJR144W	63
YJR145C	47
YJR146W	64
YJR147W	19
YJR148W	17
YJR149W	270
YJR150C	1155
YJR151C	168
YJR151W-A	662
YJR152W	391
YJR153W	8
YJR154W	106
YJR155W	12
YJR156C	7
YJR157W	25
YJR158W	92
YJR159W	127
YJR160C	2
YJR161C	14
YJR162C	871
YKL001C	43
YKL002W	98
YKL003C	12
YKL004W	170
YKL005C	88
YKL006C-A	79
YKL006W	17
YKL007W	292
YKL008C	2
YKL009W	37
YKL010C	175
YKL011C	16
YKL012W	17
YKL013C	31

YKL014C	3
YKL015W	97
YKL016C	71
YKL017C	23
YKL018C-A	21
YKL018W	61
YKL019W	48
YKL020C	101
YKL021C	35
YKL022C	4
YKL023C-A	193
YKL023W	8
YKL024C	20
YKL025C	32
YKL026C	4
YKL027W	19
YKL028W	3
YKL029C	194
YKL030W	7
YKL031W	85
YKL032C	25
YKL033W	57
YKL033W-A	14
YKL034W	23
YKL035W	41
YKL036C	149
YKL037W	72
YKL038W	290
YKL039W	81
YKL040C	8
YKL041W	52
YKL042W	12
YKL043W	100
YKL044W	108
YKL045W	218
YKL046C	230
YKL047W	28
YKL048C	20
YKL049C	30
YKL050C	21
YKL051W	13
YKL052C	12
YKL053C-A	72
YKL053W	288
YKL054C	215
YKL055C	24
YKL056C	407
YKL057C	329
YKL058W	20
YKL059C	34
YKL060C	271
YKL061W	40
YKL062W	130
YKL063C	21
YKL064W	13

YKL065C	2
YKL065W-A	76
YKL066W	112
YKL067W	8
YKL068W	122
YKL068W-A	126
YKL069W	360
YKL070W	97
YKL071W	9
YKL072W	31
YKL073W	193
YKL074C	8
YKL075C	44
YKL076C	19
YKL077W	8
YKL078W	51
YKL079W	163
YKL080W	4
YKL081W	15
YKL082C	16
YKL083W	45
YKL084W	71
YKL085W	279
YKL086W	27
YKL087C	202
YKL088W	5
YKL089W	567
YKL090W	15
YKL091C	289
YKL092C	32
YKL093W	43
YKL094W	65
YKL095W	189
YKL096C-B	37
YKL096W	82
YKL096W-A	29
YKL097C	41
YKL098W	25
YKL099C	80
YKL100C	61
YKL100W-A	48
YKL101W	8
YKL102C	241
YKL103C	20
YKL104C	16
YKL105C	25
YKL106C-A	24
YKL106W	21
YKL107W	32
YKL108W	4
YKL109W	68
YKL110C	11
YKL111C	11
YKL112W	226
YKL113C	23

YKL114C	78
YKL115C	289
YKL116C	15
YKL117W	86
YKL118W	36
YKL119C	10
YKL120W	26
YKL121W	172
YKL122C	175
YKL123W	254
YKL124W	76
YKL125W	20
YKL126W	21
YKL127W	254
YKL128C	66
YKL129C	8
YKL130C	230
YKL131W	6
YKL132C	60
YKL133C	25
YKL134C	122
YKL135C	87
YKL136W	69
YKL137W	75
YKL138C	25
YKL138C-A	48
YKL139W	16
YKL140W	49
YKL141W	114
YKL142W	62
YKL143W	10
YKL144C	300
YKL145W	123
YKL145W-A	48
YKL146W	14
YKL147C	54
YKL148C	24
YKL149C	401
YKL150W	16
YKL151C	59
YKL152C	24
YKL153W	142
YKL154W	93
YKL155C	23
YKL156C-A	53
YKL156W	147
YKL157W	1
YKL159C	20
YKL160W	39
YKL161C	112
YKL162C	43
YKL162C-A	308
YKL163W	16
YKL164C	21
YKL165C	55

YKL165C-A	216
YKL166C	194
YKL167C	82
YKL168C	20
YKL169C	88
YKL170W	31
YKL171W	301
YKL172W	23
YKL173W	82
YKL174C	178
YKL175W	20
YKL176C	159
YKL177W	119
YKL178C	43
YKL179C	72
YKL180W	305
YKL181W	452
YKL182W	294
YKL183C-A	144
YKL183W	239
YKL184W	110
YKL185W	54
YKL186C	20
YKL187C	87
YKL188C	2
YKL189W	3
YKL190W	149
YKL191W	25
YKL192C	7
YKL193C	123
YKL194C	14
YKL195W	42
YKL196C	15
YKL197C	17
YKL198C	24
YKL201C	20
YKL202W	112
YKL203C	295
YKL204W	8
YKL205W	72
YKL206C	131
YKL207W	430
YKL208W	9
YKL209C	73
YKL210W	10
YKL211C	35
YKL212W	106
YKL213C	39
YKL214C	88
YKL215C	32
YKL216W	23
YKL217W	151
YKL218C	582
YKL219W	27
YKL220C	48

YKL221W	9
YKL222C	92
YKL223W	30
YKL224C	67
YKL225W	36
YKR001C	462
YKR002W	219
YKR003W	64
YKR004C	61
YKR005C	109
YKR006C	11
YKR007W	65
YKR008W	16
YKR009C	157
YKR010C	297
YKR011C	29
YKR012C	238
YKR013W	15
YKR014C	29
YKR015C	21
YKR016W	14
YKR017C	45
YKR018C	117
YKR019C	96
YKR020W	68
YKR021W	56
YKR022C	15
YKR023W	20
YKR024C	76
YKR025W	5
YKR026C	47
YKR027W	157
YKR028W	38
YKR029C	11
YKR030W	23
YKR031C	119
YKR032W	33
YKR033C	17
YKR034W	71
YKR035C	26
YKR035W-A	111
YKR036C	10
YKR037C	16
YKR038C	27
YKR039W	29
YKR040C	10
YKR041W	21
YKR042W	4
YKR043C	112
YKR044W	53
YKR045C	16
YKR046C	23
YKR047W	10
YKR048C	25
YKR049C	150

YKR050W	15
YKR051W	40
YKR052C	118
YKR053C	150
YKR054C	33
YKR055W	49
YKR056W	93
YKR057W	32
YKR058W	7
YKR059W	50
YKR060W	73
YKR061W	74
YKR062W	61
YKR063C	142
YKR064W	55
YKR065C	35
YKR066C	89
YKR067W	128
YKR068C	42
YKR069W	66
YKR070W	443
YKR071C	25
YKR072C	74
YKR073C	24
YKR074W	47
YKR075C	124
YKR075W-A	25
YKR076W	164
YKR077W	42
YKR078W	305
YKR079C	12
YKR080W	72
YKR081C	262
YKR082W	30
YKR083C	33
YKR084C	14
YKR085C	3
YKR086W	38
YKR087C	335
YKR088C	99
YKR089C	12
YKR090W	78
YKR091W	60
YKR092C	35
YKR093W	42
YKR094C	58
YKR095W	78
YKR095W-A	77
YKR096W	62
YKR097W	46
YKR098C	14
YKR099W	71
YKR100C	22
YKR101W	76
YKR102W	129

YKR103W	19
YKR104W	6
YKR105C	31
YKR106W	34
YLL001W	227
YLL002W	21
YLL003W	55
YLL004W	69
YLL005C	92
YLL006W	481
YLL006W-A	241
YLL007C	28
YLL008W	6
YLL009C	5
YLL010C	39
YLL011W	32
YLL012W	11
YLL013C	29
YLL014W	282
YLL015W	15
YLL016W	92
YLL017W	27
YLL018C	229
YLL018C-A	64
YLL019C	17
YLL019W-A	14
YLL020C	58
YLL021W	63
YLL022C	13
YLL023C	10
YLL024C	29
YLL025W	44
YLL026W	53
YLL027W	14
YLL028W	7
YLL029W	13
YLL030C	5
YLL031C	23
YLL032C	73
YLL033W	72
YLL034C	21
YLL035W	7
YLL036C	17
YLL037W	52
YLL038C	10
YLL039C	21
YLL040C	19
YLL041C	193
YLL042C	42
YLL043W	2
YLL044W	3
YLL045C	10
YLL046C	23
YLL047W	9
YLL048C	46

YLL049W	21
YLL050C	151
YLL051C	19
YLL052C	106
YLL053C	29
YLL054C	145
YLL055W	16
YLL056C	33
YLL057C	12
YLL058W	568
YLL059C	16
YLL060C	61
YLL061W	29
YLL062C	32
YLL063C	474
YLL064C	492
YLL065W	59
YLL066C	40
YLL066W-A	25
YLL066W-B	9
YLL067C	89
YLL067W-A	100
YLR001C	52
YLR002C	266
YLR003C	50
YLR004C	36
YLR005W	69
YLR006C	90
YLR007W	22
YLR008C	131
YLR009W	62
YLR010C	49
YLR011W	54
YLR012C	26
YLR013W	85
YLR014C	15
YLR015W	59
YLR016C	93
YLR017W	124
YLR018C	63
YLR019W	76
YLR020C	71
YLR021W	47
YLR022C	15
YLR023C	121
YLR024C	6
YLR025W	46
YLR026C	104
YLR027C	244
YLR028C	55
YLR029C	54
YLR030W	153
YLR031W	36
YLR032W	18
YLR033W	64

YLR034C	66
YLR035C	11
YLR035C-A	104
YLR036C	4
YLR037C	60
YLR038C	6
YLR039C	12
YLR040C	77
YLR041W	73
YLR042C	51
YLR043C	543
YLR044C	152
YLR045C	106
YLR046C	48
YLR047C	95
YLR048W	43
YLR049C	25
YLR050C	112
YLR051C	16
YLR052W	48
YLR053C	35
YLR054C	17
YLR055C	21
YLR056W	76
YLR057W	73
YLR058C	42
YLR059C	5
YLR060W	105
YLR061W	66
YLR062C	45
YLR063W	20
YLR064W	35
YLR065C	134
YLR066W	400
YLR067C	82
YLR068W	360
YLR069C	41
YLR070C	16
YLR071C	113
YLR072W	71
YLR073C	97
YLR074C	103
YLR075W	65
YLR076C	28
YLR077W	42
YLR078C	59
YLR079W	115
YLR080W	146
YLR081W	13
YLR082C	173
YLR083C	46
YLR084C	14
YLR085C	93
YLR086W	4
YLR087C	449

YLR088W	52
YLR089C	8
YLR090W	3
YLR091W	19
YLR092W	56
YLR093C	1377
YLR094C	55
YLR095C	13
YLR096W	27
YLR097C	309
YLR098C	11
YLR099C	4
YLR099W-A	31
YLR100W	52
YLR101C	238
YLR102C	23
YLR103C	78
YLR104W	149
YLR105C	183
YLR106C	163
YLR107W	59
YLR108C	79
YLR109W	11
YLR110C	2
YLR111W	90
YLR112W	198
YLR113W	24
YLR114C	62
YLR115W	62
YLR116W	88
YLR117C	10
YLR118C	146
YLR119W	15
YLR120C	19
YLR120W-A	29
YLR121C	152
YLR122C	277
YLR123C	348
YLR124W	6
YLR125W	131
YLR126C	68
YLR127C	72
YLR128W	59
YLR129W	206
YLR130C	209
YLR131C	34
YLR132C	58
YLR133W	20
YLR134W	87
YLR135W	80
YLR136C	52
YLR137W	56
YLR138W	19
YLR139C	437
YLR140W	21

YLR141W	124
YLR142W	19
YLR143W	28
YLR144C	12
YLR145W	112
YLR146C	153
YLR146W-A	20
YLR147C	17
YLR148W	101
YLR149C	30
YLR149C-A	21
YLR150W	465
YLR151C	107
YLR152C	28
YLR153C	6
YLR154C	33
YLR154C-G	16
YLR154C-H	67
YLR154W-A	96
YLR154W-B	31
YLR154W-C	73
YLR154W-E	27
YLR154W-F	113
YLR155C	65
YLR156C-A	256
YLR156W	280
YLR157C	49
YLR157C-A	13
YLR157C-B	115
YLR157C-C	70
YLR157W-D	481
YLR157W-E	3
YLR158C	9
YLR159C-A	635
YLR159W	15
YLR160C	4
YLR161W	29
YLR162W	66
YLR162W-A	62
YLR163C	26
YLR163W-A	163
YLR164W	39
YLR165C	46
YLR166C	38
YLR167W	29
YLR168C	185
YLR169W	137
YLR170C	93
YLR171W	155
YLR172C	163
YLR173W	7
YLR174W	9
YLR175W	834
YLR176C	388
YLR177W	98

YLR178C	167
YLR179C	55
YLR180W	32
YLR181C	25
YLR182W	8
YLR183C	29
YLR184W	65
YLR185W	264
YLR186W	6
YLR187W	241
YLR188W	9
YLR189C	162
YLR190W	22
YLR191W	13
YLR192C	717
YLR193C	161
YLR194C	34
YLR195C	193
YLR196W	36
YLR197W	106
YLR198C	43
YLR199C	152
YLR200W	96
YLR201C	146
YLR202C	40
YLR203C	750
YLR204W	38
YLR205C	46
YLR206W	71
YLR207W	17
YLR208W	33
YLR209C	48
YLR210W	77
YLR211C	111
YLR212C	19
YLR213C	59
YLR214W	18
YLR215C	14
YLR216C	158
YLR217W	81
YLR218C	133
YLR219W	4
YLR220W	5
YLR221C	31
YLR222C	41
YLR222C-A	57
YLR223C	11
YLR224W	244
YLR225C	37
YLR226W	44
YLR227C	4
YLR227W-A	62
YLR227W-B	151
YLR228C	63
YLR229C	37

YLR230W	78
YLR231C	70
YLR232W	104
YLR233C	89
YLR234W	39
YLR235C	36
YLR236C	16
YLR237W	83
YLR238W	50
YLR239C	111
YLR240W	55
YLR241W	30
YLR242C	203
YLR243W	79
YLR244C	52
YLR245C	213
YLR246W	140
YLR247C	126
YLR248W	165
YLR249W	42
YLR250W	8
YLR251W	12
YLR252W	30
YLR253W	1
YLR254C	12
YLR255C	64
YLR256W	156
YLR256W-A	27
YLR257W	19
YLR258W	33
YLR259C	50
YLR260W	113
YLR261C	13
YLR262C	20
YLR262C-A	180
YLR263W	41
YLR264C-A	70
YLR264W	211
YLR265C	125
YLR266C	113
YLR267W	10
YLR268W	58
YLR269C	17
YLR270W	143
YLR271W	26
YLR272C	447
YLR273C	38
YLR274W	16
YLR275W	22
YLR276C	18
YLR277C	10
YLR278C	39
YLR279W	101
YLR280C	161
YLR281C	4

YLR282C	26
YLR283W	55
YLR284C	94
YLR285C-A	34
YLR285W	7
YLR286C	27
YLR286W-A	616
YLR287C	27
YLR287C-A	65
YLR288C	51
YLR289W	47
YLR290C	43
YLR291C	22
YLR292C	53
YLR293C	80
YLR294C	21
YLR295C	37
YLR296W	21
YLR297W	298
YLR298C	20
YLR299C-A	22
YLR299W	57
YLR300W	79
YLR301W	132
YLR302C	35
YLR303W	18
YLR304C	6
YLR305C	66
YLR306W	43
YLR307C-A	29
YLR307W	8
YLR308W	37
YLR309C	191
YLR310C	19
YLR311C	82
YLR312C	40
YLR312W-A	13
YLR313C	1
YLR314C	9
YLR315W	22
YLR316C	16
YLR317W	20
YLR318W	52
YLR319C	77
YLR320W	17
YLR321C	54
YLR322W	157
YLR323C	65
YLR324W	44
YLR325C	101
YLR326W	23
YLR327C	20
YLR328W	34
YLR329W	89
YLR330W	51

YLR331C	62
YLR332W	81
YLR333C	112
YLR334C	134
YLR335W	5
YLR336C	47
YLR337C	28
YLR338W	16
YLR339C	256
YLR340W	200
YLR341W	158
YLR342W	216
YLR342W-A	14
YLR343W	34
YLR344W	116
YLR345W	13
YLR346C	15
YLR347C	102
YLR347W-A	285
YLR348C	59
YLR349W	3
YLR350W	43
YLR351C	39
YLR352W	21
YLR353W	30
YLR354C	15
YLR355C	25
YLR356W	42
YLR357W	16
YLR358C	3
YLR359W	120
YLR360W	57
YLR361C	25
YLR361C-A	12
YLR362W	1345
YLR363C	31
YLR363W-A	14
YLR364C-A	33
YLR364W	9
YLR365W	15
YLR366W	68
YLR367W	11
YLR368W	203
YLR369W	415
YLR370C	103
YLR371W	70
YLR372W	29
YLR373C	104
YLR374C	110
YLR375W	6
YLR376C	12
YLR377C	102
YLR378C	6
YLR379W	43
YLR380W	28

YLR381W	167
YLR382C	57
YLR383W	62
YLR384C	22
YLR385C	106
YLR386W	98
YLR387C	149
YLR388W	185
YLR389C	77
YLR390W	93
YLR390W-A	19
YLR392C	29
YLR393W	120
YLR394W	230
YLR395C	18
YLR396C	43
YLR397C	17
YLR398C	17
YLR399C	195
YLR399W-A	29
YLR400W	56
YLR401C	67
YLR402W	21
YLR403W	254
YLR404W	3
YLR405W	9
YLR406C	177
YLR406C-A	23
YLR407W	35
YLR408C	102
YLR409C	115
YLR410W	403
YLR410W-A	38
YLR410W-B	133
YLR411W	45
YLR412C-A	149
YLR412W	117
YLR413W	7
YLR414C	341
YLR415C	12
YLR416C	20
YLR417W	173
YLR418C	75
YLR419W	28
YLR420W	23
YLR421C	28
YLR422W	31
YLR423C	45
YLR424W	50
YLR425W	25
YLR426W	199
YLR427W	95
YLR428C	13
YLR429W	7
YLR430W	11

YLR431C	16
YLR432W	615
YLR433C	40
YLR434C	8
YLR435W	27
YLR436C	50
YLR437C	80
YLR437C-A	15
YLR438C-A	9
YLR438W	88
YLR439W	53
YLR440C	27
YLR441C	122
YLR442C	22
YLR443W	25
YLR444C	119
YLR445W	6
YLR446W	63
YLR447C	214
YLR448W	210
YLR449W	33
YLR450W	51
YLR451W	41
YLR452C	84
YLR453C	97
YLR454W	8
YLR455W	53
YLR456W	66
YLR457C	227
YLR458W	29
YLR459W	20
YLR460C	138
YLR461W	453
YLR462W	58
YLR463C	104
YLR464W	34
YLR465C	28
YLR466C-A	122
YLR466C-B	85
YLR466W	4
YLR467C-A	13
YLR467W	65
YML001W	163
YML002W	447
YML003W	15
YML004C	14
YML005W	24
YML006C	37
YML007C-A	15
YML007W	57
YML008C	18
YML009C	53
YML009C-A	57
YML009W-B	12
YML010W	135

YML011C	15
YML012C-A	5
YML012W	14
YML013W	172
YML014W	20
YML015C	294
YML016C	19
YML017W	54
YML018C	72
YML019W	70
YML020W	16
YML021C	443
YML022W	44
YML023C	23
YML024W	49
YML025C	52
YML026C	46
YML027W	72
YML028W	19
YML029W	59
YML030W	55
YML031C-A	21
YML031W	86
YML032C	64
YML034C-A	28
YML034W	600
YML035C	99
YML036W	43
YML037C	46
YML038C	139
YML039W	20
YML040W	26
YML041C	70
YML042W	93
YML043C	270
YML045W	37
YML045W-A	953
YML046W	17
YML047C	19
YML047W-A	16
YML048W	8
YML049C	71
YML050W	136
YML051W	413
YML052W	17
YML053C	54
YML054C	29
YML054C-A	499
YML055W	9
YML056C	32
YML057C-A	11
YML057W	57
YML058W	91
YML058W-A	514
YML059C	57

YML060W	39
YML061C	442
YML062C	252
YML063W	47
YML064C	28
YML065W	21
YML066C	16
YML067C	21
YML068W	14
YML069W	75
YML070W	21
YML071C	74
YML072C	73
YML073C	26
YML074C	12
YML075C	64
YML076C	8
YML077W	120
YML078W	112
YML079W	31
YML080W	16
YML081C-A	71
YML081W	74
YML082W	28
YML083C	141
YML084W	102
YML085C	22
YML086C	1
YML087C	28
YML088W	8
YML089C	5
YML090W	123
YML091C	23
YML092C	221
YML093W	41
YML094C-A	6
YML094W	154
YML095C	493
YML096W	59
YML097C	10
YML098W	38
YML099C	143
YML099W-A	37
YML100W	361
YML100W-A	18
YML101C	36
YML101C-A	1239
YML102W	52
YML103C	104
YML104C	26
YML105C	179
YML106W	89
YML107C	286
YML108W	27
YML109W	10

YML110C	6
YML111W	59
YML112W	1
YML113W	10
YML114C	53
YML115C	6
YML116W	259
YML116W-A	32
YML117W	13
YML118W	54
YML119W	18
YML120C	19
YML121W	143
YML122C	9
YML123C	30
YML124C	95
YML125C	7
YML126C	295
YML127W	225
YML128C	127
YML129C	38
YML130C	83
YML131W	18
YML132W	58
YML133C	18
YML133W-A	160
YML133W-B	89
YMR001C	49
YMR001C-A	53
YMR002W	398
YMR003W	61
YMR004W	33
YMR005W	88
YMR006C	476
YMR007W	130
YMR008C	323
YMR009W	32
YMR010W	184
YMR011W	19
YMR012W	92
YMR013C	32
YMR013C-A	119
YMR013W-A	54
YMR014W	50
YMR015C	160
YMR016C	7
YMR017W	44
YMR018W	3
YMR019W	63
YMR020W	4
YMR021C	10
YMR022W	34
YMR023C	55
YMR024W	6
YMR025W	108

YMR026C	8
YMR027W	22
YMR028W	795
YMR029C	22
YMR030W	30
YMR030W-A	29
YMR031C	8
YMR031W-A	9
YMR032W	13
YMR033W	58
YMR034C	626
YMR035W	36
YMR036C	42
YMR037C	46
YMR038C	45
YMR039C	40
YMR040W	13
YMR041C	74
YMR042W	204
YMR043W	180
YMR044W	113
YMR045C	37
YMR046C	21
YMR046W-A	4
YMR047C	70
YMR048W	19
YMR049C	41
YMR050C	31
YMR051C	51
YMR052C-A	38
YMR052W	131
YMR053C	9
YMR054W	16
YMR055C	108
YMR056C	58
YMR057C	186
YMR058W	43
YMR059W	21
YMR060C	9
YMR061W	80
YMR062C	1281
YMR063W	10
YMR064W	32
YMR065W	544
YMR066W	120
YMR067C	15
YMR068W	93
YMR069W	41
YMR070W	48
YMR071C	238
YMR072W	388
YMR073C	30
YMR074C	43
YMR075C-A	299
YMR075W	20

YMR076C	502
YMR077C	142
YMR078C	113
YMR079W	7
YMR080C	44
YMR081C	59
YMR082C	46
YMR083W	63
YMR084W	44
YMR085W	18
YMR086C-A	39
YMR086W	12
YMR087W	6
YMR088C	14
YMR089C	10
YMR090W	127
YMR091C	39
YMR092C	86
YMR093W	99
YMR094W	23
YMR095C	44
YMR096W	177
YMR097C	10
YMR098C	344
YMR099C	16
YMR100W	36
YMR101C	40
YMR102C	46
YMR103C	24
YMR104C	24
YMR105C	25
YMR105W-A	32
YMR106C	28
YMR107W	9
YMR108W	47
YMR109W	115
YMR110C	38
YMR111C	15
YMR112C	92
YMR113W	7
YMR114C	31
YMR115W	3
YMR116C	10
YMR117C	1275
YMR118C	161
YMR119W	49
YMR119W-A	75
YMR120C	18
YMR121C	5
YMR122C	218
YMR122W-A	36
YMR123W	15
YMR124W	226
YMR125W	203
YMR126C	13

YMR127C	174
YMR128W	521
YMR129W	177
YMR130W	38
YMR131C	10
YMR132C	132
YMR133W	35
YMR134W	150
YMR135C	90
YMR135W-A	221
YMR136W	11
YMR137C	39
YMR138W	176
YMR139W	197
YMR140W	192
YMR141C	42
YMR141W-A	23
YMR142C	51
YMR143W	177
YMR144W	16
YMR145C	63
YMR146C	14
YMR147W	70
YMR148W	109
YMR149W	27
YMR150C	20
YMR151W	79
YMR152W	21
YMR153C-A	28
YMR153W	145
YMR154C	9
YMR155W	133
YMR156C	30
YMR157C	42
YMR158C-A	153
YMR158W	24
YMR158W-B	18
YMR159C	36
YMR160W	20
YMR161W	44
YMR162C	78
YMR163C	13
YMR164C	7
YMR165C	24
YMR166C	63
YMR167W	35
YMR168C	50
YMR169C	8
YMR170C	34
YMR171C	145
YMR172C-A	15
YMR172W	18
YMR173W	112
YMR173W-A	19
YMR174C	66

YMR175W	4
YMR175W-A	51
YMR176W	72
YMR177W	384
YMR178W	153
YMR179W	31
YMR180C	114
YMR181C	32
YMR182C	18
YMR182W-A	137
YMR183C	79
YMR184W	19
YMR185W	41
YMR186W	56
YMR187C	180
YMR188C	23
YMR189W	55
YMR190C	288
YMR191W	380
YMR192W	40
YMR193C-A	97
YMR193W	4
YMR194C-A	48
YMR194C-B	103
YMR194W	33
YMR195W	47
YMR196W	23
YMR197C	68
YMR198W	15
YMR199W	19
YMR200W	44
YMR201C	59
YMR202W	59
YMR203W	339
YMR204C	13
YMR205C	96
YMR206W	26
YMR207C	36
YMR208W	23
YMR209C	69
YMR210W	427
YMR211W	7
YMR212C	29
YMR213W	48
YMR214W	22
YMR215W	4
YMR216C	44
YMR217W	9
YMR218C	13
YMR219W	73
YMR220W	276
YMR221C	10
YMR222C	60
YMR223W	33
YMR224C	16

YMR225C	94
YMR226C	38
YMR227C	45
YMR228W	84
YMR229C	45
YMR230W	373
YMR230W-A	809
YMR231W	17
YMR232W	226
YMR233W	36
YMR234W	12
YMR235C	3241
YMR236W	11
YMR237W	117
YMR238W	120
YMR239C	8
YMR240C	360
YMR241W	139
YMR242C	61
YMR242W-A	644
YMR243C	94
YMR244C-A	334
YMR244W	41
YMR245W	11
YMR246W	78
YMR247C	35
YMR247W-A	215
YMR250W	27
YMR251W	38
YMR251W-A	75
YMR252C	22
YMR253C	3
YMR254C	8
YMR255W	37
YMR256C	123
YMR257C	820
YMR258C	62
YMR259C	19
YMR260C	14
YMR261C	9
YMR262W	17
YMR263W	9
YMR264W	6
YMR265C	306
YMR266W	7
YMR267W	61
YMR268C	15
YMR269W	70
YMR270C	43
YMR271C	176
YMR272C	384
YMR272W-A	27
YMR272W-B	30
YMR273C	42
YMR274C	74

YMR275C	418
YMR276W	20
YMR277W	76
YMR278W	11
YMR279C	256
YMR280C	12
YMR281W	68
YMR282C	133
YMR283C	17
YMR284W	2
YMR285C	94
YMR286W	223
YMR287C	132
YMR288W	246
YMR289W	24
YMR290C	44
YMR290W-A	54
YMR291W	10
YMR292W	105
YMR293C	94
YMR294W	48
YMR294W-A	13
YMR295C	222
YMR296C	37
YMR297W	28
YMR298W	215
YMR299C	23
YMR300C	104
YMR301C	30
YMR302C	13
YMR303C	314
YMR304C-A	24
YMR304W	35
YMR305C	7
YMR306C-A	25
YMR306W	29
YMR307C-A	19
YMR307W	32
YMR308C	15
YMR309C	17
YMR310C	39
YMR311C	94
YMR312W	90
YMR313C	19
YMR314W	15
YMR315W	99
YMR315W-A	997
YMR316C-A	111
YMR316C-B	23
YMR316W	113
YMR317W	87
YMR318C	37
YMR319C	17
YMR320W	14
YMR321C	37

YMR322C	106
YMR323W	82
YMR324C	9
YMR325W	140
YMR326C	49
YNL001W	12
YNL002C	119
YNL003C	33
YNL004W	126
YNL005C	96
YNL006W	126
YNL007C	53
YNL008C	53
YNL009W	45
YNL010W	14
YNL011C	39
YNL012W	6
YNL013C	24
YNL014W	20
YNL015W	150
YNL016W	11
YNL017C	10
YNL018C	21
YNL019C	156
YNL020C	779
YNL021W	25
YNL022C	12
YNL023C	44
YNL024C	176
YNL024C-A	31
YNL025C	14
YNL026W	346
YNL027W	42
YNL028W	8
YNL029C	15
YNL030W	122
YNL031C	21
YNL032W	7
YNL033W	49
YNL034W	2
YNL035C	6
YNL036W	79
YNL037C	33
YNL038W	12
YNL039W	41
YNL040W	105
YNL041C	19
YNL042W	143
YNL042W-B	366
YNL043C	40
YNL044W	59
YNL045W	11
YNL046W	65
YNL047C	21
YNL048W	45

YNL049C	74
YNL050C	90
YNL051W	21
YNL052W	71
YNL053W	50
YNL054W	78
YNL054W-A	14
YNL054W-B	123
YNL055C	75
YNL056W	74
YNL057W	19
YNL058C	5
YNL059C	35
YNL061W	25
YNL062C	54
YNL063W	13
YNL064C	44
YNL065W	75
YNL066W	77
YNL067W	35
YNL067W-A	139
YNL067W-B	3
YNL068C	8
YNL069C	55
YNL070W	46
YNL071W	86
YNL072W	34
YNL073W	287
YNL074C	47
YNL075W	22
YNL076W	11
YNL077W	165
YNL078W	41
YNL079C	98
YNL080C	37
YNL081C	91
YNL082W	57
YNL083W	170
YNL084C	47
YNL085W	8
YNL086W	522
YNL087W	68
YNL088W	475
YNL089C	135
YNL090W	43
YNL091W	8
YNL092W	26
YNL093W	96
YNL094W	5
YNL095C	14
YNL096C	46
YNL097C	42
YNL097C-B	87
YNL097W-A	94
YNL098C	84

YNL099C	126
YNL100W	54
YNL101W	44
YNL102W	48
YNL103W	27
YNL103W-A	22
YNL104C	229
YNL105W	15
YNL106C	67
YNL107W	100
YNL108C	13
YNL109W	157
YNL110C	60
YNL111C	23
YNL112W	126
YNL113W	69
YNL114C	142
YNL115C	20
YNL116W	76
YNL117W	9
YNL118C	42
YNL119W	85
YNL120C	4
YNL121C	82
YNL122C	106
YNL123W	32
YNL124W	7
YNL125C	221
YNL126W	29
YNL127W	5
YNL128W	110
YNL129W	57
YNL130C	66
YNL130C-A	39
YNL131W	39
YNL132W	30
YNL133C	51
YNL134C	559
YNL135C	388
YNL136W	216
YNL137C	42
YNL138W	94
YNL138W-A	59
YNL139C	39
YNL140C	20
YNL141W	30
YNL142W	18
YNL143C	248
YNL144C	31
YNL144W-A	8
YNL145W	28
YNL146C-A	62
YNL146W	47
YNL147W	35
YNL148C	85

YNL149C	97
YNL150W	19
YNL151C	22
YNL152W	105
YNL153C	22
YNL154C	405
YNL155W	92
YNL156C	210
YNL157W	16
YNL158W	22
YNL159C	79
YNL160W	7
YNL161W	43
YNL162W	49
YNL162W-A	29
YNL163C	303
YNL164C	34
YNL165W	976
YNL166C	6
YNL167C	27
YNL168C	24
YNL169C	9
YNL170W	7
YNL171C	797
YNL172W	339
YNL173C	27
YNL174W	583
YNL175C	37
YNL176C	18
YNL177C	108
YNL178W	252
YNL179C	103
YNL180C	7
YNL181W	8
YNL182C	40
YNL183C	97
YNL184C	73
YNL185C	156
YNL186W	47
YNL187W	175
YNL188W	58
YNL189W	36
YNL190W	18
YNL191W	12
YNL192W	900
YNL193W	19
YNL194C	188
YNL195C	24
YNL196C	72
YNL197C	624
YNL198C	85
YNL199C	318
YNL200C	29
YNL201C	22
YNL202W	42

YNL203C	145
YNL204C	326
YNL205C	767
YNL206C	43
YNL207W	18
YNL208W	55
YNL209W	109
YNL210W	57
YNL211C	39
YNL212W	54
YNL213C	155
YNL214W	288
YNL215W	7
YNL216W	134
YNL217W	28
YNL218W	90
YNL219C	54
YNL220W	345
YNL221C	116
YNL222W	127
YNL223W	130
YNL224C	148
YNL225C	175
YNL226W	53
YNL227C	57
YNL228W	100
YNL229C	61
YNL230C	98
YNL231C	35
YNL232W	192
YNL233W	101
YNL234W	324
YNL235C	484
YNL236W	121
YNL237W	59
YNL238W	50
YNL239W	204
YNL240C	3
YNL241C	11
YNL242W	125
YNL243W	83
YNL244C	93
YNL245C	55
YNL246W	38
YNL247W	560
YNL248C	18
YNL249C	11
YNL250W	43
YNL251C	96
YNL252C	23
YNL253W	30
YNL254C	76
YNL255C	25
YNL256W	12
YNL257C	29

YNL258C	15
YNL259C	24
YNL260C	27
YNL261W	39
YNL262W	28
YNL263C	17
YNL264C	32
YNL265C	122
YNL266W	23
YNL267W	276
YNL268W	108
YNL269W	238
YNL270C	9
YNL271C	25
YNL272C	29
YNL273W	113
YNL274C	15
YNL275W	4
YNL276C	86
YNL277W	2
YNL277W-A	15
YNL278W	29
YNL279W	29
YNL280C	472
YNL281W	255
YNL282W	25
YNL283C	43
YNL284C	57
YNL284C-A	36
YNL284C-B	27
YNL285W	13
YNL286W	3
YNL287W	73
YNL288W	423
YNL289W	37
YNL290W	16
YNL291C	39
YNL292W	22
YNL293W	15
YNL294C	186
YNL295W	19
YNL296W	80
YNL297C	202
YNL298W	111
YNL299W	185
YNL300W	23
YNL301C	124
YNL302C	5
YNL303W	9
YNL304W	52
YNL305C	61
YNL306W	185
YNL307C	72
YNL308C	187
YNL309W	107

YNL310C	29
YNL311C	32
YNL312W	11
YNL313C	16
YNL314W	23
YNL315C	51
YNL316C	79
YNL317W	1828
YNL318C	98
YNL319W	12
YNL320W	6
YNL321W	46
YNL322C	15
YNL323W	504
YNL324W	119
YNL325C	79
YNL326C	3
YNL327W	94
YNL328C	88
YNL329C	662
YNL330C	89
YNL331C	178
YNL332W	119
YNL333W	112
YNL334C	108
YNL335W	37
YNL336W	43
YNL337W	9
YNL338W	26
YNL339C	139
YNL339W-A	171
YNL339W-B	181
YNR001C	26
YNR001W-A	14
YNR002C	167
YNR003C	42
YNR003W-A	24
YNR004W	21
YNR005C	11
YNR006W	115
YNR007C	32
YNR008W	83
YNR009W	42
YNR010W	25
YNR011C	97
YNR012W	11
YNR013C	8
YNR014W	100
YNR015W	184
YNR016C	25
YNR017W	68
YNR018W	4
YNR019W	7
YNR020C	9
YNR021W	80

YNR022C	461
YNR023W	18
YNR024W	98
YNR025C	76
YNR026C	391
YNR027W	70
YNR028W	143
YNR029C	9
YNR030W	12
YNR031C	93
YNR032C-A	82
YNR032W	16
YNR033W	156
YNR034W	67
YNR034W-A	34
YNR035C	5
YNR036C	26
YNR037C	22
YNR038W	43
YNR039C	18
YNR040W	58
YNR041C	24
YNR042W	95
YNR043W	15
YNR044W	20
YNR045W	34
YNR046W	30
YNR047W	88
YNR048W	98
YNR049C	10
YNR050C	21
YNR051C	11
YNR052C	23
YNR053C	463
YNR054C	193
YNR055C	64
YNR056C	23
YNR057C	38
YNR058W	5
YNR059W	140
YNR060W	22
YNR061C	14
YNR062C	157
YNR063W	148
YNR064C	127
YNR065C	67
YNR066C	37
YNR067C	92
YNR068C	8
YNR069C	48
YNR070W	209
YNR071C	17
YNR072W	21
YNR073C	13
YNR074C	6

YNR075C-A	8
YNR075W	85
YNR076W	98
YNR077C	43
YOL001W	125
YOL002C	27
YOL003C	16
YOL004W	50
YOL005C	19
YOL006C	230
YOL007C	10
YOL008W	114
YOL009C	23
YOL010W	243
YOL011W	110
YOL012C	25
YOL013C	93
YOL013W-A	58
YOL013W-B	70
YOL014W	53
YOL015W	232
YOL016C	16
YOL017W	25
YOL018C	351
YOL019W	7
YOL019W-A	47
YOL020W	38
YOL021C	17
YOL022C	109
YOL023W	128
YOL024W	10
YOL025W	83
YOL026C	48
YOL027C	27
YOL028C	465
YOL029C	10
YOL030W	6
YOL031C	6
YOL032W	8
YOL033W	339
YOL034W	23
YOL035C	26
YOL036W	169
YOL037C	13
YOL038C-A	57
YOL038W	87
YOL039W	159
YOL040C	252
YOL041C	16
YOL042W	49
YOL043C	351
YOL044W	35
YOL045W	17
YOL046C	9
YOL047C	62

YOL048C	131
YOL049W	10
YOL050C	36
YOL051W	5
YOL052C	426
YOL052C-A	29
YOL053W	39
YOL054W	89
YOL055C	16
YOL056W	110
YOL057W	807
YOL058W	24
YOL059W	93
YOL060C	45
YOL061W	43
YOL062C	52
YOL063C	34
YOL064C	57
YOL065C	8
YOL066C	24
YOL067C	7
YOL068C	23
YOL069W	1800
YOL070C	5
YOL071W	150
YOL072W	13
YOL073C	19
YOL075C	8
YOL076W	108
YOL077C	41
YOL077W-A	38
YOL078W	186
YOL079W	224
YOL080C	24
YOL081W	247
YOL082W	148
YOL083C-A	210
YOL083W	12
YOL084W	44
YOL085C	119
YOL085W-A	38
YOL086C	31
YOL086W-A	145
YOL087C	23
YOL088C	182
YOL089C	42
YOL090W	17
YOL091W	54
YOL092W	8
YOL093W	95
YOL094C	347
YOL095C	89
YOL096C	243
YOL097C	68
YOL097W-A	71

YOL098C	170
YOL099C	44
YOL100W	9
YOL101C	38
YOL102C	39
YOL103W	65
YOL103W-A	100
YOL103W-B	25
YOL104C	128
YOL105C	35
YOL106W	24
YOL107W	14
YOL108C	14
YOL109W	119
YOL110W	30
YOL111C	42
YOL112W	37
YOL113W	43
YOL114C	51
YOL115W	31
YOL116W	143
YOL117W	38
YOL118C	34
YOL119C	16
YOL120C	174
YOL121C	70
YOL122C	199
YOL123W	5
YOL124C	45
YOL125W	1217
YOL126C	23
YOL127W	123
YOL128C	37
YOL129W	70
YOL130W	5
YOL131W	66
YOL132W	29
YOL133W	5
YOL134C	28
YOL135C	36
YOL136C	108
YOL137W	35
YOL138C	44
YOL139C	90
YOL140W	151
YOL141W	3
YOL142W	81
YOL143C	32
YOL144W	199
YOL145C	121
YOL146W	50
YOL147C	4
YOL148C	114
YOL149W	42
YOL150C	20

YOL151W	87
YOL152W	25
YOL153C	60
YOL154W	103
YOL155C	12
YOL155W-A	45
YOL156W	28
YOL157C	19
YOL158C	236
YOL159C	38
YOL159C-A	87
YOL160W	130
YOL161C	9
YOL162W	11
YOL163W	247
YOL164W	57
YOL164W-A	20
YOL165C	41
YOL166C	52
YOL166W-A	16
YOR001W	23
YOR002W	71
YOR003W	93
YOR004W	11
YOR005C	18
YOR006C	26
YOR007C	148
YOR008C	10
YOR008C-A	5
YOR008W-B	200
YOR009W	20
YOR010C	14
YOR011W	106
YOR011W-A	25
YOR012W	56
YOR013W	80
YOR014W	261
YOR015W	35
YOR016C	45
YOR017W	25
YOR018W	192
YOR019W	275
YOR020C	29
YOR020W-A	14
YOR021C	29
YOR022C	160
YOR023C	16
YOR024W	6
YOR025W	20
YOR026W	53
YOR027W	18
YOR028C	66
YOR029W	4
YOR030W	253
YOR031W	6

YOR032C	40
YOR032W-A	143
YOR033C	12
YOR034C	48
YOR034C-A	99
YOR035C	193
YOR036W	55
YOR037W	29
YOR038C	27
YOR039W	52
YOR040W	185
YOR041C	67
YOR042W	38
YOR043W	84
YOR044W	79
YOR045W	128
YOR046C	53
YOR047C	163
YOR048C	291
YOR049C	84
YOR050C	11
YOR051C	106
YOR052C	26
YOR053W	81
YOR054C	21
YOR055W	19
YOR056C	71
YOR057W	52
YOR058C	115
YOR059C	69
YOR060C	2
YOR061W	5
YOR062C	14
YOR063W	140
YOR064C	16
YOR065W	93
YOR066W	19
YOR067C	13
YOR068C	236
YOR069W	35
YOR070C	31
YOR071C	571
YOR072W	215
YOR072W-A	34
YOR072W-B	53
YOR073W	79
YOR073W-A	6
YOR074C	18
YOR075W	7
YOR076C	344
YOR077W	584
YOR078W	25
YOR079C	16
YOR080W	21
YOR081C	43

YOR082C	24
YOR083W	213
YOR084W	342
YOR085W	15
YOR086C	281
YOR087W	2
YOR089C	106
YOR090C	18
YOR091W	110
YOR092W	78
YOR093C	697
YOR094W	104
YOR095C	234
YOR096W	26
YOR097C	11
YOR098C	70
YOR099W	16
YOR100C	734
YOR101W	141
YOR102W	67
YOR103C	6
YOR104W	129
YOR105W	10
YOR106W	22
YOR107W	8
YOR108C-A	214
YOR108W	128
YOR109W	32
YOR110W	207
YOR111W	13
YOR112W	30
YOR113W	171
YOR114W	80
YOR115C	5
YOR116C	31
YOR117W	39
YOR118W	42
YOR119C	21
YOR120W	70
YOR121C	8
YOR122C	22
YOR123C	65
YOR124C	56
YOR125C	21
YOR126C	83
YOR127W	46
YOR128C	85
YOR129C	69
YOR130C	243
YOR131C	45
YOR132W	5
YOR133W	9
YOR134W	39
YOR135C	15
YOR136W	29

YOR137C	148
YOR138C	146
YOR139C	65
YOR140W	66
YOR141C	65
YOR142W	168
YOR142W-A	14
YOR142W-B	23
YOR143C	73
YOR144C	25
YOR145C	12
YOR146W	57
YOR147W	7
YOR148C	160
YOR149C	54
YOR150W	41
YOR151C	17
YOR152C	6
YOR153W	4
YOR154W	28
YOR155C	130
YOR156C	6
YOR157C	40
YOR158W	27
YOR159C	21
YOR160W	129
YOR161C	11
YOR161C-C	9
YOR161W-A	510
YOR161W-B	6
YOR162C	53
YOR163W	113
YOR164C	219
YOR165W	187
YOR166C	33
YOR167C	44
YOR168W	70
YOR169C	192
YOR170W	3
YOR171C	19
YOR172W	386
YOR173W	91
YOR174W	31
YOR175C	185
YOR176W	59
YOR177C	115
YOR178C	11
YOR179C	16
YOR180C	164
YOR181W	176
YOR182C	41
YOR183W	27
YOR184W	7
YOR185C	56
YOR186C-A	32

YOR186W	47
YOR187W	2
YOR188W	240
YOR189W	49
YOR190W	6
YOR191W	178
YOR192C	45
YOR192C-A	66
YOR192C-B	10
YOR192C-C	12
YOR193W	297
YOR194C	30
YOR195W	22
YOR196C	10
YOR197W	31
YOR198C	18
YOR199W	43
YOR200W	137
YOR201C	17
YOR202W	13
YOR203W	31
YOR204W	28
YOR205C	235
YOR206W	26
YOR207C	27
YOR208W	30
YOR209C	67
YOR210W	17
YOR211C	4
YOR212W	51
YOR213C	47
YOR214C	161
YOR215C	29
YOR216C	24
YOR217W	10
YOR218C	138
YOR219C	134
YOR220W	90
YOR221C	146
YOR222W	14
YOR223W	166
YOR224C	100
YOR225W	28
YOR226C	18
YOR227W	44
YOR228C	18
YOR229W	14
YOR230W	65
YOR231C-A	110
YOR231W	54
YOR232W	76
YOR233W	47
YOR234C	65
YOR235W	59
YOR236W	55

YOR237W	17
YOR238W	24
YOR239W	24
YOR241W	46
YOR242C	129
YOR243C	41
YOR244W	20
YOR245C	254
YOR246C	43
YOR247W	33
YOR248W	48
YOR249C	239
YOR250C	96
YOR251C	531
YOR252W	31
YOR253W	31
YOR254C	142
YOR255W	44
YOR256C	37
YOR257W	9
YOR258W	144
YOR259C	210
YOR260W	57
YOR261C	27
YOR262W	275
YOR263C	16
YOR264W	109
YOR265W	46
YOR266W	10
YOR267C	24
YOR268C	126
YOR269W	83
YOR270C	197
YOR271C	40
YOR272W	324
YOR273C	22
YOR274W	88
YOR275C	63
YOR276W	72
YOR277C	11
YOR278W	58
YOR279C	34
YOR280C	48
YOR281C	198
YOR282W	87
YOR283W	88
YOR284W	2
YOR285W	6
YOR286W	408
YOR287C	76
YOR288C	12
YOR289W	105
YOR290C	18
YOR291W	196
YOR292C	6

YOR293C-A	15
YOR293W	70
YOR294W	128
YOR295W	30
YOR296W	77
YOR297C	52
YOR298C-A	33
YOR298W	27
YOR299W	18
YOR300W	23
YOR301W	120
YOR302W	51
YOR303W	15
YOR304C-A	79
YOR304W	13
YOR305W	25
YOR306C	74
YOR307C	26
YOR308C	22
YOR309C	69
YOR310C	55
YOR311C	42
YOR312C	24
YOR313C	232
YOR314W	383
YOR314W-A	388
YOR315W	35
YOR316C	63
YOR316C-A	5
YOR317W	132
YOR318C	147
YOR319W	57
YOR320C	48
YOR321W	51
YOR322C	85
YOR323C	92
YOR324C	48
YOR325W	21
YOR326W	120
YOR327C	28
YOR328W	171
YOR329C	66
YOR329W-A	23
YOR330C	7
YOR331C	12
YOR332W	27
YOR333C	38
YOR334W	43
YOR335C	11
YOR335W-A	11
YOR336W	27
YOR337W	55
YOR338W	7
YOR339C	29
YOR340C	31

YOR341W	4
YOR342C	3
YOR343C	32
YOR343W-A	118
YOR343W-B	36
YOR344C	102
YOR345C	108
YOR346W	205
YOR347C	32
YOR348C	15
YOR349W	222
YOR350C	225
YOR351C	27
YOR352W	76
YOR353C	22
YOR354C	183
YOR355W	26
YOR356W	76
YOR357C	82
YOR358W	12
YOR359W	30
YOR360C	39
YOR361C	30
YOR362C	11
YOR363C	9
YOR364W	16
YOR365C	151
YOR366W	35
YOR367W	14
YOR368W	10
YOR369C	25
YOR370C	102
YOR371C	46
YOR372C	34
YOR373W	111
YOR374W	20
YOR375C	108
YOR376W	118
YOR376W-A	79
YOR377W	16
YOR378W	29
YOR379C	34
YOR380W	17
YOR381W	105
YOR381W-A	4
YOR382W	372
YOR383C	15
YOR384W	202
YOR385W	153
YOR386W	10
YOR387C	52
YOR388C	95
YOR389W	7
YOR390W	13
YOR391C	12

YOR392W	41
YOR393W	165
YOR394C-A	119
YOR394W	28
YOR396C-A	18
YOR396W	28
YPL001W	10
YPL002C	59
YPL003W	110
YPL004C	86
YPL005W	56
YPL006W	36
YPL007C	32
YPL008W	71
YPL009C	24
YPL010W	10
YPL011C	14
YPL012W	44
YPL013C	2
YPL014W	263
YPL015C	10
YPL016W	204
YPL017C	60
YPL018W	12
YPL019C	7
YPL020C	66
YPL021W	403
YPL022W	60
YPL023C	96
YPL024W	7
YPL025C	144
YPL026C	231
YPL027W	73
YPL028W	50
YPL029W	29
YPL030W	19
YPL031C	50
YPL032C	46
YPL033C	56
YPL034W	17
YPL035C	17
YPL036W	204
YPL037C	97
YPL038W	11
YPL038W-A	3
YPL039W	97
YPL040C	59
YPL041C	5
YPL042C	43
YPL043W	34
YPL044C	185
YPL045W	2
YPL046C	11
YPL047W	27
YPL048W	65

YPL049C	18
YPL050C	14
YPL051W	35
YPL052W	196
YPL053C	355
YPL054W	159
YPL055C	184
YPL056C	53
YPL057C	20
YPL058C	19
YPL059W	39
YPL060C-A	19
YPL060W	82
YPL061W	191
YPL062W	24
YPL063W	68
YPL064C	59
YPL065W	43
YPL066W	152
YPL067C	53
YPL068C	148
YPL069C	180
YPL070W	32
YPL071C	5
YPL072W	54
YPL073C	15
YPL074W	103
YPL075W	90
YPL076W	131
YPL077C	63
YPL078C	35
YPL079W	60
YPL080C	107
YPL081W	18
YPL082C	29
YPL083C	34
YPL084W	3
YPL085W	58
YPL086C	11
YPL087W	166
YPL088W	157
YPL089C	18
YPL090C	174
YPL091W	5
YPL092W	67
YPL093W	12
YPL094C	74
YPL095C	50
YPL096C-A	12
YPL096W	385
YPL097W	45
YPL098C	53
YPL099C	334
YPL100W	66
YPL101W	4

YPL102C	152
YPL103C	21
YPL104W	69
YPL105C	4
YPL106C	117
YPL107W	51
YPL108W	107
YPL109C	66
YPL110C	11
YPL111W	38
YPL112C	28
YPL113C	35
YPL114W	407
YPL115C	7
YPL116W	19
YPL117C	55
YPL118W	10
YPL119C	3
YPL119C-A	69
YPL120W	17
YPL121C	64
YPL122C	30
YPL123C	23
YPL124W	57
YPL125W	14
YPL126W	13
YPL127C	12
YPL128C	56
YPL129W	165
YPL130W	54
YPL131W	253
YPL132W	56
YPL133C	39
YPL134C	89
YPL135C-A	6
YPL135W	133
YPL136W	23
YPL137C	13
YPL138C	34
YPL139C	41
YPL140C	60
YPL141C	8
YPL142C	28
YPL143W	39
YPL144W	44
YPL145C	40
YPL146C	94
YPL147W	49
YPL148C	183
YPL149W	493
YPL150W	32
YPL151C	21
YPL152W	30
YPL152W-A	47
YPL153C	274

YPL154C	16
YPL155C	12
YPL156C	101
YPL157W	197
YPL158C	26
YPL159C	56
YPL160W	1
YPL161C	43
YPL162C	16
YPL163C	25
YPL164C	19
YPL165C	17
YPL166W	12
YPL167C	179
YPL168W	265
YPL169C	38
YPL170W	16
YPL171C	160
YPL172C	28
YPL173W	21
YPL174C	50
YPL175W	306
YPL176C	10
YPL177C	24
YPL178W	25
YPL179W	11
YPL180W	91
YPL181W	67
YPL182C	1
YPL183C	50
YPL183W-A	129
YPL184C	19
YPL185W	13
YPL186C	23
YPL187W	57
YPL188W	210
YPL189C-A	20
YPL189W	35
YPL190C	1
YPL191C	50
YPL192C	14
YPL193W	89
YPL194W	49
YPL195W	21
YPL196W	23
YPL197C	27
YPL198W	9
YPL199C	49
YPL200W	56
YPL201C	16
YPL202C	21
YPL203W	207
YPL204W	159
YPL205C	51
YPL206C	230

YPL207W	21
YPL208W	78
YPL209C	27
YPL210C	91
YPL211W	37
YPL212C	95
YPL213W	78
YPL214C	32
YPL215W	16
YPL216W	11
YPL217C	7
YPL218W	21
YPL219W	101
YPL220W	81
YPL221W	77
YPL222C-A	12
YPL222W	26
YPL223C	8
YPL224C	424
YPL225W	18
YPL226W	112
YPL227C	51
YPL228W	33
YPL229W	489
YPL230W	13
YPL231W	44
YPL232W	35
YPL233W	10
YPL234C	38
YPL235W	33
YPL236C	19
YPL237W	23
YPL238C	153
YPL239W	13
YPL240C	37
YPL241C	21
YPL242C	108
YPL243W	48
YPL244C	8
YPL245W	13
YPL246C	121
YPL247C	97
YPL248C	87
YPL249C	7
YPL249C-A	53
YPL250C	50
YPL250W-A	100
YPL251W	13
YPL252C	14
YPL253C	45
YPL254W	25
YPL255W	449
YPL256C	484
YPL257W	372
YPL257W-A	8

YPL257W-B	57
YPL258C	46
YPL259C	559
YPL260W	85
YPL261C	105
YPL262W	22
YPL263C	142
YPL264C	4
YPL265W	20
YPL266W	1009
YPL267W	235
YPL268W	30
YPL269W	87
YPL270W	142
YPL271W	105
YPL272C	39
YPL273W	71
YPL274W	9
YPL275W	11
YPL276W	18
YPL277C	446
YPL278C	23
YPL279C	4
YPL280W	20
YPL281C	37
YPL282C	3
YPL283C	10
YPL283W-A	28
YPL283W-B	89
YPR001W	356
YPR002C-A	122
YPR002W	38
YPR003C	84
YPR004C	44
YPR005C	19
YPR006C	16
YPR007C	33
YPR008W	124
YPR009W	56
YPR010C	25
YPR011C	9
YPR012W	32
YPR013C	21
YPR014C	30
YPR015C	5
YPR016C	492
YPR016W-A	132
YPR017C	109
YPR018W	44
YPR019W	26
YPR020W	9
YPR021C	36
YPR022C	6
YPR023C	98
YPR024W	9

YPR025C	144
YPR026W	37
YPR027C	237
YPR028W	57
YPR029C	8
YPR030W	162
YPR031W	66
YPR032W	37
YPR033C	38
YPR034W	54
YPR035W	44
YPR036W	117
YPR036W-A	7
YPR037C	21
YPR038W	79
YPR039W	30
YPR040W	28
YPR041W	38
YPR042C	64
YPR043W	125
YPR044C	122
YPR045C	31
YPR046W	171
YPR047W	46
YPR048W	76
YPR049C	54
YPR050C	79
YPR051W	56
YPR052C	22
YPR053C	241
YPR054W	3
YPR055W	38
YPR056W	30
YPR057W	32
YPR058W	30
YPR059C	393
YPR060C	4
YPR061C	43
YPR062W	144
YPR063C	148
YPR064W	29
YPR065W	26
YPR066W	59
YPR067W	41
YPR068C	180
YPR069C	22
YPR070W	201
YPR071W	20
YPR072W	30
YPR073C	14
YPR074C	57
YPR074W-A	16
YPR075C	9
YPR076W	26
YPR077C	10

YPR078C	270
YPR079W	56
YPR080W	52
YPR081C	5
YPR082C	79
YPR083W	38
YPR084W	15
YPR085C	78
YPR086W	7
YPR087W	11
YPR088C	348
YPR089W	369
YPR091C	38
YPR092W	4
YPR093C	46
YPR094W	88
YPR095C	169
YPR096C	332
YPR097W	617
YPR098C	30
YPR099C	9
YPR100W	81
YPR101W	1127
YPR102C	116
YPR103W	28
YPR104C	29
YPR105C	273
YPR106W	32
YPR107C	30
YPR108W	26
YPR108W-A	388
YPR109W	33
YPR110C	6
YPR111W	29
YPR112C	85
YPR113W	287
YPR114W	38
YPR115W	123
YPR116W	55
YPR117W	257
YPR118W	26
YPR119W	24
YPR120C	57
YPR121W	156
YPR122W	70
YPR123C	93
YPR124W	78
YPR125W	25
YPR126C	172
YPR127W	24
YPR128C	8
YPR129W	33
YPR130C	23
YPR131C	32
YPR132W	337

YPR133C	40
YPR133W-A	13
YPR134W	51
YPR135W	42
YPR136C	78
YPR137C-A	162
YPR137C-B	15
YPR137W	76
YPR138C	7
YPR139C	155
YPR140W	9
YPR141C	45
YPR142C	7
YPR143W	43
YPR144C	18
YPR145C-A	121
YPR145W	48
YPR146C	40
YPR147C	66
YPR148C	49
YPR149W	3
YPR150W	26
YPR151C	14
YPR152C	107
YPR153W	7
YPR154W	78
YPR155C	64
YPR156C	123
YPR157W	74
YPR158C-C	26
YPR158C-D	72
YPR158W	17
YPR158W-A	45
YPR158W-B	58
YPR159C-A	9
YPR159W	10
YPR160C-A	83
YPR160W	99
YPR160W-A	18
YPR161C	20
YPR162C	118
YPR163C	5
YPR164W	358
YPR165W	27
YPR166C	46
YPR167C	45
YPR168W	12
YPR169W	50
YPR169W-A	51
YPR170C	30
YPR170W-A	50
YPR170W-B	26
YPR171W	23
YPR172W	145
YPR173C	240

YPR174C	16
YPR175W	37
YPR176C	173
YPR177C	30
YPR178W	63
YPR179C	331
YPR180W	14
YPR181C	24
YPR182W	44
YPR183W	105
YPR184W	15
YPR185W	37
YPR186C	99
YPR187W	36
YPR188C	27
YPR189W	121
YPR190C	491
YPR191W	131
YPR192W	25
YPR193C	9
YPR194C	80
YPR195C	21
YPR196W	4
YPR197C	33
YPR198W	27
YPR199C	9
YPR200C	41
YPR201W	32
YPR202W	161
YPR203W	58
YPR204C-A	20
YPR204W	61
Q0010	13
Q0017	14
Q0032	5
Q0045	100
Q0050	98
Q0055	8
Q0060	1
Q0065	138
Q0070	10
Q0075	56
Q0080	49
Q0085	201
Q0092	27
Q0105	240
Q0110	16
Q0115	38
Q0120	100
Q0130	44
Q0140	120
Q0142	93
Q0143	16
Q0144	55
Q0160	182

Q0182	56
Q0250	27
Q0255	73
Q0275	25
Q0297	123
R0010W	7
R0020C	8
R0030W	11
R0040C	43
